# Anthracycline-containing and taxane-containing chemotherapy for early-stage operable breast cancer: a patient-level meta-analysis of 100 000 women from 86 randomised trials

**DOI:** 10.1016/S0140-6736(23)00285-4

**Published:** 2023-04-15

**Authors:** Early Breast Cancer Trialists’ Collaborative Group (EBCTCG)

## Abstract

**Background:**

Anthracycline–taxane chemotherapy for early-stage breast cancer substantially improves survival compared with no chemotherapy. However, concerns about short-term and long-term side-effects of anthracyclines have led to increased use of taxane chemotherapy without anthracycline, which could compromise efficacy. We aimed to better characterise the benefits and risks of including anthracycline, and the comparative benefits of different anthracycline–taxane regimens.

**Methods:**

We did an individual patient-level meta-analysis of randomised trials comparing taxane regimens with versus without anthracycline, and updated our previous meta-analysis of anthracycline regimens with versus without taxane, as well as analysing 44 trials in six related comparisons. We searched databases, including MEDLINE, Embase, the Cochrane Library, and meeting abstracts to identify trials assessing anthracycline and taxane chemotherapy. Adjuvant or neoadjuvant trials were eligible if they began before Jan 1, 2012. Primary outcomes were breast cancer recurrence and cause-specific mortality. Log-rank analyses yielded first-event rate ratios (RRs) and CIs.

**Findings:**

28 trials of taxane regimens with or without anthracycline were identified, of which 23 were deemed eligible, and 15 provided data on 18 103 women. Across all 15 trials that provided individual data, recurrence rates were 14% lower on average (RR 0·86, 95% CI 0·79–0·93; p=0·0004) with taxane regimens including anthracycline than those without. Non-breast cancer deaths were not increased but there was one additional acute myeloid leukaemia case per 700 women treated. The clearest reductions in recurrence were found when anthracycline was added concurrently to docetaxel plus cyclophosphamide versus the same dose of docetaxel plus cyclophosphamide (10-year recurrence risk 12·3% *vs* 21·0%; risk difference 8·7%, 95% CI 4·5–12·9; RR 0·58, 0·47–0·73; p<0·0001). 10-year breast cancer mortality in this group was reduced by 4·2% (0·4–8·1; p=0·0034). No significant reduction in recurrence risk was found for sequential schedules of taxane plus anthracycline when compared with docetaxel plus cyclophosphamide (RR 0·94, 0·83–1·06; p=0·30). For the analysis of anthracycline regimens with versus without taxane, 35 trials (n=52 976) provided individual patient data. Larger recurrence reductions were seen from adding taxane to anthracycline regimens when the cumulative dose of anthracycline was the same in each group (RR 0·87, 0·82–0·93; p<0·0001; n=11 167) than in trials with two-fold higher cumulative doses of non-taxane (mostly anthracycline) in the control group than in the taxane group (RR 0·96, 0·90–1·03; p=0·27; n=14 620). Direct comparisons between anthracycline and taxane regimens showed that a higher cumulative dose and more dose-intense schedules were more efficacious. The proportional reductions in recurrence for taxane plus anthracycline were similar in oestrogen receptor-positive and oestrogen receptor-negative disease, and did not differ by age, nodal status, or tumour size or grade.

**Interpretation:**

Anthracycline plus taxane regimens are most efficacious at reducing breast cancer recurrence and death. Regimens with higher cumulative doses of anthracycline plus taxane provide the greatest benefits, challenging the current trend in clinical practice and guidelines towards non-anthracycline chemotherapy, particularly shorter regimens, such as four cycles of docetaxel–cyclophosphamide. By bringing together data from almost all relevant trials, this meta-analysis provides a reliable evidence base to inform individual treatment decisions, clinical guidelines, and the design of future clinical trials.

**Funding:**

Cancer Research UK, UK Medical Research Council.

## Introduction

Cytotoxic chemotherapy for early-stage, operable breast cancer substantially reduces the risk of recurrence and death. Meta-analyses by the Early Breast Cancer Trialists’ Collaborative Group (EBCTCG) have shown 20–25% proportional reductions in breast cancer mortality with cyclophosphamide plus methotrexate plus fluorouracil, or with four cycles of anthracycline plus cyclophosphamide, compared with no chemotherapy.^1,2^ Adding taxane (docetaxel or paclitaxel) to anthracycline-containing chemotherapy, or substantially increasing the cumulative dose of anthracycline, reduces breast cancer mortality by a further 10–15%.^3^ An additional benefit can be achieved by increasing the dose intensity of chemotherapy.^4^ The results from these meta-analyses indicate that, compared with no chemotherapy, anthracycline plus taxane chemotherapy can reduce breast cancer mortality rates by about 40% during the first decade after diagnosis, with similar proportional reductions irrespective of patient age or tumour characteristics, including size, grade, nodal involvement, hormone receptor status, and expression of HER2.

However, the optimal use of chemotherapy is uncertain. Anthracyclines increase the long-term risk of cardiovascular disease and acute myeloid leukaemia,^5–7^ and dose-dependent peripheral neuropathy is problematic with taxanes.^8^ Hence, particularly for women with low-risk tumours treated with optimal surgery or radiotherapy (and endocrine or anti-HER2 therapy when appropriate), the benefits of anthracycline plus taxane chemotherapy might be insufficient to outweigh the risks compared with less intensive or no chemotherapy. This patient-level meta-analysis aimed to better characterise the benefits and risks of different taxane and anthracycline chemotherapy regimens for early-stage breast cancer.

## Methods

### Search strategy and selection criteria

Methods of identifying trials, data collection, checking, analysis, and presentation for this patient-level meta-analysis are as described in previous EBCTCG reports,^2,3,9,10^ and conform to PRISMA guidelines (individual patient data).^11^

Briefly, we searched databases, including MEDLINE, Embase, the Cochrane Library, and meeting abstracts, to identify trials in any language assessing anthracycline and taxane chemotherapy (appendix pp 92–94). Randomised trials of adjuvant or neoadjuvant chemotherapy were eligible if they had mature follow-up data (ie, began before Jan 1, 2012). The last search was done in September, 2022. We focused on trials comparing taxane-based regimens with versus without anthracycline, which had not previously been subject to EBCTCG meta-analysis. We also updated the EBCTCG meta-analysis of anthracycline-based chemotherapy with versus without taxane.^3^ Further meta-analyses compared anthracycline versus taxane chemotherapy, paclitaxel versus docetaxel (trials of nab-paclitaxel were not included as most started after 2012), taxane-dose fractionation (eg, giving drugs once-weekly at approximately a third of the dose used in a 3-weekly regimen), the sequence of administration of taxanes and anthracyclines, longer versus shorter anthracycline regimens, and higher-dose versus lower-dose anthracycline-based chemotherapy. The EBCTCG secretariat did the searches; the eligibility of each study for a particular meta-analysis was initially determined by RB, RG, RKH, and JBr, and finalised through presentation and discussion of the findings with the writing committee, the EBCTCG steering committee, and the broader membership of the EBCTCG. There was no data extraction; instead, individual patient data were provided. Individual patient data were sought from trial groups during 2018–21 and included randomisation date, allocated treatment, age, menopausal status, BMI, tumour diameter, grade, histology, spread to locoregional lymph nodes, oestrogen receptor (ER) status, progesterone receptor status, HER2 status, cell proliferation (Ki-67), dates and sites of any breast cancer recurrence or other second primary cancer, and the date and underlying cause of any death. The trials in these meta-analyses were mostly conducted before genomic profiles were routinely available; therefore, no data were available to collect from trialists. The statistical analysis plan is provided in the appendix (pp 61–91).

### Data analysis

The primary outcomes were recurrence of invasive breast cancer (distant, locoregional, or new primary in the contralateral breast), breast cancer mortality, death without recurrence, and all-cause mortality. Prespecified primary subgroup investigations were by follow-up period (years 0–1, 2–4, 5–9, ≥10), site of recurrence, age, BMI, ER and progesterone receptor status, nodal status, tumour diameter, tumour grade, tumour histology (ductal, lobular), HER2 status, and cell proliferation index (Ki-67 <10%, 10% to <20%, 20% to <50%, ≥50%).

Statistical methods (stratified log-rank analyses and Kaplan-Meier graphs) are as described in previous EBCTCG reports,^2,3,9,10^ and in the statistical analysis plan (appendix pp 61–91). Time-to-event analyses were stratified by age, ER status, nodal status (unless neoadjuvant chemotherapy was administered), year of follow-up, and trial. Analyses included all women, regardless of treatment compliance (intention-to-treat analyses). Log-rank analyses were used to estimate the first-event rate ratio (RR); 95% CIs were used for meta-analyses, and 99% CIs for individual trials or subgroups. Breast cancer mortality RRs were estimated by subtracting log-rank analyses of mortality without recurrence from those of overall mortality, avoiding the need to determine which deaths after recurrence were from breast cancer.^2^ For each comparison, forest plots and Kaplan-Meier graphs describe the separate trials and their results, and subgroup analyses used χ^2^ tests for heterogeneity or trend to explore whether proportional reductions varied by trial, or by patient or tumour-related characteristics. Statistical analyses used in-house Fortran programs.

### Role of the funding source

The funders of the study had no role in the study design, data collection, data analysis, data interpretation, or writing of the report.

## Results

28 trials of taxane regimens with anthracycline versus without anthracycline were identified, of which five were deemed ineligible (appendix p 3). Patient-level data were provided for 15 of the 23 eligible trials, including 18 103 (93·2%) of 19 434 women receiving taxane regimens with anthracycline or without anthracycline (appendix pp 4–5). In trials providing data, the median participant age was 53 years (IQR 46–60), and of 18 103 women, 9731 (53%) had cancers with lymph node involvement, 12 244 (67%) had ER-positive tumours, and 2577 (14%) had HER2-positive tumours. Almost all women with HER2-positive disease were scheduled to receive trastuzumab as allocated treatment,^12,13^ or as the local standard of care.^14–16^ Median follow-up was 5·4 years (IQR 4·5–6·9).

The non-anthracycline comparator in 11 of the 15 eligible trials for which patient data were provided (13 855 [76·5%] of 18 103 women; appendix pp 61–91) was docetaxel 75 mg/m^2^ plus cyclophosphamide 600 mg/m^2^, administered once every 3 weeks for six cycles. Three of these 11 trials (n=2469) assessed administration of anthracycline given concurrently with docetaxel and cyclophosphamide; the only difference between the treatment groups was the addition of anthracycline (generally doxorubicin, cumulative dose 300 mg/m^2^) in each of the six cycles. The other eight (n=11 386) administered anthracycline and taxane cycles sequentially, mostly every 3 weeks for six or eight complete cycles. These regimens allowed a higher taxane dose to be administered per cycle. However, because only half of the cycles included taxane, the cumulative taxane dose was lower with sequential anthracycline plus taxane than with six cycles of docetaxel plus cyclophosphamide (cumulative docetaxel dose was typically 300 mg/m^2^ *vs* 450 mg/m^2^). For the same reason, the cumulative dose of anthracycline was also a third lower with sequential treatment than concurrent treatment, with most trials of sequential anthracycline plus taxane administering a cumulative dose of 300 mg/m^2^ epirubicin, which is considered biologically equivalent to 200 mg/m^2^ doxorubicin.^17^ The National Surgical Adjuvant Breast and Bowel Project B-49^18^ permitted investigators a choice of anthracycline-containing schedules, including doxorubicin plus docetaxel and cyclophosphamide, but was included in the sequential group because only 112 (12%) of 932 participants received six cycles of anthracycline plus docetaxel plus cyclophosphamide.

**Figure 1:**
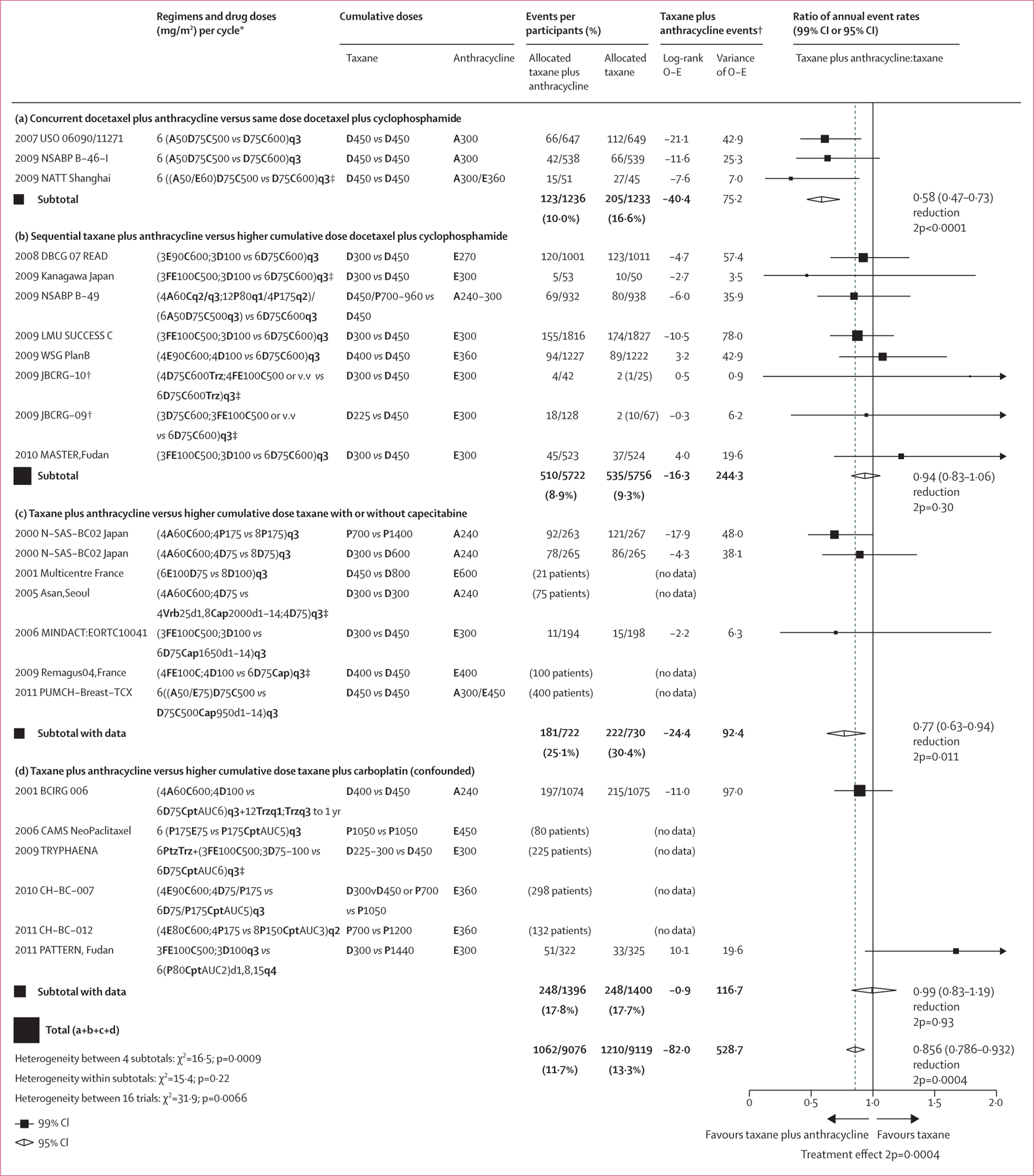
Recurrence of breast cancer (first invasive local, distant, or new contralateral primary) in the 15 trials with patient-level data comparing taxane plus anthracycline versus taxane without anthracycline 24 trials in total. One trial (N-SAS-BC 02) is shown on two lines as it was a 2 X 2 trial. Eight trials did not provide data. Taxanes were D and P. Anthracyclines were A and E. Other agents were C, F, M, Trz, Vrb, Cap, Cpt, and Ptz. 99% CIs are provided for individual trial data; 95% CIs are provided for subtotal and total data. A=doxorubicin. AUC=area under the curve. C=cyclophosphamide. Cap=capecitabine. Cpt=carboplatin. D=docetaxel. d=day of cycle. E=epirubicin. F=fluorouracil. M=methotrexate. O–E=observed minus expected. P=paclitaxel. Ptz=pertuzumab. q1=weekly. q2=every 2 weeks. q3=every 3 weeks. q4=every 4 weeks. Trz=trastuzumab. Vrb=vinorelbine. v.v=vice versa. yr=year. 2p=two-sided p value. *Any unstated doses are the same as for the non-anthracycline comparator. The regimens being compared in each study are described by the number of cycles, the drug abbreviation and dose in mg/m², and the frequency of the doses; a solidus (/) indicates or; a semicolon indicates then (sequential treatment). †For balance, control patients in three-way trials or trial strata count half or twice in subtotal(s) and in the final total of events and patients. ‡Pre-operative chemotherapy.

Two eligible trials^15,19^ (n=1452) compared sequential anthracycline plus taxane with taxane-based schedules other than six cycles of docetaxel plus cyclophosphamide, either single-agent docetaxel or paclitaxel, or docetaxel plus capecitabine. Another two eligible trials^12,20^ compared sequential anthracycline plus taxane with concurrent taxane plus carboplatin in specific tumour subtypes: HER2-positive tumours in the BCIRG-006 trial (n=2149)^12^ and triple-negative tumours in the PATTERN trial (n=647).^20^ Again, with sequential administration, cumulative taxane doses were lower with anthracycline plus taxane than with the non-anthracycline comparator, and cumulative anthracycline doses were lower than with concurrent anthracycline plus taxane (doses for each trial are provided in figure 1). The eight small trials that have not provided data (appendix pp 64–67), including 1331 (6·8%) of all 19 434 women randomly assigned, compared taxane plus anthracycline with taxane regimens other than six cycles of docetaxel plus cyclophosphamide, four as a neoadjuvant treatment.

**Figure 2:**
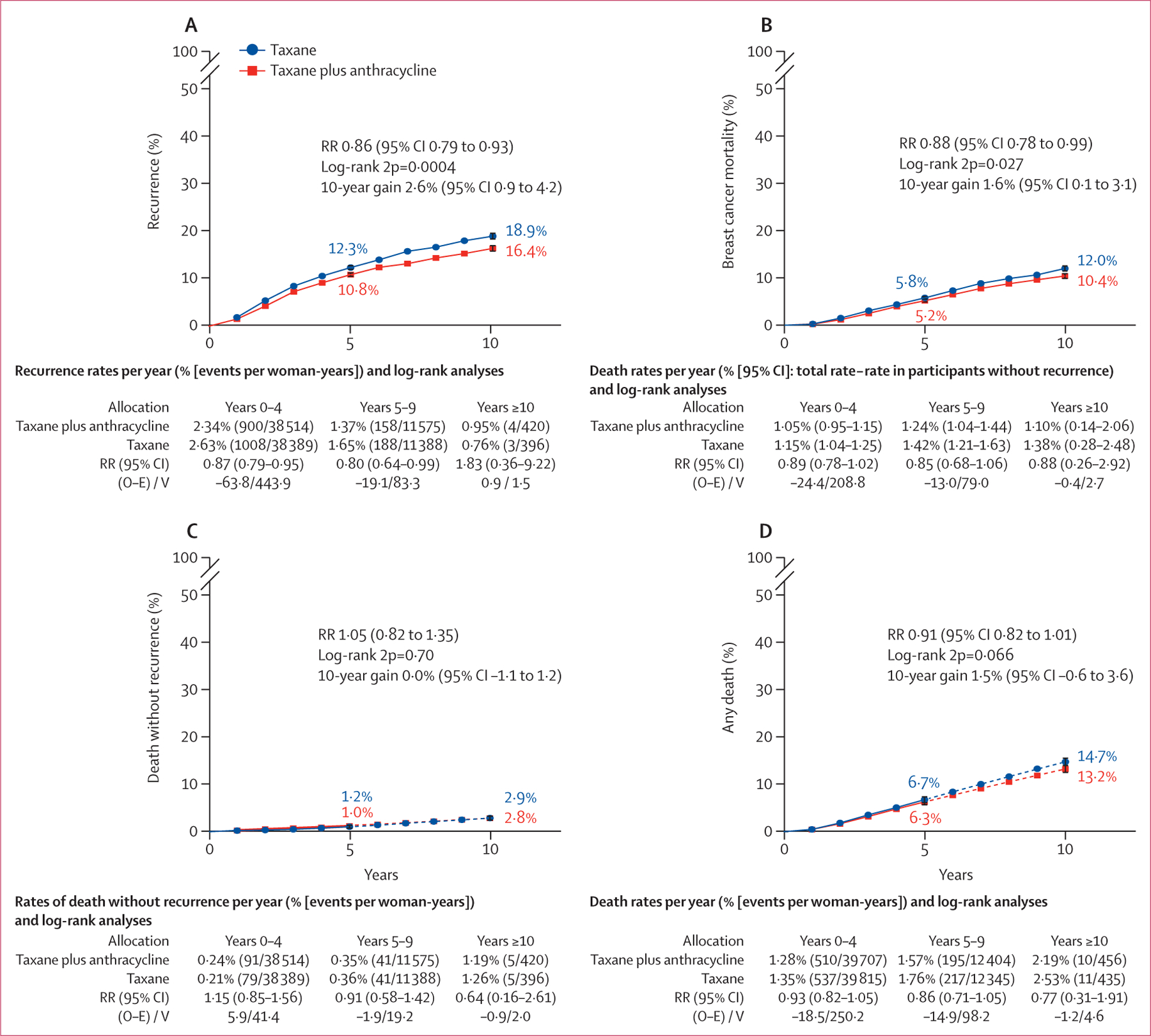
10-year cumulative risk of outcomes with taxane plus anthracycline versus taxane without anthracycline All analyses included 18 103 participants (9076 in taxane plus anthracycline group, 9027 in taxane only group). 10-year cumulative risk of any invasive recurrence (A), breast cancer mortality (B), death without recurrence (C)*, and any death (D)*. Error bars show 95% CI. O–E=observed minus expected. RR=rate ratio. V=variance. 2p=two-sided p value. *Smoothed after 5 years (denoted by dotted line).

Results for recurrence of any first invasive breast cancer are shown in figure 1. For each trial contributing to this meta-analysis, the information included the year recruitment started, trial name, chemotherapy schedules, cumulative doses of taxane and anthracycline, log-rank statistics, and the ratio of annual event rates. Similar plots for distant, locoregional, and contralateral recurrence, breast cancer mortality, death without recurrence (in year 0 and overall), and all-cause mortality are shown in the appendix (pp 7–13). Kaplan Meier plots of pooled analyses for invasive recurrence, breast cancer mortality, death without recurrence, and any death are shown in figure 2. Across all eligible trials that provided data, patients assigned to anthracycline plus taxane had a 14% lower rate of breast cancer recurrence than did patients assigned to taxane without anthracycline; the 10-year absolute risk reduction was 2·6% ([95% CI 0·9–4·2] 16·4% *vs* 18·9%). The annual rate of breast cancer death was reduced by 12%. The 10-year absolute risk reduction was 1·6% ([0·1–3·1] 10·4% anthracycline plus taxane *vs* 12·0% taxane) with no increase in deaths without recurrence. All-cause mortality was also reduced but not significantly.

**Figure 3:**
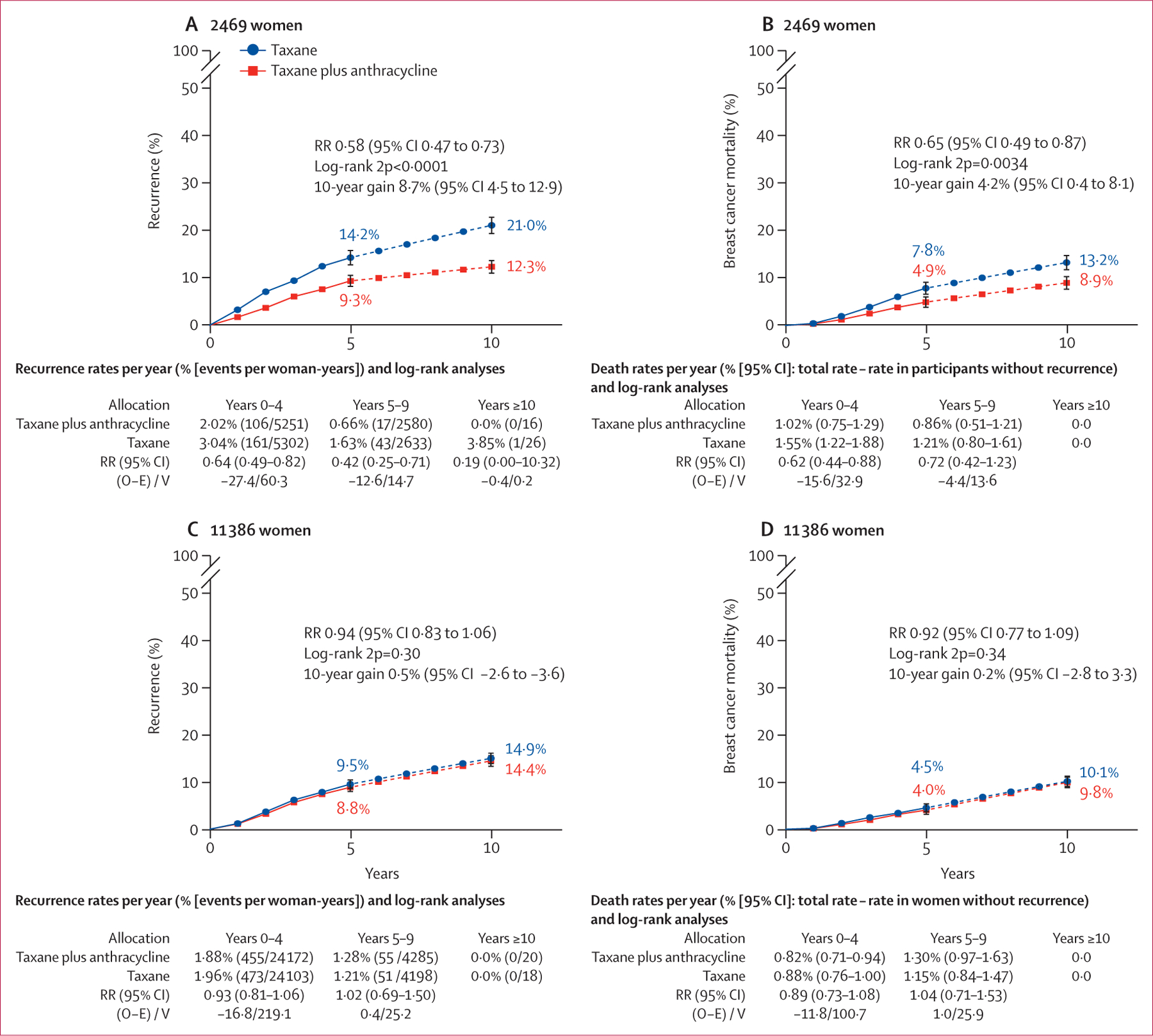
10-year cumulative risk of any recurrence and breast cancer mortality in patients on taxane-based regimens with anthracycline versus without anthracycline Risk of recurrence (A) or breast cancer mortality (B) in 2469 patients on concurrent anthracycline plus docetaxel plus cyclophosphamide (n=1236) versus same cumulative dose docetaxel plus cyclophosphamide (n=1233), and risk of recurrence (C) or breast cancer mortality (D) in 11 386 patients on sequential anthracycline and taxane (n=5722) versus higher cumulative dose docetaxel plus cyclophosphamide (n=5664), smoothed after 5 years (denoted by dotted line). Error bars show 95% CI. O–E=observed minus expected. RR=rate ratio. V=variance. 2p=two-sided p value.

There was heterogeneity (p=0·0009) in recurrence reductions between the four sets of trial comparisons, with the additional benefit of anthracycline most clearly seen in three trials comparing anthracycline plus docetaxel plus cyclophosphamide versus the same dose of docetaxel plus cyclophosphamide, both administered every 3 weeks for six cycles. With the addition of concurrent anthracycline, the 10-year recurrence risk was substantially reduced (8·7%, 95% CI 4·5–12·9) as was 10-year breast cancer mortality (4·2%, 0·4–8·1; figure 3A, B).

In eight trials comparing regimens of sequential anthracycline plus taxane versus six cycles of the same dose docetaxel and cyclophosphamide,^13,14,18,21–24^ there was no significant difference in recurrence risk (figure 3C) or breast cancer mortality (figure 3D). Similarly, less benefit from anthracycline was seen in trials that compared sequential anthracycline–taxane chemotherapy with single-agent taxane or docetaxel–capecitabine-based schedules (figure 1C). No overall difference was apparent in trials comparing taxane plus anthracycline with taxane plus carboplatin (figure 1D); a modest reduction in recurrence in the BCIRG-06 trial was negated by an increase in recurrence with anthracycline in the PATTERN trial.^20^ This difference might be explained by the two-fold higher cumulative taxane dose and the inclusion of carboplatin in the non-anthracycline group.

**Figure 4:**
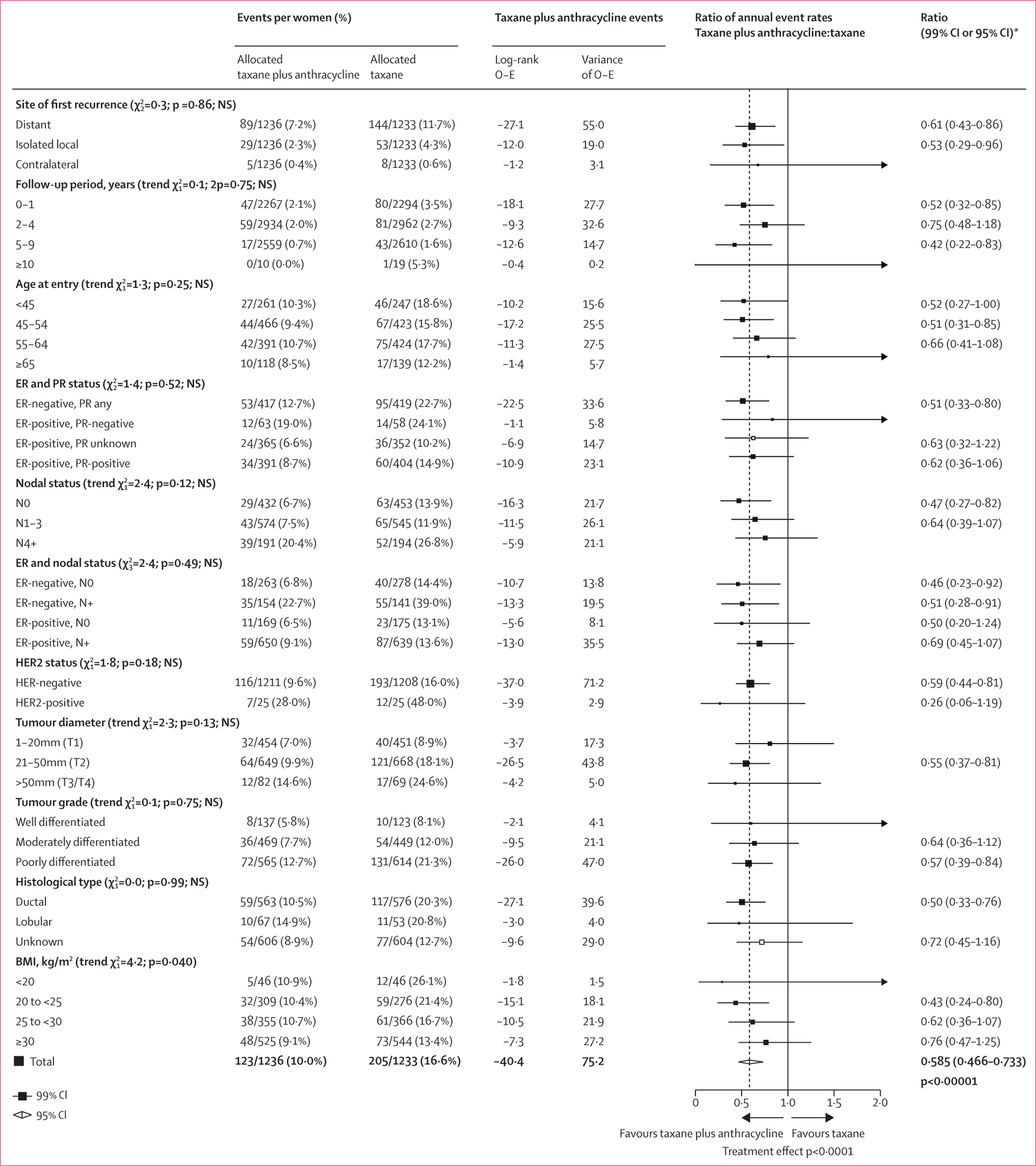
Subgroup analyses of recurrence with concurrent anthracycline plus docetaxel plus cyclophosphamide versus same dose docetaxel plus cyclophosphamide Subgroup analyses of first invasive recurrence of breast cancer. O–E=observed minus expected. ER=oestrogen receptor. NS=not significant. N0=node-negative. N+=node-positive. PR=progesterone receptor. 2p=two-sided p value.*99% CIs are provided for individual subgroup data; 95% CI is provided for the total data.

**Figure 5:**
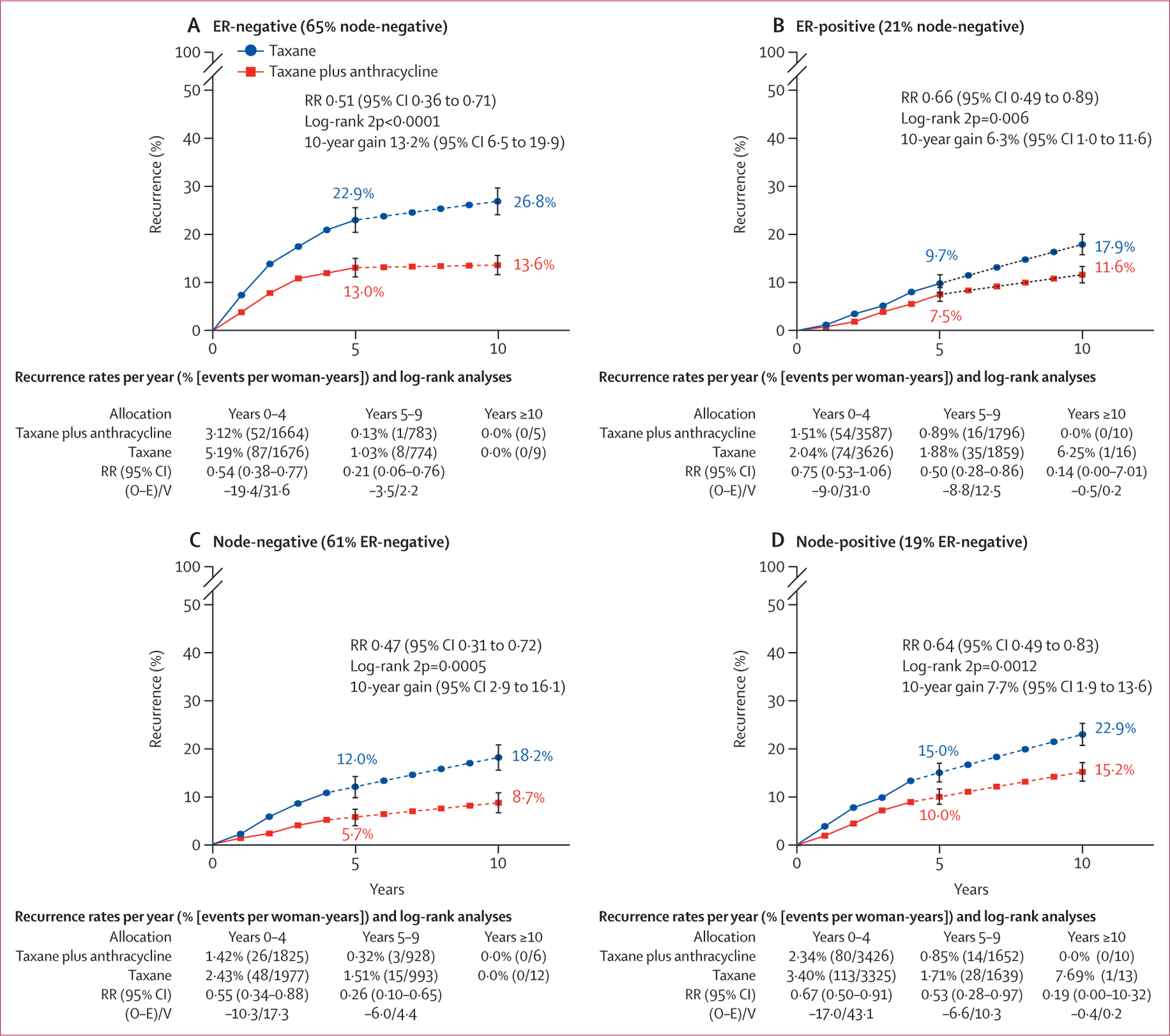
10-year cumulative risk of recurrence with concurrent anthracycline plus docetaxel plus cyclophosphamide versus same dose docetaxel plus cyclophosphamide, by oestrogen receptor and nodal status 10-year risk of any recurrence in 836 patients on concurrent anthracycline plus docetaxel plus cyclophosphamide (n=417) versus same dose docetaxel plus cyclophosphamide (n=419) who were ER-negative (A) and 1633 patients (819 *vs* 814) who were ER-positive (B), both smoothed after 5 years (dotted line), and in 885 patients (432 *vs* 453) with node-negative cancer (C) and 1584 patients (804 *vs 7*80) with node-positive cancer (D), both smoothed after 4 years (dotted line). Error bars show 95% CI. ER=oestrogen receptor. O–E=observed minus expected. RR=rate ratio. V=variance. 2p=two-sided p value.

The subgroup analyses for first invasive recurrence from the three trials that administered concurrent anthracycline with docetaxel and cyclophosphamide, which showed the clearest benefit of all treatment comparisons, are presented in figure 4. Similar subgroup analyses of distant recurrence and breast cancer mortality, and subgroup analyses for pooled data from all comparisons in figure 1, are shown in the appendix (pp 14–18). Most recurrences were distant metastases, which occurred at a lower rate in patients allocated to concurrent anthracycline with docetaxel and cyclophosphamide. Isolated locoregional recurrence was similarly reduced; there were too few new contralateral primary cancers for meaningful comparison (figure 4). The proportional reduction in recurrence persisted in years 0–1, 2–4, and 5–9 with little data beyond year 10. Rate reductions did not differ substantially by patient age (but few patients older than 65 years were included) or by pathological risk characteristics, such as tumour size, grade, and histological subtype, and nodal status. There was no significant difference (p=0·25) in recurrence rate reductions between ER-negative and ER-positive cancers. However, patients with ER-negative cancer had a higher risk of early recurrence than patients with ER-positive tumours (figure 5A, B). Figures 5C and 5D show similar proportional reductions in risk of recurrence by nodal status, with significant benefit in node-negative and node-positive cancer. As a higher proportion of node-negative tumours than node-positive tumours were ER-negative (541 [61%] of 885 *vs* 295 [19%] of 1584), 10-year recurrence rates and absolute benefits were similar. Too few patients had HER2-positive cancers for meaningful comparison of anthracycline efficacy in trials of anthracycline plus docetaxel plus cyclophosphamide versus the same dose of docetaxel plus cyclophosphamide. Just five^14,16,21,22,25^ of the 15 eligible trials with individual data collected included HER2-negative and HER2-positive tumours, thus allowing within-trial subgroup comparisons of efficacy by HER2 status. In these trials (n=267), there was no indication of differential efficacy by HER2 status (appendix pp 16–18). There was also no indication of differential efficacy by HER2 status in indirect comparisons between trials restricted to HER2-negative^18,20^ or HER2-positive tumours,^12,13^ although such comparisons are potentially misleading because of differences in chemotherapy regimens between trials (appendix pp 16–18). Information was insufficient for subgroup analysis of Ki-67 or *TOP2A*, or by genomic profiles.

For the combined analysis of all trials, there was no significant difference between treatment groups for death without recurrence (figure 2C), including from cardiovascular disease or other primary cancers. Deaths from non-breast cancer causes were only weakly related to tumour size or nodal status, suggesting few mortalities were misclassified as breast cancer deaths (appendix p 23). The overall incidence of new, non-breast primary cancers was also similar with and without anthracycline, although the incidence of acute myeloid leukaemia was increased with anthracycline. In trials with data, 12 (0·18%) of 6768 patients had acute myeloid leukaemia after anthracycline administration versus two (0·03%) of 6783 who did not receive anthracycline (p=0·013; appendix pp 21–22), equating to about one additional case of acute myeloid leukaemia per 700 women treated. Patient-level data on non-fatal toxicity were available for just two trials; these and selected toxicity data from trial publications are described in the appendix (pp 55–57). Few non-fatal cardiac events were reported, but in trials that included systematic investigations for asymptomatic disease more cardiac abnormalities were detected among women treated with anthracyclines than in those who did not have anthracyclines. In two trials (USOR 06–090 and NSABP B-46-I)^18^ of docetaxel plus cyclophosphamide with or without concurrent anthracycline reporting treatment toxicity, adverse events of grade 3 or worse were similar with and without anthracycline (580 [50·1%] of 1148 participants *vs* 581 [49·7%] of 1170 participants for any toxicity, 289 [25·2%] *vs* 320 [27·4%] for neutropenia, and 84 [7·3%] *vs* 77 [6·6%] for neutropenic sepsis; granulocyte colony stimulating factor prophylaxis was mandated in the anthracycline group only). Fatigue was more frequent with anthracycline than without anthracycline (80 [7·0%] *vs* 42 [3·6%]) and neuropathy of grade 2 or worse was less frequent (71 [6·2%] *vs* 87 [7·4%]).

**Figure 6:**
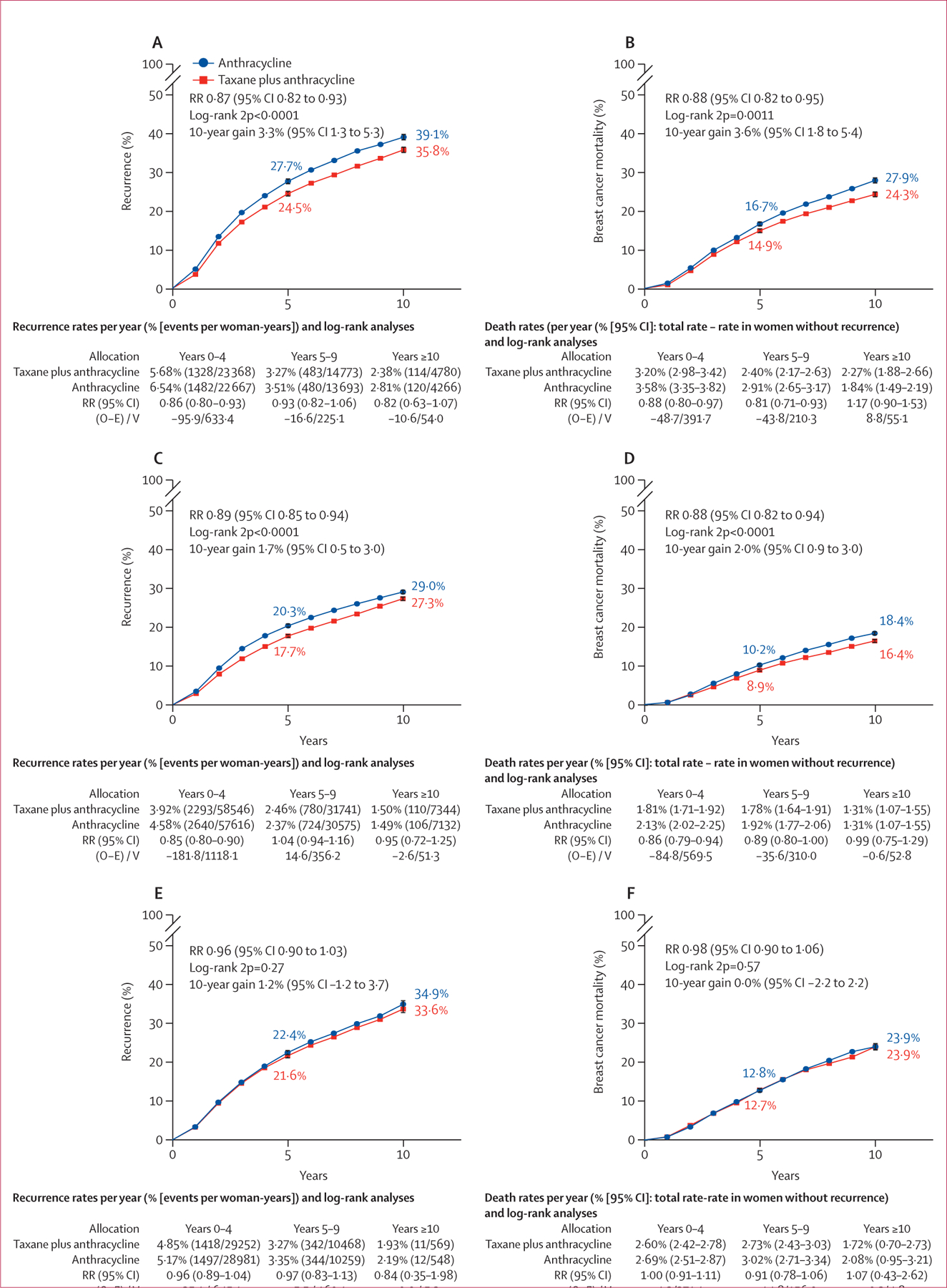
10-year cumulative risk of any recurrence and breast cancer mortality with anthracycline-based regimens with taxane versus without taxane Risk of recurrence (A) or breast cancer mortality (B) in 11 167 patients with anthracycline plus taxane (n=5590) versus the same chemotherapy without taxane (n=5577), risk of recurrence (C) or breast cancer mortality (D) in 27 089 patients with anthracycline plus taxane (n=13 528) versus a higher (but less than double) dose of non-taxane chemotherapy (n=13 561), and risk of recurrence (E) or breast cancer mortality (F) in 14 620 patients with taxane plus anthracycline (n=7322) versus double the dose of non-taxane chemotherapy (n=7298) . Error bars show 95% CI. O–E=observed minus expected. RR=rate ratio. V=variance. 2p=two-sided p value.

In an updated separate EBCTCG meta-analysis of trials, we assessed anthracycline-based regimens with versus without taxane (figure 6; appendix pp 24–25, 32–34).^3^ We identified 44 eligible trials, of which 35 provided patient-level data including 52 976 women. Nine trials did not have data. In trials assessing four cycles of taxane (paclitaxel or docetaxel) following a standard anthracycline regimen (11 167 participants in five trials; figure 6A), recurrence proportionally reduced by 13% with the addition of a taxane, translating to an absolute reduction of 3·3% (95% CI 1·3–5·3) in 10-year risk. With longer follow-up than in the previous meta-analysis, persistent reductions in breast cancer mortality were apparent in years 5–9, and the absolute reduction in 10-year breast cancer mortality was 3·6% (1·8–5·4; figure 6B). Less absolute benefit was seen for recurrence and breast cancer mortality when the cumulative dose of non-taxane in the comparator group was higher, but less than double that in the taxane group (figure 6C, D). When the cumulative dose of non-taxane chemotherapy was doubled in the control group (figure 6E, F), there was no significant difference in the rate of recurrence between groups.

In a separate meta-analysis of taxane regimens without anthracycline versus anthracycline regimens without taxane, we identified six eligible trials. Data were available from four of these six randomised trials (6019 [98·8%] of 6095 patients) comparing a taxane-based versus an anthracycline-based regimen (appendix pp 26, 35–37). The docetaxel trials compared regimens of treatment administered once every 3 weeks—either four cycles of docetaxel plus cyclophosphamide versus four cycles of doxorubicin plus cyclophosphamide or six cycles of docetaxel plus capecitabine versus six cycles of fluorouracil, epirubicin, and cyclophosphamide or fluorouracil, doxorubicin, and cyclophosphamide. A borderline significant reduction in recurrence favouring docetaxel was observed (RR 0·73, 95% CI 0·55–0·96; p=0·025). By contrast, recurrence rates were higher with paclitaxel, administered once a week as a single agent, than with doxorubicin plus cyclophosphamide or epirubicin administered every 2 weeks or 3 weeks (RR 1·30, 1·09–1·56; p=0·0041). This difference between the proportional reductions in trials of docetaxel plus cyclophosphamide or docetaxel plus capecitabine versus anthracycline, compared with that in trials of single-agent paclitaxel versus anthracycline regimen, was highly significant (p=0·0005).

A meta-analysis of trials directly comparing docetaxel with paclitaxel was performed with data provided from four of six trials (7257 [97·2%] of 7467 randomly assigned patients). Forest plots with trials divided by frequency of docetaxel and paclitaxel administration are shown in the appendix (pp 27, 38–40). Administration once every 2 weeks or 3 weeks was dominated by two trials (ECOG EST1199^26^ and N-SAS-BC 02, Japan^19^), which both administered four cycles of doxorubicin plus cyclophosphamide to each group followed by docetaxel 75–100 mg/m^2^ or paclitaxel 175 mg/m^2^ administered once every 3 weeks for four cycles. N-SAS-BC 02 included two additional groups that compared single-agent docetaxel versus paclitaxel administered once every 3 weeks for eight cycles. There was a significant reduction in recurrence (RR 0·74, 95% CI 0·66–0·84; p<0·0001) and breast cancer mortality (0·80, 0·69–0·93; p=0·0037) favouring docetaxel. However, there was no significant difference between 9 cycles or 12 cycles of weekly docetaxel 35 mg/m^2^ versus paclitaxel 80 mg/m^2^ (1·13, 0·98–1·31, p=0·085). The difference in proportional reductions in trials with schedules of once every 2 weeks or 3 weeks compared with once-per-week schedules was highly significant (p<0·0001). Overall, irrespective of schedule, there were significantly fewer recurrences with docetaxel than paclitaxel (0·89, 0·81–0·97; p=0·011).

In a further meta-analysis of taxane dose fractionation, we identified ten eligible trials. Data obtained from eight of the ten trials (9516 [97·2%] of 9787 women) comparing more versus less frequent scheduling of about the same taxane dose (appendix pp 28, 41–43, 59–60) showed no overall difference in recurrence rates (RR 0·97, 95% CI 0·89–1·05; p=0·42). However, there were significantly fewer recurrences (0·86, 0·78–0·96; p=0·0064), but not breast cancer deaths (0·90, 0·79–1·02; p=0·10), with paclitaxel administered once a week compared with less frequent paclitaxel treatment. The greatest difference was seen in the ECOG EST1199^26^ comparison of paclitaxel 80 mg/m^2^ administered once a week versus the less dose-intense 175 mg/m^2^ administered once every 3 weeks. In the SWOG S0221 trial^27^ little difference was seen when the same paclitaxel 80 mg/m^2^ once a week was compared with paclitaxel 175 mg/m^2^ administered once every 2 weeks; the groups had similar dose intensities. Trials of docetaxel fractionation mostly administered treatment at about the same dose intensity, (ie, 35 mg/m^2^ once a week versus 100 mg/m^2^ once every 3 weeks), but found more recurrences with weekly dosing (1·22, 1·05–1·42; p=0·011). Toxicity, in particular neutropenia, was higher with 3-weekly than with once-weekly or twice-weekly docetaxel, or with paclitaxel (appendix pp 58–60).

Our analysis of trials comparing the order of administration of anthracycline and taxane chemotherapy identified 16 trials that compared the sequence of administration: anthracycline then taxane versus the same drug regimens in the opposite order. Data from ten of these trials (2046 [78·8%] of 2598 patients) are shown in the appendix (pp 29, 44–46). No significant difference in recurrence was seen between the anthracycline then taxane and taxane then anthracycline sequences (RR 1·09, 95% CI 0·91–1·30; p=0·34).

For our analysis of duration of anthracycline chemotherapy, data were available from seven of eight trials comparing longer versus shorter duration of anthracycline-based chemotherapy and one of three trials of longer versus shorter anthracycline plus taxane regimens (8239 [89·5%] of 9203 patients; appendix pp 30, 47–49, 53). Most trials compared six versus four cycles, with GeparTrio^28^ comparing eight versus six cycles of docetaxel plus doxorubicin plus cyclophosphamide administered once every 3 weeks. Across all trials there was a 13% average reduction in recurrence favouring longer duration treatment (RR 0·87, 95% CI 0·78–0·96; p=0·0083).

A further meta-analysis assessed available data from 11 of 16 trials comparing higher versus lower doses of anthracycline chemotherapy (7988 [95·8%] of 8336 patients; appendix pp 31, 50–52, 54). The average reduction in recurrence across all trials was 14% (RR 0·86, 95% CI 0·80–0·92; p<0·0001). However, a significantly (p=0·023) greater benefit was seen in trials in which the difference in cumulative anthracycline dose exceeded 100 mg of doxorubicin (or 150 mg epirubicin) than in trials with smaller differences in cumulative doses (0·77, 0·63–0·93 *vs* 0·99, 0·89–1·11). The benefits were similarly large in trials that increased the cumulative doses of other drugs as well as anthracycline (0·74, 0·66–0·84; p<0·0001).

## Discussion

A combination of anthracycline and taxane has, for more than a decade, been considered optimal chemotherapy for women with early-stage breast cancer who are deemed to be at high enough risk, and fit enough, to benefit from such treatment. However, concerns about the short-term and long-term toxicity of anthracyclines, and consequent desires to de-escalate or optimise treatment, have increased the use of non-anthracycline chemotherapy. This treatment often comprises four or six cycles of docetaxel plus cyclophosphamide, an approach endorsed in editorials and current clinical guidelines (European Society for Medical Oncology, St Gallen International Consensus Guidelines).^29–31^

This meta-analysis of patient-level data from trials comparing taxane-based chemotherapy with and without anthracycline shows that, across all trials, recurrence rates were 14% lower on average with anthracycline. Recurrence reductions were seen in years 0–4 and 5–9, leading to an absolute improvement of 2·6% in 10-year recurrence risk, and 1·6% in 10-year breast cancer mortality. Although meta-analyses should include all relevant randomised trials, variations in trial design and eligibility criteria can complicate analyses. Some trials added anthracycline concurrently with docetaxel plus cyclophosphamide, others compared sequential anthracycline and taxane administered every 3 weeks versus six cycles of docetaxel plus cyclophosphamide, or similar regimens, and some compared taxane with anthracycline versus taxane with carboplatin. By far the largest improvement of 8·7% (95% CI 4·5–12·9) in the 10-year absolute risk of recurrence was seen in the three concurrent administration trials, in which the only difference between the treatment groups was the addition of doxorubicin in each cycle of docetaxel plus cyclophosphamide. Although CIs do not exclude a benefit half this size, even at the lower confidence limit of 4·5%, the benefit is larger than the upper limit for benefit in the sequential administration trials. The substitution of anthracycline cycles for three of the docetaxel plus cyclophosphamide cycles, which resulted in cumulative doses of anthracycline and taxane that were a third lower in the sequential administration than in the concurrent administration trials, probably explains the reduced benefit. Other explanations, such as the use of epirubicin in place of doxorubicin, seem less plausible.^3,17^

The larger benefit observed with concurrent than with sequential anthracycline plus taxane appears discordant with a previous EBCTCG meta-analysis, which showed a greater proportional reduction in recurrence with sequential than concurrent treatment.^4^ Sequential administration allows drugs to be safely administered at a higher dose per cycle than when administered concurrently,^32^ but benefits also depend on the number of cycles and cumulative dose administered. For example, in the NSABP B-30 study,^33^ four cycles of anthracycline then four cycles of taxane were superior to four cycles of doxorubicin plus docetaxel plus cyclophosphamide, a lower cumulative dose regimen. However, in the BCIRG-005 trial,^34^ the same sequential anthracycline plus taxane regimen was no more efficacious than six cycles of doxorubicin plus docetaxel plus cyclophosphamide, a higher cumulative dose comparator. This finding suggests that the cumulative dose is at least as important as the dose intensity.

Sequential taxane plus anthracycline can also be delivered once every 2 weeks, which has higher efficacy than administering the same chemotherapy once every 3 weeks,^4^ as did most trials in this meta-analysis. Such dose-dense regimens, with adequate cumulative doses, should be of similar efficacy to concurrent dosing once every 3 weeks. In the NSABP B-38 study, sequential anthracycline plus paclitaxel administered once every 2 weeks for eight cycles showed similar efficacy to the marginally higher cumulative dose of doxorubicin plus docetaxel plus cyclophosphamide administered once every 3 weeks for six cycles.^35^

The importance of cumulative dose was also apparent in the updated meta-analysis of trials comparing anthracycline regimens with and without taxane. The largest proportional reductions in recurrence and breast cancer mortality were achieved when taxane cycles were added after anthracycline cycles, and the cumulative dose of anthracycline was the same in both groups. With longer follow-up, the updated results now show that reductions in breast cancer mortality persist in years 5–9, so the 10-year absolute benefit is larger than the previously reported 5-year benefit. The benefits seen with longer versus shorter treatment, and higher anthracycline dose per cycle, provide further evidence of the importance of the cumulative dose. No trials were identified that compared six versus four cycles of docetaxel plus cyclophosphamide, but these results indicate that the widely used four-cycle regimen might be less efficacious than six cycles of docetaxel plus cyclophosphamide used as a comparator in many trials in this meta-analysis.

Comparisons between the two different taxane agents were complicated by differences in doses, frequency of administration, and use of concomitant drugs. Across all direct randomised comparisons, fewer recurrences were seen with docetaxel than paclitaxel. Indirect comparisons between trials comparing docetaxel versus anthracycline and paclitaxel versus anthracycline regimens also favoured docetaxel. However, docetaxel was only superior to paclitaxel when administered once every 3 weeks, with fewer recurrences with paclitaxel administered once a week than docetaxel administered once a week. The paclitaxel administered weekly delivers a larger cumulative dose and higher dose intensity than every 3 weeks, which might partly explain why it appears more efficacious. By contrast, in the EST1199 trial, docetaxel administered once a week appeared to be less efficacious than administration every 3 weeks despite similar cumulative doses.^26^ However, four other trials comparing docetaxel administered once a week versus every 3 weeks provided no support, so this might have been a chance finding.

Consistent with many previous EBCTCG meta-analyses, subgroup comparisons showed similar proportional reductions irrespective of recorded patient and tumour characteristics, including age, hormone receptor status, tumour size, tumour grade, histological type, and nodal status. Women with ER-negative cancers are at greater risk of earlier recurrence than those with ER-positive disease and thus gain greater absolute benefits in years 0–4 despite the proportional reductions in recurrence being similar in ER-negative and ER-positive disease. Despite reports suggesting reduced benefits from chemotherapy in postmenopausal rather than premenopausal women with ER-positive cancers,^36,37^ there was no indication of reduced benefit in women aged older than 55 years irrespective of ER status. We found no indication that proportional reductions in recurrence with anthracycline were any different in HER2-positive and HER2-negative disease, as previously suggested.^38^ However, too few trials included HER2-negative and HER2-positive tumours for meaningful subgroup investigation of differential efficacy of anthracycline or taxane according to HER2 amplification, and too few participants had data on Ki-67, *TOP2A*, or gene expression for subgroup analysis.

Benefits from more efficacious chemotherapy regimens need to outweigh any additional short-term and long-term side-effects. Long-term dose-dependent risks of acute myeloid leukaemia and heart failure with anthracyclines are well established.^5–7^ This meta-analysis suggests that treating 1000 women with anthracycline would cause one or two cases of acute myeloid leukaemia, which is fewer than previous reports suggest.^7^ This finding might be because doses of cyclophosphamide, which is also leukaemogenic, were generally the same or lower in the anthracycline group than the comparator group, whereas previous studies often report the incidence of acute myeloid leukaemia following anthracycline plus cyclophosphamide.^7^

Despite individual studies having reported increases in cardiac abnormalities with anthracyclines, no increase was apparent in cardiovascular death, or overall rates of death without recurrence in those who received an anthracycline. However, patients with elevated cardiovascular risk were often excluded, and the median follow-up for trials of taxane with or without anthracycline was only 5·4 years (IQR 4·5–6·9). Longer-term follow-up and more detailed investigation of subclinical changes in cardiac function are needed to fully evaluate cardiovascular risks. The available data on short-term toxicity, most extracted from trial reports, showed no consistent differences in grade 3 or worse toxic effects with and without anthracycline. Notably, despite a lower cumulative taxane dose, neuropathy was not reduced with sequential anthracycline plus taxane compared with six cycles of docetaxel plus cyclophosphamide.^18^ Quality-of-life data were not available from most trials and so this outcome could not be assessed.

Discussions of the potential benefits and risks of different chemotherapy regimens should be informed by the best available evidence, which this study and previous EBCTCG meta-analyses provide. This meta-analysis shows that larger benefits can be achieved by adding anthracycline to a taxane regimen than with a taxane regimen without anthracycline. Regimens with higher cumulative doses of anthracycline and taxane provide the greatest benefits, challenging the current trend in clinical practice and international guidelines towards non-anthracycline chemotherapy, particularly shorter regimens such as four cycles of docetaxel–cyclophosphamide. Absolute benefits for an individual patient, unlike harms,^5–8^ increase with increasing risk of recurrence. Thus, the long-term risk of recurrence, considering any endocrine therapy or anti-HER2 drugs, is a key factor in determining the desirability and type of chemotherapy.

## Supplementary Material

Supplementary Materials

## Data Availability

The policy on data sharing from this study is available online: http://www.ctsu.ox.ac.uk/research/data-access-policies/data-access-and-sharing-policy/view.

## Research in context

### Evidence before this study

Regimens containing anthracycline and taxane are considered to be the most effective adjuvant chemotherapy for early-stage breast cancer, reducing the annual risk of death by at least a third compared with no chemotherapy. However, uncertainty persists about optimal regimens, with concerns that the absolute benefits for some patients might be insufficient to justify the short-term toxicity and longer-term risks of cardiovascular disease and leukaemia. Despite conflicting evidence from individual randomised trials, non-anthracycline-based schedules, such as four or six cycles of docetaxel and cyclophosphamide, are being increasingly used in clinical practice.

### Added value of this study

This collaborative meta-analysis, including individual patient-level data from more than 100 000 women in 86 trials of anthracycline and taxane-based chemotherapy, by including all available evidence, provides more accurate and balanced estimates of the comparative benefits and risks of different chemotherapy regimens than do the individual trials. The large number of available trials makes this the first study to reliably show that anthracycline and taxane significantly reduces breast cancer recurrence and mortality compared with taxane regimens without anthracycline. This meta-analysis also shows the importance of increased cumulative doses and that the proportional reductions in recurrence were not significantly different in women older than 55 years, or those with hormone receptor-positive, or node-negative tumours, for whom there are more concerns about the added value of chemotherapy.

### Implications of all the available evidence

This study confirms that for women with early-stage breast cancer, at sufficient risk of recurrence and fit enough to be offered chemotherapy, combination anthracycline and taxane chemotherapy provides larger benefit than either drug alone. The finding that regimens with higher cumulative doses of anthracycline and taxane provide the greatest benefits, which challenges the current trend in clinical practice towards non-anthracycline chemotherapy, particularly shorter regimens, such as four cycles of docetaxel–cyclophosphamide. This and previous meta-analyses by the Early Breast Cancer Trialists’ Collaborative Group provide a reliable evidence base to inform individual treatment decisions, clinical guidelines, and the design of future clinical trials.

## Early Breast Cancer Trialists’ Collaborative Group (EBCTCG)

Writing committee: Jeremy Braybrooke, Rosie Bradley, Richard Gray, Robert K Hills, Hongchao Pan, Richard Peto, David Dodwell, Paul McGale, Carolyn Taylor (Clinical Trial Service Unit, Nuffield Department of Population Health, University of Oxford, Oxford, UK); Tomohiko Aihara (Aihara Hospital, Osaka, Japan); Stewart Anderson, Greg Yothers (Department of Biostatistics, University of Pittsburgh Graduate School of Public Health, Pittsburgh, PA, USA); Joanne Blum, Joyce O’Shaughnessy (Baylor Charles A Sammons Cancer Center, Dallas, TX, USA); Fatima Cardoso (Champalimaud Clinical Centre, Lisbon, Portugal); Xiaosong Chen (Shanghai Jiaotong University School of Medicine, Shanghai, China); John Crown (St Vincent’s University Hospital, Dublin, Ireland); Bent Ejlertsen, Maj-Britt Jensen (Danish Breast Cancer Group, Rigshospitalet, Copenhagen, Denmark); Thomas W P Friedl, Wolfgang Janni (Universitätsklinikum Ulm, Ulm, Germany); N Harbeck (Brustzentrum der Universität München, Munich, Germany); Eleftherios Mamounas (UF Health Cancer Center Orlando, Orlando, FL, USA); K Narui (Yokohama City University Medical Center, Yokohama, Japan); Ulrike Nitz (Johanniter—Ev Krankenhaus Bethesda Mönchengladbach, Mönchengladbach, Germany); Larry Norton (Memorial Sloan Kettering Cancer Centre, New York City, NY, USA); Martine Piccart (Institut Jules Bordet, Brussels, Belgium); Nicholas Robert (Ontada, Madison, CT, USA); Zhi-Ming Shao, Ke-Da Yu (Fudan University Shanghai Cancer Center, Shanghai, China); Dennis Slamon (David Geffen School of Medicine at UCLA, Los Angeles, CA, USA); Joseph Sparano (Tisch Cancer Institute, Icahn School of Medicine at Mount Sinai, New York, NY, USA); Toru Watanabe (Hamamatsu Oncology Center, Hamamatsu, Japan); Jonas Bergh (joint senior author; Karolinska Institutet and Karolinska Comprehensive Cancer Centre and University Hospital, Stockholm, Sweden); Sandra Swain (joint senior author; Georgetown University Medical Center, Washington, DC, USA).

### EBCTCG secretariat

R Berry, C Boddington, R Bradley, J Braybrooke, M Clarke, C Davies, L Davies, D Dodwell, F Duane, V Evans, J Gay, L Gettins, J Godwin, R Gray, R K Hills, S James, H Liu, Z Liu, E MacKinnon, G Mannu, P McGale, T McHugh, P Morris, M Nakahara, H Pan, R Peto, S Read, E Straiton, C Taylor.

### Groups (lead investigators) contributing data

American College of Surgeons Oncology Group (ACOSOG) USA (A U Buzdar, V J Suman, K K Hunt); Anglo-Celtic Cooperative Oncology Group, UK (J Crown, C F Leonard, J Mansi); Association Européenne de Recherche en Oncologie (AERO), France (C Delbaldo, P Piedbois, E Quinaux); Austrian Breast Cancer Study Group (ABCSG), Vienna, Austria (C Fesl, M Gnant, L Sölkner, G G Steger); Bergen Breast Cancer Group, Norway (H P Eikesdal, P E Lønning); Breast Cancer International Research Group (BCIRG)/Translational Research on Oncology (TRIO), USA, (V Bee, J Crown, H Fung, J Mackey, M Martin, M Press, D Slamon); Breast International Group (BIG), Brussels, Belgium, (D Cameron, J Crown, E de Azambuja, R Gelber, M J Piccart, M Regan); Bordet Institute, Brussels, Belgium (A Di Leo, V van Dooren, J M Nogaret, M J Piccart); Canadian Cancer Trials Group (MA.21), Kingston, ON, Canada (J M S Bartlett, B E Chen, K Gelmon, P E Goss, M N Levine, W Parulekar, K I Pritchard, L E Shepherd); Cancer and Leukemia Group B (CALGB), Washington DC, USA (D Berry, C Cirrincione, L Norton, L N Shulman, E Winer); Dana-Farber Cancer Institute, Boston, MA, USA (R S Gelman, J R Harris, C Henderson, C L Shapiro, E Winer); Danish Breast Cancer Cooperative Group (DBCG), Copenhagen, Denmark (B Ejlertsen, M-B Jensen, A Knoop, H T Mouridsen, B V Offersen, T F Tvedskov); Dutch Breast Cancer Trialists’ Group (BOOG), Netherlands, (E van Leeuwen-Stok, S Linn, A G J van Rossum, H van Tinteren, E van Werkhoven); Eastern Cooperative Oncology Group (ECOG), Boston, MA, USA (N E Davidson, L Goldstein, R J Gray, J A Sparano); European Cooperative Trial in Operable Breast Cancer (ECTO), Italy, (W Eiermann, L Gianni, P Valagussa); European Organization for Research and Treatment of Cancer (EORTC), Brussels, Belgium/Breast International Group (EORTC10994/BIG 0–01; EORTC10041/MINDACT; J Bogaerts, H Bonnefoi, M Piccart, C Poncet); Finnish Breast Cancer Group, Finland (R Huovinen, H Joensuu); French Adjuvant Study Group (GFEA), Guyancourt, France (J Bonneterre, P Fargeot, P Fumoleau, P Kerbrat, E Luporsi, M Namer); Fudan University Shanghai Cancer Center, Shanghai, China (Z-M Shao, K-D Yu); GEICAM, Spanish Breast Cancer Group, Spain (E Carrasco, M Martin, M A Segui); German Breast Group (GBG 42/NNBC 3), Germany (C Meisner, S Loibl, V Nekljudova, C Thomssen); German Breast Group (GeparTrio), Germany (G von Minckwitz, S Kümmel, S Loibl); Gruppo Oncologico Dell’Italia Meridionale (GOIM), Rome, Italy (M Lopez, P Vici); Hellenic Cooperative Oncology Group, Athens, Greece (G Fountzilas, G-A Koliou); Hellenic Oncology Research Group, Greece (D Mavroudis, E Saloustros); Institut Curie—Hôpital René Huguenin (RAPP-01), Paris, St Cloud, France (E Brain); Institut Gustave-Roussy (FNCLCC), Villejuif, France (S Delaloge, S Michiels, P Pélissier); Instituto Nacional de Cancer, Brazil (J Bines, R M B Sarmento); Istituto Nazionale per lo Studio e la Cura dei Tumori, Milan, Italy (G Bonadonna, C Brambilla, A Rossi, P Valagussa); International Collaborative Cancer Group, London, UK (J M Bliss, R C Coombes, L Kilburn, M Marty); Istituto Scientifico Romagnolo per lo Studio e la Cura dei Tumori (IBIS 02), Meldola, Italy (D Amadori, F Boccardo, O Nanni, A Rubagotti, E Scarpi); Japan Breast Cancer Research Group (JBCRG), Japan (N Masuda, M Toi, T Ueno); Kanagawa Breast Oncology Group, Japan (T Ishikawa, K Narui); Kobe Breast Cancer Oncology Group, Japan (K Matsumoto, S Takao); Ludwig-Maximilians University (ADEBAR trial), Munich, Germany (W Janni, H Sommer); Metaxas Memorial Cancer Hospital, Athens, Greece (P Foroglou, G Giokas, D Kondylis, B Lissaios); Multicentre Group, Germany (S Kümmel, V Nekljudova, M Reinisch); National Cancer Center, Goyang, South Korea (K S Lee, B-H Nam, J Ro); National Cancer Institute (BREAST-10), Naples, Italy (A de Matteis, F Perrone); National Surgical Adjuvant Breast and Bowel Project (NSABP), Pittsburgh, PA, USA (S Anderson, E P Mamounas, G Tang, N Wolmark); National Surgical Adjuvant Study Group (N-SAS-BC), Japan, (T Aihara, Y Hozumi, Y Nomura); Neo-tAnGo Trial Group, UK, (H Earl, L Hiller, A-L Vallier); North-West Oncology Group (GONO), Italy (L Del Mastro, M Venturini); PACS Study Group, France (S Delaloge, T Delozier, J Lemonnier, A L Martin, H Roché, M Spielmann); Shanghai Jiao Tong University School of Medicine, China (X Chen, K Shen); Southwest Oncology Group, San Antonio, TX, USA (K Albain, W Barlow, G T Budd, J Gralow, D Hayes); SUCCESS Study Group, Germany (T WP Friedl, W Janni, H Sommer); NCRI TACT Trial Group, London, UK (P Barrett-Lee, J M Bliss, P Ellis, L Kilburn); University Federico II (TAXit216), Naples, Italy (A R Bianco, M De Laurentis, S De Placido); University Hospitals, Leuven and St-Augustinus Hospital, Wilrijk, Belgium (H Wildiers); University of Texas MD Anderson Cancer Center, Houston, TX, USA (A U Buzdar, L Hsu); University of Hull and Lincoln County Hospital, UK (O Eremin, L G Walker); Uppsala-Örebro Cancer Study Group (SBG), Sweden (J Ahlgren, J Bergh, C Blomqvist, L Holmberg, H Lindman); US Oncology, Houston, TX, USA (L Asmar, S E Jones, J O’Shaughnessy); West German Study Group (WSG Plan B), Germany (O Gluz, N Harbeck, C Liedtke, U Nitz).

### EBCTCG steering committee

Jonas Bergh, Sandra Swain (Co-Chairs), David Cameron (Vice-Chair), Kathy Albain, Stewart Anderson, Rodrigo Arriagada, John Bartlett, Elizabeth Bergsten-Nordström, Judith Bliss, Rosie Bradley*, Etienne Brain, Jeremy Braybrooke*, Lisa Carey, Mike Clarke*, Robert Coleman, Jack Cuzick, Nancy Davidson, Lucia Del Mastro, Angelo Di Leo, James Dignam, David Dodwell*, Mitch Dowsett, Fran Duane*, Bent Ejlertsen, Prudence A Francis, Richard Gelber, Michael Gnant, Matthew P Goetz, Pam Goodwin, Richard Gray*, Pat Halpin-Murphy, Dan Hayes, Catherine Hill, Robert K Hills*, Reshma Jagsi, Wolfgang Janni, Zulian Liu*, Sibylle Loibl, Elizabeth MacKinnon*, Eleftherios Mamounas, Gurdeep Mannu*, Miguel Martín, Paul McGale*, Hirofumi Mukai, Valentina Nekljudova, Larry Norton, Yasuo Ohashi, Hongchao Pan*, Richard Peto*, Martine Piccart, Lori Pierce, Philip Poortmans, Kathleen I Pritchard, Vinod Raina, Daniel Rea, Meredith Regan, John Robertson, Emiel Rutgers, Roberto Salgado, Dennis Slamon, Tanja Spanic, Joseph Sparano, Guenther Steger, Carolyn Taylor*, Gong Tang, Masakazu Toi, Andrew Tutt, Giuseppe Viale, Xiang Wang, Tim Whelan, Nicholas Wilcken, Norman Wolmark, Ke-Da Yu. *EBCTCG Secretariat, Clinical Trial Service Unit, Nuffield Department of Population Health.

## References

[1] Early Breast Cancer Trialists’ Collaborative Group. Polychemotherapy for early breast cancer: an overview of the randomised trials. Lancet 1998; 352: 930–42. 9752815

[2] Early Breast Cancer Trialists’ Collaborative Group (EBCTCG). Effects of chemotherapy and hormonal therapy for early breast cancer on recurrence and 15-year survival: an overview of the randomised trials. Lancet 2005; 365: 1687–717. 15894097 10.1016/S0140-6736(05)66544-0

[3] Peto R, Davies C, Godwin J, Comparisons between different polychemotherapy regimens for early breast cancer: meta-analyses of long-term outcome among 100 000 women in 123 randomised trials. Lancet 2012; 379: 432–44. 22152853 10.1016/S0140-6736(11)61625-5 PMC3273723

[4] Gray R, Bradley R, Braybrooke J, Increasing the dose intensity of chemotherapy by more frequent administration or sequential scheduling: a patient-level meta-analysis of 37 298 women with early breast cancer in 26 randomised trials. Lancet 2019; 393: 1440–52. 30739743 10.1016/S0140-6736(18)33137-4 PMC6451189

[5] Greenlee H, Iribarren C, Rana JS, Risks of cardiovascular disease in women with and without breast cancer: the Pathways Heart Study. J Clin Oncol 2022; 40: 1647–58. 35385342 10.1200/JCO.21.01736 PMC9113215

[6] Praga C, Bergh J, Bliss J, Risk of acute myeloid leukemia and myelodysplastic syndrome in trials of adjuvant epirubicin for early breast cancer: correlation with doses of epirubicin and cyclophosphamide. J Clin Oncol 2005; 23: 4179–91. 15961765 10.1200/JCO.2005.05.029

[7] Freedman RA, Seisler DK, Foster JC, Risk of acute myeloid leukemia and myelodysplastic syndrome among older women receiving anthracycline-based adjuvant chemotherapy for breast cancer on Modern Cooperative Group Trials (Alliance A151511). Breast Cancer Res Treat 2017; 161: 363–73. 27866278 10.1007/s10549-016-4051-1 PMC5226883

[8] Crown J, O’Leary M. The taxanes: an update. Lancet 2000; 355: 1176–78. 10791395 10.1016/S0140-6736(00)02074-2

[9] Early Breast Cancer Trialists’ Collaborative Group. Treatment of early breast cancer: worldwide evidence, 1985–1990. Oxford: Oxford University Press, 1990.

[10] Early Breast Cancer Trialists’ Collaborative Group (EBCTCG). Aromatase inhibitors versus tamoxifen in early breast cancer: patient-level meta-analysis of the randomised trials. Lancet 2015; 386: 1341–52. 26211827 10.1016/S0140-6736(15)61074-1

[11] Stewart LA, Clarke M, Rovers M, Preferred Reporting Items for Systematic Review and Meta-Analyses of Individual Participant Data: the PRISMA-IPD Statement. JAMA 2015; 313: 1657–65. 25919529 10.1001/jama.2015.3656

[12] Slamon D, Eiermann W, Robert N, Adjuvant trastuzumab in HER2-positive breast cancer. N Engl J Med 2011; 365: 1273–83. 21991949 10.1056/NEJMoa0910383 PMC3268553

[13] Ueno T, Masuda N, Sato N, Multicenter study of primary systemic therapy with docetaxel, cyclophosphamide and trastuzumab for HER2-positive operable breast cancer: the JBCRG-10 study. Jpn J Clin Oncol 2020; 50: 3–11. 31821506 10.1093/jjco/hyz119 PMC6978625

[14] Ejlertsen B, Tuxen MK, Jakobsen EH, Adjuvant cyclophosphamide and docetaxel with or without epirubicin for early TOP2A-normal breast cancer: DBCG 07-READ, an open-label, phase III, randomized trial. J Clin Oncol 2017; 35: 2639–46. 28661759 10.1200/JCO.2017.72.3494

[15] Cardoso F, van’t Veer LJ, Bogaerts J, 70-gene signature as an aid to treatment decisions in early-stage breast cancer. N Engl J Med 2016; 375: 717–29. 27557300 10.1056/NEJMoa1602253

[16] Chen X, Ye G, Zhang C, Li X, Shen K. Non-anthracycline-containing docetaxel and cyclophosphamide regimen is associated with sustained worse outcome compared with docetaxel, anthracycline and cyclophosphamide in neoadjuvant treatment of triple negative and HER2-positive breast cancer patients: updated follow-up data from NATT study. Chin J Cancer Res 2016; 28: 561–69. 28174484 10.21147/j.issn.1000-9604.2016.06.02 PMC5242451

[17] Perez DJ, Harvey VJ, Robinson BA, A randomized comparison of single-agent doxorubicin and epirubicin as first-line cytotoxic therapy in advanced breast cancer. J Clin Oncol 1991; 9: 2148–52. 1960557 10.1200/JCO.1991.9.12.2148

[18] Blum JL, Flynn PJ, Yothers G, Anthracyclines in early breast cancer: the ABC Trials—USOR 06–090, NSABP B-46-I/USOR 07132, and NSABP B-49 (NRG Oncology). J Clin Oncol 2017; 35: 2647–55. 28398846 10.1200/JCO.2016.71.4147 PMC5549453

[19] Watanabe T, Kuranami M, Inoue K, Comparison of an AC-taxane versus AC-free regimen and paclitaxel versus docetaxel in patients with lymph node-positive breast cancer: final results of the National Surgical Adjuvant Study of Breast Cancer 02 trial, a randomized comparative phase 3 study. Cancer 2017; 123: 759–68. 28081304 10.1002/cncr.30421 PMC6668007

[20] Yu KD, Ye FG, He M, Effect of adjuvant paclitaxel and carboplatin on survival in women with triple-negative breast cancer. A phase 3 randomized trial. JAMA Oncol 2020; 6: 1390–96. 32789480 10.1001/jamaoncol.2020.2965 PMC7426881

[21] Narui K, Ishikawa T, Shimizu D, Anthracycline could be essential for triple-negative breast cancer: a randomised phase II study by the Kanagawa Breast Oncology Group (KBOG) 1101. Breast 2019; 47: 1–9. 31229857 10.1016/j.breast.2019.06.003

[22] Nitz U, Gluz O, Clemens M, West German Study PlanB trial: adjuvant four cycles of epirubicin and cyclophosphamide plus docetaxel versus six cycles of docetaxel and cyclophosphamide in HER2-negative early breast cancer. J Clin Oncol 2019; 37: 799–808. 30785826 10.1200/JCO.18.00028

[23] Janni W, Nitz U, Rack BK, Pooled analysis of two randomized phase III trials (PlanB/SuccessC) comparing six cycles of docetaxel and cyclophosphamide to sequential anthracycline taxane chemotherapy in patients with intermediate and high risk HER2-negative early breast cancer (n=5,923). J Clin Oncol 2018; 36: 522.

[24] Yu K-D, Liu X-Y, Chen L, Anthracycline-free or short-term regimen as adjuvant chemotherapy for operable breast cancer: a phase III randomised non-inferiority trial. Lancet Reg Health West Pac 2021; 11: 100158. 34327363 10.1016/j.lanwpc.2021.100158 PMC8315472

[25] Piccart M, van ‘t Veer LJ, Poncet C, 70-gene signature as an aid for treatment decisions in early breast cancer: updated results of the phase 3 randomised MINDACT trial with an exploratory analysis by age. Lancet Oncol 2021; 22: 476–88. 33721561 10.1016/S1470-2045(21)00007-3

[26] Sparano JA, Wang M, Martino S, Weekly paclitaxel in the adjuvant treatment of breast cancer. N Engl J Med 2008; 358: 1663–71. 18420499 10.1056/NEJMoa0707056 PMC2743943

[27] Budd GT, Barlow WE, Moore HC, SWOG S0221: a phase III trial comparing chemotherapy schedules in high-risk early-stage breast cancer. J Clin Oncol 2015; 3: 58–64 10.1200/JCO.2014.56.3296 PMC4268253 25422488

[28] von Minckwitz G, Blohmer JU, Costa SD, Response-guided neoadjuvant chemotherapy for breast cancer. J Clin Oncol 2013; 31: 3623–30. 24002511 10.1200/JCO.2012.45.0940

[29] Hurvitz SA, McAndrew NP, Bardia A, A careful reassessment of anthracycline use in curable breast cancer. NPJ Breast Cancer 2021; 7: 134. 34625570 10.1038/s41523-021-00342-5 PMC8501074

[30] Cardoso F, Kyriakides S, Ohno S, Early breast cancer: ESMO Clinical Practice Guidelines for diagnosis, treatment and follow-up. Ann Oncol 2019; 30: 1194–220. 31161190 10.1093/annonc/mdz173

[31] Burstein HJ, Curigliano G, Thürlimann B, Customizing local and systemic therapies for women with early breast cancer: the St. Gallen International Consensus Guidelines for treatment of early breast cancer 2021. Ann Oncol 2021; 32: 1216–35. 34242744 10.1016/j.annonc.2021.06.023 PMC9906308

[32] Norton L Evolving concepts in the systemic drug therapy of breast cancer. Semin Oncol 1997; 24 (suppl 10): S10-3–10. 9275000

[33] Swain SM, Jeong J-H, Geyer CE Jr, Longer therapy, iatrogenic amenorrhea, and survival in early breast cancer. N Engl J Med 2010; 362: 2053–65. 20519679 10.1056/NEJMoa0909638 PMC2935316

[34] Mackey JR, Pieńkowski T, Crown J, Long-term outcomes after adjuvant treatment of sequential versus combination docetaxel with doxorubicin and cyclophosphamide in node-positive breast cancer: BCIRG-005 randomized trial. Ann Oncol 2016; 27: 1041–47. 26940688 10.1093/annonc/mdw098

[35] Swain SM, Tang G, Geyer CE Jr, Definitive results of a phase III adjuvant trial comparing three chemotherapy regimens in women with operable, node-positive breast cancer: the NSABP B-38 trial. J Clin Oncol 2013; 31: 3197–204. 23940225 10.1200/JCO.2012.48.1275 PMC3757290

[36] Sparano JA, Gray RJ, Makower DF, Adjuvant chemotherapy guided by a 21-gene expression assay in breast cancer. N Engl J Med 2018; 379: 111–21. 29860917 10.1056/NEJMoa1804710 PMC6172658

[37] Kalinsky K, Barlow WE, Gralow JR, 21-gene assay to inform chemotherapy benefit in node-positive breast cancer. N Engl J Med 2021; 385: 2336–47. 34914339 10.1056/NEJMoa2108873 PMC9096864

[38] Di Leo A, Desmedt C, Bartlett JM, HER2 and TOP2A as predictive markers for anthracycline-containing chemotherapy regimens as adjuvant treatment of breast cancer: a meta-analysis of individual patient data. Lancet Oncol 2011; 12: 1134–42. 21917518 10.1016/S1470-2045(11)70231-5

