# Supplementary material for: Anthracycline-containing and taxane-containing chemotherapy for early-stage operable breast cancer: a patient-level meta-analysis of 100 000 women from 86 randomised trials
Source: Lancet. Author manuscript; Available in PMC 2024 Apr 17. (PMC11023015; doi:10.1016/S0140-6736(23)00285-4)
Supplement: Supplementary Materials [file NIHMS1964839-supplement-Supplementary_Materials.pdf]

THE LANCET

Supplementary appendix

This appendix formed part of the original submission and has been peer reviewed. We post it as supplied by the authors.

Supplement to: Early Breast Cancer Trialists' Collaborative Group (EBCTCG). Anthracycline-containing and taxane-containing chemotherapy for early-stage operable breast cancer: a patient-level meta-analysis of 100 000 women from 86 randomised trials. *Lancet* 2023; **401:** 1277–92.

Webappendix: Supplementary figures and tables for "Anthracycline and taxane containing chemotherapy for early-stage operable breast cancer: meta-analyses of 100,000 women in 86 randomised trials"

CONTENTS LIST (Click on any listed item with a page number to jump to it.)

Page 3: CONSORT of trials included

Page 4-5: Table of eligibility and baseline characteristics by trial

Page 6: Kaplan-Meier graphs, for 4 different endpoints (any recurrence, distant recurrence at any time, local recurrence as first event, contralateral recurrence as first event)

Pages 7-13: Forest plots, one line per-trial for 7 different endpoints (distant recurrence at any time, local recurrence as first event, contralateral recurrence as first event, breast cancer mortality, death without recurrence in year 0, death without recurrence, all-cause mortality)

Page 14-15: Subgroup analyses for distant recurrence and breast cancer mortality for group A

Page 16-18: Subgroup analyses for recurrence, distant recurrence and breast cancer mortality for all trials

Pages 19-20: 5 year risk of recurrence within subgroups for tumour size and tumour grade

Page 21: Mortality by cause and incidence of second cancers

Page 22: AML incidence, forest plot

Page 23: Death without recurrence by tumour size and nodal status

Page 24: Forest plot, one line per-trial for any recurrence: Taxane-plus-anthracycline versus anthracycline

Page 25: Kaplan-Meier curves (any recurrence, breast cancer mortality, death without recurrence, all-cause mortality) for taxane-plus-anthracycline versus anthracycline based regimens

Pages 26-31: Forest plots, one line per-trial for any recurrence

- **Page 26:** Taxane versus anthracycline
- **Page 27:** Head-to-head taxane (Docetaxel vs Paclitaxel)
- **Page 28:** Chemotherapy in fractions
- **Page 29:** Sequencing order of anthracycline and taxane chemotherapy
- **Page 30:** Longer versus shorter anthracycline/taxane regimems
- **Page 31:** Higher versus lower dose

Pages 32-52: Forest plots, one line per-trial (breast cancer mortality, death without recurrence and all-cause mortality)

- **Pages 32-34:** Taxane-plus-anthracycline versus anthracycline based regimens
- **Pages 35-37:** Taxane versus anthracycline
- **Pages 38-40:** Head-to-head taxane (Docetaxel vs Paclitaxel)
- **Pages 41-43:** Chemotherapy in fractions
- **Pages 44-46:** Sequencing order of anthracycline and taxane chemotherapy
- **Pages 47-49:** Longer versus shorter anthracycline/taxane regimems
- **Pages 50-52:** Higher versus lower dose

Page 53-54: Kaplan-Meier curves (any recurrence, breast cancer mortality, death without recurrence and all-cause mortality)

- **Page 53:** Longer versus shorter anthracycline/taxane regimens
- **Page 54:** Higher versus lower dose

Pages 55-60: Toxicity (as reported in the trials publications)

- **Pages 55-57:** Taxane plus anthracycline versus taxane without anthracycline
- **Page 58:** Docetaxel versus paclitaxel
- **Page 59-60:** Paclitaxel or docetaxel in smaller fractions versus larger fractions

Pages 61-91: Statistical Analysis Plan including list of relevant trials and publications, and variables requested

Pages 92-94: EBCTCG search strategies

P3: CONSORT of trials included in the meta-analysis of taxane plus anthracycline versus taxane without anthracycline

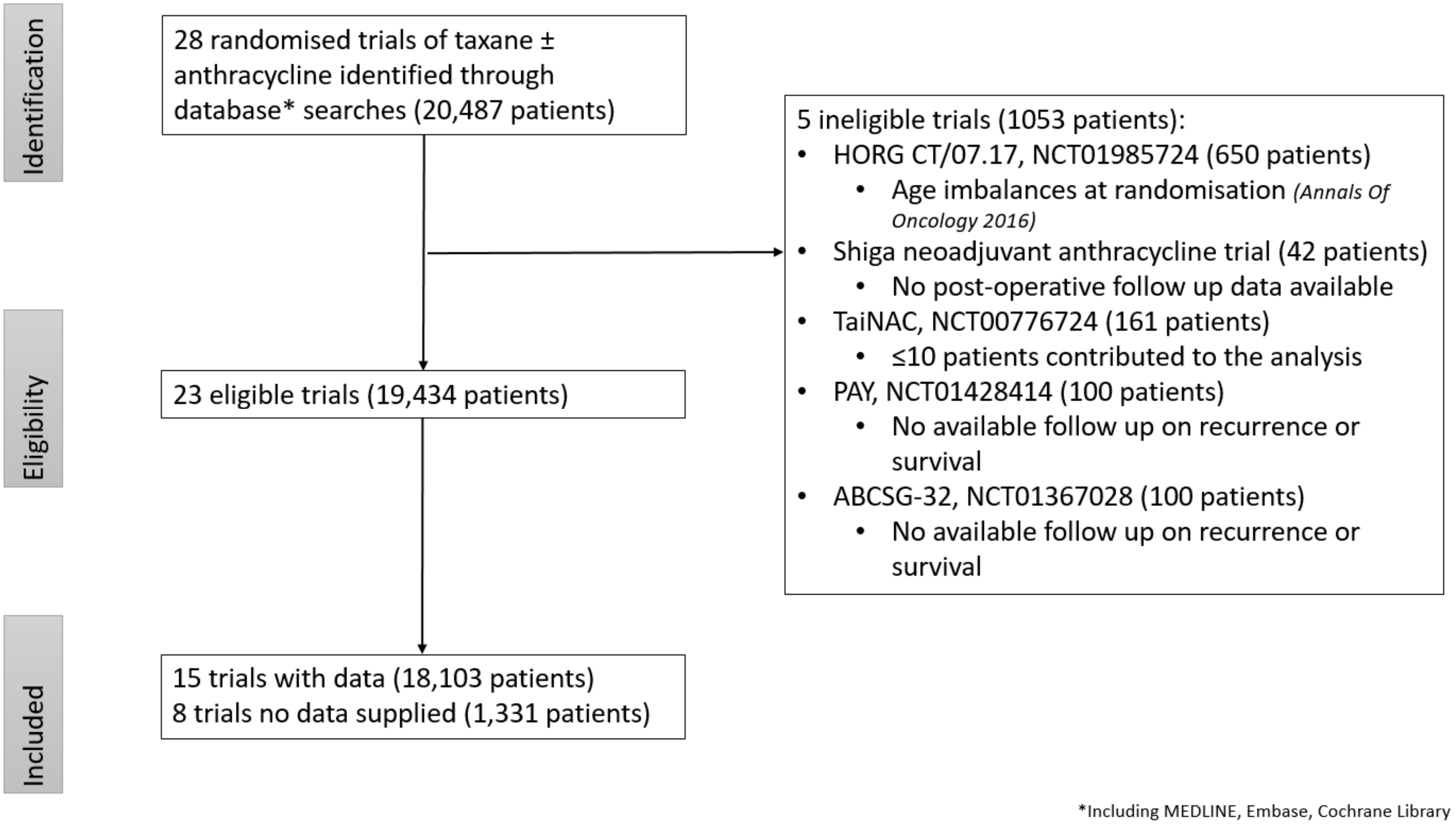

P4-5: Table of eligibility and baseline characteristics by trial

Trial name (years of recruitment)	No. of patients randomised	Key eligibility criteria	Median age in years (range)	Nodal status	Tumour size	Tumour grade	ER status	HER2 status	Median follow up in years
**(a) Concurrent docetaxel plus anthracycline versus same dose docetaxel plus cyclophosphamide**									
USO 06090/11271<br>(2007-2009)	1296	18 to 70yrs<br>N+ or high risk N0<br>ER any<br>HER2-negative	53 (26 - 71)	35% N0<br>51% N1-3<br>11% N4-9<br>3% N10+	38% T1<br>57% T2<br>5% T3/T4	12% Well differentiated<br>38% Moderately<br>45% Poorly<br>5% Unknown	30% ER-negative<br>70% ER-positive	100% HER2-negative	9.4<br>(7.8 – 10.0)
NSABP B-46-I<br>(2009-2012)	1077	18 to 70yrs<br>N+ or high risk N0<br>ER any<br>HER2-negative	53 (26 - 70)	38% N0<br>43% N1-3<br>14% N4-9<br>5% N10+	39% T1<br>54% T2<br>8% T3/T4	10% Well differentiated<br>37% Moderately<br>52% Poorly<br>1% Unknown	33% ER-negative<br>67% ER-positive	100% HER2-negative	6.3<br>(5.1 – 7.1)
Shanghai Jiao Tong<br>(2009-2011)	96	18 to 70yrs<br>TNBC or HER2-positive	48 (25 - 69)	*17% N0<br>*83% N+	100% Unknown	24% Moderately differentiated<br>31% Poorly differentiated<br>45% Unknown	84% ER-negative<br>16% ER-positive	51% HER2-negative<br>49% HER2-positive	4.7<br>(4.1 – 5.3)
**(b) Sequential taxane plus anthracycline versus higher cumulative dose docetaxel plus cyclophosphamide**									
HORG CT/07.17<br>(2007-2013)<br>Ineligible due to age imbalances	*650*	*18 to 75yrs<br>N+<br>HER2-negative*	*24 (56 - 80)*	*63% N1-3<br>28% N4-9<br>9% N10+*	*100% Unknown*	*6% Well-differentiated<br>53% Moderately<br>36% Poorly<br>5% Unknown*	*15% ER-negative<br>84% ER-positive<br>1% ER-unknown*	*100% HER2-negative*	*4.0<br>(2.8 – 4.9)*
DBCG 07 READ<br>(2008-2012)	2012	18 to 75yrs<br>N+ or high risk N0<br>ER any<br>HER2-any<br>TOP2A normal	51 (23 - 74)	45% N0<br>41% N1-3<br>10% N4-9<br>4% N10+	59% T1<br>39% T2<br>2% T3/T4	17% Well differentiated<br>46% Moderately<br>32% Poorly<br>6% Unknown	26% ER-negative<br>74% ER-positive	89% HER2-negative<br>11% HER2-positive	8.4<br>(7.5 – 7.4)
Kanagawa Japan<br>(2009-2011)	103	20 to 80yrs<br>HR negative	55 (26 - 78)	*40% N0<br>*60% N+	*17% T1<br>*64% T2<br>*18% T3/T4	*17% Well differentiated<br>*24% Moderately<br>*58% Poorly<br>*1% Unknown	93% ER-negative<br>7% ER-positive<br>(Allred score 3-6)	72% HER2-negative<br>28% HER2-positive	3.9<br>(3.3 – 5.1)
NSABP B-49<br>(2012-2013)	1870	≥18yrs<br>N+ or high risk N0<br>Any ER<br>HER2-negative	55 (21 - 82)	45% N0<br>40% N1-3<br>11% N4-9<br>4% N10+	43% T1<br>51% T2<br>6% T3/T4	9% Well differentiated<br>36% Moderately<br>55% Poorly	31% ER-negative<br>69% ER-positive	100% HER2-negative	4.4<br>(4.0 – 4.8)
LMU SUCCESS C<br>(2009-2011)	3643	≥18yrs<br>N+ or high risk N0<br>ER any<br>HER2-negative	55 (24 - 79)	42% N0<br>47% N1-3<br>8% N4-9<br>3% N10+	44% T1<br>50% T2<br>6% T3/T4	6% Well differentiated<br>51% Moderately<br>43% Poorly	25% ER-negative<br>75% ER-positive	100% HER2-negative	5.4<br>(5.0 – 5.5)
WSG Plan B<br>(2009-2011)	2449	18 to 75yrs<br>N+ or high risk N0<br>ER any<br>HER2-negative	55 (25 - 77)	59% N0<br>34% N1-3<br>5% N4-9<br>2% N10+	54% T1<br>43% T2<br>3% T3/T4	4% Well differentiated<br>50% Moderately<br>42% Poorly<br>4% Unknown	19% ER-negative<br>73% ER-positive<br>8% Unknown	99% HER2-negative<br>1% HER2-positive	5.0<br>(4.6 – 5.1)

JBCRG-10<br>(2009-2011)	67	20 to 70yrs<br>T1c-3, N0-1, M0<br>ER any<br>HER2-positive	54 (34 - 70)	*63% N0<br>*37% N+	11% T1<br>76% T2<br>13% T3/T4	12% Well differentiated<br>40% Moderately<br>34% Poorly<br>14% Unknown	41% ER-negative<br>59% ER-positive	100% HER2-positive	4.4<br>(4.2 – 4.9)
JBCRG-09<br>(2009-2012)	195	20 to 70yrs<br>T1c-3, N0-1, M0<br>ER-positive<br>HER2-negative	48 (26 - 69)	*49% N0<br>*51% N+	14% T1<br>73% T2<br>13% T3/T4	18% Well differentiated<br>40% Moderately<br>14% Poorly<br>28% Unknown	100% ER-positive	100% HER2-negative	5.9<br>(5.3 – 6.5)
MASTER Fudan<br>(2010-2017)	1047	18 to 75yrs<br>N+ or high risk N0<br>ER any<br>HER2-negative	50 (27 - 70)	41% N0<br>59% N+	44% T1<br>56% T2	66% Moderately<br>34% Poorly	8% ER-negative<br>92% ER-positive	100% HER2-negative	4.7<br>(3.7 – 6.3)
**(c) Taxane plus anthracycline versus taxane ± capecitabine**									
N-SAS-BC 02<br>(2000-2006)	1060	18 to 70yrs<br>N+<br>ER any<br>HER2-any	53 (24 - 70)	100% N1-3	37% T1<br>55% T2<br>7% T3/T4<br>1% Unknown	100% Unknown	43% ER-negative<br>57% ER-positive	100% Unknown	7.5<br>(6.1 – 8.4)
MINDACT<br>(2006-2011)	392	18 to 70yrs<br>N1-3<br>ER any<br>HER2-any	53 (28 - 70)	99% N1-3<br>1% N4-9	54% T1<br>42% T2<br>4% T3/T4	9% Well differentiated<br>61% Moderately<br>30% Poorly	10% ER-negative<br>90% ER-positive	90% HER2-negative<br>10% HER2-positive	4.9<br>(4.3 – 5.5)
TaiNAC<br>(2009-2014)<br>Ineligible as ≤10 randomised	*10*	*Age 20+<br>Size 30mm+<br>HER2-negative*	*46 (30 - 63)*	*100% Unknown*	*20% T1<br>60% T2<br>10% T3<br>10% Unknown*	*10% Well-differentiated<br>20% Moderately<br>20% Poorly<br>50% Unknown*	*20% ER-negative<br>60% ER-positive<br>20% ER-unknown*	*100% HER2-negative*	*2.6<br>(0.7 – 3.0)*
ABCSG-32<br>(2011-2014)<br>Ineligible no follow-up	*100*	*≥18yrs<br>ER any<br>HER2-positive*	*52 (23 – 79)*	**63% N0<br>*37% N+*	**35% T1<br>*53% T2<br>*11% T3/4*	**2% Well differentiated<br>*30% Moderately<br>*63% Poorly<br>*5% Unknown*	*47% ER-negative<br>53% ER-unknown*	*100% HER2-positive*	*0.5<br>(0.4 – 0.5)*
**(d) Taxane plus anthracycline versus taxane plus carboplatin (confounded)**									
BCIRG 006<br>(2001-2004)	2149	18 to 70yrs<br>N+ or high risk N0<br>ER any<br>HER2-positive	49 (22 - 74)	29% N0<br>38% N1-3<br>22% N4-9<br>11% N10+	39% T1<br>54% T2<br>6% T3/T4	1% Well differentiated<br>29% Moderately<br>65% Poorly<br>5% Unknown	50% ER-negative<br>50% ER-positive	100% HER2-positive	10.5<br>(9.0 – 10.6)
TaiNAC<br>(2009-2014)<br>Ineligible as ≤10 randomised	*8*	*Age 20+<br>Size 30mm+<br>HER2-negative*	*52 (38 – 61)*	*100% Unknown*	*25% T1<br>75% T2*	*25% Well-differentiated<br>38% Moderately<br>37% Unknown*	*75% ER-positive<br>25% ER-unknown*	*100%- HER2-negative*	*1.6<br>(1.0 – 3.4)*
PATTERN Fudan<br>(2011-2016)	647	18 to 70yrs<br>N+ or N0 if T>10mm<br>TNBC	51 (23 - 70)	74% N0<br>26% N+	54% T1<br>46% T2/3	27% Moderately<br>73% Poorly	100% ER-negative	100% HER2-negative	5.0<br>(4.0 – 6.9)

*Before neo-adjuvant therapy

HORG CT/07.17, ABCSG-32 and TaiNAC provided data but these trials were ineligible for this meta-analysis. See CONSORT on page 3.

P6: 10-year (*5-year) cumulative risk of (A) any recurrence, (B) distant recurrence at any time, (C)* isolated local recurrence as first event, (D)* contralateral recurrence as first event in trials of taxane plus anthracycline versus taxane without anthracycline

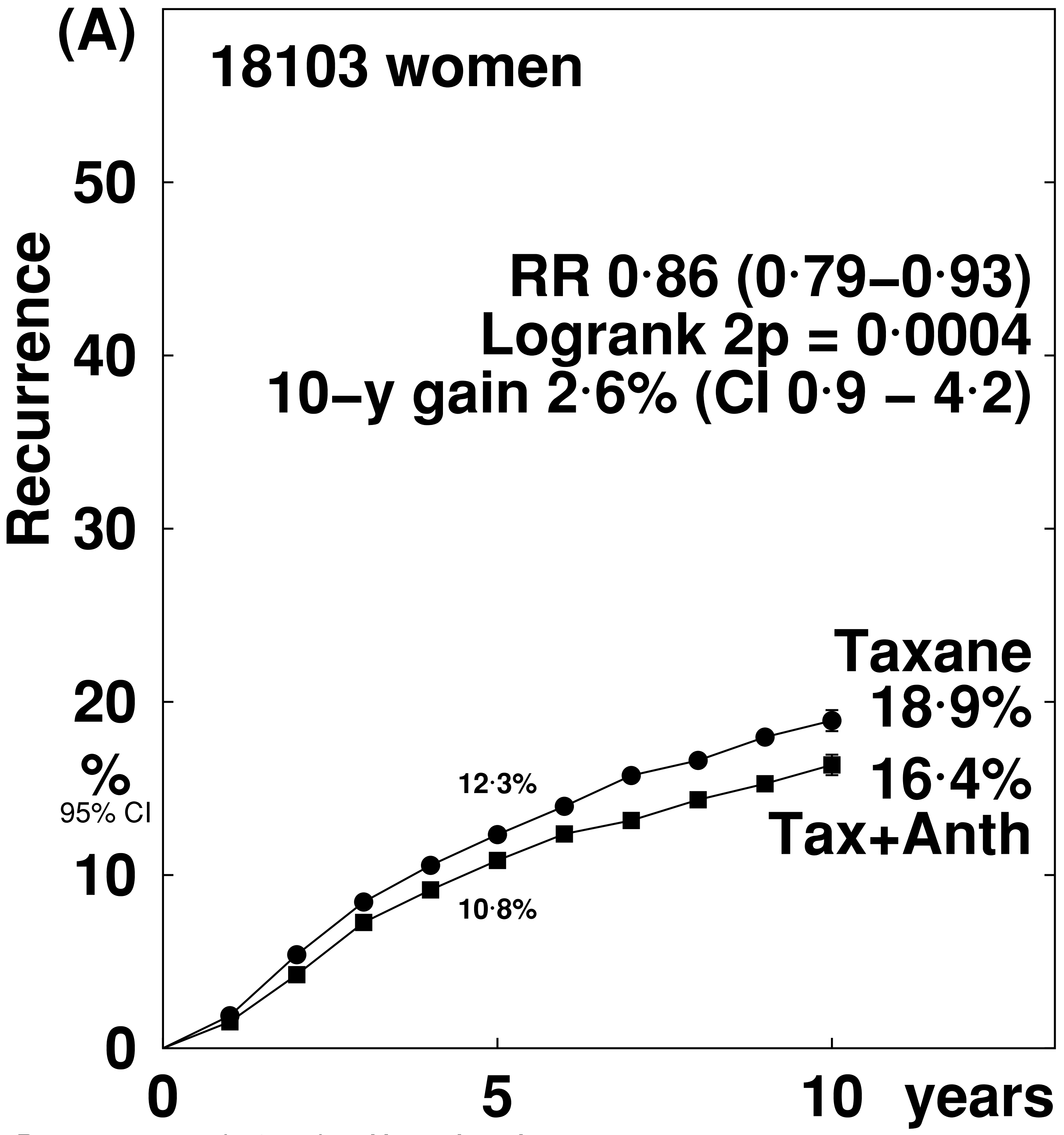

Recurrence rates (% / year) and logrank analyses

Allocation	Years 0 – 4	Years 5 – 9	Year 10+
Tax+Anth	2·34 (900 / 38514)	1·37 (158 / 11575)	0·95 (4 / 420)
Taxane	2·63 (1008 / 38389)	1·65 (188 / 11388)	0·76 (3 / 396)
Rate ratio, from	0·87 CI 0·79 – 0·95	0·80 CI 0·64 – 0·99	1·83 CI 0·36 – 9·22
(O–E) / V	–63·8 / 443·9	–19·1 / 83·3	0·9 / 1·5

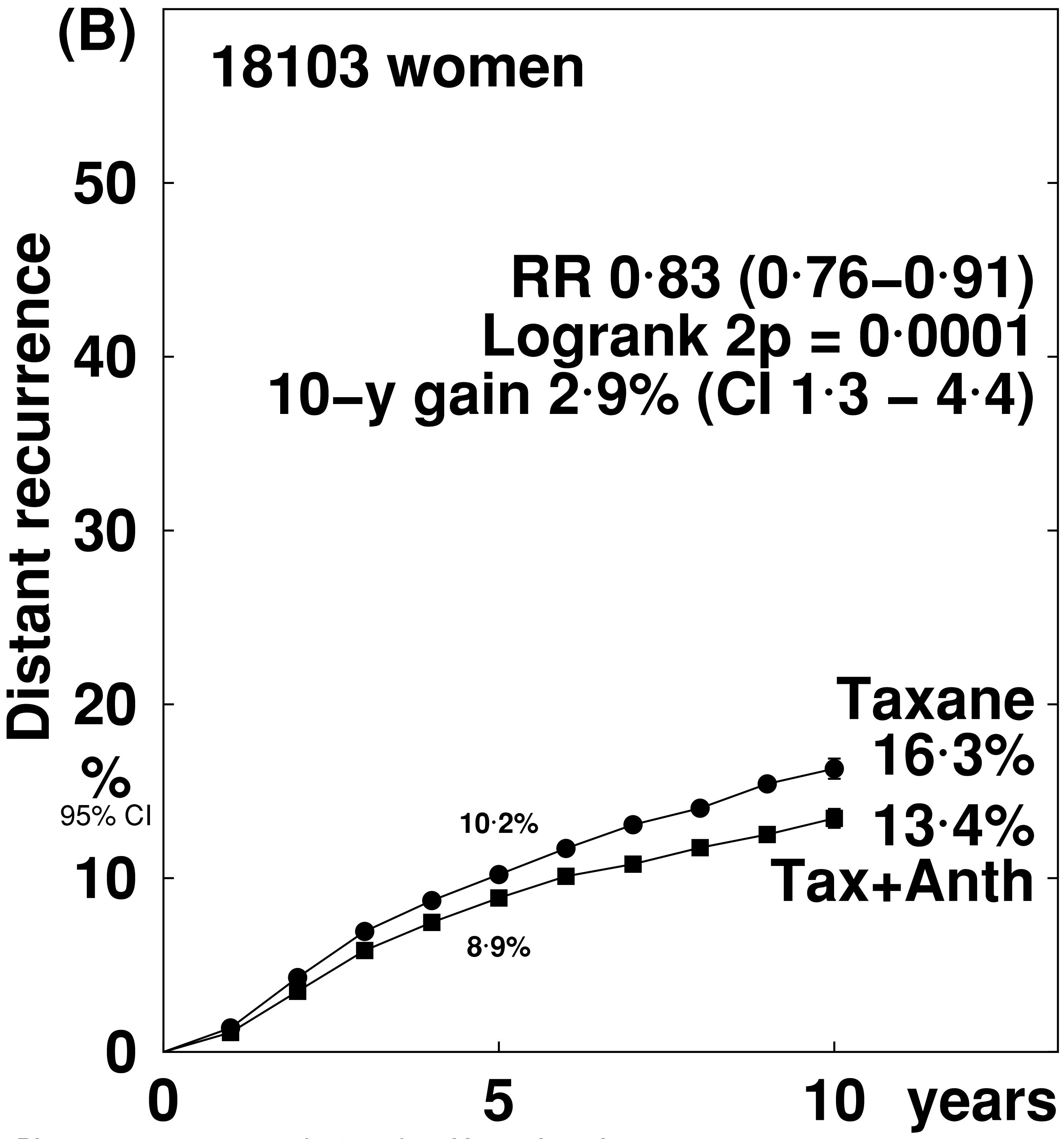

Distant recurrence rates (% / year) and logrank analyses

Allocation	Years 0 – 4	Years 5 – 9	Year 10+
Tax+Anth	1·88 (732 / 38875)	1·10 (131 / 11893)	0·23 (1 / 434)
Taxane	2·15 (833 / 38790)	1·46 (171 / 11714)	0·73 (3 / 412)
Rate ratio, from	0·86 CI 0·77 – 0·95	0·73 CI 0·58 – 0·91	0·42 CI 0·06 – 3·02
(O–E) / V	–56·8 / 368·4	–23·2 / 72·9	–0·9 / 1·0

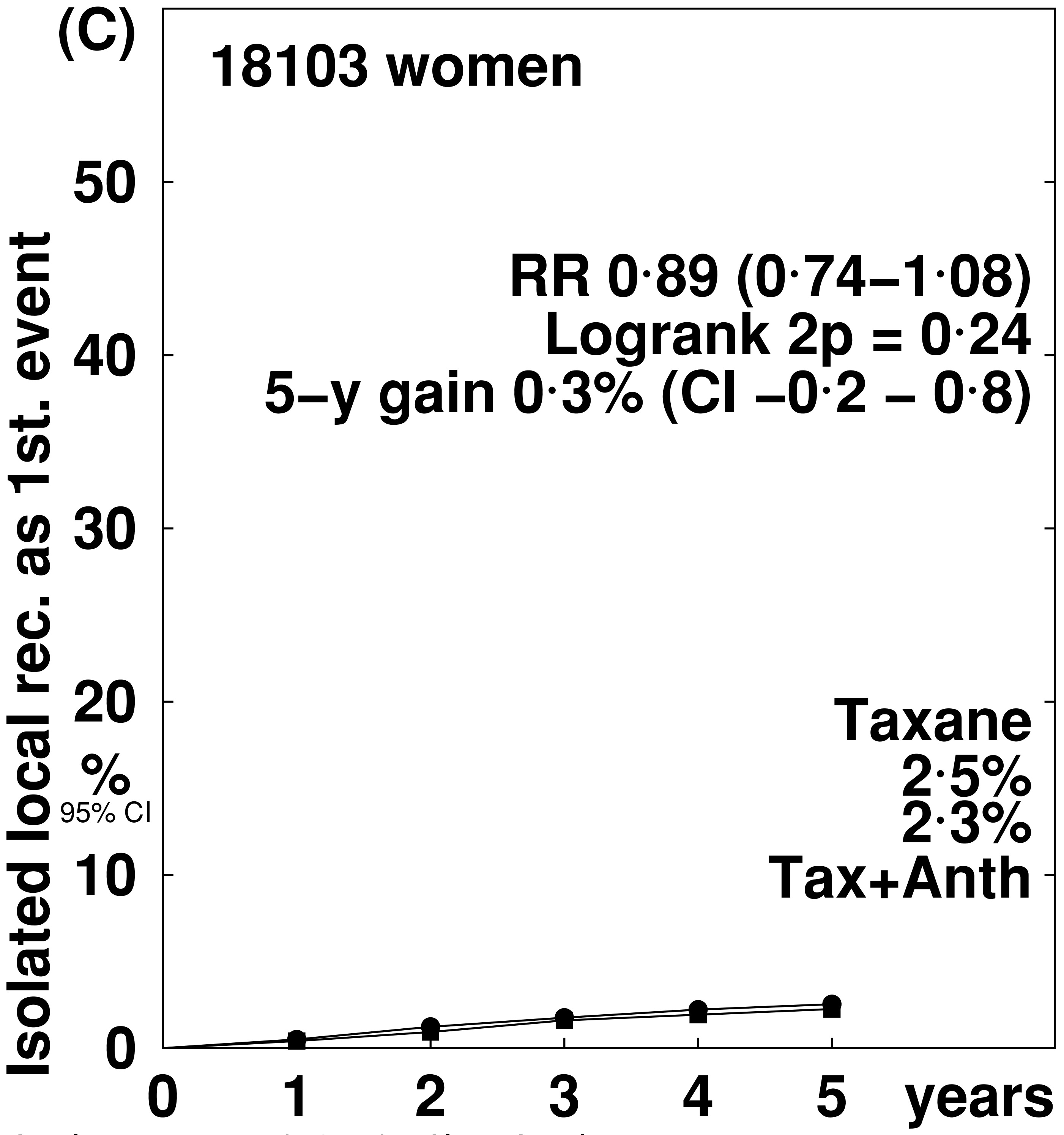

Local recurrence rates (% / year) and logrank analyses

Allocation	Years 0 – 1	Years 2 – 4	Year 5+
Tax+Anth	0·47 (81 / 17098)	0·47 (101 / 21416)	0·27 (32 / 11995)
Taxane	0·61 (104 / 17041)	0·44 (94 / 21347)	0·28 (33 / 11784)
Rate ratio, from	0·76 CI 0·56 – 1·02	1·02 CI 0·77 – 1·36	0·95 CI 0·58 – 1·55
(O–E) / V	–12·3 / 44·1	1·0 / 47·0	–0·9 / 15·7

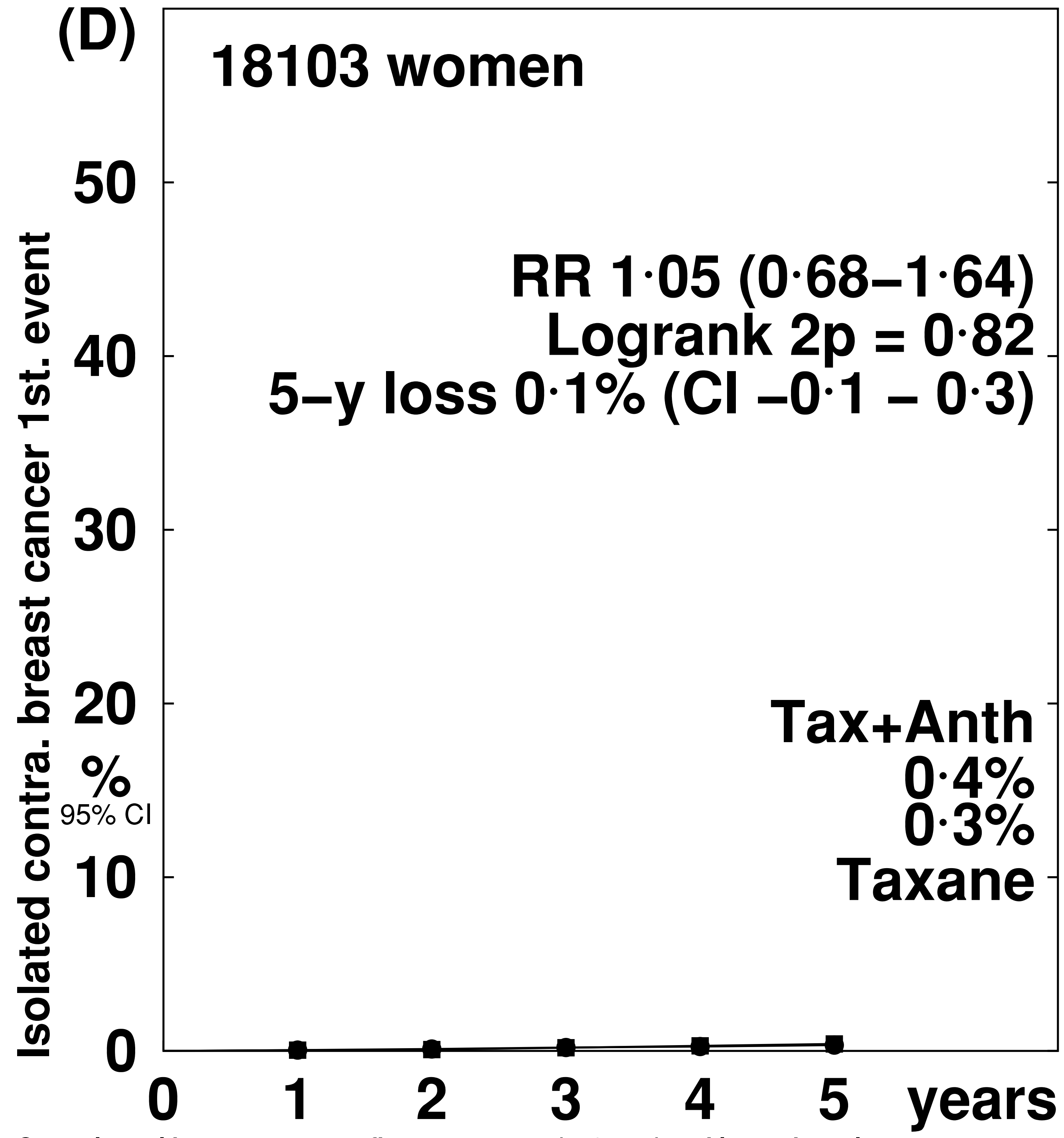

Contralateral breast cancer as first event rates (% / year) and logrank analyses

Allocation	Years 0 – 1	Years 2 – 4	Year 5+
Tax+Anth	0·04 (7 / 17098)	0·11 (23 / 21416)	0·10 (12 / 11995)
Taxane	0·05 (9 / 17041)	0·07 (16 / 21347)	0·11 (13 / 11784)
Rate ratio, from	0·71 CI 0·26 – 1·93	1·44 CI 0·76 – 2·72	0·83 CI 0·37 – 1·83
(O–E) / V	–1·3 / 3·9	3·5 / 9·5	–1·2 / 6·1

P7: Distant recurrence at any time in trials of taxane plus anthracycline versus taxane without anthracycline

Year code	Study name	Regimens compared and drug doses (mg/m²) per cycle. Any unstated doses are as in the non–anthracycline comparator	Cumulative doses Taxane	Cumulative doses Anth	Events/Women Allocated Tax+Anth	Events/Women Adjusted Taxane	Tax+Anth events* Logrank O–E	Tax+Anth events* Variance of O–E	Ratio of annual event rates Tax+Anth : Taxane
**(a) Concurrent docetaxel plus anthracycline vs same dose docetaxel plus cyclophosphamide**									
07&	USO 06090 / 11271	6(A50D75C500 v D75C600)q3	D450 vs D450	A300	53/647	95/649	−19·1	36·0	
09J	NSABP B–46–I	6(A50D75C500 v D75C600)q3	D450 vs D450	A300	34/538	55/539	−10·4	20·9	
09R	NATT Shanghai	6((A50/E60) D75C500 v D75C600)q3‡	D450 vs D450	A300/E360	9/51	21/45	−7·5	6·0	
■	**(a) subtotal**				**96/ 1236 (7·8%)**	**171/ 1233 (13·9%)**	**−37·0**	**62·9**	**0·56 (0·43 – 0·71) reduction 2p < 0·00001**
**(b) Sequential taxane plus anthracycline versus higher cumulative dose docetaxel plus cyclophosphamide**									
08L	DBCG 07 READ	(3E90C600;3D100 v 6D75C600)q3	D300 vs D450	E270	92/1001	101/1011	−6·9	45·7	
09&	Kanagawa Japan	(3FE100C500;3D100 v 6D75C600)q3‡	D300 vs D450	E300	4/53	10/50	−3·2	3·3	
09=	NSABP B–49	(4A60Cq2/q3;12P80q1/4P175q2)/(6A50D75C500q3) v 6D75C600q3	D450/P700–960 vs D450	A240–300	54/932	66/938	−6·1	29·0	
09E1	LMU SUCCESS C	(3FE100C500; 3D100 v 6D75C600)q3	D300 vs D450	E300	138/1816	157/1827	−10·5	70·2	
09M	WSG Plan B	(4E90C600;4D100 v 6D75C600)q3	D400 vs D450	E360	72/1227	65/1222	4·4	32·2	
09U	JBCRG–10 *	(4D75C600Trz; 4FE100C500 or v.v v 6D75C600Trz)q3‡	D300 vs D450	E300	2/42	2(1/25)	0·0	0·6	
09V	JBCRG–09 *	(3D75C600; 3FE100C500 or v.v v 6D75C600)q3‡	D225 vs D450	E300	16/128	2(7/67)	1·0	5·1	
10V	MASTER, Fudan	(3FE100C500;3D100 v 6D75C600)q3	D300 vs D450	E300	37/523	34/524	1·2	17·0	
■	**(b) subtotal**				**415/ 5722 (7·3%)**	**449/ 5756 (7·8%)**	**−20·0**	**203·1**	**0·91 (0·79 – 1·04) reduction 2p > 0.1; NS**
**(c) Taxane plus anthracycline versus higher taxane ± capecitabine**									
00W	N–SAS–BC 02 Japan	(4A60C600;4P175 v 8P175)q3	P700 vs P1400	A240	79/263	102/267	−14·3	41·7	
00W	N–SAS–BC 02 Japan	(4A60C600;4D75 v 8D75)q3	D300 vs D600	A240	69/265	76/265	−3·3	33·9	
01V	Multicentre France	(6E100D75 v 8D100)q3	D450 vs D800	E600	*(21 patients)*		*(no data)*		
05]	Asan, Seoul	(4A60C600;4D75 v 4Vrb25d1,8Cap2000d1–14;4D75)q3‡	D300 vs D300	A240	*(75 patients)*		*(no data)*		
06Z3	MINDACT:EORTC10041	(3FE100C500;3D100 v 6D75Cap1650d1–14)q3	D300 vs D450	E300	8/194	11/198	−1·5	4·6	
09[2	Remagus 04, France	(4FE100C; 4D100 v 6D75Cap)q3‡	D400 vs D450	E400	*(100 patients)*		*(no data)*		
11N	PUMCH–Breast–TCX	6((A50/E75)D75C500 v D75C500Cap950d1–14)q3	D450 vs D450	A300/E450	*(400 patients)*		*(no data)*		
■	**(c) subtotal with data §**				**156/ 722 (21·6%)**	**189/ 730 (25·9%)**	**−19·1**	**80·3**	**0·79 (0·63 – 0·98) reduction 2p = 0·03**
**(d) Taxane plus anthracycline versus higher taxane plus carboplatin (confounded)**									
01M	BCIRG 006	(4A60C600;4D100 v 6D75CptAUC6)q3+12Trzq1; Trzq3 to 1yr	D400 vs D450	A240	164/1074	186/1075	−12·2	83·3	
06~	CAMS NeoPaclitaxel	6(P175E75 v P175CptAUC5)q3	P1050 vs P1050	E450	*(80 patients)*		*(no data)*		
09T	TRYPHAENA	6PtzTrz+(3FE100C500;3D75–100 v 6D75CptAUC6)q3‡	D225–300 vs D450	E300	*(225 patients)*		*(no data)*		
10U	CH–BC–007	(4E90C600; 4D75/P175 v 6D75/P175CptAUC5)q3	D300 v D450 or P700 vs P1050	E360	*(298 patients)*		*(no data)*		
11M	CH–BC–012	(4E80C600;4P175 v 8P150CptAUC3)q2	P700 vs P1200	E360	*(132 patients)*		*(no data)*		
11R	PATTERN, Fudan	3FE100C500;3D100q3 v 6(P80CptAUC2)d1,8,15q4	D300 vs P1440	E300	33/322	20/325	7·4	12·7	
■	**(d) subtotal with data §**				**197/ 1396 (14·1%)**	**206/ 1400 (14·7%)**	**−4·8**	**96·0**	**0·95 (0·78 – 1·16) reduction 2p > 0.1; NS**
■	**Total (a + b + c + d)**				**864/ 9076 (9·5%)**	**1015/ 9119 (11·1%)**	**−80·9**	**442·3**	**0·833 (0·759 – 0·914) reduction 2p = 0·0001**

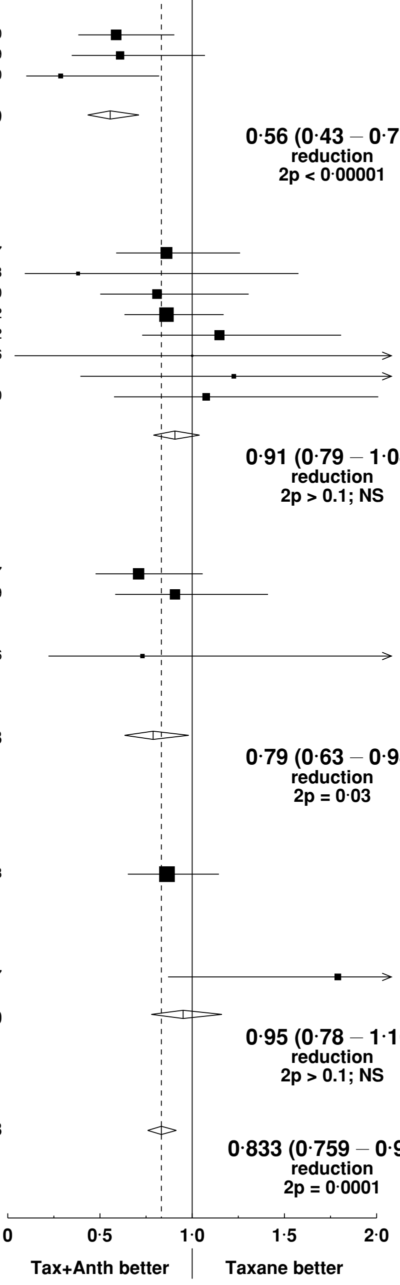

■ 99% or ◇ 95% confidence intervals

Heterogeneity between 4 subtotals: χ^2_3 = 13·7; p = 0·003

Heterogeneity within subtotals: χ^2_{12} = 15·9; p > 0.1; NS

Heterogeneity between 16 trials: χ^2_{15} = 29·6; p = 0·01

* For balance, control patients in 3–way trials or trial strata count half or twice in subtotal(s) and in final total of events/patients.

‡ Pre–operative chemotherapy

v.v Vice versa

Taxanes: D = docetaxel; P = paclitaxel

Anthracyclines: A = doxorubicin (Adriamycin); E = epirubicin

Other agents: C = cyclophosphamide; F = fluorouracil; M = methotrexate; Trz = trastuzumab; Vrb = vinorelbine; Cap = capecitabine; Cpt = carboplatin; Ptz = pertuzumab

P8: Isolated local recurrence as first event in trials of taxane plus anthracycline versus taxane without anthracycline

Year code	and study name	Regimens compared and drug doses (mg/m²) per cycle. Any unstated doses are as in the non–anthracycline comparator	Cumulative doses Taxane	Cumulative doses Anth	Events/Women Allocated Tax+Anth	Events/Women Adjusted Taxane	Tax+Anth events* Logrank O–E	Tax+Anth events* Variance of O–E	Ratio of annual event rates Tax+Anth : Taxane
**(a) Concurrent docetaxel plus anthracycline vs same dose docetaxel plus cyclophosphamide**									
07&	USO 06090 / 11271	$6(A_{50}D_{75}C_{500}$ v $D_{75}C_{600})_{q3}$	D_{450} vs D_{450}	A_{300}	15/647	27/649	–5·7	10·2	
09J	NSABP B–46–I	$6(A_{50}D_{75}C_{500}$ v $D_{75}C_{600})_{q3}$	D_{450} vs D_{450}	A_{300}	8/538	14/539	–2·9	5·4	
09R	NATT Shanghai	$6((A_{50}/E_{60})\ D_{75}C_{500}$ v $D_{75}C_{600})_{q3}$‡	D_{450} vs D_{450}	A_{300}/E_{360}	6/51	12/45	–3·5	3·4	
■	**(a) subtotal**				**29/ 1236 (2·3%)**	**53/ 1233 (4·3%)**	**–12·0**	**19·0**	**0·53 (0·34 – 0·83) reduction 2p = 0·006**
**(b) Sequential taxane plus anthracycline versus higher cumulative dose docetaxel plus cyclophosphamide**									
08L	DBCG 07 READ	$(3E_{90}C_{600};3D_{100}$ v $6D_{75}C_{600})_{q3}$	D_{300} vs D_{450}	E_{270}	20/1001	14/1011	2·4	8·3	
09&	Kanagawa Japan	$(3FE_{100}C_{500};3D_{100}$ v $6D_{75}C_{600})_{q3}$‡	D_{300} vs D_{450}	E_{300}	1/53	0/50	0·5	0·2	
09=	NSABP B–49	$(4A_{60}C_{q2/q3};12P_{80q1}/4P_{175q2})/(6A_{50}D_{75}C_{500q3})$ v $6D_{75}C_{600q3}$	$D_{450}/P_{700-960}$ vs D_{450}	$A_{240-300}$	18/932	22/938	–2·1	9·8	
09E1	LMU SUCCESS C	$(3FE_{100}C_{500};\ 3D_{100}$ v $6D_{75}C_{600})_{q3}$	D_{300} vs D_{450}	E_{300}	28/1816	22/1827	3·0	12·4	
09M	WSG Plan B	$(4E_{90}C_{600};4D_{100}$ v $6D_{75}C_{600})_{q3}$	D_{400} vs D_{450}	E_{360}	20/1227	24/1222	–2·1	10·6	
09U	JBCRG–10 *	$(4D_{75}C_{600}Trz;\ 4FE_{100}C_{500}$ or v.v v $6D_{75}C_{600}Trz)_{q3}$‡	D_{300} vs D_{450}	E_{300}	2/42	2(0/25)	0·5	0·2	
09V	JBCRG–09 *	$(3D_{75}C_{600};\ 3FE_{100}C_{500}$ or v.v v $6D_{75}C_{600})_{q3}$‡	D_{225} vs D_{450}	E_{300}	4/128	2(5/67)	–1·9	2·0	
10V	MASTER, Fudan	$(3FE_{100}C_{500};3D_{100}$ v $6D_{75}C_{600})_{q3}$	D_{300} vs D_{450}	E_{300}	8/523	3/524	2·8	2·7	
■	**(b) subtotal**				**101/ 5722 (1·8%)**	**95/ 5756 (1·7%)**	**3·2**	**46·3**	**1·07 (0·80 – 1·43) increase 2p > 0.1; NS**
**(c) Taxane plus anthracycline versus higher taxane ± capecitabine**									
00W	N–SAS–BC 02 Japan	$(4A_{60}C_{600};4P_{175}$ v $8P_{175})_{q3}$	P_{700} vs P_{1400}	A_{240}	20/263	25/267	–3·0	10·7	
00W	N–SAS–BC 02 Japan	$(4A_{60}C_{600};4D_{75}$ v $8D_{75})_{q3}$	D_{300} vs D_{600}	A_{240}	11/265	15/265	–2·1	6·4	
01V	Multicentre France	$(6E_{100}D_{75}$ v $8D_{100})_{q3}$	D_{450} vs D_{800}	E_{600}	*(21 patients)*		*(no data)*		
05]	Asan, Seoul	$(4A_{60}C_{600};4D_{75}$ v $4Vrb_{25d1,8}Cap_{2000d1-14};4D_{75})_{q3}$‡	D_{300} vs D_{300}	A_{240}	*(75 patients)*		*(no data)*		
06Z3	MINDACT:EORTC10041	$(3FE_{100}C_{500};3D_{100}$ v $6D_{75}Cap_{1650d1-14})_{q3}$	D_{300} vs D_{450}	E_{300}	2/194	3/198	–0·6	1·2	
09[2	Remagus 04, France	$(4FE_{100}C;\ 4D_{100}$ v $6D_{75}Cap)_{q3}$‡	D_{400} vs D_{450}	E_{400}	*(100 patients)*		*(no data)*		
11N	PUMCH–Breast–TCX	$6((A_{50}/E_{75})D_{75}C_{500}$ v $D_{75}C_{500}Cap_{950d1-14})_{q3}$	D_{450} vs D_{450}	A_{300}/E_{450}	*(400 patients)*		*(no data)*		
■	**(c) subtotal with data §**				**33/ 722 (4·6%)**	**43/ 730 (5·9%)**	**–5·7**	**18·4**	**0·73 (0·46 – 1·16) reduction 2p > 0.1; NS**
**(d) Taxane plus anthracycline versus higher taxane plus carboplatin (confounded)**									
01M	BCIRG 006	$(4A_{60}C_{600};4D_{100}$ v $6D_{75}Cpt_{AUC6})_{q3}+12Trz_{q1};\ Trz_{q3}$ to 1yr	D_{400} vs D_{450}	A_{240}	41/1074	41/1075	–0·4	19·9	
06~	CAMS NeoPaclitaxel	$6(P_{175}E_{75}$ v $P_{175}Cpt_{AUC5})_{q3}$	P_{1050} vs P_{1050}	E_{450}	*(80 patients)*		*(no data)*		
09T	TRYPHAENA	$6PtzTrz+(3FE_{100}C_{500};3D_{75-100}$ v $6D_{75}Cpt_{AUC6})_{q3}$‡	$D_{225-300}$ vs D_{450}	E_{300}	*(225 patients)*		*(no data)*		
10U	CH–BC–007	$(4E_{90}C_{600};\ 4D_{75}/P_{175}$ v $6D_{75}/P_{175}Cpt_{AUC5})_{q3}$	D_{300} v D_{450} or P_{700} vs P_{1050}	E_{360}	*(298 patients)*		*(no data)*		
11M	CH–BC–012	$(4E_{80}C_{600};4P_{175}$ v $8P_{150}Cpt_{AUC3})_{q2}$	P_{700} vs P_{1200}	E_{360}	*(132 patients)*		*(no data)*		
11R	PATTERN, Fudan	$3FE_{100}C_{500};3D_{100q3}$ v $6(P_{80}Cpt_{AUC2})_{d1,8,15q4}$	D_{300} vs P_{1440}	E_{300}	10/322	4/325	2·7	3·2	
■	**(d) subtotal with data §**				**51/ 1396 (3·7%)**	**45/ 1400 (3·2%)**	**2·4**	**23·1**	**1·11 (0·74 – 1·67) increase 2p > 0.1; NS**
■	**Total (a + b + c + d)**				**214/ 9076 (2·4%)**	**236/ 9119 (2·6%)**	**–12·2**	**106·8**	**0·892 (0·738 – 1·079) reduction 2p > 0.1; NS**

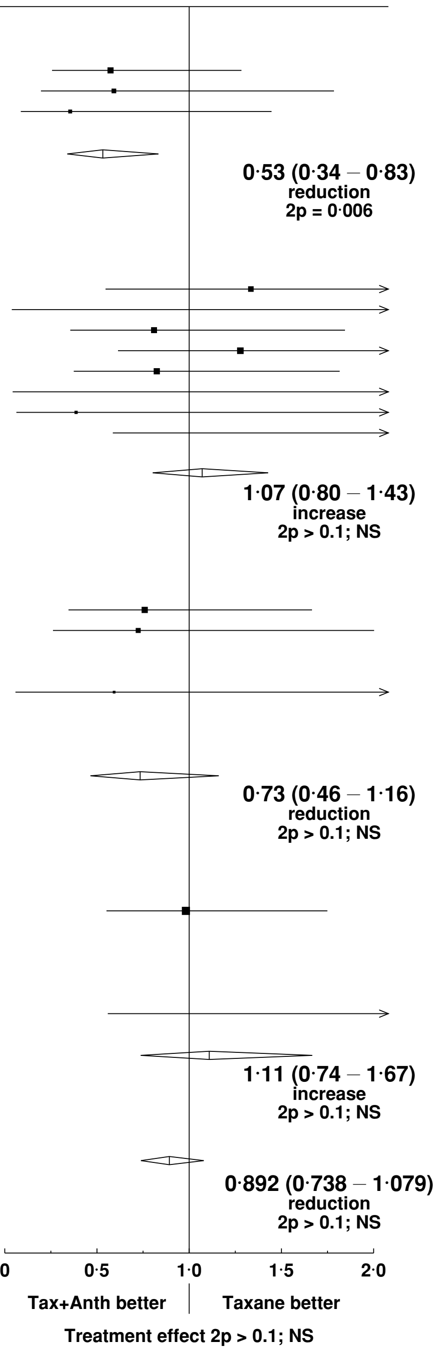

■ 99% or ◇ 95% confidence intervals

Heterogeneity between 4 subtotals: $\chi^2_3 = 8{\cdot}4$; p = 0·04

Heterogeneity within subtotals: $\chi^2_{12} = 11{\cdot}5$; p > 0.1; NS

Heterogeneity between 16 trials: $\chi^2_{15} = 19{\cdot}9$; p > 0.1; NS

* For balance, control patients in 3–way trials or trial strata count half or twice in subtotal(s) and in final total of events/patients.

‡ Pre–operative chemotherapy

v.v Vice versa

Taxanes: D = docetaxel; P = paclitaxel

Anthracyclines: A = doxorubicin (Adriamycin); E = epirubicin

Other agents: C = cyclophosphamide; F = fluorouracil; M = methotrexate; Trz = trastuzumab; Vrb = vinorelbine; Cap = capecitabine; Cpt = carboplatin; Ptz = pertuzumab

P9: Contralateral recurrence as first event in trials of taxane plus anthracycline versus taxane without anthracycline

Year code	Study name	Regimens compared and drug doses (mg/m²) per cycle. Any unstated doses are as in the non–anthracycline comparator	Cumulative doses: Taxane	Cumulative doses: Anth	Events/Women: Allocated Tax+Anth	Events/Women: Adjusted Taxane	Tax+Anth events*: Logrank O–E	Tax+Anth events*: Variance of O–E	Ratio of annual event rates Tax+Anth : Taxane
**(a) Concurrent docetaxel plus anthracycline vs same dose docetaxel plus cyclophosphamide**									
07&	USO 06090 / 11271	6(A50D75C500 v D75C600)q3	D450 vs D450	A300	2/647	3/649	−0·5	1·2	
09J	NSABP B–46–I	6(A50D75C500 v D75C600)q3	D450 vs D450	A300	2/538	5/539	−1·0	1·7	
09R	NATT Shanghai	6((A50/E60) D75C500 v D75C600)q3‡	D450 vs D450	A300/E360	1/51	0/45	0·3	0·2	
	**(a) subtotal**				**5/ 1236 (0·4%)**	**8/ 1233 (0·6%)**	**−1·2**	**3·1**	**0·68 (0·22 – 2·05) reduction 2p > 0.1; NS**
**(b) Sequential taxane plus anthracycline versus higher cumulative dose docetaxel plus cyclophosphamide**									
08L	DBCG 07 READ	(3E90C600;3D100 v 6D75C600)q3	D300 vs D450	E270	16/1001	12/1011	0·9	6·9	
09&	Kanagawa Japan	(3FE100C500;3D100 v 6D75C600)q3‡	D300 vs D450	E300	0/53	0/50			
09=	NSABP B–49	(4A60Cq2/q3;12P80q1/4P175q2)/(6A50D75C500q3) v 6D75C600q3	D450/P700–960 vs D450	A240–300	6/932	1/938	2·0	1·7	
09E1	LMU SUCCESS C	(3FE100C500; 3D100 v 6D75C600)q3	D300 vs D450	E300	0/1816	0/1827			
09M	WSG Plan B	(4E90C600;4D100 v 6D75C600)q3	D400 vs D450	E360	2/1227	1/1222	0·5	0·7	
09U	JBCRG–10 *	(4D75C600Trz; 4FE100C500 or v.v v 6D75C600Trz)q3‡	D300 vs D450	E300	0/42	2(0/25)			
09V	JBCRG–09 *	(3D75C600; 3FE100C500 or v.v v 6D75C600)q3‡	D225 vs D450	E300	0/128	2(0/67)			
10V	MASTER, Fudan	(3FE100C500;3D100 v 6D75C600)q3	D300 vs D450	E300	0/523	0/524			
	**(b) subtotal**				**24/ 5722 (0·4%)**	**14/ 5756 (0·2%)**	**3·4**	**9·3**	**1·45 (0·76 – 2·76) increase 2p > 0.1; NS**
**(c) Taxane plus anthracycline versus higher taxane ± capecitabine**									
00W	N–SAS–BC 02 Japan	(4A60C600;4P175 v 8P175)q3	P700 vs P1400	A240	0/263	0/267			
00W	N–SAS–BC 02 Japan	(4A60C600;4D75 v 8D75)q3	D300 vs D600	A240	0/265	0/265			
01V	Multicentre France	(6E100D75 v 8D100)q3	D450 vs D800	E600	*(21 patients)*		*(no data)*		
05]	Asan, Seoul	(4A60C600;4D75 v 4Vrb25d1,8Cap2000d1–14;4D75)q3‡	D300 vs D300	A240	*(75 patients)*		*(no data)*		
06Z3	MINDACT:EORTC10041	(3FE100C500;3D100 v 6D75Cap1650d1–14)q3	D300 vs D450	E300	1/194	1/198	−0·0	0·5	
09[2	Remagus 04, France	(4FE100C; 4D100 v 6D75Cap)q3‡	D400 vs D450	E400	*(100 patients)*		*(no data)*		
11N	PUMCH–Breast–TCX	6((A50/E75)D75C500 v D75C500Cap950d1–14)q3	D450 vs D450	A300/E450	*(400 patients)*		*(no data)*		
	**(c) subtotal with data §**				**1/ 722 (0·1%)**	**1/ 730 (0·1%)**	**−0·0**	**0·5**	**0·94 (0·06 – 15·15) reduction 2p > 0.1; NS**
**(d) Taxane plus anthracycline versus higher taxane plus carboplatin (confounded)**									
01M	BCIRG 006	(4A60C600;4D100 v 6D75CptAUC6)q3+12Trzq1; Trzq3 to 1yr	D400 vs D450	A240	4/1074	6/1075	−1·1	2·5	
06~	CAMS NeoPaclitaxel	6(P175E75 v P175CptAUC5)q3	P1050 vs P1050	E450	*(80 patients)*		*(no data)*		
09T	TRYPHAENA	6PtzTrz+(3FE100C500;3D75–100 v 6D75CptAUC6)q3‡	D225–300 vs D450	E300	*(225 patients)*		*(no data)*		
10U	CH–BC–007	(4E90C600; 4D75/P175 v 6D75/P175CptAUC5)q3	D300 v D450 or P700 vs P1050	E360	*(298 patients)*		*(no data)*		
11M	CH–BC–012	(4E80C600;4P175 v 8P150CptAUC3)q2	P700 vs P1200	E360	*(132 patients)*		*(no data)*		
11R	PATTERN, Fudan	3FE100C500;3D100q3 v 6(P80CptAUC2)d1,8,15q4	D300 vs P1440	E300	8/322	9/325	−0·1	4·1	
	**(d) subtotal with data §**				**12/ 1396 (0·9%)**	**15/ 1400 (1·1%)**	**−1·2**	**6·6**	**0·84 (0·39 – 1·80) reduction 2p > 0.1; NS**
	**Total (a + b + c + d)**				**42/ 9076 (0·5%)**	**38/ 9119 (0·4%)**	**1·0**	**19·5**	**1·053 (0·676 – 1·642) increase 2p > 0.1; NS**

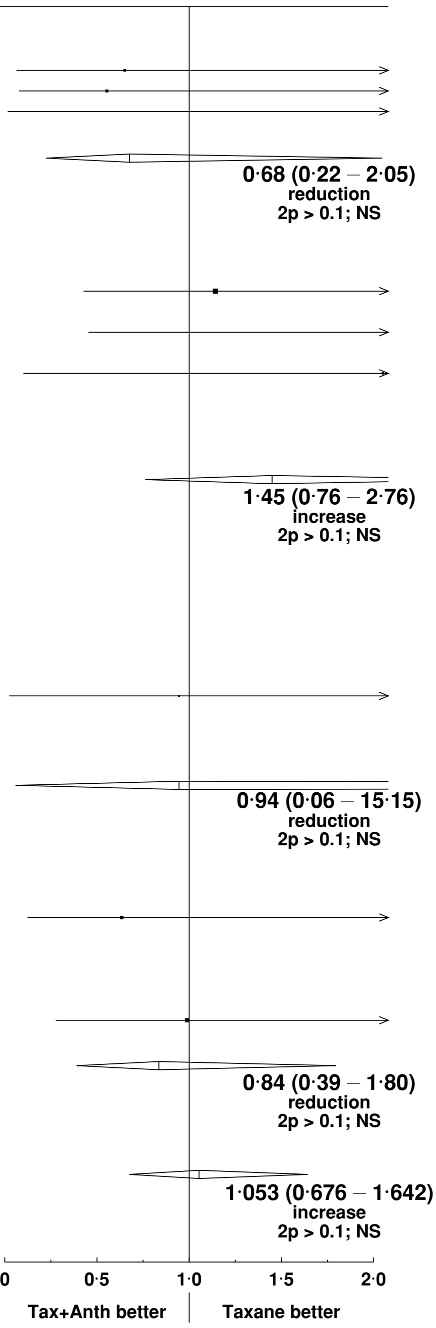

■ 99% or ◇ 95% confidence intervals

Heterogeneity between 4 subtotals: χ^2_3 = 1·9; p > 0.1; NS

Heterogeneity within subtotals: χ^2_5 = 2·7; p > 0.1; NS

Heterogeneity between 9 trials: χ^2_8 = 4·6; p > 0.1; NS

Treatment effect 2p > 0.1; NS, adverse

* For balance, control patients in 3–way trials or trial strata count half or twice in subtotal(s) and in final total of events/patients.

‡ Pre–operative chemotherapy

v.v Vice versa

Taxanes: D = docetaxel; P = paclitaxel

Anthracyclines: A = doxorubicin (Adriamycin); E = epirubicin

Other agents: C = cyclophosphamide; F = fluorouracil; M = methotrexate; Trz = trastuzumab; Vrb = vinorelbine; Cap = capecitabine; Cpt = carboplatin; Ptz = pertuzumab

P10: Breast cancer mortality in trials of taxane plus anthracycline versus taxane without anthracycline

Year code and study name		Regimens compared and drug doses (mg/m²) per cycle. Any unstated doses are as in the non–anthracycline comparator	Cumulative doses Taxane	Anth	Deaths/Women Allocated Tax+Anth	Deaths/Women Adjusted Taxane	Tax+Anth deaths* Logrank O–E	Tax+Anth deaths* Variance of O–E
**(a) Concurrent docetaxel plus anthracycline vs same dose docetaxel plus cyclophosphamide**								
07&	USO 06090 / 11271	6(A50D75C500 v D75C600)q3	D450 vs D450	A300	48/647	72/649	−9·5	28·8
09J	NSABP B–46–I	6(A50D75C500 v D75C600)q3	D450 vs D450	A300	25/538	30/539	−2·2	12·9
09R	NATT Shanghai	6((A50/E60) D75C500 v D75C600)q3‡	D450 vs D450	A300/E360	5/51	18/45	−8·3	4·8
■	**(a) subtotal**				**78/ 1236 (6·3%)**	**120/ 1233 (9·7%)**	**−20·0**	**46·5**
**(b) Sequential taxane plus anthracycline versus higher cumulative dose docetaxel plus cyclophosphamide**								
08L	DBCG 07 READ	(3E90C600;3D100 v 6D75C600)q3	D300 vs D450	E270	73/1001	79/1011	−6·0	36·2
09&	Kanagawa Japan	(3FE100C500;3D100 v 6D75C600)q3‡	D300 vs D450	E300	4/53	8/50	−2·2	2·9
09=	NSABP B–49	(4A60Cq2/q3;12P80q1/4P175q2)/(6A50D75C500q3) v 6D75C600q3	D450/P700–960 vs D450	A240–300	32/932	32/938	−0·7	15·3
09E1	LMU SUCCESS C	(3FE100C500; 3D100 v 6D75C600)q3	D300 vs D450	E300	75/1816	84/1827	−5·6	38·1
09M	WSG Plan B	(4E90C600;4D100 v 6D75C600)q3	D400 vs D450	E360	49/1227	44/1222	2·3	22·0
09U	JBCRG–10 *	(4D75C600Trz; 4FE100C500 or v.v v 6D75C600Trz)q3‡	D300 vs D450	E300	1/42	2(1/25)	−0·2	0·5
09V	JBCRG–09 *	(3D75C600; 3FE100C500 or v.v v 6D75C600)q3‡	D225 vs D450	E300	6/128	2(4/67)	−0·4	2·2
10V	MASTER, Fudan	(3FE100C500;3D100 v 6D75C600)q3	D300 vs D450	E300	23/523	17/524	1·9	9·5
■	**(b) subtotal**				**263/ 5722 (4·6%)**	**274/ 5756 (4·8%)**	**−10·8**	**126·6**
**(c) Taxane plus anthracycline versus higher taxane ± capecitabine**								
00W	N–SAS–BC 02 Japan	(4A60C600;4P175 v 8P175)q3	P700 vs P1400	A240	49/263	48/267	0·2	23·2
00W	N–SAS–BC 02 Japan	(4A60C600;4D75 v 8D75)q3	D300 vs D600	A240	37/265	36/265	1·0	17·6
01V	Multicentre France	(6E100D75 v 8D100)q3	D450 vs D800	E600	*(21 patients)*		*(no data)*	
05]	Asan, Seoul	(4A60C600;4D75 v 4Vrb25d1,8Cap2000d1–14;4D75)q3‡	D300 vs D300	A240	*(75 patients)*		*(no data)*	
06Z3	MINDACT:EORTC10041	(3FE100C500;3D100 v 6D75Cap1650d1–14)q3	D300 vs D450	E300	2/194	6/198	−2·2	1·9
09[2	Remagus 04, France	(4FE100C; 4D100 v 6D75Cap)q3‡	D400 vs D450	E400	*(100 patients)*		*(no data)*	
11N	PUMCH–Breast–TCX	6((A50/E75)D75C500 v D75C500Cap950d1–14)q3	D450 vs D450	A300/E450	*(400 patients)*		*(no data)*	
■	**(c) subtotal with data §**				**88/ 722 (12·2%)**	**90/ 730 (12·3%)**	**−1·0**	**42·7**
**(d) Taxane plus anthracycline versus higher taxane plus carboplatin (confounded)**								
01M	BCIRG 006	(4A60C600;4D100 v 6D75CptAUC6)q3+12Trzq1; Trzq3 to 1yr	D400 vs D450	A240	121/1074	142/1075	−10·8	63·3
06~	CAMS NeoPaclitaxel	6(P175E75 v P175CptAUC5)q3	P1050 vs P1050	E450	*(80 patients)*		*(no data)*	
09T	TRYPHAENA	6PtzTrz+(3FE100C500;3D75–100 v 6D75CptAUC6)q3‡	D225–300 vs D450	E300	*(225 patients)*		*(no data)*	
10U	CH–BC–007	(4E90C600; 4D75/P175 v 6D75/P175CptAUC5)q3	D300 v D450 or P700 vs P1050	E360	*(298 patients)*		*(no data)*	
11M	CH–BC–012	(4E80C600;4P175 v 8P150CptAUC3)q2	P700 vs P1200	E360	*(132 patients)*		*(no data)*	
11R	PATTERN, Fudan	3FE100C500;3D100q3 v 6(P80CptAUC2)d1,8,15q4	D300 vs P1440	E300	28/322	19/325	4·9	11·3
■	**(d) subtotal with data §**				**149/ 1396 (10·7%)**	**161/ 1400 (11·5%)**	**−5·9**	**74·6**
■	**Total (a + b + c + d)**				**578/ 9076 (6·4%)**	**645/ 9119 (7·1%)**	**−37·7**	**290·4**

Ratio of annual death rates
Tax+Anth : Taxane

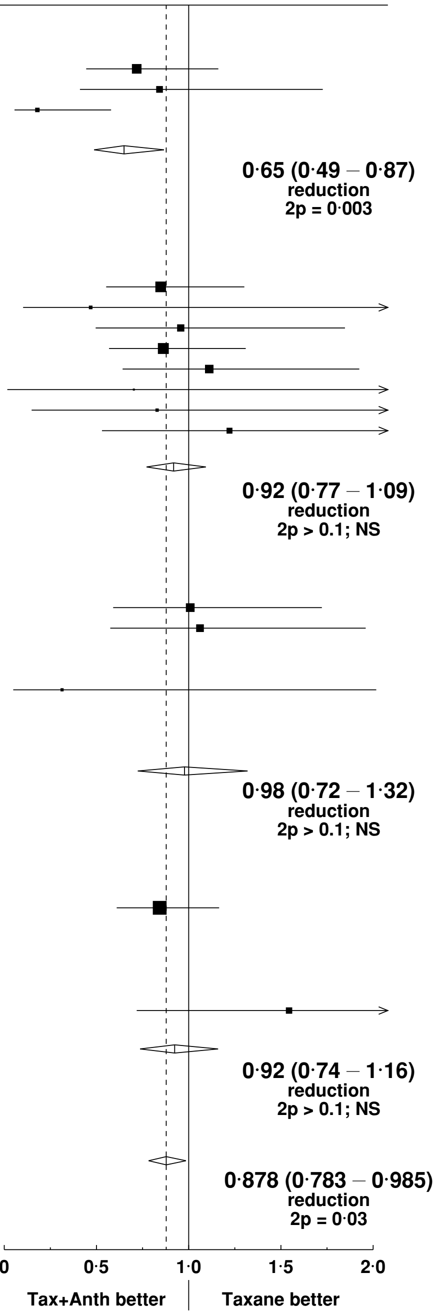

99% or 95% confidence intervals

Heterogeneity between 4 subtotals: χ^2_3 = 5·2; p > 0.1; NS

Heterogeneity within subtotals: χ^2_{12} = 18·6; p = 0·10

Heterogeneity between 16 trials: χ^2_{15} = 23·8; p = 0·07

* For balance, control patients in 3–way trials or trial strata count half or twice in subtotal(s) and in final total of deaths/patients.

‡ Pre–operative chemotherapy

v.v Vice versa

Taxanes: D = docetaxel; P = paclitaxel

Anthracyclines: A = doxorubicin (Adriamycin); E = epirubicin

Other agents: C = cyclophosphamide; F = fluorouracil; M = methotrexate; Trz = trastuzumab; Vrb = vinorelbine; Cap = capecitabine; Cpt = carboplatin; Ptz = pertuzumab

P11: Death without recurrence in year 0 in trials of taxane plus anthracycline versus taxane without anthracycline

Year code and study name	Regimens compared and drug doses (mg/m²) per cycle. Any unstated doses are as in the non–anthracycline comparator	Cumulative doses Taxane	Cumulative doses Anth	Deaths/Women Allocated Tax+Anth	Deaths/Women Adjusted Taxane	Tax+Anth deaths* Logrank O–E	Tax+Anth deaths* Variance of O–E	Ratio of annual death rates Tax+Anth : Taxane
**(a) Concurrent docetaxel plus anthracycline vs same dose docetaxel plus cyclophosphamide**								
07& USO 06090 / 11271	$6(A_{50}D_{75}C_{500}$ v $D_{75}C_{600})_{q3}$	D_{450} vs D_{450}	A_{300}	5/647	0/649	2·6	1·2	
09J NSABP B–46–I	$6(A_{50}D_{75}C_{500}$ v $D_{75}C_{600})_{q3}$	D_{450} vs D_{450}	A_{300}	3/538	4/539	–0·2	1·7	
09R NATT Shanghai	$6((A_{50}/E_{60})\ D_{75}C_{500}$ v $D_{75}C_{600})_{q3}$‡	D_{450} vs D_{450}	A_{300}/E_{360}	0/51	0/45			
**(a) subtotal**				**8/ 1236 (0·6%)**	**4/ 1233 (0·3%)**	**2·4**	**2·9**	**2·31 (0·73 – 7·30) increase 2p > 0.1; NS**
**(b) Sequential taxane plus anthracycline versus higher cumulative dose docetaxel plus cyclophosphamide**								
08L DBCG 07 READ	$(3E_{90}C_{600};3D_{100}$ v $6D_{75}C_{600})_{q3}$	D_{300} vs D_{450}	E_{270}	0/1001	1/1011	–0·5	0·2	
09& Kanagawa Japan	$(3FE_{100}C_{500};3D_{100}$ v $6D_{75}C_{600})_{q3}$‡	D_{300} vs D_{450}	E_{300}	0/53	0/50			
09= NSABP B–49	$(4A_{60}C_{q2/q3};12P_{80q1}/4P_{175q2})/(6A_{50}D_{75}C_{500q3})$ v $6D_{75}C_{600q3}$	$D_{450}/P_{700-960}$ vs D_{450}	$A_{240-300}$	4/932	4/938	0·1	2·0	
09E1 LMU SUCCESS C	$(3FE_{100}C_{500};\ 3D_{100}$ v $6D_{75}C_{600})_{q3}$	D_{300} vs D_{450}	E_{300}	6/1816	0/1827	2·9	1·5	
09M WSG Plan B	$(4E_{90}C_{600};4D_{100}$ v $6D_{75}C_{600})_{q3}$	D_{400} vs D_{450}	E_{360}	1/1227	6/1222	–2·3	1·7	
09U JBCRG–10 *	$(4D_{75}C_{600}Trz;\ 4FE_{100}C_{500}$ or v.v v $6D_{75}C_{600}Trz)_{q3}$‡	D_{300} vs D_{450}	E_{300}	2/42	2(0/25)	0·5	0·2	
09V JBCRG–09 *	$(3D_{75}C_{600};\ 3FE_{100}C_{500}$ or v.v v $6D_{75}C_{600})_{q3}$‡	D_{225} vs D_{450}	E_{300}	0/128	2(0/67)			
10V MASTER, Fudan	$(3FE_{100}C_{500};3D_{100}$ v $6D_{75}C_{600})_{q3}$	D_{300} vs D_{450}	E_{300}	0/523	0/524			
**(b) subtotal**				**13/ 5722 (0·2%)**	**11/ 5756 (0·2%)**	**0·7**	**5·6**	**1·13 (0·49 – 2·57) increase 2p > 0.1; NS**
**(c) Taxane plus anthracycline versus higher taxane ± capecitabine**								
00W N–SAS–BC 02 Japan	$(4A_{60}C_{600};4P_{175}$ v $8P_{175})_{q3}$	P_{700} vs P_{1400}	A_{240}	1/263	0/267	0·5	0·2	
00W N–SAS–BC 02 Japan	$(4A_{60}C_{600};4D_{75}$ v $8D_{75})_{q3}$	D_{300} vs D_{600}	A_{240}	0/265	0/265			
01V Multicentre France	$(6E_{100}D_{75}$ v $8D_{100})_{q3}$	D_{450} vs D_{800}	E_{600}	*(21 patients)*		*(no data)*		
05] Asan, Seoul	$(4A_{60}C_{600};4D_{75}$ v $4Vrb_{25d1,8}Cap_{2000d1-14};4D_{75})_{q3}$‡	D_{300} vs D_{300}	A_{240}	*(75 patients)*		*(no data)*		
06Z3 MINDACT:EORTC10041	$(3FE_{100}C_{500};3D_{100}$ v $6D_{75}Cap_{1650d1-14})_{q3}$	D_{300} vs D_{450}	E_{300}	1/194	1/198	0·0	0·5	
09[2 Remagus 04, France	$(4FE_{100}C;\ 4D_{100}$ v $6D_{75}Cap)_{q3}$‡	D_{400} vs D_{450}	E_{400}	*(100 patients)*		*(no data)*		
11N PUMCH–Breast–TCX	$6((A_{50}/E_{75})D_{75}C_{500}$ v $D_{75}C_{500}Cap_{950d1-14})_{q3}$	D_{450} vs D_{450}	A_{300}/E_{450}	*(400 patients)*		*(no data)*		
**(c) subtotal with data §**				**2/ 722 (0·3%)**	**1/ 730 (0·1%)**	**0·5**	**0·7**	**1·97 (0·20 – 19·08) increase 2p > 0.1; NS**
**(d) Taxane plus anthracycline versus higher taxane plus carboplatin (confounded)**								
01M BCIRG 006	$(4A_{60}C_{600};4D_{100}$ v $6D_{75}Cpt_{AUC6})_{q3}+12Trz_{q1};\ Trz_{q3}$ to 1yr	D_{400} vs D_{450}	A_{240}	1/1074	4/1075	–1·6	1·2	
06~ CAMS NeoPaclitaxel	$6(P_{175}E_{75}$ v $P_{175}Cpt_{AUC5})_{q3}$	P_{1050} vs P_{1050}	E_{450}	*(80 patients)*		*(no data)*		
09T TRYPHAENA	$6PtzTrz+(3FE_{100}C_{500};3D_{75-100}$ v $6D_{75}Cpt_{AUC6})_{q3}$‡	$D_{225-300}$ vs D_{450}	E_{300}	*(225 patients)*		*(no data)*		
10U CH–BC–007	$(4E_{90}C_{600};\ 4D_{75}/P_{175}$ v $6D_{75}/P_{175}Cpt_{AUC5})_{q3}$	D_{300} v D_{450} or P_{700} vs P_{1050}	E_{360}	*(298 patients)*		*(no data)*		
11M CH–BC–012	$(4E_{80}C_{600};4P_{175}$ v $8P_{150}Cpt_{AUC3})_{q2}$	P_{700} vs P_{1200}	E_{360}	*(132 patients)*		*(no data)*		
11R PATTERN, Fudan	$3FE_{100}C_{500};3D_{100q3}$ v $6(P_{80}Cpt_{AUC2})_{d1,8,15q4}$	D_{300} vs P_{1440}	E_{300}	0/322	0/325			
**(d) subtotal with data §**				**1/ 1396 (0·1%)**	**4/ 1400 (0·3%)**	**–1·6**	**1·2**	**0·27 (0·05 – 1·57) reduction 2p > 0.1; NS**
**Total (a + b + c + d)**				**24/ 9076 (0·3%)**	**20/ 9119 (0·2%)**	**2·0**	**10·5**	**1·207 (0·660 – 2·208) increase 2p > 0.1; NS**

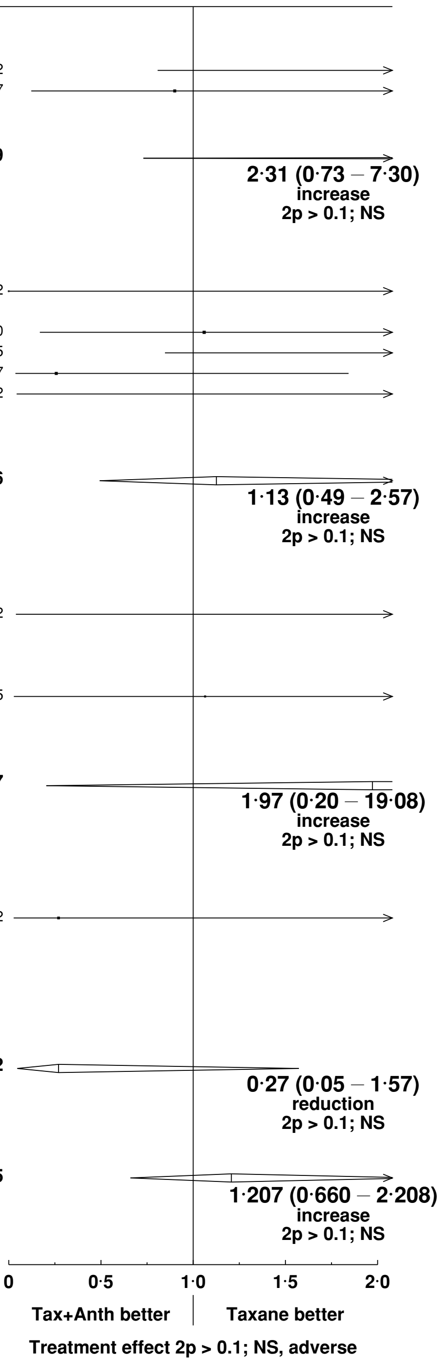

99% or 95% confidence intervals

Heterogeneity between 4 subtotals: $\chi^2_3 = 4{\cdot}2$; p > 0.1; NS

Heterogeneity within subtotals: $\chi^2_6 = 14{\cdot}7$; p = 0·02

Heterogeneity between 10 trials: $\chi^2_9 = 18{\cdot}9$; p = 0·03

* For balance, control patients in 3–way trials or trial strata count half or twice in subtotal(s) and in final total of deaths/patients.

‡ Pre–operative chemotherapy
v.v Vice versa
Taxanes: D = docetaxel; P = paclitaxel
Anthracyclines: A = doxorubicin (Adriamycin); E = epirubicin
Other agents: C = cyclophosphamide; F = fluorouracil; M = methotrexate; Trz = trastuzumab; Vrb = vinorelbine; Cap = capecitabine; Cpt = carboplatin; Ptz = pertuzumab

P12: Death without recurrence in trials of taxane plus anthracycline versus taxane without anthracycline

Year code and study name	Regimens compared and drug doses (mg/m²) per cycle. Any unstated doses are as in the non–anthracycline comparator	Cumulative doses: Taxane	Cumulative doses: Anth	Deaths/Women: Allocated Tax+Anth	Deaths/Women: Adjusted Taxane	Tax+Anth deaths*: Logrank O–E	Tax+Anth deaths*: Variance of O–E
**(a) Concurrent docetaxel plus anthracycline vs same dose docetaxel plus cyclophosphamide**							
07& USO 06090 / 11271	6(A$_{50}$D$_{75}$C$_{500}$ v D$_{75}$C$_{600}$)$_{q3}$	D$_{450}$ vs D$_{450}$	A$_{300}$	23/647	17/649	3·3	9·4
09J NSABP B–46–I	6(A$_{50}$D$_{75}$C$_{500}$ v D$_{75}$C$_{600}$)$_{q3}$	D$_{450}$ vs D$_{450}$	A$_{300}$	12/538	7/539	2·4	4·6
09R NATT Shanghai	6((A$_{50}$/E$_{60}$) D$_{75}$C$_{500}$ v D$_{75}$C$_{600}$)$_{q3}$‡	D$_{450}$ vs D$_{450}$	A$_{300}$/E$_{360}$	0/51	0/45		
■ **(a) subtotal**				**35/ 1236 (2·8%)**	**24/ 1233 (1·9%)**	**5·7**	**14·0**
**(b) Sequential taxane plus anthracycline versus higher cumulative dose docetaxel plus cyclophosphamide**							
08L DBCG 07 READ	(3E$_{90}$C$_{600}$;3D$_{100}$ v 6D$_{75}$C$_{600}$)$_{q3}$	D$_{300}$ vs D$_{450}$	E$_{270}$	26/1001	32/1011	–4·2	14·1
09& Kanagawa Japan	(3FE$_{100}$C$_{500}$;3D$_{100}$ v 6D$_{75}$C$_{600}$)$_{q3}$‡	D$_{300}$ vs D$_{450}$	E$_{300}$	0/53	0/50		
09= NSABP B–49	(4A$_{60}$C$_{q2/q3}$;12P$_{80q1}$/4P$_{175q2}$)/(6A$_{50}$D$_{75}$C$_{500q3}$) v 6D$_{75}$C$_{600q3}$	D$_{450}$/P$_{700–960}$ vs D$_{450}$	A$_{240–300}$	14/932	8/938	3·2	5·4
09E1 LMU SUCCESS C	(3FE$_{100}$C$_{500}$; 3D$_{100}$ v 6D$_{75}$C$_{600}$)$_{q3}$	D$_{300}$ vs D$_{450}$	E$_{300}$	13/1816	7/1827	2·7	4·6
09M WSG Plan B	(4E$_{90}$C$_{600}$;4D$_{100}$ v 6D$_{75}$C$_{600}$)$_{q3}$	D$_{400}$ vs D$_{450}$	E$_{360}$	12/1227	14/1222	–1·1	6·3
09U JBCRG–10 *	(4D$_{75}$C$_{600}$Trz; 4FE$_{100}$C$_{500}$ or v.v v 6D$_{75}$C$_{600}$Trz)$_{q3}$‡	D$_{300}$ vs D$_{450}$	E$_{300}$	2/42	2(0/25)	0·5	0·2
09V JBCRG–09 *	(3D$_{75}$C$_{600}$; 3FE$_{100}$C$_{500}$ or v.v v 6D$_{75}$C$_{600}$)$_{q3}$‡	D$_{225}$ vs D$_{450}$	E$_{300}$	1/128	2(0/67)	0·2	0·2
10V MASTER, Fudan	(3FE$_{100}$C$_{500}$;3D$_{100}$ v 6D$_{75}$C$_{600}$)$_{q3}$	D$_{300}$ vs D$_{450}$	E$_{300}$	1/523	4/524	–1·4	1·2
■ **(b) subtotal**				**69/ 5722 (1·2%)**	**65/ 5756 (1·1%)**	**–0·1**	**32·1**
**(c) Taxane plus anthracycline versus higher taxane ± capecitabine**							
00W N–SAS–BC 02 Japan	(4A$_{60}$C$_{600}$;4P$_{175}$ v 8P$_{175}$)$_{q3}$	P$_{700}$ vs P$_{1400}$	A$_{240}$	1/263	3/267	–1·1	1·0
00W N–SAS–BC 02 Japan	(4A$_{60}$C$_{600}$;4D$_{75}$ v 8D$_{75}$)$_{q3}$	D$_{300}$ vs D$_{600}$	A$_{240}$	5/265	1/265	1·4	1·2
01V Multicentre France	(6E$_{100}$D$_{75}$ v 8D$_{100}$)$_{q3}$	D$_{450}$ vs D$_{800}$	E$_{600}$	*(21 patients)*		*(no data)*	
05] Asan, Seoul	(4A$_{60}$C$_{600}$;4D$_{75}$ v 4Vrb$_{25d1,8}$Cap$_{2000d1–14}$;4D$_{75}$)$_{q3}$‡	D$_{300}$ vs D$_{300}$	A$_{240}$	*(75 patients)*		*(no data)*	
06Z3 MINDACT:EORTC10041	(3FE$_{100}$C$_{500}$;3D$_{100}$ v 6D$_{75}$Cap$_{1650d1–14}$)$_{q3}$	D$_{300}$ vs D$_{450}$	E$_{300}$	2/194	2/198	0·0	1·0
09[2 Remagus 04, France	(4FE$_{100}$C; 4D$_{100}$ v 6D$_{75}$Cap)$_{q3}$‡	D$_{400}$ vs D$_{450}$	E$_{400}$	*(100 patients)*		*(no data)*	
11N PUMCH–Breast–TCX	6((A$_{50}$/E$_{75}$)D$_{75}$C$_{500}$ v D$_{75}$C$_{500}$Cap$_{950d1–14}$)$_{q3}$	D$_{450}$ vs D$_{450}$	A$_{300}$/E$_{450}$	*(400 patients)*		*(no data)*	
■ **(c) subtotal with data §**				**8/ 722 (1·1%)**	**6/ 730 (0·8%)**	**0·3**	**3·2**
**(d) Taxane plus anthracycline versus higher taxane plus carboplatin (confounded)**							
01M BCIRG 006	(4A$_{60}$C$_{600}$;4D$_{100}$ v 6D$_{75}$Cpt$_{AUC6}$)$_{q3}$+12Trz$_{q1}$; Trz$_{q3}$ to 1yr	D$_{400}$ vs D$_{450}$	A$_{240}$	20/1074	25/1075	–3·3	10·8
06~ CAMS NeoPaclitaxel	6(P$_{175}$E$_{75}$ v P$_{175}$Cpt$_{AUC5}$)$_{q3}$	P$_{1050}$ vs P$_{1050}$	E$_{450}$	*(80 patients)*		*(no data)*	
09T TRYPHAENA	6PtzTrz+(3FE$_{100}$C$_{500}$;3D$_{75–100}$ v 6D$_{75}$Cpt$_{AUC6}$)$_{q3}$‡	D$_{225–300}$ vs D$_{450}$	E$_{300}$	*(225 patients)*		*(no data)*	
10U CH–BC–007	(4E$_{90}$C$_{600}$; 4D$_{75}$/P$_{175}$ v 6D$_{75}$/P$_{175}$Cpt$_{AUC5}$)$_{q3}$	D$_{300}$ v D$_{450}$ or P$_{700}$ vs P$_{1050}$	E$_{360}$	*(298 patients)*		*(no data)*	
11M CH–BC–012	(4E$_{80}$C$_{600}$;4P$_{175}$ v 8P$_{150}$Cpt$_{AUC3}$)$_{q2}$	P$_{700}$ vs P$_{1200}$	E$_{360}$	*(132 patients)*		*(no data)*	
11R PATTERN, Fudan	3FE$_{100}$C$_{500}$;3D$_{100q3}$ v 6(P$_{80}$Cpt$_{AUC2}$)$_{d1,8,15q4}$	D$_{300}$ vs P$_{1440}$	E$_{300}$	5/322	5/325	0·4	2·5
■ **(d) subtotal with data §**				**25/ 1396 (1·8%)**	**30/ 1400 (2·1%)**	**–2·9**	**13·3**
■ **Total (a + b + c + d)**				**137/ 9076 (1·5%)**	**125/ 9119 (1·4%)**	**3·1**	**62·6**

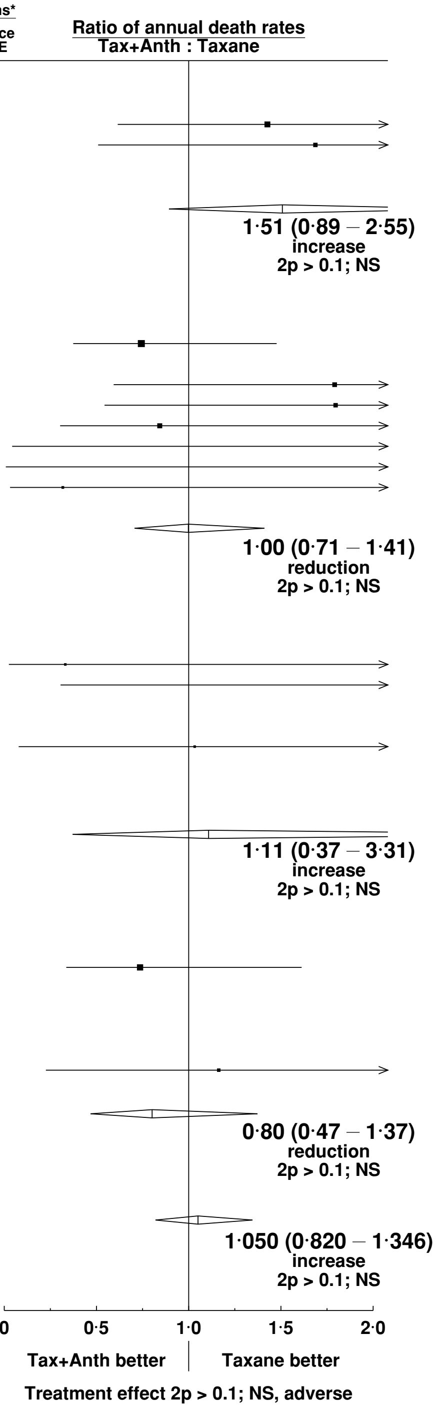

■ 99% or ◇ 95% confidence intervals

Heterogeneity between 4 subtotals: $\chi^2_3 = 2·9$; p > 0.1; NS

Heterogeneity within subtotals: $\chi^2_{10} = 11·1$; p > 0.1; NS

Heterogeneity between 14 trials: $\chi^2_{13} = 14·0$; p > 0.1; NS

* For balance, control patients in 3–way trials or trial strata count half or twice in subtotal(s) and in final total of deaths/patients.

‡ Pre–operative chemotherapy

v.v Vice versa

Taxanes: D = docetaxel; P = paclitaxel

Anthracyclines: A = doxorubicin (Adriamycin); E = epirubicin

Other agents: C = cyclophosphamide; F = fluorouracil; M = methotrexate; Trz = trastuzumab; Vrb = vinorelbine; Cap = capecitabine; Cpt = carboplatin; Ptz = pertuzumab

P13: All-cause mortality in trials of taxane plus anthracycline versus taxane without anthracycline

Year code and study name		Regimens compared and drug doses (mg/m²) per cycle. Any unstated doses are as in the non–anthracycline comparator	Cumulative doses Taxane	Anth	Deaths/Women Allocated Tax+Anth	Deaths/Women Adjusted Taxane	Tax+Anth deaths* Logrank O–E	Tax+Anth deaths* Variance of O–E	Ratio of annual death rates Tax+Anth : Taxane
**(a) Concurrent docetaxel plus anthracycline vs same dose docetaxel plus cyclophosphamide**									
07&	USO 06090 / 11271	$6(A_{50}D_{75}C_{500}\ v\ D_{75}C_{600})_{q3}$	D_{450} vs D_{450}	A_{300}	71/647	89/649	−6·2	38·2	
09J	NSABP B−46−I	$6(A_{50}D_{75}C_{500}\ v\ D_{75}C_{600})_{q3}$	D_{450} vs D_{450}	A_{300}	37/538	37/539	0·2	17·5	
09R	NATT Shanghai	$6((A_{50}/E_{60})\ D_{75}C_{500}\ v\ D_{75}C_{600})_{q3}$‡	D_{450} vs D_{450}	A_{300}/E_{360}	5/51	18/45	−8·3	4·8	
■	**(a) subtotal**				**113/ 1236 (9·1%)**	**144/ 1233 (11·7%)**	**−14·3**	**60·5**	**0·79 (0·61 – 1·02) reduction 2p = 0·07**
**(b) Sequential taxane plus anthracycline versus higher cumulative dose docetaxel plus cyclophosphamide**									
08L	DBCG 07 READ	$(3E_{90}C_{600};3D_{100}\ v\ 6D_{75}C_{600})_{q3}$	D_{300} vs D_{450}	E_{270}	99/1001	111/1011	−10·1	50·2	
09&	Kanagawa Japan	$(3FE_{100}C_{500};3D_{100}\ v\ 6D_{75}C_{600})_{q3}$‡	D_{300} vs D_{450}	E_{300}	4/53	8/50	−2·2	2·9	
09=	NSABP B−49	$(4A_{60}C_{q2/q3};12P_{80q1}/4P_{175q2})/(6A_{50}D_{75}C_{500q3})\ v\ 6D_{75}C_{600q3}$	$D_{450}/P_{700-960}$ vs D_{450}	$A_{240-300}$	46/932	40/938	2·5	20·8	
$09E_1$	LMU SUCCESS C	$(3FE_{100}C_{500};\ 3D_{100}\ v\ 6D_{75}C_{600})_{q3}$	D_{300} vs D_{450}	E_{300}	88/1816	91/1827	−2·9	42·7	
09M	WSG Plan B	$(4E_{90}C_{600};4D_{100}\ v\ 6D_{75}C_{600})_{q3}$	D_{400} vs D_{450}	E_{360}	61/1227	58/1222	1·2	28·3	
09U	JBCRG−10 *	$(4D_{75}C_{600}Trz;\ 4FE_{100}C_{500}$ or v.v v $6D_{75}C_{600}Trz)_{q3}$‡	D_{300} vs D_{450}	E_{300}	3/42	2(1/25)	0·3	0·7	
09V	JBCRG−09 *	$(3D_{75}C_{600};\ 3FE_{100}C_{500}$ or v.v v $6D_{75}C_{600})_{q3}$‡	D_{225} vs D_{450}	E_{300}	7/128	2(4/67)	−0·2	2·4	
10V	MASTER, Fudan	$(3FE_{100}C_{500};3D_{100}\ v\ 6D_{75}C_{600})_{q3}$	D_{300} vs D_{450}	E_{300}	24/523	21/524	0·5	10·8	
■	**(b) subtotal**				**332/ 5722 (5·8%)**	**339/ 5756 (5·9%)**	**−10·9**	**158·8**	**0·93 (0·80 – 1·09) reduction 2p > 0.1; NS**
**(c) Taxane plus anthracycline versus higher taxane ± capecitabine**									
00W	N−SAS−BC 02 Japan	$(4A_{60}C_{600};4P_{175}\ v\ 8P_{175})_{q3}$	P_{700} vs P_{1400}	A_{240}	50/263	51/267	−0·9	24·2	
00W	N−SAS−BC 02 Japan	$(4A_{60}C_{600};4D_{75}\ v\ 8D_{75})_{q3}$	D_{300} vs D_{600}	A_{240}	42/265	37/265	2·4	18·9	
01V	Multicentre France	$(6E_{100}D_{75}\ v\ 8D_{100})_{q3}$	D_{450} vs D_{800}	E_{600}	*(21 patients)*		*(no data)*		
05]	Asan, Seoul	$(4A_{60}C_{600};4D_{75}\ v\ 4Vrb_{25d1,8}Cap_{2000d1-14};4D_{75})_{q3}$‡	D_{300} vs D_{300}	A_{240}	*(75 patients)*		*(no data)*		
$06Z_3$	MINDACT:EORTC10041	$(3FE_{100}C_{500};3D_{100}\ v\ 6D_{75}Cap_{1650d1-14})_{q3}$	D_{300} vs D_{450}	E_{300}	4/194	8/198	−2·2	2·9	
$09[_2$	Remagus 04, France	$(4FE_{100}C;\ 4D_{100}\ v\ 6D_{75}Cap)_{q3}$‡	D_{400} vs D_{450}	E_{400}	*(100 patients)*		*(no data)*		
11N	PUMCH−Breast−TCX	$6((A_{50}/E_{75})D_{75}C_{500}\ v\ D_{75}C_{500}Cap_{950d1-14})_{q3}$	D_{450} vs D_{450}	A_{300}/E_{450}	*(400 patients)*		*(no data)*		
■	**(c) subtotal with data §**				**96/ 722 (13·3%)**	**96/ 730 (13·2%)**	**−0·6**	**45·9**	**0·99 (0·74 – 1·32) reduction 2p > 0.1; NS**
**(d) Taxane plus anthracycline versus higher taxane plus carboplatin (confounded)**									
01M	BCIRG 006	$(4A_{60}C_{600};4D_{100}\ v\ 6D_{75}Cpt_{AUC6})_{q3}+12Trz_{q1};\ Trz_{q3}$ to 1yr	D_{400} vs D_{450}	A_{240}	141/1074	167/1075	−14·1	74·1	
06~	CAMS NeoPaclitaxel	$6(P_{175}E_{75}\ v\ P_{175}Cpt_{AUC5})_{q3}$	P_{1050} vs P_{1050}	E_{450}	*(80 patients)*		*(no data)*		
09T	TRYPHAENA	$6PtzTrz+(3FE_{100}C_{500};3D_{75-100}\ v\ 6D_{75}Cpt_{AUC6})_{q3}$‡	$D_{225-300}$ vs D_{450}	E_{300}	*(225 patients)*		*(no data)*		
10U	CH−BC−007	$(4E_{90}C_{600};\ 4D_{75}/P_{175}\ v\ 6D_{75}/P_{175}Cpt_{AUC5})_{q3}$	D_{300} v D_{450} or P_{700} vs P_{1050}	E_{360}	*(298 patients)*		*(no data)*		
11M	CH−BC−012	$(4E_{80}C_{600};4P_{175}\ v\ 8P_{150}Cpt_{AUC3})_{q2}$	P_{700} vs P_{1200}	E_{360}	*(132 patients)*		*(no data)*		
11R	PATTERN, Fudan	$3FE_{100}C_{500};3D_{100q3}\ v\ 6(P_{80}Cpt_{AUC2})_{d1,8,15q4}$	D_{300} vs P_{1440}	E_{300}	33/322	24/325	5·3	13·8	
■	**(d) subtotal with data §**				**174/ 1396 (12·5%)**	**191/ 1400 (13·6%)**	**−8·8**	**87·9**	**0·90 (0·73 – 1·11) reduction 2p > 0.1; NS**
■	**Total (a + b + c + d)**				**715/ 9076 (7·9%)**	**770/ 9119 (8·4%)**	**−34·6**	**353·0**	**0·907 (0·817 – 1·006) reduction 2p = 0·07**

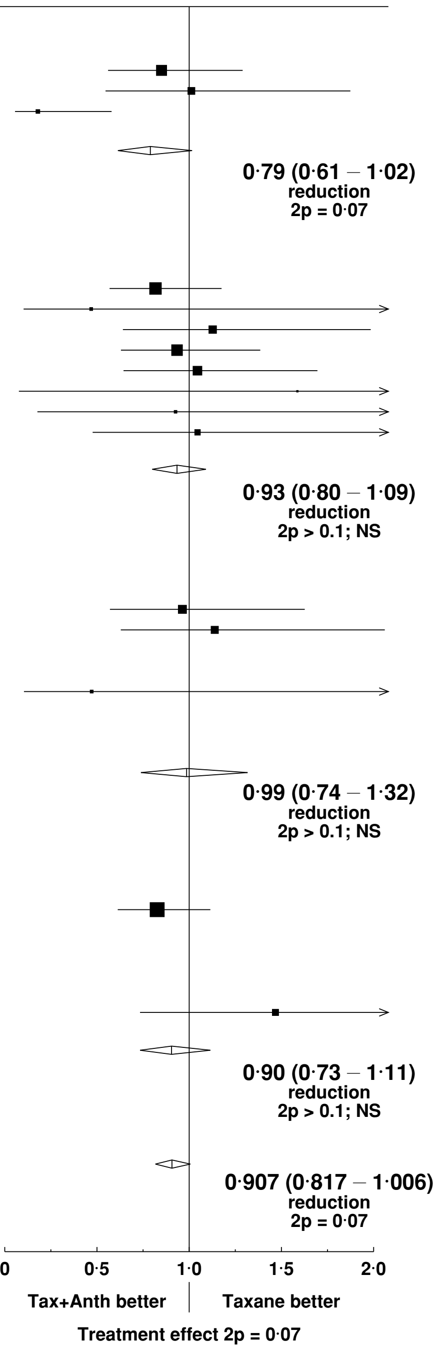

■ 99% or ◇ 95% confidence intervals

Heterogeneity between 4 subtotals: $\chi^2_3 = 1·6$; p > 0.1; NS

Heterogeneity within subtotals: $\chi^2_{12} = 21·4$; p = 0·04

Heterogeneity between 16 trials: $\chi^2_{15} = 23·0$; p = 0·08

* For balance, control patients in 3–way trials or trial strata count half or twice in subtotal(s) and in final total of deaths/patients.

‡ Pre-operative chemotherapy

v.v Vice versa

Taxanes: D = docetaxel; P = paclitaxel

Anthracyclines: A = doxorubicin (Adriamycin); E = epirubicin

Other agents: C = cyclophosphamide; F = fluorouracil; M = methotrexate; Trz = trastuzumab; Vrb = vinorelbine; Cap = capecitabine; Cpt = carboplatin; Ptz = pertuzumab

P14: Subgroup analyses of concurrent anthracycline plus docetaxel plus cyclophosphamide versus same dose docetaxel plus cyclophosphamide; distant recurrence at any time

Category	Events/Women: Allocated Tax+Anth	Events/Women: Allocated Taxane	Tax+Anth events*: Logrank O−E	Tax+Anth events*: Variance of O−E	Ratio (& CI)
**(a) Follow up period, years (trend χ^2_1 = 0.4; 2p > 0.1; NS)**					
0–1	34/2282 (1·5%)	57/2322 (2·5%)	−12·6	21·0	0·55 (0·31 − 0·96)
2–4	45/2988 (1·5%)	71/3038 (2·3%)	−11·9	27·3	0·65 (0·39 − 1·06)
5–9	17/2618 (0·6%)	42/2693 (1·6%)	−12·1	14·4	0·43 (0·22 − 0·85)
10+	0/11 (0·0%)	1/19 (5·3%)	−0·4	0·2	
**(b) Age at entry (trend χ^2_1 = 2.5; 2p > 0.1; NS)**					
Age < 45	17/261 (6·5%)	37/247 (15·0%)	−10·9	12·3	0·41 (0·20 − 0·86)
45 – 54	36/466 (7·7%)	57/423 (13·5%)	−14·9	21·8	0·51 (0·29 − 0·88)
55 – 64	34/391 (8·7%)	61/424 (14·4%)	−9·9	22·6	0·65 (0·38 − 1·11)
65+	9/118 (7·6%)	16/139 (11·5%)	−1·2	5·4	
**(c) ER status (χ^2_1 = 0.9; 2p > 0.1: NS)**					
ER−poor	38/417 (9·1%)	74/419 (17·7%)	−19·1	26·5	0·49 (0·29 − 0·80)
ER+	58/819 (7·1%)	97/814 (11·9%)	−18·2	38·1	0·62 (0·41 − 0·94)
**(d) Nodal status (trend χ^2_1 = 1.7; 2p > 0.1; NS)**					
N0/N−	22/432 (5·1%)	50/453 (11·0%)	−13·3	17·3	0·46 (0·25 − 0·86)
N1−3	31/574 (5·4%)	51/545 (9·4%)	−10·1	19·9	0·60 (0·34 − 1·07)
N4+	36/191 (18·8%)	51/194 (26·3%)	−7·0	20·5	0·71 (0·40 − 1·26)
N unknown	7/39 (17·9%)	19/41 (46·3%)	−6·6	5·2	0·28 (0·09 − 0·87)
**(e) ER & nodal status (χ^2_3 = 2.9; 2p > 0.1: NS)**					
ER−poor/N0	15/263 (5·7%)	29/278 (10·4%)	−7·3	10·3	0·49 (0·22 − 1·10)
ER−poor/N+	23/154 (14·9%)	45/141 (31·9%)	−13·0	15·6	0·44 (0·23 − 0·84)
ER+/N0	7/169 (4·1%)	21/175 (12·0%)	−6·0	6·9	0·42 (0·16 − 1·12)
ER+/N+	51/650 (7·8%)	76/639 (11·9%)	−12·0	31·2	0·68 (0·43 − 1·08)
**(f) HER2 status (χ^2_1 = 0.1; 2p > 0.1: NS)**					
HER2−	93/1211 (7·7%)	165/1208 (13·7%)	−35·3	60·2	0·56 (0·40 − 0·77)
HER2+	3/25 (12·0%)	6/25 (24·0%)	−1·5	1·7	
**(g) Tumour diameter (trend χ^2_1 = 2.1; 2p > 0.1; NS)**					
1 – 20 mm (T1)	22/454 (4·8%)	27/451 (6·0%)	−2·6	11·9	
21 – 50 mm (T2)	56/649 (8·6%)	108/668 (16·2%)	−23·9	39·2	0·54 (0·36 − 0·82)
> 50 mm (T3/T4)	9/82 (11·0%)	15/69 (21·7%)	−3·9	4·1	
Other / unknown	9/51 (17·6%)	21/45 (46·7%)	−7·7	6·4	0·30 (0·11 − 0·82)
**(h) Tumour grade (trend χ^2_1 = 0.1; 2p > 0.1; NS)**					
Well−differentiated	6/137 (4·4%)	8/123 (6·5%)	−2·1	3·1	
Moderately	29/469 (6·2%)	45/449 (10·0%)	−8·0	17·7	0·64 (0·35 − 1·18)
Poorly	56/565 (9·9%)	112/614 (18·2%)	−24·6	39·8	0·54 (0·36 − 0·81)
Grade unknown	5/65 (7·7%)	6/47 (12·8%)	−0·8	2·6	
**(i) Histological type (χ^2_1 = 0.0; 2p > 0.1; NS)**					
Ductal	43/563 (7·6%)	95/576 (16·5%)	−23·9	32·2	0·48 (0·30 − 0·75)
Lobular	9/67 (13·4%)	11/53 (20·8%)	−3·4	3·9	
Unknown	44/606 (7·3%)	65/604 (10·8%)	−9·2	24·1	0·68 (0·40 − 1·15)
**(j) BMI (trend χ^2_1 = 2.2; 2p > 0.1; NS)**					
<20	2/46 (4·3%)	9/46 (19·6%)	−0·6	0·7	
20 – <25	28/309 (9·1%)	48/276 (17·4%)	−11·6	15·7	0·48 (0·25 − 0·91)
25 – <30	25/355 (7·0%)	51/366 (13·9%)	−12·8	17·3	0·48 (0·26 − 0·89)
30+	41/525 (7·8%)	63/544 (11·6%)	−6·8	23·2	0·75 (0·44 − 1·27)
**■ Total**	**96/1236 (7·8%)**	**171/1233 (13·9%)**	**−37·0**	**62·9**	**0·555 (0·434 − 0·711) 2p < 0·00001**

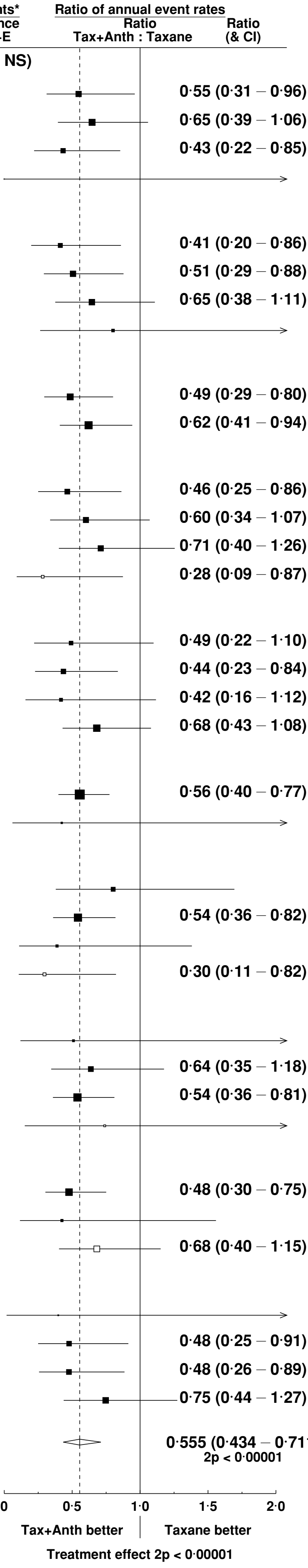

■ 99% or ◇ 95% confidence intervals

P15: Subgroup analyses of concurrent anthracycline plus docetaxel plus cyclophosphamide versus same dose docetaxel plus cyclophosphamide; breast cancer mortality

Category	Events/Women Allocated Tax+Anth	Events/Women Allocated Taxane	Tax+Anth events* Logrank O−E	Tax+Anth events* Variance of O−E	Ratio (& CI)
**(a) Follow up period, years** (trend χ^2_1 = 0.1; 2p > 0.1; NS)					
**0−1**	15/2304 (0·7%)	22/2346 (0·9%)	−3·9	8·5	
**2−4**	40/3051 (1·3%)	64/3154 (2·0%)	−11·8	24·3	0·62 (0·37 − 1·04)
**5−9**	23/2665 (0·9%)	34/2797 (1·2%)	−4·4	13·6	
**10+**	0/11 (0·0%)	0/20 (0·0%)	−0·0	0·0	
**(b) Age at entry** (trend χ^2_1 = 1.0; 2p > 0.1; NS)					
**Age < 45**	14/261 (5·4%)	23/247 (9·3%)	−5·0	8·5	
**45 − 54**	28/466 (6·0%)	42/423 (9·9%)	−10·3	16·4	0·53 (0·28 − 1·01)
**55 − 64**	30/391 (7·7%)	44/424 (10·4%)	−3·4	17·4	
**65+**	6/118 (5·1%)	11/139 (7·9%)	−1·4	3·7	
**(c) ER status** (χ^2_1 = 1.1; 2p > 0.1: NS)					
**ER−poor**	34/417 (8·2%)	58/419 (13·8%)	−12·8	21·8	0·56 (0·32 − 0·97)
**ER+**	44/819 (5·4%)	62/814 (7·6%)	−7·3	26·0	0·76 (0·46 − 1·25)
**(d) Nodal status** (trend χ^2_1 = 2.4; 2p > 0.1; NS)					
**N0/N−**	20/432 (4·6%)	33/453 (7·3%)	−5·9	12·5	0·62 (0·30 − 1·29)
**N1−3**	23/574 (4·0%)	39/545 (7·2%)	−7·6	15·1	0·60 (0·31 − 1·17)
**N4+**	32/191 (16·8%)	31/194 (16·0%)	1·5	14·5	
**N unknown**	3/39 (7·7%)	17/41 (41·5%)	−8·0	4·3	0·16 (0·04 − 0·54)
**(e) ER & nodal status** (χ^2_3 = 3.1; 2p > 0.1: NS)					
**ER−poor/N0**	14/263 (5·3%)	19/278 (6·8%)	−3·0	7·6	
**ER−poor/N+**	20/154 (13·0%)	39/141 (27·7%)	−10·7	13·6	0·46 (0·23 − 0·92)
**ER+/N0**	6/169 (3·6%)	14/175 (8·0%)	−3·1	4·8	
**ER+/N+**	38/650 (5·8%)	48/639 (7·5%)	−4·0	21·0	
**(f) HER2 status** (χ^2_1 = 0.4; 2p > 0.1: NS)					
**HER2−**	76/1211 (6·3%)	117/1208 (9·7%)	−18·9	44·5	0·65 (0·44 − 0·96)
**HER2+**	2/25 (8·0%)	3/25 (12·0%)	−1·1	1·1	
**(g) Tumour diameter** (trend χ^2_1 = 1.0; 2p > 0.1; NS)					
**1 − 20 mm (T1)**	18/454 (4·0%)	19/451 (4·2%)	−0·7	9·0	
**21 − 50 mm (T2)**	47/649 (7·2%)	73/668 (10·9%)	−9·5	28·3	0·72 (0·44 − 1·16)
**> 50 mm (T3/T4)**	8/82 (9·8%)	10/69 (14·5%)	−2·1	2·8	
**Other / unknown**	5/51 (9·8%)	18/45 (40·0%)	−8·4	4·9	0·18 (0·06 − 0·57)
**(h) Tumour grade** (trend χ^2_1 = 0.0; 2p > 0.1; NS)					
**Well−differentiated**	3/137 (2·2%)	4/123 (3·3%)	−0·7	1·7	
**Moderately**	21/469 (4·5%)	29/449 (6·5%)	−3·5	12·0	
**Poorly**	51/565 (9·0%)	81/614 (13·2%)	−11·7	31·3	0·69 (0·43 − 1·09)
**Grade unknown**	3/65 (4·6%)	6/47 (12·8%)	−1·9	2·1	
**(i) Histological type** (χ^2_1 = 0.0; 2p > 0.1; NS)					
**Ductal**	38/563 (6·7%)	75/576 (13·0%)	−17·3	26·6	0·52 (0·32 − 0·86)
**Lobular**	8/67 (11·9%)	9/53 (17·0%)	−2·4	3·3	
**Unknown**	32/606 (5·3%)	36/604 (6·0%)	0·3	15·3	
**(j) BMI** (trend χ^2_1 = 2.2; 2p > 0.1; NS)					
**<20**	2/46 (4·3%)	7/46 (15·2%)	−1·3	0·4	
**20 − <25**	18/309 (5·8%)	37/276 (13·4%)	−10·6	11·5	0·40 (0·18 − 0·85)
**25 − <30**	21/355 (5·9%)	36/366 (9·8%)	−6·8	12·9	0·59 (0·29 − 1·20)
**30+**	37/525 (7·0%)	40/544 (7·4%)	0·9	16·8	
**Total**	**78/1236 (6·3%)**	**120/1233 (9·7%)**	**−20·0**	**46·5**	**0·650 (0·487 − 0·866)** 2p = 0·003

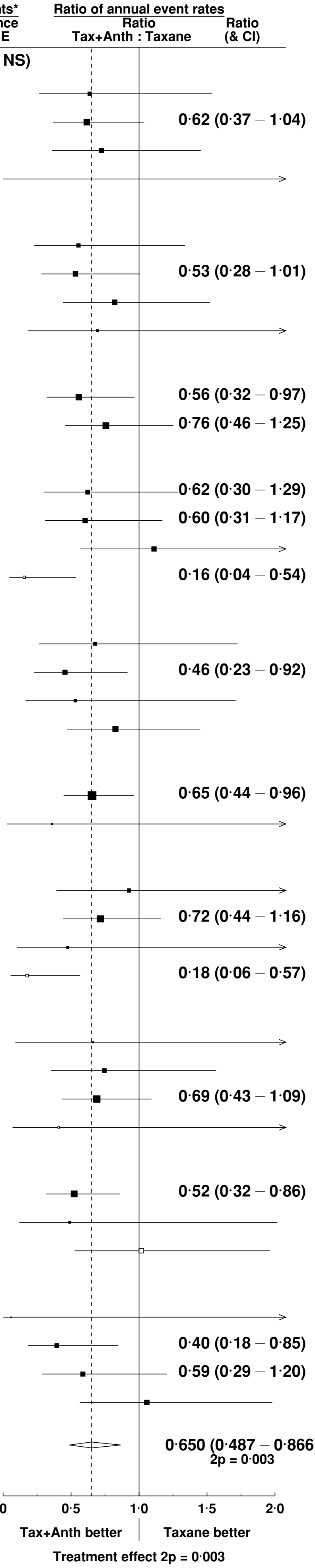

99% or 95% confidence intervals

P16: Subgroup analyses of taxane plus anthracycline versus taxane without anthracycline; any recurrence. All trial comparisons (a)-(d).

Category	Events/Women Allocated Tax+Anth	Events/Women Allocated Taxane	Tax+Anth events Logrank O–E	Tax+Anth events Variance of O–E	Ratio (& CI)
**(a) Trial groupings** (χ^2_3 = 16.5; p = 0.0009)					
6DC ± Conc Anth	123/1236 (10·0%)	205/1233 (16·6%)	−40·4	75·2	0·58 (0·43 − 0·79)
Anth→ 3/4Tax v 6DC	510/5722 (8·9%)	535/5756 (9·3%)	−16·3	244·3	0·94 (0·79 − 1·10)
Anth→ 3/4Tax v 6/8Tax	181/722 (25·1%)	222/730 (30·4%)	−24·4	92·4	0·77 (0·59 − 1·00)
Tax+Anth v Tax+CPlat	248/1396 (17·8%)	248/1400 (17·7%)	−0·9	116·7	0·99 (0·78 − 1·26)
**(b) Site of first recurrence** (χ^2_2 = 1.1; p > 0.1: NS)					
Distant	806/9076 (8·9%)	936/9119 (10·3%)	−70·9	410·5	0·84 (0·74 − 0·96)
Isolated local	214/9076 (2·4%)	236/9119 (2·6%)	−12·2	106·8	0·89 (0·70 − 1·15)
Contralateral	42/9076 (0·5%)	38/9119 (0·4%)	1·0	19·5	
**(c) Follow up period, years** (trend χ^2_1 = 0.7; 2p > 0.1; NS)					
0–1	376/17013 (2·2%)	466/17121 (2·7%)	−47·6	192·0	0·78 (0·65 − 0·94)
2–4	524/21278 (2·5%)	551/21435 (2·6%)	−16·2	251·8	0·94 (0·80 − 1·10)
5–9	158/11475 (1·4%)	190/11325 (1·7%)	−19·1	83·3	0·80 (0·60 − 1·05)
10+	4/392 (1·0%)	3/371 (0·8%)	0·9	1·5	
**(d) Age at entry** (trend χ^2_1 = 0.2; 2p > 0.1; NS)					
Age < 45	269/1862 (14·4%)	306/1848 (16·6%)	−16·8	129·9	0·88 (0·70 − 1·10)
45 – 54	372/3256 (11·4%)	406/3358 (12·1%)	−17·7	183·9	0·91 (0·75 − 1·10)
55 – 64	296/2801 (10·6%)	360/2756 (13·1%)	−37·1	152·2	0·78 (0·64 − 0·97)
65+	125/1157 (10·8%)	138/1157 (11·9%)	−5·4	58·9	0·91 (0·65 − 1·28)
**(e) ER status** (χ^2_1 = 0.0; 2p > 0.1: NS)					
ER−	496/2895 (17·1%)	550/2854 (19·3%)	−34·2	245·6	0·87 (0·74 − 1·03)
ER?	9/108 (8·3%)	11/94 (11·7%)	−1·1	4·6	
ER+	557/6073 (9·2%)	649/6171 (10·5%)	−43·5	290·1	0·86 (0·74 − 1·00)
**(f) Nodal status** (trend χ^2_1 = 0.0; 2p > 0.1; NS)					
N0/N−	267/3633 (7·3%)	306/3699 (8·3%)	−16·8	136·8	0·88 (0·71 − 1·10)
N1–3	451/3760 (12·0%)	527/3739 (14·1%)	−46·5	229·9	0·82 (0·69 − 0·97)
N4+	258/1121 (23·0%)	286/1111 (25·7%)	−17·9	124·5	0·87 (0·69 − 1·09)
N unknown	86/562 (15·3%)	91/570 (16·0%)	−0·9	37·4	
**(g) ER & nodal status** (χ^2_3 = 0.4; 2p > 0.1: NS)					
ER−poor/N0	148/1590 (9·3%)	177/1647 (10·7%)	−12·5	78·7	0·85 (0·64 − 1·14)
ER−poor/N+	343/1255 (27·3%)	363/1161 (31·3%)	−24·8	159·9	0·86 (0·70 − 1·05)
ER+/N0	115/1979 (5·8%)	127/2006 (6·3%)	−4·1	57·4	
ER+/N+	442/4091 (10·8%)	521/4160 (12·5%)	−37·7	231·8	0·85 (0·72 − 1·01)
**(h) HER2 status** (χ^2_3 = 0.5; 2p > 0.1: NS)					
HER2−*	124/1126 (11·0%)	143/1141 (12·5%)	−12·6	60·6	0·81 (0·58 − 1·13)
HER2+*	27/173 (15·6%)	32/163 (19·6%)	−1·9	10·9	
HER2 unknown*	170/528 (32·2%)	207/532 (38·9%)	−22·2	86·2	0·77 (0·59 − 1·02)
HER2− eligibility	540/6133 (8·8%)	611/6158 (9·9%)	−32·1	270·4	0·89 (0·76 − 1·04)
HER2+ eligibility	201/1116 (18·0%)	217/1125 (19·3%)	−10·5	97·9	0·90 (0·69 − 1·17)
**(i) Tumour diameter** (trend χ^2_1 = 1.4; 2p > 0.1; NS)					
1 – 20 mm (T1)	331/4106 (8·1%)	337/4038 (8·3%)	−7·2	159·9	0·96 (0·78 − 1·17)
21 – 50 mm (T2)	613/4411 (13·9%)	731/4555 (16·0%)	−56·7	312·7	0·83 (0·72 − 0·96)
> 50 mm (T3/T4)	97/450 (21·6%)	104/427 (24·4%)	−5·7	37·5	
Other / unknown	21/109 (19·3%)	38/99 (38·4%)	−12·0	11·6	0·36 (0·17 − 0·76)
**(j) Tumour grade** (trend χ^2_1 = 0.1; 2p > 0.1; NS)					
Well−differentiated	28/611 (4·6%)	32/608 (5·3%)	−3·2	13·9	
Moderately	295/3710 (8·0%)	321/3754 (8·6%)	−15·4	147·7	0·90 (0·73 − 1·11)
Poorly	536/3906 (13·7%)	598/3933 (15·2%)	−25·4	268·9	0·91 (0·78 − 1·06)
Grade unknown	203/849 (23·9%)	259/824 (31·4%)	−32·4	106·1	0·74 (0·57 − 0·95)
**(k) Histological type** (χ^2_1 = 0.4; 2p > 0.1: NS)					
Ductal	744/5790 (12·8%)	874/5831 (15·0%)	−70·7	373·8	0·83 (0·72 − 0·95)
Lobular	60/594 (10·1%)	70/600 (11·7%)	−8·1	24·7	0·72 (0·43 − 1·21)
Unknown	258/2692 (9·6%)	266/2688 (9·9%)	−1·6	118·2	0·99 (0·78 − 1·25)
**(l) BMI** (trend χ^2_1 = 0.1; 2p > 0.1; NS)					
<20	86/507 (17·0%)	102/524 (19·5%)	−5·7	32·2	
20 − <25	366/2971 (12·3%)	460/3040 (15·1%)	−43·2	186·0	0·79 (0·66 − 0·96)
25 − <30	284/2383 (11·9%)	315/2332 (13·5%)	−27·2	134·5	0·82 (0·65 − 1·02)
30+	217/2151 (10·1%)	247/2159 (11·4%)	−18·0	99·8	0·84 (0·65 − 1·08)
Unknown	109/1064 (10·2%)	86/1064 (8·1%)	11·6	45·9	
**(m) Ki−67** (trend χ^2_1 = 1.2; 2p > 0.1; NS)					
<10%	9/363 (2·5%)	21/380 (5·5%)	−3·2	6·0	
10 − <20%	29/629 (4·6%)	29/652 (4·4%)	2·3	12·4	
20 − <50%	97/840 (11·5%)	103/812 (12·7%)	−8·4	44·4	0·83 (0·56 − 1·22)
50+%	71/366 (19·4%)	71/369 (19·2%)	4·3	29·0	
Unknown	856/6878 (12·4%)	986/6906 (14·3%)	−72·2	427·1	0·84 (0·75 − 0·96)
**Total**	**1062/ 9076 (11·7%)**	**1210/ 9119 (13·3%)**	**−82·0**	**528·7**	**0·856 (0·786 − 0·932)** 2p = 0·0004

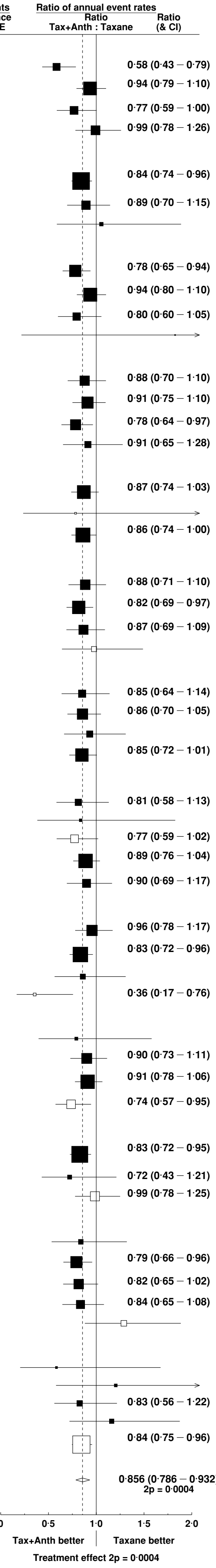

■ 99% or ◇ 95% confidence intervals

*Trials which randomised both HER2 negative and positive tumours shown separately to those trials which had HER2 negative only or positive only eligibility criteria.

P17: Subgroup analyses of taxane plus anthracycline versus taxane without anthracycline; distant recurrence at any time. All trial comparisons (a)-(d).

Category	Events/Women Allocated Tax+Anth	Events/Women Allocated Taxane	Tax+Anth events Logrank O−E	Tax+Anth events Variance of O−E	Ratio (& CI)
**(a) Trial groupings** (χ^2_3 = 13.7; p = 0.003)					
**6DC ± Conc Anth**	96/1236 (7·8%)	171/1233 (13·9%)	−37·0	62·9	**0·56 (0·40 − 0·77)**
**Anth→ 3/4Tax v 6DC**	415/5722 (7·3%)	449/5756 (7·8%)	−20·0	203·1	**0·91 (0·76 − 1·09)**
**Anth→ 3/4Tax v 6/8Tax**	156/722 (21·6%)	189/730 (25·9%)	−19·1	80·3	**0·79 (0·59 − 1·05)**
**Tax+Anth v Tax+CPlat**	197/1396 (14·1%)	206/1400 (14·7%)	−4·8	96·0	**0·95 (0·73 − 1·24)**
**(b) Follow up period, years** (trend χ^2_1 = 0.2; 2p > 0.1; NS)					
**0–1**	308/17071 (1·8%)	369/17207 (2·1%)	−32·9	156·9	**0·81 (0·66 − 1·00)**
**2–4**	424/21579 (2·0%)	469/21760 (2·2%)	−23·9	211·5	**0·89 (0·75 − 1·07)**
**5–9**	131/11790 (1·1%)	174/11655 (1·5%)	−23·2	72·9	**0·73 (0·54 − 0·98)**
**10+**	1/409 (0·2%)	3/384 (0·8%)	−0·9	1·0	
**(c) Age at entry** (trend χ^2_1 = 0.0; 2p > 0.1; NS)					
**Age < 45**	205/1862 (11·0%)	248/1848 (13·4%)	−20·9	104·8	**0·82 (0·64 − 1·05)**
**45 − 54**	310/3256 (9·5%)	340/3358 (10·1%)	−14·9	154·7	**0·91 (0·74 − 1·12)**
**55 − 64**	241/2801 (8·6%)	305/2756 (11·1%)	−36·5	127·8	**0·75 (0·60 − 0·94)**
**65+**	108/1157 (9·3%)	122/1157 (10·5%)	−3·7	51·9	
**(d) ER status** (χ^2_1 = 0.2; 2p > 0.1: NS)					
**ER−**	377/2895 (13·0%)	447/2854 (15·7%)	−39·8	195·7	**0·82 (0·68 − 0·98)**
**ER?**	8/108 (7·4%)	5/94 (5·3%)	1·2	3·2	
**ER+**	479/6073 (7·9%)	563/6171 (9·1%)	−39·8	252·4	**0·85 (0·73 − 1·00)**
**(e) Nodal status** (trend χ^2_1 = 0.2; 2p > 0.1; NS)					
**N0/N−**	181/3633 (5·0%)	229/3699 (6·2%)	−20·9	99·1	**0·81 (0·63 − 1·05)**
**N1−3**	379/3760 (10·1%)	445/3739 (11·9%)	−38·1	195·4	**0·82 (0·68 − 0·99)**
**N4+**	236/1121 (21·1%)	265/1111 (23·9%)	−18·0	116·2	**0·86 (0·67 − 1·09)**
**N unknown**	68/562 (12·1%)	76/570 (13·3%)	−3·9	31·6	
**(f) ER & nodal status** (χ^2_3 = 0.8; 2p > 0.1: NS)					
**ER−poor/N0**	97/1590 (6·1%)	129/1647 (7·8%)	−15·1	54·7	**0·76 (0·54 − 1·07)**
**ER−poor/N+**	276/1255 (22·0%)	308/1161 (26·5%)	−27·3	134·9	**0·82 (0·65 − 1·02)**
**ER+/N0**	81/1979 (4·1%)	99/2006 (4·9%)	−6·6	43·2	**0·86 (0·58 − 1·27)**
**ER+/N+**	398/4091 (9·7%)	463/4160 (11·1%)	−30·7	208·4	**0·86 (0·72 − 1·03)**
**(g) HER2 status** (χ^2_3 = 0.8; 2p > 0.1: NS)					
**HER2−***	96/1126 (8·5%)	118/1141 (10·3%)	−13·5	49·1	**0·76 (0·53 − 1·10)**
**HER2+***	17/173 (9·8%)	25/163 (15·3%)	−1·8	8·4	
**HER2 unknown***	148/528 (28·0%)	178/532 (33·5%)	−17·7	75·7	**0·79 (0·59 − 1·06)**
**HER2− eligibility**	437/6133 (7·1%)	506/6158 (8·2%)	−32·0	223·1	**0·87 (0·73 − 1·03)**
**HER2+ eligibility**	166/1116 (14·9%)	188/1125 (16·7%)	−12·2	83·9	**0·86 (0·65 − 1·14)**
**(h) Tumour diameter** (trend χ^2_1 = 0.1; 2p > 0.1; NS)					
**1 − 20 mm (T1)**	243/4106 (5·9%)	270/4038 (6·7%)	−15·1	123·5	**0·88 (0·70 − 1·12)**
**21 − 50 mm (T2)**	520/4411 (11·8%)	620/4555 (13·6%)	−49·3	268·5	**0·83 (0·71 − 0·97)**
**> 50 mm (T3/T4)**	87/450 (19·3%)	94/427 (22·0%)	−4·1	34·1	
**Other / unknown**	14/109 (12·8%)	31/99 (31·3%)	−11·5	9·7	**0·31 (0·13 − 0·70)**
**(i) Tumour grade** (trend χ^2_1 = 0.2; 2p > 0.1; NS)					
**Well−differentiated**	21/611 (3·4%)	27/608 (4·4%)	−4·0	11·0	
**Moderately**	248/3710 (6·7%)	280/3754 (7·5%)	−18·2	127·5	**0·87 (0·69 − 1·09)**
**Poorly**	419/3906 (10·7%)	487/3933 (12·4%)	−29·5	216·8	**0·87 (0·73 − 1·04)**
**Grade unknown**	176/849 (20·7%)	221/824 (26·8%)	−26·0	92·6	**0·76 (0·58 − 0·99)**
**(j) Histological type** (χ^2_1 = 0.4; 2p > 0.1: NS)					
**Ductal**	609/5790 (10·5%)	730/5831 (12·5%)	−63·3	313·4	**0·82 (0·71 − 0·95)**
**Lobular**	55/594 (9·3%)	65/600 (10·8%)	−7·8	22·6	**0·71 (0·41 − 1·22)**
**Unknown**	200/2692 (7·4%)	220/2688 (8·2%)	−7·7	95·5	**0·92 (0·71 − 1·20)**
**(k) BMI** (trend χ^2_1 = 0.1; 2p > 0.1; NS)					
**<20**	65/507 (12·8%)	84/524 (16·0%)	−7·0	26·4	**0·77 (0·46 − 1·26)**
**20 − <25**	300/2971 (10·1%)	380/3040 (12·5%)	−35·7	155·8	**0·80 (0·65 − 0·98)**
**25 − <30**	234/2383 (9·8%)	271/2332 (11·6%)	−27·2	114·5	**0·79 (0·62 − 1·00)**
**30+**	186/2151 (8·6%)	214/2159 (9·9%)	−16·3	85·6	**0·83 (0·63 − 1·09)**
**Unknown**	79/1064 (7·4%)	66/1064 (6·2%)	6·4	34·6	
**(l) Ki−67** (trend χ^2_1 = 1.4; 2p > 0.1; NS)					
**<10%**	6/363 (1·7%)	17/380 (4·5%)	−4·0	4·8	
**10 − <20%**	24/629 (3·8%)	23/652 (3·5%)	2·1	10·3	
**20 − <50%**	72/840 (8·6%)	81/812 (10·0%)	−7·9	34·3	**0·80 (0·51 − 1·23)**
**50+%**	55/366 (15·0%)	57/369 (15·4%)	2·3	22·8	
**Unknown**	707/6878 (10·3%)	837/6906 (12·1%)	−69·8	362·6	**0·82 (0·72 − 0·94)**
**Total**	**864/9076 (9·5%)**	**1015/9119 (11·1%)**	**−80·9**	**442·3**	**0·833 (0·759 − 0·914) 2p = 0·0001**

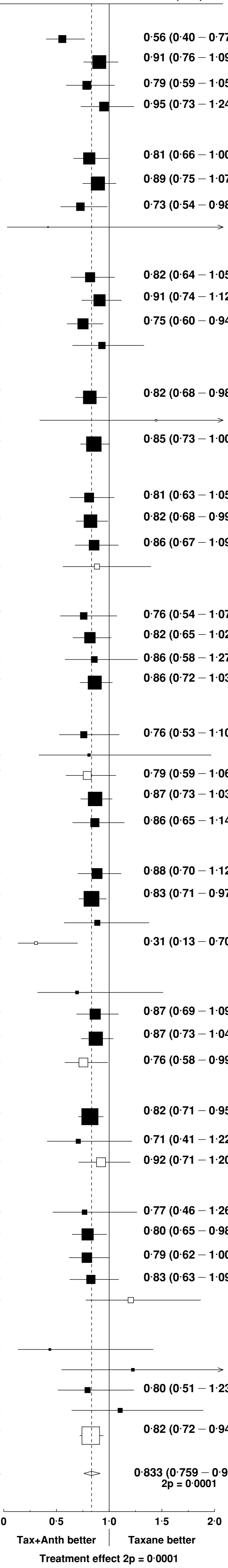

*Trials which randomised both HER2 negative and positive tumours shown separately to those trials which had HER2 negative only or positive only eligibility criteria.

P18: Subgroup analyses of taxane plus anthracycline versus taxane without anthracycline; breast cancer mortality. All trial comparisons (a)-(d).

Category	Events/Women Allocated Tax+Anth	Events/Women Allocated Taxane	Tax+Anth events Logrank O−E	Tax+Anth events Variance of O−E	Ratio (& CI)
**(a) Trial groupings (χ^2_3 = 5.2; p > 0.1: NS)**					
6DC ± Conc Anth	78/1236 (6·3%)	120/1233 (9·7%)	−20·0	46·5	0·65 (0·45 − 0·95)
Anth→ 3/4Tax v 6DC	263/5722 (4·6%)	274/5756 (4·8%)	−10·8	126·6	0·92 (0·73 − 1·15)
Anth→ 3/4Tax v 6/8Tax	88/722 (12·2%)	90/730 (12·3%)	−1·0	42·7	
Tax+Anth v Tax+CPlat	149/1396 (10·7%)	161/1400 (11·5%)	−5·9	74·6	0·92 (0·69 − 1·24)
**(b) Follow up period, years (trend χ^2_1 = 0.1; 2p > 0.1; NS)**					
0–1	105/17253 (0·6%)	129/17410 (0·7%)	−13·9	54·8	0·78 (0·55 − 1·10)
2–4	314/22317 (1·4%)	333/22685 (1·5%)	−10·4	153·9	0·93 (0·76 − 1·15)
5–9	154/12325 (1·2%)	177/12358 (1·4%)	−13·0	79·0	0·85 (0·63 − 1·13)
10+	5/430 (1·2%)	6/410 (1·5%)	−0·4	2·7	
**(c) Age at entry (trend χ^2_1 = 0.0; 2p > 0.1; NS)**					
Age < 45	137/1862 (7·4%)	161/1848 (8·7%)	−11·3	70·9	0·85 (0·63 − 1·16)
45 – 54	185/3256 (5·7%)	205/3358 (6·1%)	−10·6	93·6	0·89 (0·68 − 1·16)
55 – 64	184/2801 (6·6%)	199/2756 (7·2%)	−11·2	89·7	0·88 (0·67 − 1·16)
65+	72/1157 (6·2%)	80/1157 (6·9%)	−3·7	33·4	
**(d) ER status (χ^2_1 = 0.0; 2p > 0.1: NS)**					
ER−	298/2895 (10·3%)	328/2854 (11·5%)	−17·9	150·8	0·89 (0·72 − 1·10)
ER?	6/108 (5·6%)	3/94 (3·2%)	1·3	2·2	
ER+	274/6073 (4·5%)	314/6171 (5·1%)	−16·2	143·0	0·89 (0·72 − 1·11)
**(e) Nodal status (trend χ^2_1 = 0.8; 2p > 0.1; NS)**					
N0/N−	123/3633 (3·4%)	150/3699 (4·1%)	−11·4	65·9	0·84 (0·61 − 1·15)
N1−3	235/3760 (6·2%)	264/3739 (7·1%)	−15·9	120·5	0·88 (0·69 − 1·11)
N4+	177/1121 (15·8%)	175/1111 (15·8%)	−2·4	81·7	0·97 (0·73 − 1·29)
N unknown	43/562 (7·7%)	56/570 (9·8%)	−7·9	22·3	0·70 (0·41 − 1·21)
**(f) ER & nodal status (χ^2_3 = 0.4; 2p > 0.1: NS)**					
ER−poor/N0	77/1590 (4·8%)	95/1647 (5·8%)	−8·8	41·5	0·81 (0·54 − 1·21)
ER−poor/N+	217/1255 (17·3%)	225/1161 (19·4%)	−13·2	104·2	0·88 (0·68 − 1·13)
ER+/N0	43/1979 (2·2%)	54/2006 (2·7%)	−3·7	23·2	
ER+/N+	231/4091 (5·6%)	260/4160 (6·2%)	−11·3	119·4	0·91 (0·72 − 1·15)
**(g) HER2 status (χ^2_3 = 3.2; 2p > 0.1: NS)**					
HER2−*	72/1126 (6·4%)	95/1141 (8·3%)	−14·9	38·0	0·68 (0·45 − 1·03)
HER2+*	12/173 (6·9%)	16/163 (9·8%)	−1·0	5·9	
HER2 unknown*	86/528 (16·3%)	84/532 (15·8%)	1·2	40·8	
HER2− eligibility	286/6133 (4·7%)	306/6158 (5·0%)	−9·3	140·1	0·94 (0·75 − 1·16)
HER2+ eligibility	122/1116 (10·9%)	144/1125 (12·8%)	−11·0	63·7	0·84 (0·61 − 1·16)
**(h) Tumour diameter (trend χ^2_1 = 0.0; 2p > 0.1; NS)**					
1 – 20 mm (T1)	156/4106 (3·8%)	168/4038 (4·2%)	−10·3	78·0	0·88 (0·65 − 1·17)
21 – 50 mm (T2)	352/4411 (8·0%)	386/4555 (8·5%)	−11·7	175·1	0·94 (0·77 − 1·14)
> 50 mm (T3/T4)	60/450 (13·3%)	65/427 (15·2%)	−3·6	24·6	
Other / unknown	10/109 (9·2%)	26/99 (26·3%)	−11·0	7·8	0·24 (0·10 − 0·61)
**(i) Tumour grade (trend χ^2_1 = 0.7; 2p > 0.1; NS)**					
Well−differentiated	7/611 (1·1%)	15/608 (2·5%)	−3·2	5·1	
Moderately	149/3710 (4·0%)	162/3754 (4·3%)	−8·7	75·6	0·89 (0·66 − 1·20)
Poorly	316/3906 (8·1%)	347/3933 (8·8%)	−11·2	159·2	0·93 (0·76 − 1·14)
Grade unknown	106/849 (12·5%)	121/824 (14·7%)	−8·3	53·7	0·86 (0·60 − 1·22)
**(j) Histological type (χ^2_1 = 2.9; 2p = 0.09)**					
Ductal	408/5790 (7·0%)	467/5831 (8·0%)	−30·7	207·8	0·86 (0·72 − 1·03)
Lobular	34/594 (5·7%)	47/600 (7·8%)	−8·9	14·7	0·55 (0·28 − 1·07)
Unknown	136/2692 (5·1%)	131/2688 (4·9%)	5·1	61·0	
**(k) BMI (trend χ^2_1 = 0.1; 2p > 0.1; NS)**					
<20	36/507 (7·1%)	52/524 (9·9%)	−6·1	16·1	
20 – <25	195/2971 (6·6%)	228/3040 (7·5%)	−14·2	98·0	0·86 (0·67 − 1·12)
25 – <30	159/2383 (6·7%)	174/2332 (7·5%)	−9·7	75·4	0·88 (0·65 − 1·18)
30+	135/2151 (6·3%)	148/2159 (6·9%)	−11·0	60·3	0·83 (0·60 − 1·16)
Unknown	53/1064 (5·0%)	43/1064 (4·0%)	3·8	22·9	
**(l) Ki−67 (trend χ^2_1 = 0.7; 2p > 0.1; NS)**					
<10%	5/363 (1·4%)	12/380 (3·2%)	−2·9	3·8	
10 – <20%	18/629 (2·9%)	13/652 (2·0%)	2·8	6·6	
20 – <50%	43/840 (5·1%)	56/812 (6·9%)	−9·0	22·7	0·67 (0·39 − 1·15)
50+%	51/366 (13·9%)	52/369 (14·1%)	1·5	20·9	
Unknown	461/6878 (6·7%)	512/6906 (7·4%)	−27·3	230·7	0·89 (0·75 − 1·05)
**Total**	**578/ 9076 (6·4%)**	**645/ 9119 (7·1%)**	**−37·7**	**290·4**	**0·878 (0·783 − 0·985) 2p = 0·03**

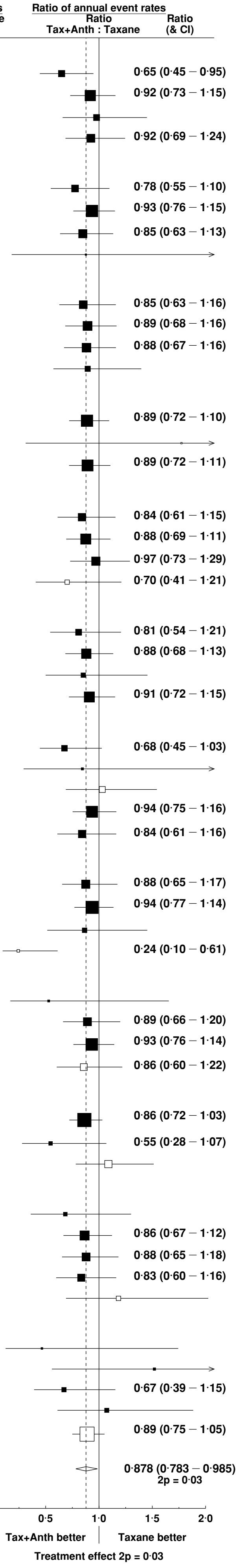

*Trials which randomised both HER2 negative and positive tumours shown separately to those trials which had HER2 negative only or positive only eligibility criteria.

P 19: 5-year risk of recurrence split by tumour size in trials of taxane plus anthracycline versus taxane without anthracycline. All trial comparisons (a)-(d).

1–20 mm (T1)

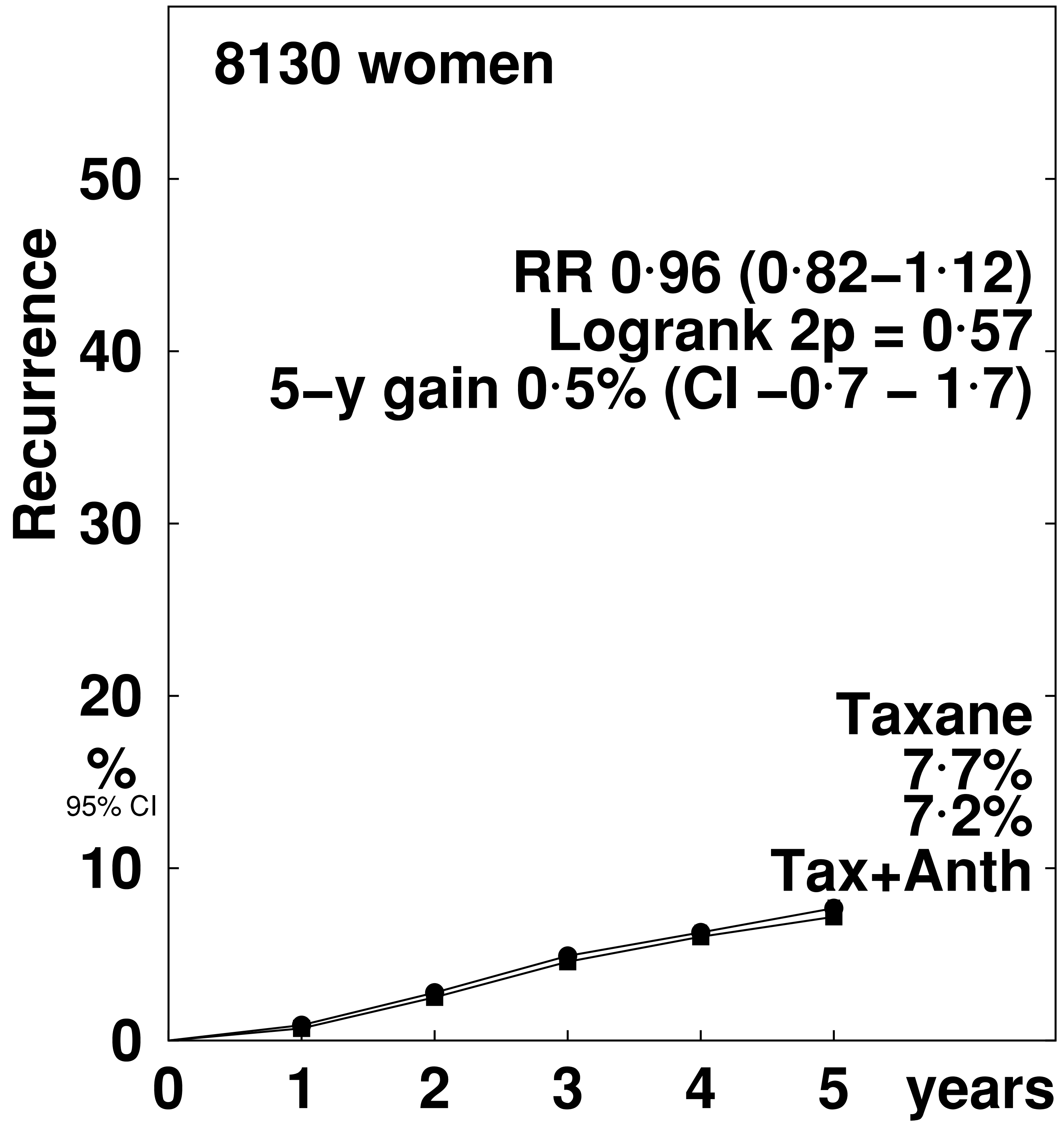

Recurrence rates (% / year) and logrank analyses

Allocation	Years 0 – 1	Years 2 – 4	Year 5+
Tax+Anth	1·28 (100 / 7802)	1·68 (169 / 10075)	1·10 (62 / 5660)
Taxane	1·38 (106 / 7681)	1·73 (173 / 9989)	1·05 (58 / 5535)
Rate ratio, from	0·90 CI 0·68 – 1·19	0·95 CI 0·77 – 1·19	1·06 CI 0·74 – 1·53
(O–E) / V	–5·2 / 49·0	–3·8 / 81·9	1·8 / 28·9

Entry age 21–50 mm (T2)

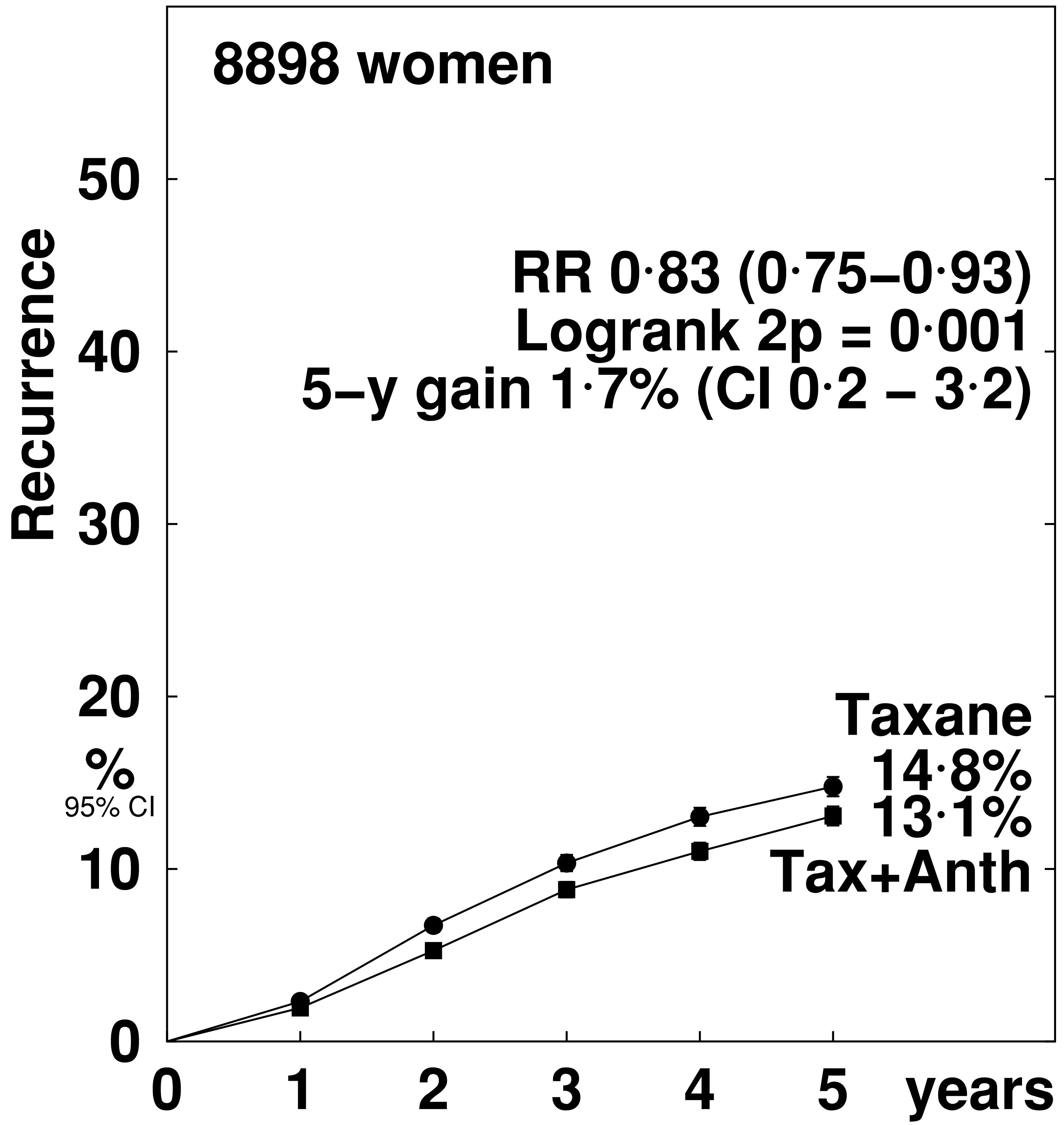

Recurrence rates (% / year) and logrank analyses

Allocation	Years 0 – 1	Years 2 – 4	Year 5+
Tax+Anth	2·73 (226 / 8274)	2·96 (301 / 10155)	1·48 (86 / 5797)
Taxane	3·40 (286 / 8419)	3·03 (313 / 10316)	2·10 (122 / 5806)
Rate ratio, from	0·78 CI 0·65 – 0·93	0·95 CI 0·80 – 1·11	0·68 CI 0·52 – 0·90
(O–E) / V	–29·9 / 117·9	–7·9 / 145·3	–18·8 / 49·5

> 50 mm (T3/T4)

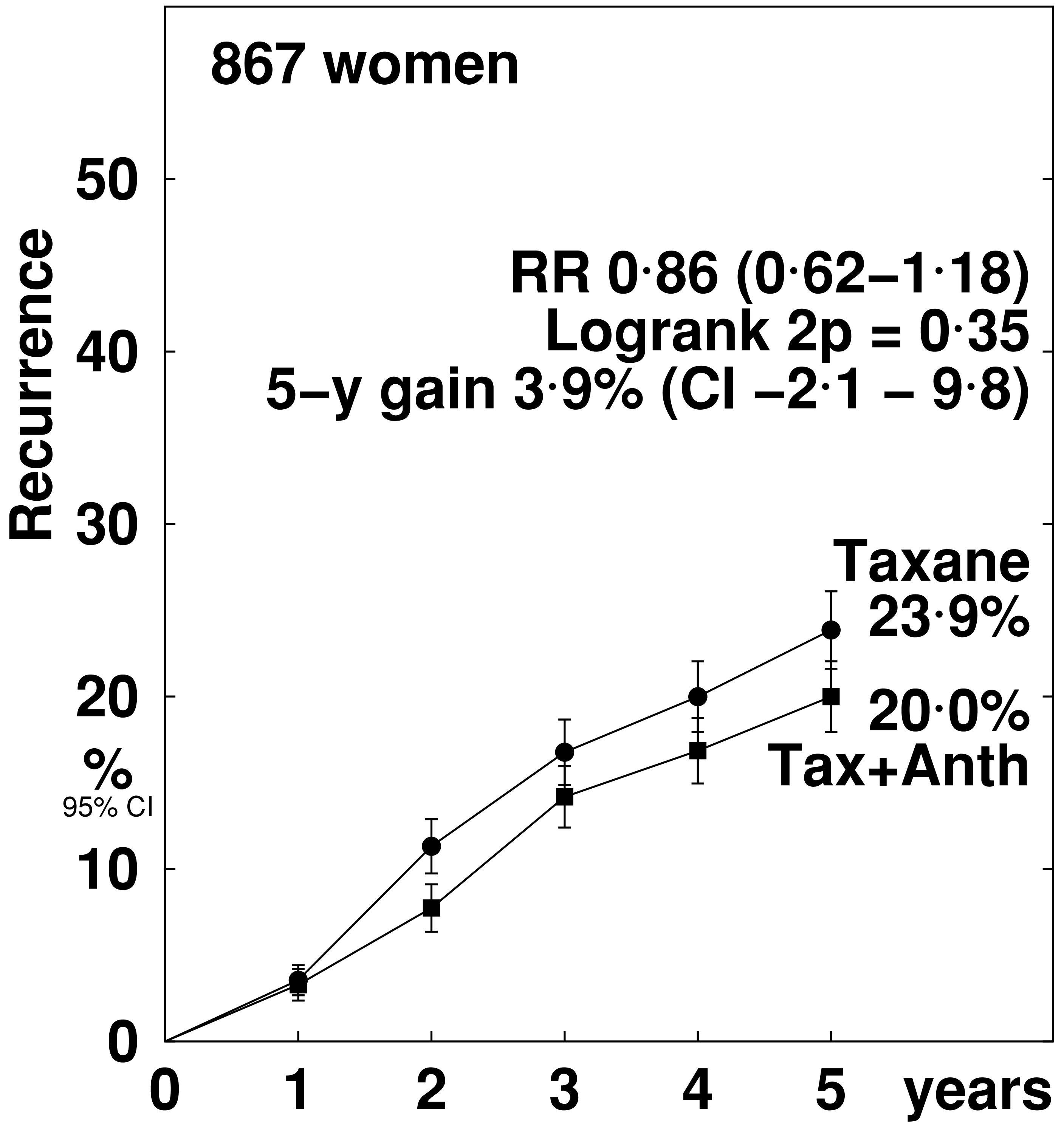

Recurrence rates (% / year) and logrank analyses

Allocation	Years 0 – 1	Years 2 – 4	Year 5+
Tax+Anth	4·34 (36 / 829)	4·74 (47 / 992)	2·68 (14 / 523)
Taxane	5·61 (44 / 784)	5·30 (48 / 906)	2·53 (11 / 434)
Rate ratio, from	0·66 CI 0·39 – 1·09	0·93 CI 0·59 – 1·48	1·47 CI 0·59 – 3·65
(O–E) / V	–6·2 / 14·8	–1·3 / 18·0	1·8 / 4·7

P 20: 5-year risk of recurrence split by tumour grade in trials of taxane plus anthracycline versus taxane without anthracycline. All trial comparisons (a)-(d).

Well−differentiated

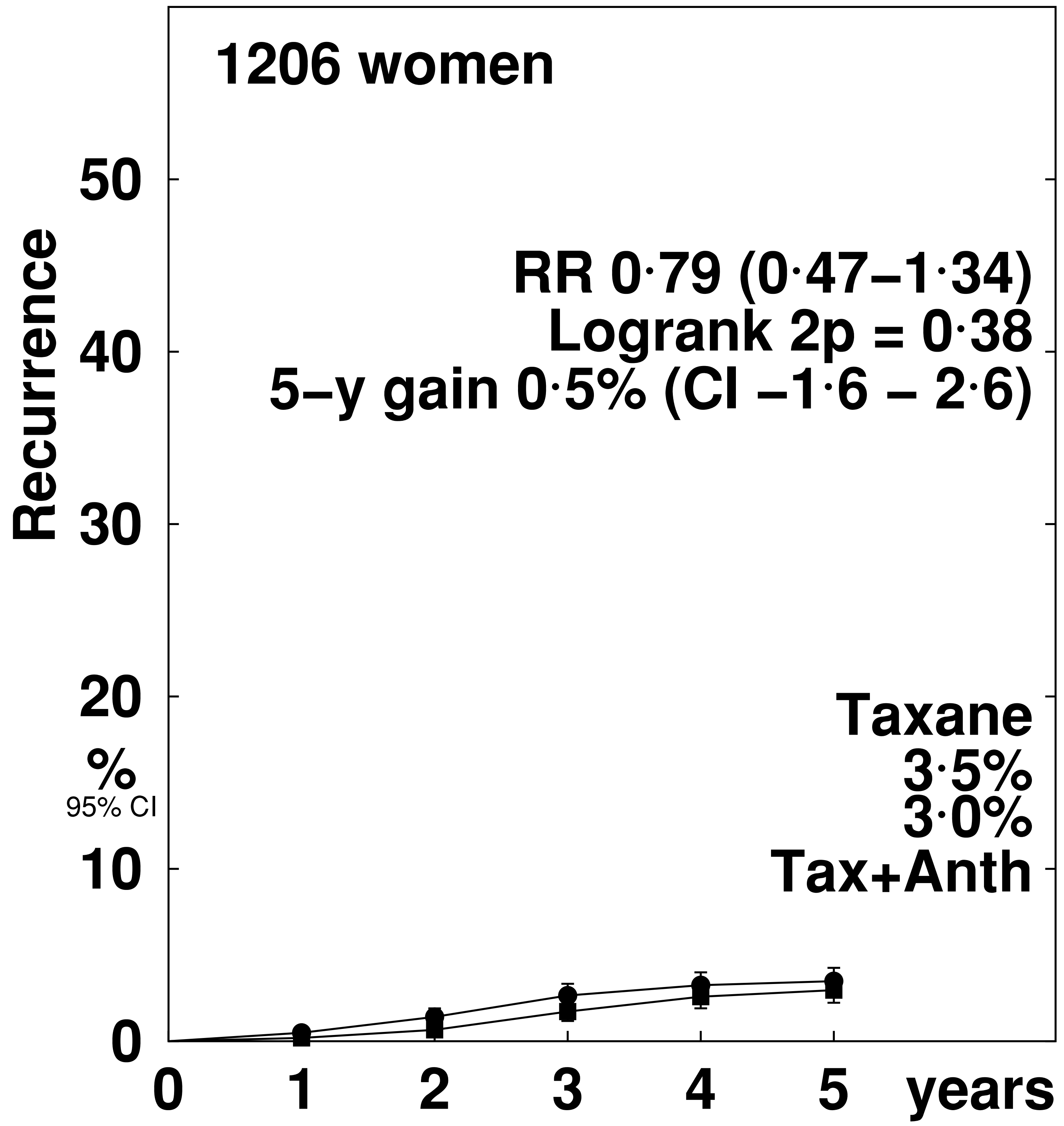

Recurrence rates (% / year) and logrank analyses

Allocation	Years 0 − 1	Years 2 − 4	Year 5+
Tax+Anth	0·34 (4 / 1169)	0·82 (13 / 1581)	1·05 (11 / 1044)
Taxane	0·70 (8 / 1141)	0·71 (11 / 1544)	1·24 (13 / 1045)
Rate ratio, from	0·48 CI 0·15 − 1·52	1·10 CI 0·48 − 2·51	0·73 CI 0·31 − 1·71
(O−E) / V	−2·1 / 2·9	0·5 / 5·6	−1·7 / 5·3

Moderately

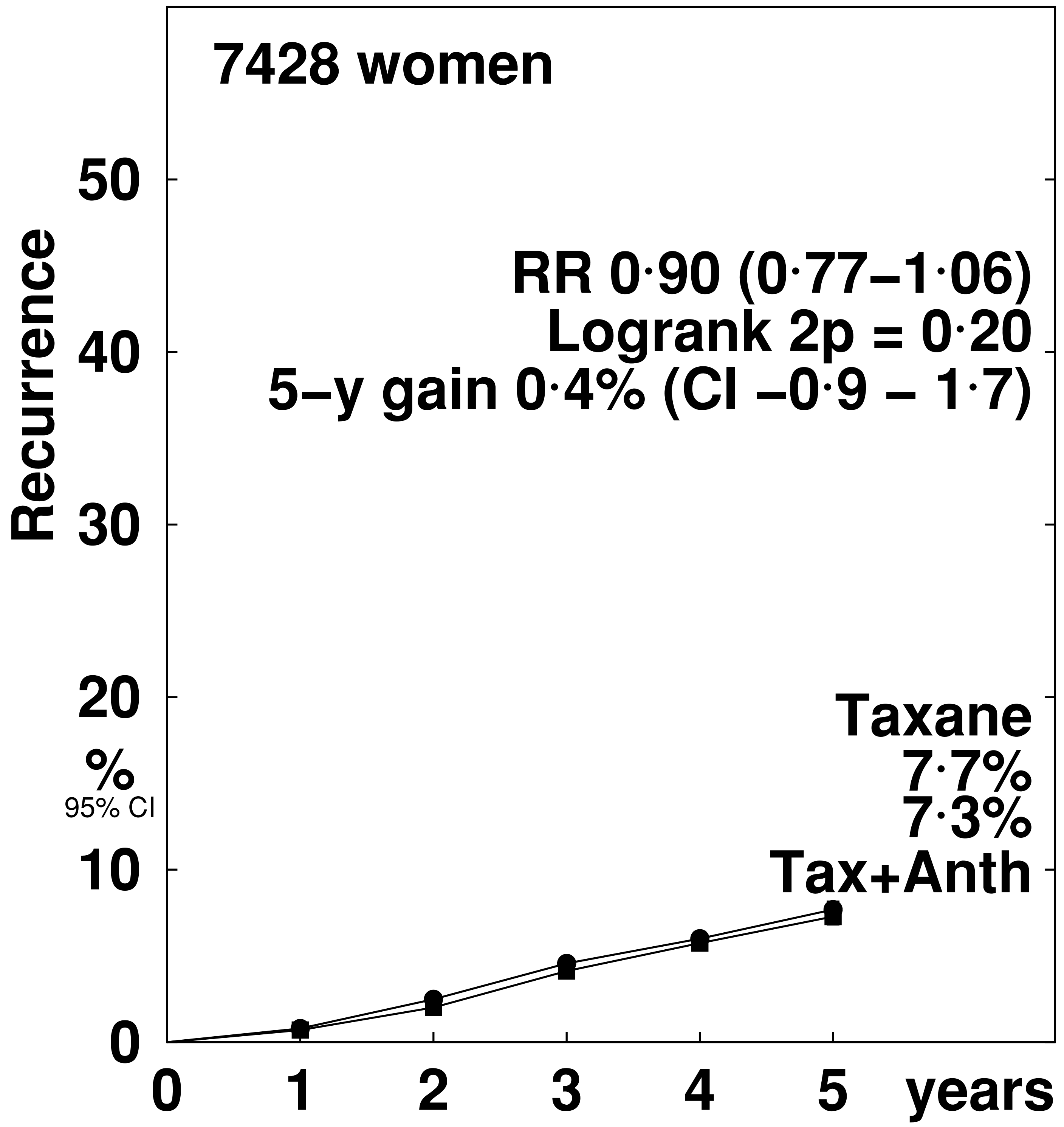

Recurrence rates (% / year) and logrank analyses

Allocation	Years 0 − 1	Years 2 − 4	Year 5+
Tax+Anth	1·04 (73 / 7053)	1·88 (170 / 9063)	1·16 (52 / 4491)
Taxane	1·23 (87 / 7098)	1·82 (166 / 9134)	1·48 (64 / 4315)
Rate ratio, from	0·81 CI 0·59 − 1·11	1·01 CI 0·82 − 1·26	0·75 CI 0·52 − 1·08
(O−E) / V	−8·3 / 38·4	1·1 / 81·0	−8·3 / 28·3

Poorly

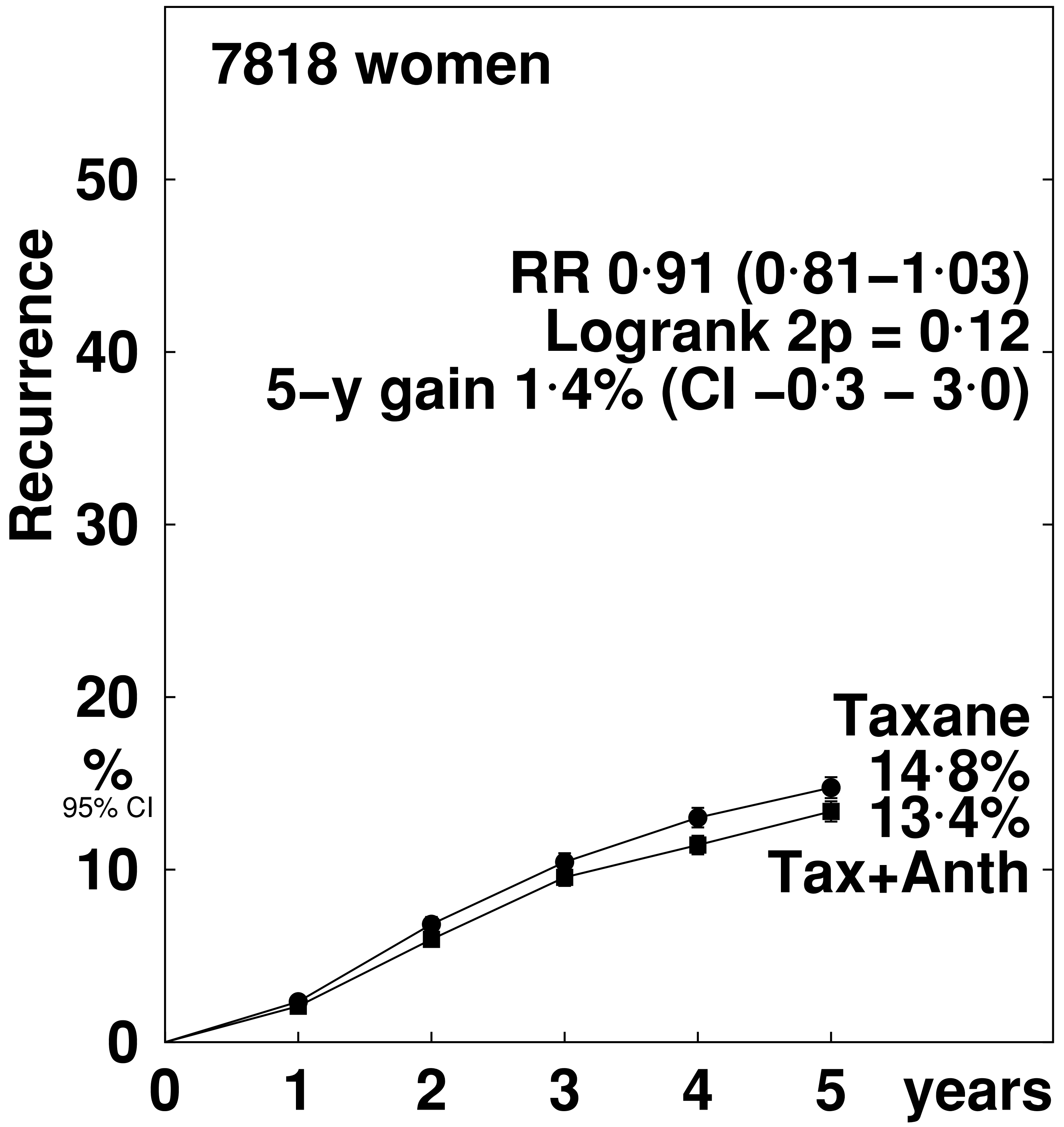

Recurrence rates (% / year) and logrank analyses

Allocation	Years 0 − 1	Years 2 − 4	Year 5+
Tax+Anth	3·04 (221 / 7277)	2·80 (247 / 8817)	1·35 (68 / 5043)
Taxane	3·51 (257 / 7315)	3·04 (271 / 8926)	1·30 (67 / 5154)
Rate ratio, from	0·87 CI 0·72 − 1·04	0·92 CI 0·77 − 1·10	1·04 CI 0·74 − 1·46
(O−E) / V	−16·1 / 111·9	−10·5 / 124·3	1·1 / 32·8

P21: Mortality by cause and incidence of second cancers. All trial comparisons (a)-(d).

	Events Tax+Anth (n=9076)	Taxane (n=9169)	(O-E)	Variance	Rate Ratio	95% CI	p
Death without recurrence	137	125	3.1	62.6	1.05	0.82 - 1.35	0.70
Death with recurrence	578	645	-37.7	290.4	0.88	0.78 – 0.99	0.03
Any death	715	770	-34.6	353.0	0.91	0.82 – 1.01	0.07
**Death without recurrence (selected groups of causes)**							
**Vascular disease:**	30	20	4.6	12.1	1.46	0.83 – 2.57	0.19
Stroke	8	6	1.0	3.5	1.33	0.47 – 3.79	0.59
Pulmonary embolus	2	1	0.5	0.7	2.04	0.20 – 21.3	0.55
Heart & other vascular	20	13	3.1	7.9	1.48	0.74 – 2.97	0.27
**Other primary cancer:**	34	29	0.4	14.7	1.03	0.62 – 1.71	0.92
Acute myeloid leukaemia	4	2	1.0	1.5	1.93	0.39 – 9.57	0.42
Lung	5	7	-0.9	3.0	0.74	0.24 – 2.30	0.60
Other cancer	25	20	0.3	10.3	1.03	0.56 – 1.90	0.93
Other specified cause	30	36	-2.9	15.8	0.83	0.51 – 1.36	0.47
Unknown cause	43	40	1.0	20.1	1.05	0.68 – 1.63	0.82
**Death without recurrence**							
By entry age (years) %/woman-years							
<55	0.16 (47/29125)	0.12 (36/29615)	6.4	20.5	1.37	0.89 – 2.11	0.16
55 – 69	0.35 (70/19832)	0.39 (75/19358)	-4.3	35.5	0.89	0.64 – 1.23	0.47
≥70	1.39 (20/1439)	0.92 (14/1525)	1.0	6.6	1.16	0.54 – 2.50	0.70
All ages	0.27 (137/50396)	0.25 (125/50498)	3.1	62.6	1.05	0.82 – 1.35	0.70
**Death without recurrence during first year**							
By entry age (years) %/woman-years							
<55	0.06 (3/4855)	0.04 (2/4928)	0.5	1.2	1.52	0.25 – 9.08	0.65
55 – 69	0.47 (16/3430)	0.44 (15/3407)	1.0	7.6	1.14	0.56 – 2.32	0.72
≥70	1.81 (5/276)	1.04 (3/286)	0.5	1.7	1.34	0.30 – 6.03	0.70
All ages	0.28 (24/8561)	0.23 (20/8621)	2.0	10.5	1.21	0.66 – 2.22	0.54
**Second primary cancer incidence**							
By entry age (years) %/woman-years							
<55	0.41 (99/24386)	0.35 (88/24867)	6.9	45.9	1.16	0.87 – 1.55	0.31
55 – 69	0.57 (87/15698)	0.57 (88/15439)	-2.8	42.8	0.94	0.69 – 1.26	0.67
≥70	0.82 (7/854)	0.45 (4/901)	0.2	1.9	1.11	0.27 – 4.61	0.89
All ages	0.47 (193/40938)	0.44 (180/41207)	4.3	90.5	1.05	0.85 – 1.29	0.65

Second cancer incidence	Tax+Anth	Taxane
**Haematological**		
AML	12	2
Lymphoma	4	2
**Lung/Thoracic**	23	33
**Colorectal**	15	9
**Upper Gastrointestinal**		
Gastric	7	4
Oesophagus	1	0
Small intestine	0	2
Pancreas	3	7
**Gynaecological**		
Uterine/Endometrial	12	8
Ovarian	6	10
Cervical	3	8
Vulva	0	3
**Urological**		
Renal	4	3
Bladder	2	0
**Thyroid**	17	14
**Epiglottis / Tonsil**	2	0
**Melanoma**	8	11
**Brain**	1	1
**Soft tissue /Bone**	3	3
**Unspecified**	70	60
	193	180

P22: AML incidence in trials of taxane plus anthracycline versus taxane without anthracycline. All trial comparisons (a)-(d)

Year code and study name	Regimens compared and drug doses (mg/m²) per cycle. Any unstated doses are as in the non-anthracycline comparator	Cumulative doses Taxane	Cumulative doses Anth	Events/Women Allocated Tax+Anth	Events/Women Allocated Taxane	Tax+Anth events* Logrank O–E	Tax+Anth events* Variance of O–E	Ratio of annual event rates Tax+Anth : Taxane
**(a) Concurrent docetaxel plus anthracycline vs same dose docetaxel plus cyclophosphamide**								
07& USO 06090 / 11271	$6(A_{50}D_{75}C_{500}\ v\ D_{75}C_{600})_{q3}$	D_{450} vs D_{450}	A_{300}	1/647	0/649	0·5	0·2	
09J NSABP B–46–I	$6(A_{50}D_{75}C_{500}\ v\ D_{75}C_{600})_{q3}$	D_{450} vs D_{450}	A_{300}	2/538	0/539	0·9	0·5	
09R NATT Shanghai	$6((A_{50}/E_{60})\ D_{75}C_{500}\ v\ D_{75}C_{600})_{q3}$‡	D_{450} vs D_{450}	A_{300}/E_{360}	0/51	0/45			
**(a) subtotal**				**3/ 1236 (0·2%)**	**0/ 1233 (0·0%)**	**1·4**	**0·7**	**6·82 (0·71 – 65·73) increase 2p = 0·10**
**(b) Sequential taxane plus anthracycline versus higher cumulative dose docetaxel plus cyclophosphamide**								
08L DBCG 07 READ	$(3E_{90}C_{600};3D_{100}\ v\ 6D_{75}C_{600})_{q3}$	D_{300} vs D_{450}	E_{270}	3/1001	1/1011	1·0	1·0	
09& Kanagawa Japan	$(3FE_{100}C_{500};3D_{100}\ v\ 6D_{75}C_{600})_{q3}$‡	D_{300} vs D_{450}	E_{300}	0/53	0/50			
09= NSABP B–49	$(4A_{60}C_{q2/q3};12P_{80q1}/4P_{175q2})/(6A_{50}D_{75}C_{500q3})\ v\ 6D_{75}C_{600q3}$	$D_{450}/P_{700-960}$ vs D_{450}	$A_{240-300}$	3/932	0/938	1·5	0·7	
09M WSG Plan B	$(4E_{90}C_{600};4D_{100}\ v\ 6D_{75}C_{600})_{q3}$	D_{400} vs D_{450}	E_{360}	0/1227	0/1222			
10V MASTER, Fudan	$(3FE_{100}C_{500};3D_{100}\ v\ 6D_{75}C_{600})_{q3}$	D_{300} vs D_{450}	E_{300}	0/523	0/524			
**(b) subtotal**				**6/ 3736 (0·2%)**	**1/ 3745 (0·0%)**	**2·5**	**1·7**	**4·28 (0·97 – 18·90) increase 2p = 0·06**
**(c) Taxane plus anthracycline versus higher taxane ± capecitabine**								
00W N–SAS–BC 02 Japan	$(4A_{60}C_{600};4P_{175}\ v\ 8P_{175})_{q3}$	P_{700} vs P_{1400}	A_{240}	0/263	0/267			
00W N–SAS–BC 02 Japan	$(4A_{60}C_{600};4D_{75}\ v\ 8D_{75})_{q3}$	D_{300} vs D_{600}	A_{240}	0/265	0/265			
01V Multicentre France	$(6E_{100}D_{75}\ v\ 8D_{100})_{q3}$	D_{450} vs D_{800}	E_{600}	*(21 patients)*		*(no data)*		
05] Asan, Seoul	$(4A_{60}C_{600};4D_{75}\ v\ 4Vrb_{25d1,8}Cap_{2000d1-14};4D_{75})_{q3}$‡	D_{300} vs D_{300}	A_{240}	*(75 patients)*		*(no data)*		
06Z3 MINDACT:EORTC10041	$(3FE_{100}C_{500};3D_{100}\ v\ 6D_{75}Cap_{1650d1-14})_{q3}$	D_{300} vs D_{450}	E_{300}	1/194	0/198	0·1	0·1	
09[2 Remagus 04, France	$(4FE_{100}C;\ 4D_{100}\ v\ 6D_{75}Cap)_{q3}$‡	D_{400} vs D_{450}	E_{400}	*(100 patients)*		*(no data)*		
11N PUMCH–Breast–TCX	$6((A_{50}/E_{75})D_{75}C_{500}\ v\ D_{75}C_{500}Cap_{950d1-14})_{q3}$	D_{450} vs D_{450}	A_{300}/E_{450}	*(400 patients)*		*(no data)*		
**(c) subtotal with data §**				**1/ 722 (0·1%)**	**0/ 730 (0·0%)**	**0·1**	**0·1**	**3·21 (0·01 – 869·34) increase 2p > 0.1; NS**
**(d) Taxane plus anthracycline versus higher taxane plus carboplatin (confounded)**								
01M BCIRG 006	$(4A_{60}C_{600};4D_{100}\ v\ 6D_{75}Cpt_{AUC6})_{q3}+12Trz_{q1};\ Trz_{q3}$ to 1yr	D_{400} vs D_{450}	A_{240}	2/1074	1/1075	0·5	0·7	
06~ CAMS NeoPaclitaxel	$6(P_{175}E_{75}\ v\ P_{175}Cpt_{AUC5})_{q3}$	P_{1050} vs P_{1050}	E_{450}	*(80 patients)*		*(no data)*		
09T TRYPHAENA	$6PtzTrz+(3FE_{100}C_{500};3D_{75-100}\ v\ 6D_{75}Cpt_{AUC6})_{q3}$‡	$D_{225-300}$ vs D_{450}	E_{300}	*(225 patients)*		*(no data)*		
10U CH–BC–007	$(4E_{90}C_{600};\ 4D_{75}/P_{175}\ v\ 6D_{75}/P_{175}Cpt_{AUC5})_{q3}$	D_{300} v D_{450} or P_{700} vs P_{1050}	E_{360}	*(298 patients)*		*(no data)*		
11M CH–BC–012	$(4E_{80}C_{600};4P_{175}\ v\ 8P_{150}Cpt_{AUC3})_{q2}$	P_{700} vs P_{1200}	E_{360}	*(132 patients)*		*(no data)*		
**(d) subtotal with data §**				**2/ 1074 (0·2%)**	**1/ 1075 (0·1%)**	**0·5**	**0·7**	**1·86 (0·19 – 17·91) increase 2p > 0.1; NS**
**Total (a + b + c + d)**				**12/ 6768 (0·2%)**	**2/ 6783 (0·0%)**	**4·6**	**3·4**	**3·900 (1·339 – 11·360) increase 2p = 0·01**

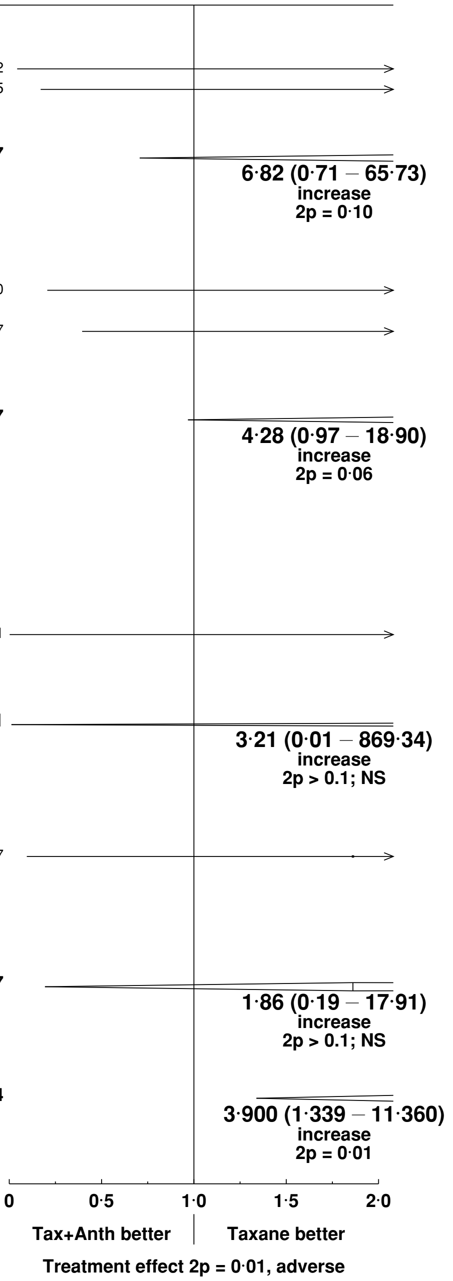

■ 99% or ◇ 95% confidence intervals

Heterogeneity between 4 subtotals: χ^2_3 = 0·7; p > 0.1; NS

Heterogeneity within subtotals: χ^2_2 = 0·5; p > 0.1; NS

Heterogeneity between 6 trials: χ^2_5 = 1·1; p > 0.1; NS

‡ Pre–operative chemotherapy

v.v Vice versa

Taxanes: D = docetaxel; P = paclitaxel

Anthracyclines: A = doxorubicin (Adriamycin); E = epirubicin

Other agents: C = cyclophosphamide; F = fluorouracil; M = methotrexate; Trz = trastuzumab; Vrb = vinorelbine; Cap = capecitabine; Cpt = carboplatin; Ptz = pertuzumab

P23: Sensitivity analyses for death without recurrence by tumour size and nodal status. All trial comparisons (a)-(d).

Category	Events/Women: Allocated Tax+Anth	Events/Women: Allocated Taxane	Tax+Anth events*: Logrank O−E	Tax+Anth events*: Variance of O−E
**(a) Tumour size (trend χ^2_1 = 0.1; 2p > 0.1; NS)**				
**1 – 20 mm (T1)**	62/4106 (1·5%)	51/4038 (1·3%)	3·8	27·9
**21 – 50 mm (T2)**	67/4411 (1·5%)	65/4555 (1·4%)	1·4	32·3
**> 50 mm (T3/T4)**	8/450 (1·8%)	9/427 (2·1%)	0·6	3·9
**Other / unknown**	0/109 (0·0%)	0/99 (0·0%)		
**(b) Nodal status (trend χ^2_1 = 0.2; 2p > 0.1; NS)**				
**N0**	45/3633 (1·2%)	44/3699 (1·2%)	−0·2	21·3
**N1−3**	67/3760 (1·8%)	47/3739 (1·3%)	7·6	27·3
**N4+**	23/1121 (2·1%)	30/1111 (2·7%)	−3·5	12·5
**N unknown**	2/562 (0·4%)	4/570 (0·7%)	−0·9	1·5
**■ Total**	**137/ 9076 (1·5%)**	**125/ 9119 (1·4%)**	**3·1**	**62·6**

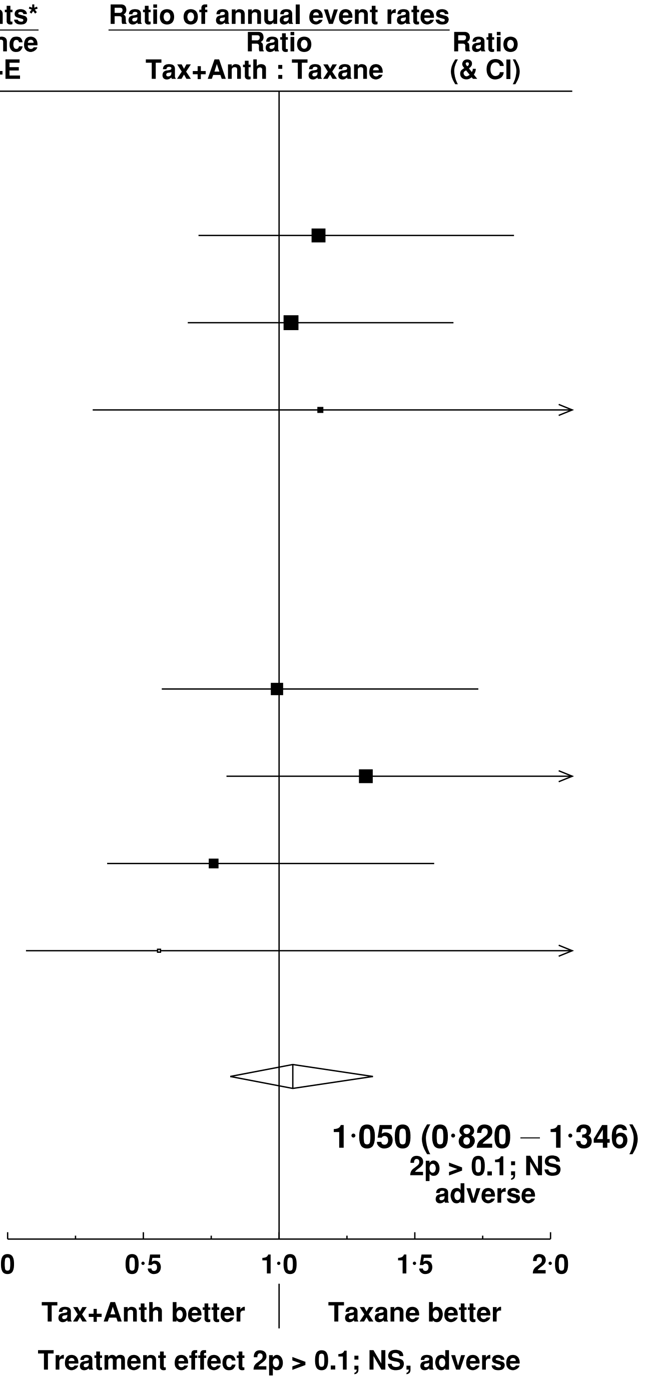

■ 99% or ◇ 95% confidence intervals

P24: Any recurrence in trials of taxane-plus-anthracycline-based regimen vs same, or more (<doubled or ~doubled) non-taxane cytotoxic chemotherapy

Year code and study name		Regimens compared and drug doses (mg/m²) per cycle. Any unstated doses are as in the controls	Cumulative doses of taxane & anthracycline	Anthra-cycline difference	Events/Women: Allocated taxane	Events/Women: Allocated non-tax.	Taxane events*: Logrank O–E	Taxane events*: Variance of O–E	Ratio of annual event rates Taxane : Non-tax.
**(a) Taxane (D or P)–plus–anthracycline (A or E)–based regimen † vs the SAME non–taxane cytotoxic chemotherapy (ie, unconfounded trials of adding a taxane)**									
94D$_1$	CALGB 9344	4**AC**; 4**P**$_{175}$ vs 4**A**$_{90}$**C**$_{600}$	†**P**$_{700}$ **A**$_{360}$ vs **A**$_{360}$	0	218/531	234/526	–16·8	100·8	
94D$_2$	CALGB 9344	4**AC**; 4**P**$_{175}$ vs 4**A**$_{75}$**C**$_{600}$	†**P**$_{700}$ **A**$_{300}$ vs **A**$_{300}$	0	220/525	257/528	–24·5	106·5	
94D$_3$	CALGB 9344	4**AC**; 4**P**$_{175}$ vs 4**A**$_{60}$**C**$_{600}$	†**P**$_{700}$ **A**$_{240}$ vs **A**$_{240}$	0	226/534	249/526	–18·0	107·4	
95J	NSABP B–27*	4**AC**‡; 4**D**$_{100}$‡ vs 4(**A**$_{60}$**C**$_{600}$)‡	†**D**$_{400}$ **A**$_{240}$ vs **A**$_{240}$	0	574/1607	598/1608	–18·1	271·0	
95K	NSABP B–28	4**AC**; 4**P**$_{225}$ vs 4**A**$_{60}$**C**$_{600}$	†**P**$_{900}$ **A**$_{240}$ vs **A**$_{240}$	0	476/1531	508/1529	–22·9	226·0	
98B	Taxit216 Italy	4**E**; 4**D**$_{100}$; 4**CMF**$_{q4}$ vs 4**E**$_{120}$; 4(**C**$_{600×2}$**M**$_{40×2}$**F**$_{600×2}$)$_{q4}$	†**D**$_{400}$ **E**$_{480}$ vs **E**$_{480}$	0	122/486	144/486	–14·0	61·5	
99T	GOIM 9902 Italy	4**D**$_{100}$; 4**EC** vs 4**E**$_{120}$**C**$_{600}$	†**D**$_{400}$ **E**$_{480}$ vs **E**$_{480}$	0	89/376	92/374	–8·8	39·4	
01Z	PEGASE 07 France	4**EC**‡; 4**D**$_{75}$**F**$_{750x5}$ vs 4**E**$_{150}$**C**$_{4000}$‡	†**D**$_{300}$ **E**$_{600}$ vs **E**$_{600}$	0	*(174 patients)*		*(no data)*		
04!	Craiova Dolj	4**P**$_{200}$‡; 4**EC**‡ vs 4**E**$_{60}$**C**$_{600}$‡	†**P**$_{800}$ **E**$_{240}$ vs **E**$_{240}$	0	*(124 patients)*		*(no data)*		
**(a) subtotal with data §**					**1925/ 5590 (34·4%)**	**2082/ 5577 (37·3%)**	**–123·1**	**912·5**	**0·87 (0·82 – 0·93) reduction 2p = 0·00005**
**(b) Taxane–plus–anthracycline–based regimen (taxane courses given alone) † vs MORE (but < doubled) non–taxane cytotoxic chemotherapy**									
95T	HORG Greece	4**D**$_{100}$; 4**EC** vs 6(**F**$_{700}$**E**$_{75}$**C**$_{700}$)	†**D**$_{400}$ **E**$_{300}$ vs **E**$_{450}$¶	**E**$_{150}$	105/391	121/397	–11·7	48·9	
97R	HE1097 Greece	3**E**$_{q2}$; 3**P**$_{250q2}$; 3**CMF**$_{q2}$ vs 4(**E**$_{110}$)$_{q2}$; 4(**C**$_{840}$**M**$_{50}$**F**$_{840}$)$_{q2}$	†**P**$_{750}$ **E**$_{330}$ vs **E**$_{440}$	**E**$_{110}$	130/304	123/300	0·0	53·5	
97V	IBIS 02 Italy	4**P**$_{175}$;4**E**$_{75}$**Vrb**$_{25x2}$ vs 4**E**$_{100}$;4(**C**$_{600x2}$**M**$_{40x2}$**F**$_{600x2}$)$_{q4}$	†**P**$_{700}$ **E**$_{300}$ vs **E**$_{400}$	**E**$_{100}$	54/122	45/122	5·9	21·6	
98D$_1$	BIG 02–98	(3**A**$_{75}$;3**D**$_{100}$ vs 4**A**$_{75}$ or 4**A**$_{60}$**C**$_{600}$); 3(**C**$_{600x2}$**M**$_{40x2}$**F**$_{600x2}$)$_{q4}$	†**D**$_{300}$ **A**$_{225}$ vs **A**$_{270}$ (240/300)	**A**$_{45}$	69/227	85/237	–5·4	33·8	
98D$_2$	BIG 02–98	(3**A**$_{75}$;3**D**$_{100}$ vs 4**A**$_{75}$ or 4**A**$_{60}$**C**$_{600}$); 3(**C**$_{600x2}$**M**$_{40x2}$**F**$_{600x2}$)$_{q4}$	†**D**$_{300}$ **A**$_{225}$ vs **A**$_{270}$ (240/300)	**A**$_{45}$	129/732	160/731	–16·5	68·1	
99K	GEICAM 9906 Spain	4**FEC**; 8(**P**$_{100}$)$_{q1}$ vs 6(**F**$_{600}$**E**$_{90}$**C**$_{600}$)	†**P**$_{800}$ **E**$_{360}$ vs **E**$_{540}$	**E**$_{180}$	176/614	214/632	–22·1	89·7	
00E	FinHer/FBCG 00–01	3**D**$_{(80/100)}$; 3**FEC** vs 3**Vrb**$_{25×3}$; 3(**F**$_{600}$**E**$_{60}$**C**$_{600}$)	†**D**$_{240}$ **E**$_{180}$ vs **E**$_{180}$¶	0≠	118/502	162/507	–21·8	62·5	
00F$_1$	MA.21, Canada	6(**E**$_{120}$**C**$_{830}$)$_{q2}$; 4**P**$_{175}$ vs 6(**F**$_{500x2}$**E**$_{60x2}$**C**$_{75x14}$)$_{q4}$	†**P**$_{700}$ **E**$_{720}$ vs **E**$_{720}$	0≠	76/285	79/281	–0·9	35·3	
00F$_2$	MA.21, Canada	6(**E**$_{120}$**C**$_{830}$)$_{q2}$; 4**P**$_{175}$ vs 6(**F**$_{500x2}$**E**$_{60x2}$**C**$_{75x14}$)$_{q4}$	†**P**$_{700}$ **E**$_{720}$ vs **E**$_{720}$	0≠	92/416	93/420	–0·3	44·6	
00S$_1$	WSG/AGO AM–02	4**E**$_{90}$**C**$_{600}$; 4**D**$_{100}$ vs 6(**F**$_{500}$**E**$_{100}$**C**$_{500}$)	†**D**$_{400}$ **E**$_{360}$ vs **E**$_{600}$	**E**$_{240}$	82/811	98/795	–8·8	43·5	
00U	AERO B–2000 France	4**FEC**; 4**P**$_{175}$ vs 6(**F**$_{500}$**E**$_{100}$**C**$_{500}$)	†**P**$_{700}$ **E**$_{400}$ vs **E**$_{600}$	**E**$_{200}$	130/420	133/417	1·3	59·2	
03$	GEICAM 2004–04	12**P**$_{80q1}$‡;4**FAC**‡ vs 6(**F**$_{500}$**A**$_{50}$**C**$_{500}$)‡	†**P**$_{960}$ **A**$_{200}$ vs **A**$_{300}$¶	**A**$_{100}$	34/138	33/135	1·1	10·0	
03R	GIM 1 Italy	4**E**$_{90}$**C**; 4**D**$_{100}$ vs 6(**F**$_{600}$**E**$_{75}$**C**$_{600}$)	†**D**$_{400}$ **E**$_{360}$ vs **E**$_{450}$¶	**E**$_{90}$	*(1636 patients)*		*(no data)*		
03U	GEICAM 2003–02	4**FEC**; 8(**P**$_{100}$)$_{q1}$ vs 6(**F**$_{500}$**E**$_{50}$**C**$_{500}$)	†**P**$_{800}$ **E**$_{200}$ vs **E**$_{300}$¶	**E**$_{100}$	102/951	110/974	–4·4	51·6	
**(b) subtotal with data §**					**1297/ 5913 (21·9%)**	**1456/ 5948 (24·5%)**	**–83·7**	**622·1**	**0·87 (0·81 – 0·95) reduction 2p = 0·0008**
**(c) Taxane–plus–anthracycline–based regimen (taxane given concurrently) © vs MORE (but < doubled) non–taxane cytotoxic chemotherapy**									
95E	LMU Munich Germany	6**E**$_{60}$**D**$_{75}$ vs 4**E**$_{90}$**C**$_{600}$;3**C**$_{600}$**M**$_{40}$**F**$_{600}$	©**D**$_{300}$ **E**$_{360}$ vs **E**$_{360}$	0≠	*(36 patients)*		*(no data)*		
96#	H–V Athens	3**EP**‡;3**EP**$_{200}$ vs 3**FEC**‡;3**F**$_{600}$**E**$_{75}$**C**$_{600}$	©**P**$_{1200}$ **E**$_{450}$ vs **E**$_{450}$¶	0≠	*(70 patients)*		*(no data)*		
96%	Multicentre Germany	4(**EP**$_{175}$)$_{q2}$; 3**CMF**$_{q2}$ vs 4(**E**$_{90}$**C**$_{600}$); 3(**C**$_{600}$**M**$_{40}$**F**$_{600}$)	©**P**$_{700}$ **E**$_{360}$ vs **E**$_{360}$	0≠	49/113	65/115	–10·8	23·5	
96W$_1$	ECTO Italy*	(4**A**$_{60}$**P**$_{200}$; 4**CMF**$_{q4}$) vs 4**A**$_{75}$; 4(**C**$_{600×2}$**M**$_{40×2}$**F**$_{600×2}$)$_{q4}$	©**P**$_{800}$ **A**$_{240}$ vs **A**$_{300}$	**A**$_{60}$	113/451	135/453	–14·4	57·6	
96W$_2$	ECTO Italy*	(4**A**$_{60}$**P**$_{200}$; 4**CMF**$_{q4}$)‡ vs **same control patients**	©**P**$_{800}$ **A**$_{240}$ vs **A**$_{300}$	**A**$_{60}$	140/451	135/453	–1·9	63·6	
96X	GONO Italy MIG 5	4**E**$_{90}$**P**$_{175}$ vs 6(**F**$_{600}$**E**$_{60}$**C**$_{600}$)	©**P**$_{700}$ **E**$_{360}$ vs **E**$_{360}$¶	0≠	155/535	143/520	5·5	68·8	
97H$_1$	*Multicentre France*	*4**AP**$_{200}$‡ vs 4**A**$_{60}$**C**$_{600}$‡*	*©**P**$_{800}$ **A**$_{240}$ vs **A**$_{240}$*	*0≠*	*0/133*	*0/67*			
97L	BCIRG 001 / GEICAM 9703	6**D**$_{75}$**AC** vs 6(**F**$_{500}$**A**$_{50}$**C**$_{500}$)	©**D**$_{450}$ **A**$_{300}$ vs **A**$_{300}$	0≠	233/745	283/746	–32·8	118·7	
98D$_1$	BIG 02–98	(4**A**$_{50}$**D**$_{75}$ vs 4**A**$_{75}$ or 4**A**$_{60}$**C**$_{600}$); 3(**C**$_{100x14}$**M**$_{40x2}$**F**$_{600x2}$)$_{q4}$	©**D**$_{300}$ **A**$_{200}$ vs **A**$_{270}$ (240/300)	**A**$_{70}$	81/221	85/237	3·3	36·1	
98D$_2$	BIG 02–98	(4**A**$_{50}$**D**$_{75}$ vs 4**A**$_{75}$ or 4**A**$_{60}$**C**$_{600}$); 3(**C**$_{100x14}$**M**$_{40x2}$**F**$_{600x2}$)$_{q4}$	©**D**$_{300}$ **A**$_{200}$ vs **A**$_{270}$ (240/300)	**A**$_{70}$	156/739	160/731	–4·0	73·9	
98T	ECOG EST2197	4**AD**$_{60}$ vs 4**A**$_{60}$**C**$_{600}$	©**D**$_{240}$ **A**$_{240}$ vs **A**$_{240}$	0≠	349/1476	325/1476	11·3	162·1	
99%	GEICAM 9805 / TARGET 0	6**D**$_{75}$**AC** vs 6(**F**$_{500}$**A**$_{50}$**C**$_{500}$)	©**D**$_{450}$ **A**$_{300}$ vs **A**$_{300}$¶	0≠	79/539	89/521	–10·3	39·6	
99A	Anglo–Celtic II	6**A**$_{50}$**D**$_{75}$‡ vs 6**A**$_{60}$**C**$_{600}$‡	©**D**$_{450}$ **A**$_{300}$ vs **A**$_{360}$	**A**$_{60}$	70/183	82/180	–6·8	33·2	
99N	RAPP–01 France	4**A**$_{50}$**D**$_{75}$ vs 4**A**$_{60}$**C**$_{600}$	©**D**$_{300}$ **A**$_{200}$ vs **A**$_{240}$	**A**$_{40}$	30/311	33/316	–1·5	15·4	
01E$_{1+3}$	PACS 04 France	6**E**$_{75}$**D**$_{75}$ vs 6(**F**$_{500}$**E**$_{100}$**C**$_{500}$)	©**D**$_{450}$ **E**$_{450}$ vs **E**$_{600}$	**E**$_{150}$	373/1494	417/1515	–21·7	185·2	
02L	PetrovStPetersb'g	4**P**$_{200}$**A**‡ vs 4**F**$_{600}$**A**$_{60}$**C**$_{600}$‡	©**P**$_{800}$ **A**$_{240}$ vs **A**$_{240}$		*(103 patients)*		*(no data)*		
02T	Craiova Dolj	4**E**$_{75}$**P**$_{175}$‡ vs 4**F**$_{500}$**E**$_{100}$**C**$_{500}$‡	©**P**$_{700}$ **E**$_{300}$ vs **E**$_{400}$	**E**$_{100}$	*(98 patients)*		*(no data)*		
04N$_{3+4}$	MATADOR/BOOG 05–02	6**D**$_{75}$**A**$_{50}$**C**$_{600}$ vs 6**A**$_{60}$**C**$_{600q2}$	©**D**$_{450}$ **A**$_{300}$ vs **A**$_{360}$	**A**$_{60}$	52/332	56/332	–2·0	24·0	
09~$_1$	TaiNAC Taiwan	4**D**$_{35x2}$**E**$_{45x2}$‡ vs 4**E**$_{45x2}$**F**$_{2000x2}$**Fol**$_{300x2}$‡	©**D**$_{280}$ **E**$_{360}$ vs **E**$_{360}$	0≠	6/25	6/18	0·1	1·9	
**(c) subtotal with data §**					**1886/ 7748 (24·3%)**	**2014/ 7680 (26·2%)**	**–69·8**	**782·4**	**0·91 (0·85 – 0·98) reduction 2p = 0·01**
**(d) Taxane–plus–anthracycline–based regimen † vs DOUBLED (or ≈ doubled) non–taxane cytotoxic chemotherapy**									
94B	MDA 94–002	4**P**$_{250}$‡; 4**FAC** vs 4**FAC**‡; 4(**F**$_{500×2}$**A**$_{50}$**C**$_{500}$)	†**P**$_{1000}$ **A**$_{200}$ vs **A**$_{400}$¶	**A**$_{200}$	63/239	66/235	–0·3	28·7	
96F	*Aberdeen Scotland*	*4**VcrAC**‡; 4**D**$_{100}$ vs 4**VcrAC**‡; 4(**Vcr**$_{1·5}$**A**$_{50}$**C**$_{1000}$)*	*†**D**$_{400}$ **A**$_{200}$ vs **A**$_{400}$¶*	***A**$_{200}$*	*4/52*	*12/52*	*–4·0*	*3·4*	
97A	DEVA UK	3**E**$_{q4}$; 3**D**$_{100}$ vs 6(**E**$_{50×2}$)$_{q4}$	†**D**$_{300}$ **E**$_{300}$ vs **E**$_{600}$	**E**$_{300}$	78/406	102/397	–14·5	37·0	
97J	PACS 01 France	3**FEC**; 3**D**$_{100}$ vs 6(**F**$_{500}$**E**$_{100}$**C**$_{500}$)	†**D**$_{300}$ **E**$_{300}$ vs **E**$_{600}$	**E**$_{300}$	288/1003	328/996	–22·5	142·0	
00F$_1$	MA.21, Canada	4**A**$_{60}$**C**$_{600}$; 4**P**$_{175}$ vs 6(**F**$_{500x2}$**E**$_{60x2}$**C**$_{75x14}$)$_{q4}$	†**P**$_{700}$ **A**$_{240}$ vs **E**$_{720}$	**E**$_{720}$–**A**$_{240}$	106/290	79/281	16·1	39·6	
00F$_2$	MA.21, Canada	4**A**$_{60}$**C**$_{600}$; 4**P**$_{175}$ vs 6(**F**$_{500x2}$**E**$_{60x2}$**C**$_{75x14}$)$_{q4}$	†**P**$_{700}$ **A**$_{240}$ vs **E**$_{720}$	**E**$_{720}$–**A**$_{240}$	115/412	93/420	14·1	50·0	
01F	TACT UK	4**FEC**; 4**D**$_{100}$ vs 8(**F**$_{600}$**E**$_{60}$**C**$_{600}$)	†**D**$_{400}$ **E**$_{240}$ vs **E**$_{480}$¶	**E**$_{240}$	351/1258	387/1265	–13·7	167·5	
01G$_{1+2}$	TACT UK	4(**F**$_{600}$**E**$_{60}$**C**$_{600}$); 4**D**$_{100}$ vs 4**E**$_{100}$; 4(**C**$_{600×2}$**M**$_{40×2}$**F**$_{600×2}$)$_{q4}$	†**D**$_{400}$ **E**$_{240}$ vs **E**$_{400}$	**E**$_{160}$	253/815	268/824	–5·9	117·7	
01J	EORTC10994/BIG 0–01	3**D**$_{100}$‡;3**E**$_{90}$**D**$_{75}$‡ vs 6**F**$_{500}$**E**$_{100}$**C**$_{500}$‡	†**D**$_{300}$ **E**$_{270}$ vs **E**$_{600}$	**E**$_{330}$	283/928	328/928	–18·5	125·8	
01T	LMU Munich ADEBAR	4**E**$_{90}$**C**$_{600}$; 4**D**$_{100}$ vs 6(**F**$_{500x2}$**E**$_{60x2}$**C**$_{75x14}$)$_{q4}$	†**D**$_{400}$ **E**$_{360}$ vs **E**$_{720}$	**E**$_{360}$	176/688	147/675	13·4	73·6	
02D	GBG 42 / NNBC 3–Eur.	3**FEC**; 3**D**$_{100}$ vs 6(**F**$_{500}$**E**$_{100}$**C**$_{500}$)	†**D**$_{300}$ **E**$_{300}$ vs **E**$_{600}$	**E**$_{300}$	72/1333	72/1327	–0·5	34·7	
02G	Bari Italy	(4**EVrb**)‡;(4**D**$_{100}$ vs 4**E**$_{120}$**Vrb**$_{25x2}$)	†**D**$_{400}$ **E**$_{480}$ vs **E**$_{960}$	**E**$_{480}$			*(no data)*		
07"	Kolkata MC India	3**AC**;3**P**$_{175}$ vs 6**A**$_{60}$**C**$_{600}$	†**P**$_{525}$ **A**$_{180}$ vs **A**$_{360}$	**A**$_{180}$	*(50 patients)*		*(no data)*		
**(d) subtotal with data §**					**1789/ 7424 (24·1%)**	**1882/ 7400 (25·4%)**	**–36·2**	**820·1**	**0·96 (0·89 – 1·02) reduction 2p > 0.1; NS**
**Total (a + b + c + d)**					**6897/ 26675 (25·9%)**	**7434/ 26605 (27·9%)**	**–312·8**	**3137·1**	**0·905 (0·874 – 0·937) reduction 2p < 0·00001**

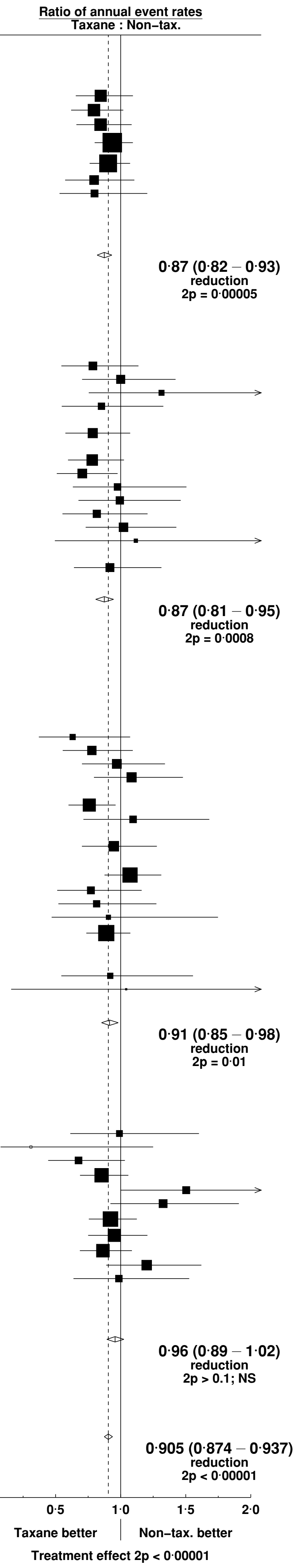

■ 99% or ◇ 95% confidence intervals

Heterogeneity between 4 subtotals: $\chi^2_3 = 4{\cdot}5$; p > 0.1; NS

Heterogeneity within subtotals: $\chi^2_{35} = 60{\cdot}0$; p = 0·005

Heterogeneity between 39 trials: $\chi^2_{38} = 64{\cdot}5$; p = 0·005

* For 3–way trials versus the same control patients
† Taxane courses do not overlap with any other chemotherapy courses; hence, total chemotherapy duration is increased
‡ Pre–operative chemotherapy
© Concurrent treatment with taxane and anthracycline; total chemotherapy duration and number of courses not increased
≠ Same cumulative anthracycline dose, but differences in other drugs
¶ Control anthracycline dose less than E90 or A60 per cycle
Taxanes: D = docetaxel; P = paclitaxel. Anthracyclines: A = doxorubicin (Adriamycin); E = Epirubicin. Other agents: C = cyclophosphamide; F = fluorouracil; Fol = folinic acid; M = methotrexate; Vcr = vincristine; Vrb = vinorelbine
(Not shown: G–CSF, erythropoietin, trastuzumab, antibiotic, hormonal, local or steroid therapies)
All regimens q3week (unless specified as q1, q2 or q4). Semicolon [;] indicates treatment sequence. ×14 means d1–14 po; ×2 (×3) means d1, d8 (d15) iv
03$ at discretion local investigators could have used epirubicin 100mg/m2 instead of doxorubicin 50mg/m2

P25: 10-year cumulative risk of (A) any recurrence, (B) breast cancer mortality, (C) death without recurrence, (D) all-cause mortality; in trials of taxane-plus-anthracycline-based regimen vs same, or more (<doubled or ~doubled) non-taxane cytotoxic chemotherapy

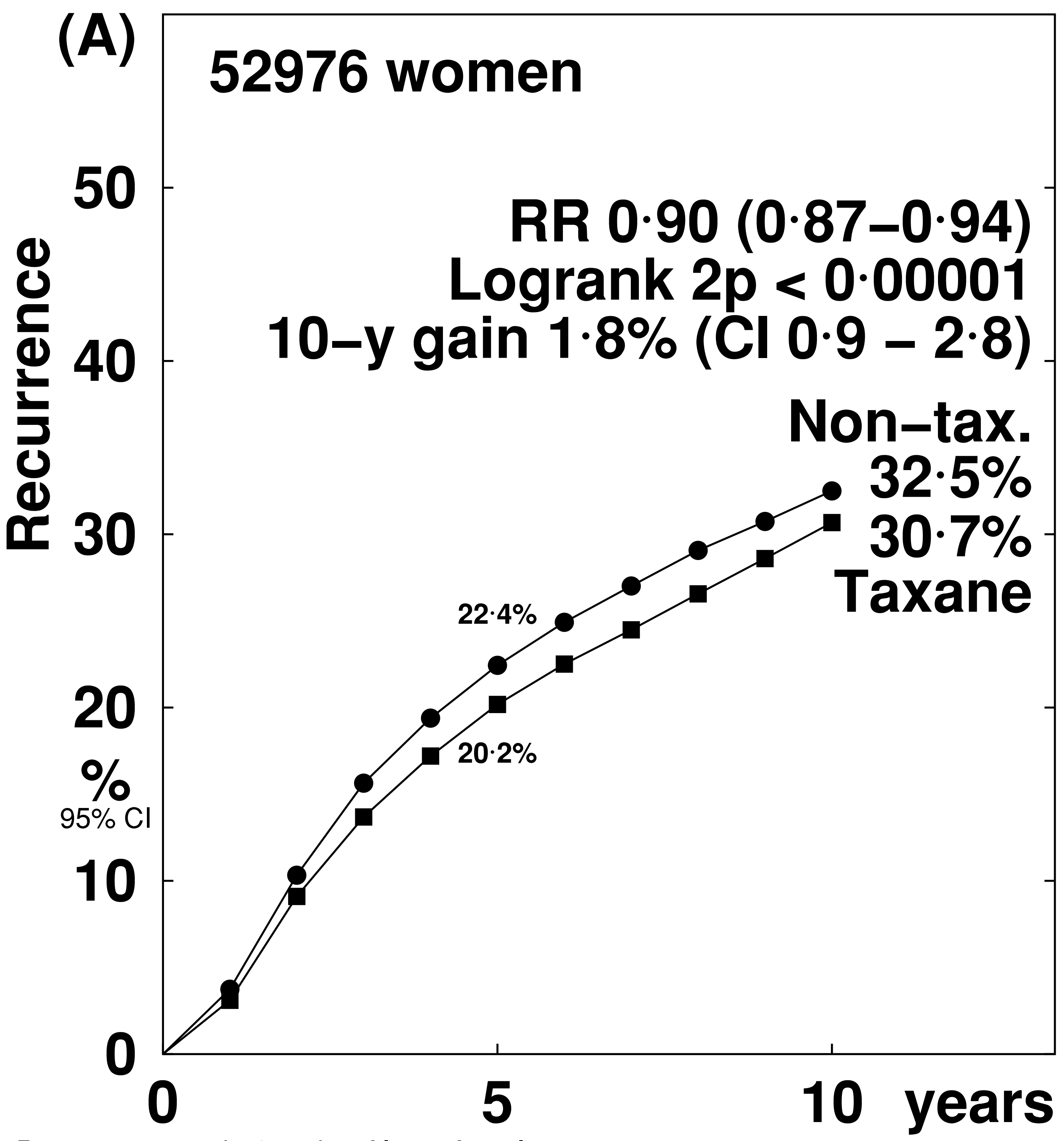

Recurrence rates (% / year) and logrank analyses

Allocation	Years 0 – 4	Years 5 – 9	Year 10+
Taxane	4·53 (5047 / 111389)	2·81 (1609 / 57166)	1·85 (237 / 12790)
Non–tax.	5·14 (5629 / 109489)	2·84 (1554 / 54691)	1·98 (239 / 12041)
Rate ratio, from	0·88 CI 0·85 – 0·92	0·99 CI 0·92 – 1·06	0·89 CI 0·74 – 1·07
(O–E) / V	–303·2 / 2400·4	–8·5 / 743·4	–13·3 / 111·0

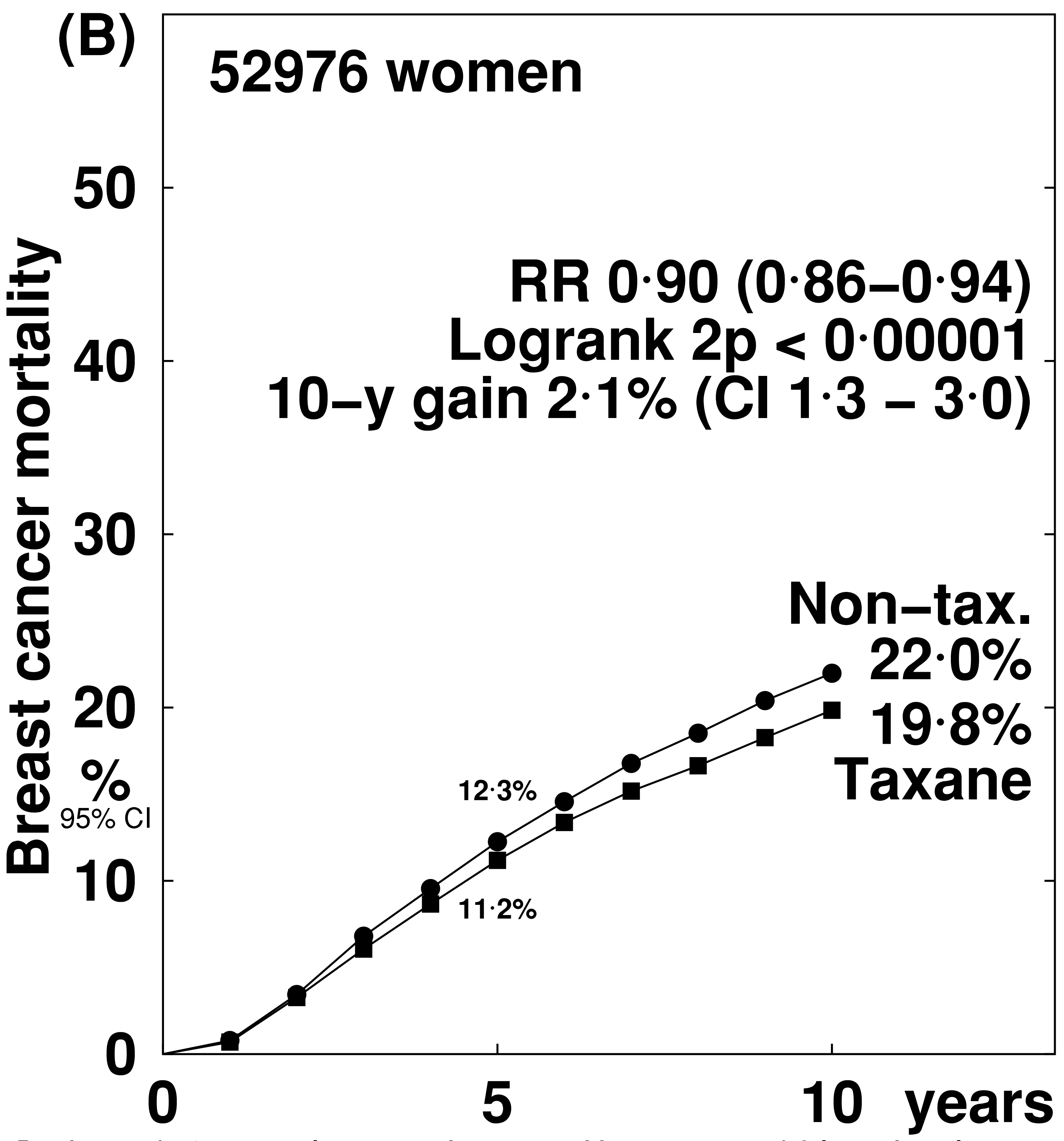

Death rates (% / year: total rate – rate in women without recurrence) & logrank analyses

Allocation	Years 0 – 4	Years 5 – 9	Year 10+
Taxane	2·31 CI 2·23 – 2·40	2·12 CI 2·00 – 2·23	1·70 CI 1·49 – 1·90
Non–tax.	2·58 CI 2·49 – 2·67	2·38 CI 2·26 – 2·50	1·54 CI 1·34 – 1·74
Rate ratio, from	0·91 CI 0·86 – 0·95	0·87 CI 0·81 – 0·94	1·09 CI 0·91 – 1·31
(O–E) / V	–133·1 / 1334·2	–95·1 / 679·6	9·9 / 113·4

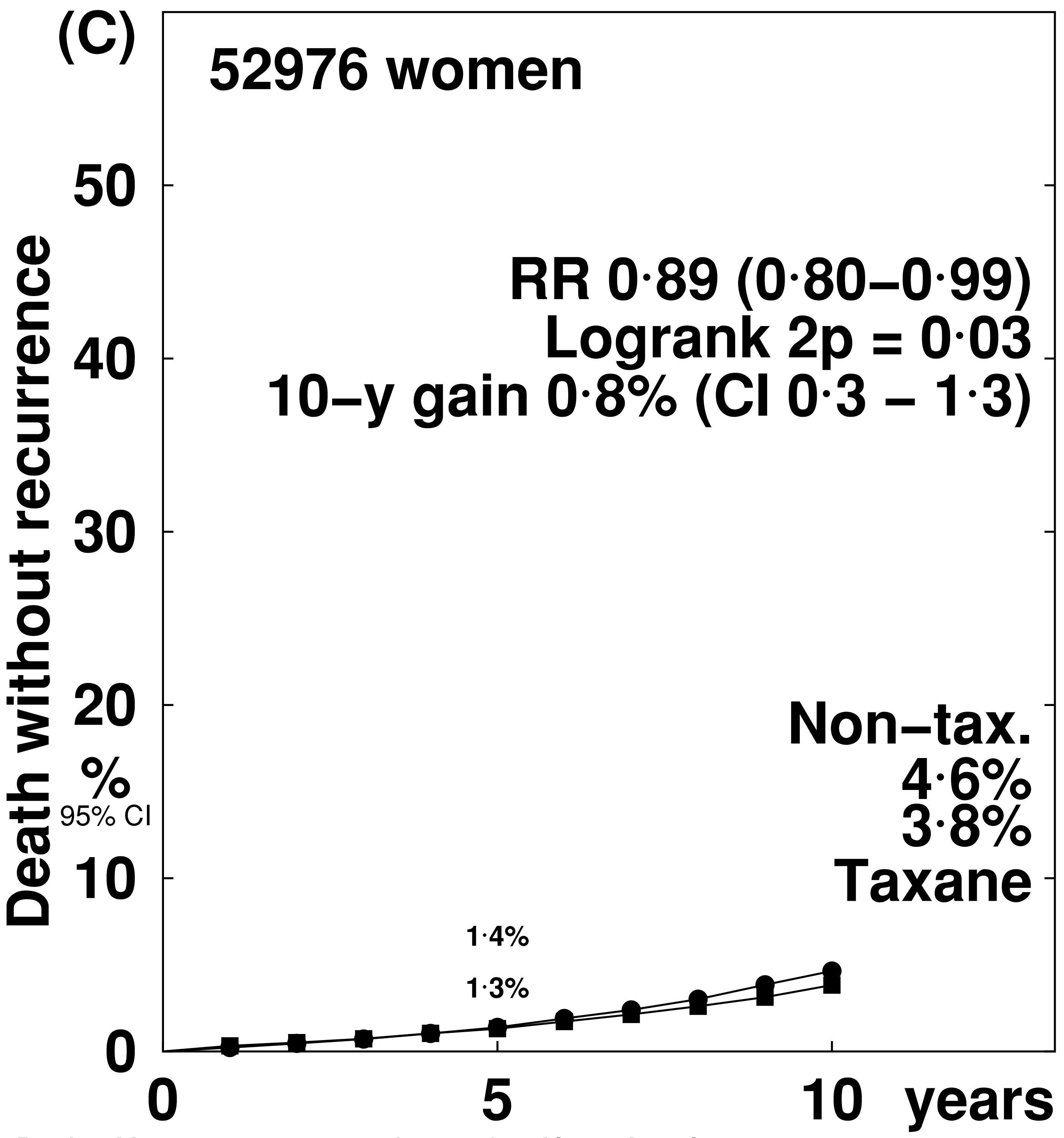

Death–without–recurrence rates (% / year) and logrank analyses

Allocation	Years 0 – 4	Years 5 – 9	Year 10+
Taxane	0·26 (293 / 111389)	0·50 (283 / 57166)	1·19 (152 / 12790)
Non–tax.	0·27 (301 / 109489)	0·63 (347 / 54691)	1·16 (140 / 12041)
Rate ratio, from	0·97 CI 0·82 – 1·14	0·77 CI 0·65 – 0·90	1·04 CI 0·82 – 1·33
(O–E) / V	–5·1 / 143·6	–39·3 / 149·2	2·8 / 65·8

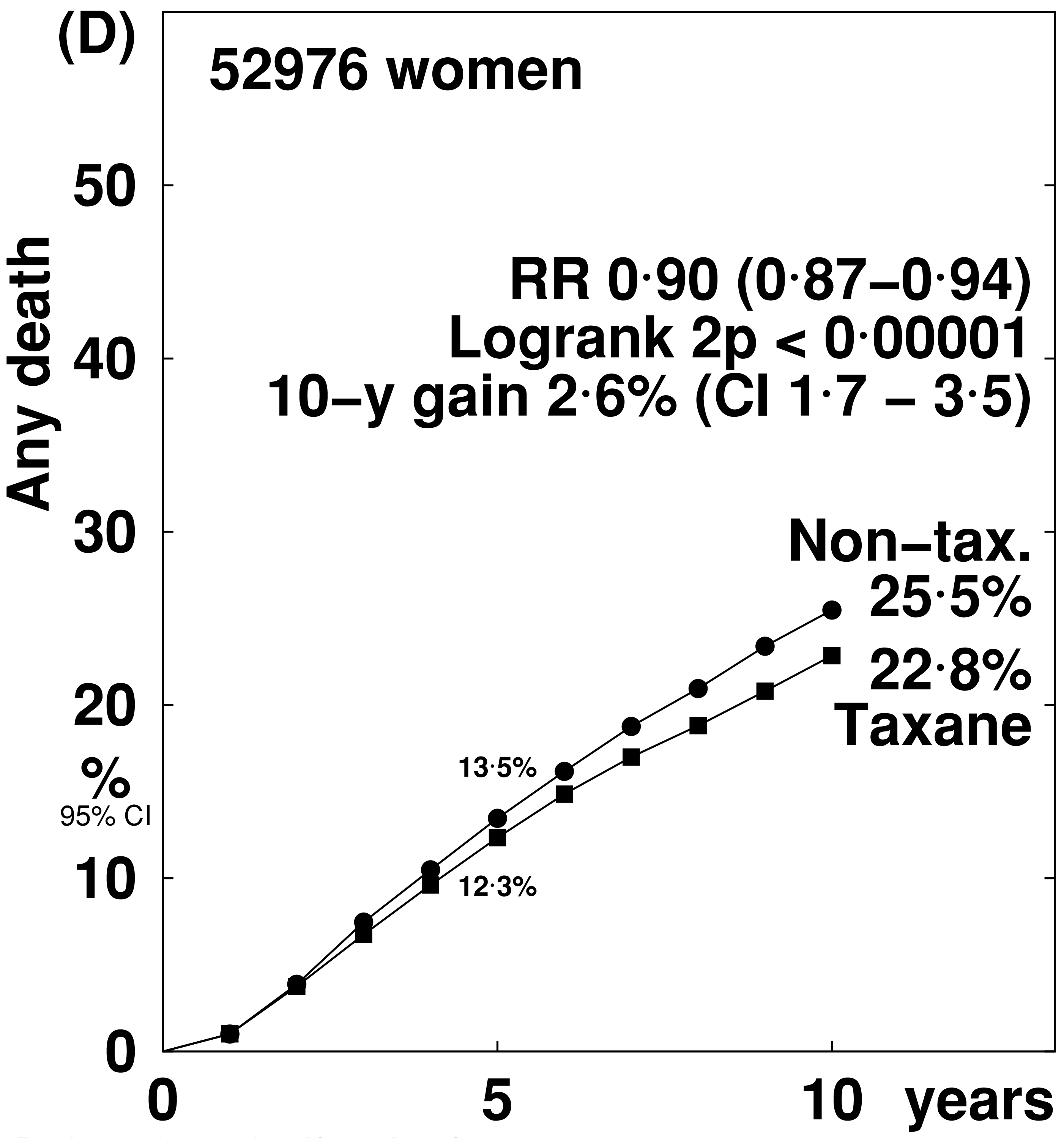

Death rates (% / year) and logrank analyses

Allocation	Years 0 – 4	Years 5 – 9	Year 10+
Taxane	2·57 (3040 / 118173)	2·59 (1663 / 64274)	2·79 (414 / 14837)
Non–tax.	2·85 (3349 / 117405)	3·01 (1878 / 62350)	2·65 (370 / 13967)
Rate ratio, from	0·91 CI 0·87 – 0·96	0·85 CI 0·79 – 0·91	1·07 CI 0·93 – 1·24
(O–E) / V	–138·1 / 1477·8	–134·4 / 828·8	12·7 / 179·2

P26: Any recurrence in trials of taxane-based regimens versus anthracyline-based regimens

Year code and study name	Treatment comparison	Cumulative taxane and anthracycline doses	Events/Women: Allocated Taxane	Events/Women: Allocated Anth	Taxane events: Logrank O−E	Taxane events: Variance of O−E
**(a) Docetaxel vs Anthracycline**						
97N USO 97−35	4($D_{75}C_{600}$ v AC)$_{q3}$	D_{300} vs A_{240}	63/506	82/510	−8·5	32·5
06Z1 MINDACT:EORTC10041	6($D_{75}Cap_{1650x14}$)$_{q3}$ v 6F(E/A)$C_{q3/q4}$	D_{450} vs E_{600}/A_{300}	32/454	44/455	−7·5	18·1
08) NCC Singapore	neo4($D_{75}C_{600}$ v AC)$_{q3}$	D_{300} vs A_{240}	(20 patients)		(no data)	
08Y Duke Univ USA	neo4($D_{75}C_{600}$ v AC)$_{q3}$	D_{300} vs A_{240}	(56 patients)		(no data)	
**■ (a) subtotal with data §**			**95/ 960 (9·9%)**	**126/ 965 (13·1%)**	**−16·0**	**50·6**
**(b) Paclitaxel vs Anthracycline**						
97[Epi−Tax trial Norway	4(P_{200} v E_{90})$_{q3}$‡	P_{800} vs E_{360}	62/114	52/109	4·5	23·2
02B CALGB 40101	12/18P_{80q1} v 4/6AC_{q3}	$P_{960/1440}$ vs $A_{240/360}$	43/287	42/284	0·6	20·2
02X CALGB 40101	(4/6P_{175} v 4/6AC)$_{q2}$	$P_{700/1050}$ vs $A_{240/360}$	185/1653	133/1647	26·3	75·9
**■ (b) subtotal**			**290/ 2054 (14·1%)**	**227/ 2040 (11·1%)**	**31·4**	**119·4**
**■ Total (a + b)**			**385/ 3014 (12·8%)**	**353/ 3005 (11·7%)**	**15·4**	**170·0**

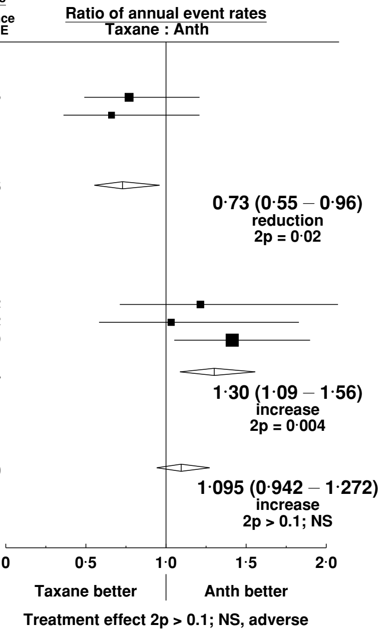

■ 99% or ◇ 95% confidence intervals

Difference between treatment effects in 2 subtotals: $\chi^2_1 = 12{\cdot}0$; 2p = 0·0005

Heterogeneity within subtotals: $\chi^2_3 = 2{\cdot}0$; p > 0.1; NS

Heterogeneity between 5 trials: $\chi^2_4 = 13{\cdot}9$; p = 0·007

§ 2 trials with no data do not contribute to subtotals or to the overall total.

‡ Pre−operative chemotherapy

Taxanes: D = docetaxel; P = paclitaxel

Anthracyclines: A = doxorubicin (Adriamycin); E = epirubicin

Other agents: C = cyclophosphamide; F = fluorouracil; Cap = capecitabine

P27: Any recurrence in trials of Docetaxel versus Paclitaxel

Year code and study name	Treatment comparison	Cumulative docetaxel and paclitaxel doses	Events/Women: Allocated Docetaxel	Events/Women: Allocated Paclitaxel	Docetaxel events: Logrank O–E	Docetaxel events: Variance of O–E
**(a) Docetaxel q2 or q3 vs Paclitaxel q2 or q3**						
99V ECOG EST1199	4**AC**; 4(**D**$_{100}$ v **P**$_{175}$)$_{q3}$	**D**$_{400}$ vs **P**$_{700}$	297/1262	390/1272	−47·8	158·4
00W N–SAS–BC 02 Japan	±4**AC**/**EC**; (4/8**D**$_{75}$ v 4/8**P**$_{175}$)$_{q3}$	**D**$_{300/600}$ vs **P**$_{700/1400}$	164/530	213/530	−28·9	85·8
04R HORG CT/04.22 Greece	4**FEC**; 4(**D**$_{75}$ v **P**$_{175}$)$_{q2}$	**D**$_{300}$ vs **P**$_{700}$	47/240	50/241	−1·2	20·4
??K Mansoura Egypt	4**AC**; 4(**D**$_{100}$ v **P**$_{175}$)$_{q3}$	**D**$_{400}$ vs **P**$_{700}$	*(160 patients)*		*(no data)*	
**(a) subtotal with data §**			**508/ 2032 (25·0%)**	**653/ 2043 (32·0%)**	**−77·9**	**264·6**
**(b) Docetaxel q1 vs Paclitaxel q1**						
99V ECOG EST1199	4**AC**; 12(**D**$_{35}$ v **P**$_{80}$)$_{q1}$	**D**$_{420}$ vs **P**$_{960}$	362/1260	323/1258	27·9	161·3
05C HE 10/05 Greece	3**E**$_{110q2}$; 3**CMF**; 9(**D**$_{35}$ v **P**$_{80}$)$_{q1}$	**D**$_{315}$ vs **P**$_{720}$	55/334	60/330	−4·3	26·9
07' KBCOG–06 Japan	4(**D**$_{75q3}$ v **P**$_{80d1,8,15q4}$)‡; 4**FEC**$_{q3}$‡	**D**$_{300}$ vs **P**$_{240}$	*(50 patients)*		*(no data)*	
**(b) subtotal with data §**			**417/ 1594 (26·2%)**	**383/ 1588 (24·1%)**	**23·6**	**188·1**
**Total (a + b)**			**925/ 3626 (25·5%)**	**1036/ 3631 (28·5%)**	**−54·3**	**452·7**

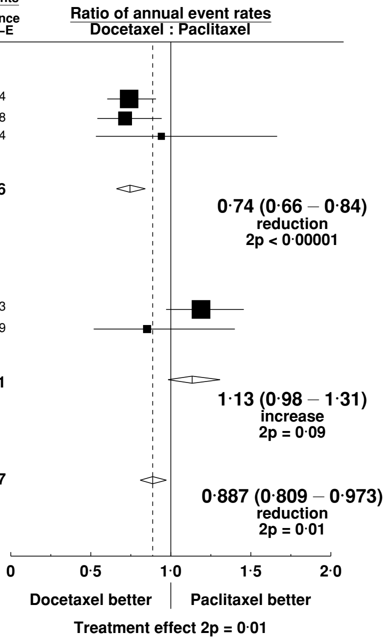

99% or 95% confidence intervals

Difference between treatment effects in 2 subtotals: $\chi^2_1 = 19{\cdot}4$; 2p = 0·00001

Heterogeneity within subtotals: $\chi^2_3 = 3{\cdot}8$; p > 0.1; NS

Heterogeneity between 5 trials: $\chi^2_4 = 23{\cdot}2$; p = 0·0001

§ 2 trials with no data do not contribute to subtotals or to the overall total.

‡ Pre−operative chemotherapy
Taxanes: D = docetaxel; P = paclitaxel
Anthracyclines: A = doxorubicin (Adriamycin); E = epirubicin
Other agents: C = cyclophosphamide; F = fluorouracil

P28: Any recurrence in trials comparing similar cumulative dose taxane chemotherapy delivered in smaller vs larger fractions

Year code and study name	Regimens compared and drug doses (mg/m²) per cycle	Difference mg/w	Events/Women: Allocated Smaller	Events/Women: Allocated Larger	Smaller events: Logrank O–E	Smaller events: Variance of O–E
**(a) Chemotherapy in smaller fractions: Paclitaxel**						
98K123456 MDA 98–240	(12$P_{80/150/175\,q1}$ v 4$P_{225\,q3}$); 4FAC	$P_{720–2100/12}$ vs $P_{900/12}$	14/128	29/126	−0·6	4·7
99V ECOG EST1199	4AC; (12$P_{80\,q1}$ v 4$P_{175\,q3}$)	$P_{960/12}$ vs $P_{700/12}$	323/1258	390/1272	−38·5	165·6
03* SWOG S0221	(15($A_{24}C_{60d1–7}$)$_{q1}$/6AC_{q2}); (12$P_{80\,q1}$ v 6$P_{175\,q2}$)	$P_{960/12}$ vs $P_{1050/12}$	306/1641	322/1653	−5·6	146·7
05C HE 10/05 Greece	3$E_{110\,q2}$; (3CMF_{q2};9$P_{80\,q1}$ v 3$P_{200\,q2}$;3CMF_{q2})	$P_{720/9}$ vs $P_{600/6}$	60/330	73/337	−6·0	30·7
05R PKU Beijing	CPt_{AUC6}‡ + 4($P_{60d1,8,15}$ v P_{175})$_{q3}$‡	$P_{720/12}$ vs $P_{700/12}$	(220 patients)		(no data)	
**(a) subtotal with data §**			**703/ 3357 (20·9%)**	**814/ 3388 (24·0%)**	**−50·8**	**347·7**
**(b) Chemotherapy in smaller fractions: Docetaxel**						
99V ECOG EST1199	4AC; (12$D_{35\,q1}$ v 4$D_{100\,q3}$)	$D_{420/12}$ vs $D_{400/12}$	362/1260	297/1262	34·4	154·7
00^ U Hull / Lincoln CH	4AC; (12$D_{33\,q1}$ v 4$D_{100\,q3}$)	$D_{400/12}$ vs $D_{400/12}$	10/41	11/41	−0·4	3·3
04Z BREAST−10	12$E_{30}D_{35\,q1}$ v 4$E_{75}D_{80\,q3}$	$D_{400/12}$ vs $D_{320/12}$	7/22	5/22	0·3	2·1
05@ UZLeuven Belgium	(4$F_{375}E_{75}C_{375}$;4$D_{75/v.v}$)$_{q2}$ v (3$F_{500}E_{100}C_{500}$;3$D_{100/v.v}$)$_{q3}$	$D_{300/8}$ vs $D_{300/9}$	8/54	11/31	−2·0	4·0
07e Emory Taxane trial	8($D_{50}Cap_{2000d1–7}$)$_{q2}$ v (4D_{100};4$Cap_{2000d1–14}$)$_{q3}$	$D_{400/12}$ vs $D_{400/12}$	(51 patients)		(no data)	
09~5 TaiNAC Taiwan	4($D_{35}E_{45d1+8}$)$_{q3}$‡ v 4($D_{70}E_{90}$)$_{q3}$‡	$D_{280/12}$ vs $D_{280/12}$	5/20	3/18	0·3	1·7
**(b) subtotal with data §**			**392/ 1397 (28·1%)**	**327/ 1374 (23·8%)**	**32·7**	**165·8**
**Total (a + b)**			**1095/ 4754 (23·0%)**	**1141/ 4762 (24·0%)**	**−18·1**	**513·5**

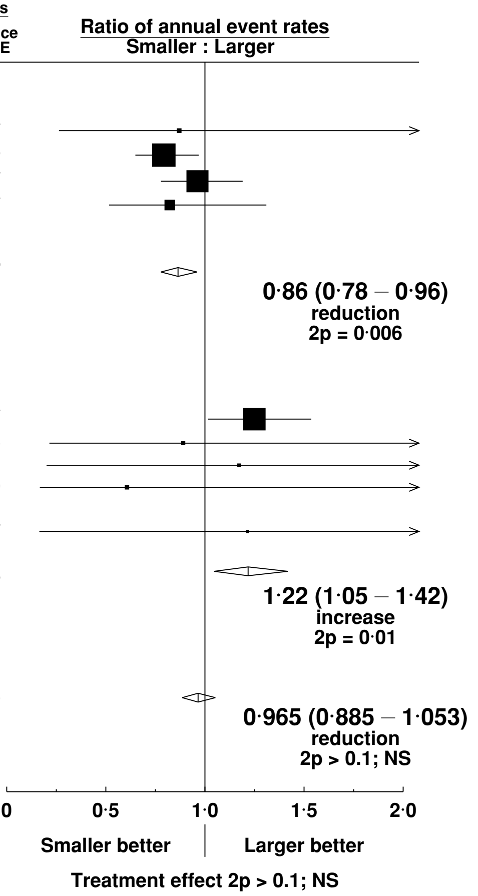

99% or 95% confidence intervals

Difference between treatment effects in 2 subtotals: χ^2_1 = 13·2; 2p = 0·0003

Heterogeneity within subtotals: χ^2_7 = 5·4; p > 0.1; NS

Heterogeneity between 9 trials: χ^2_8 = 18·6; p = 0·02

§ 2 trials with no data do not contribute to subtotals or to the overall total.

‡ Pre-operative chemotherapy
v.v Vice versa
Taxanes: D = docetaxel; P = paclitaxel
Anthracyclines: A = doxorubicin (Adriamycin); E = epirubicin
Other agents: C = cyclophosphamide; F = fluorouracil; M = methotrexate; Cap = capecitabine; Cpt = carboplatin

P29: Any recurrence in trials comparing sequencing order of anthracycline and taxane chemotherapy

Year code and study name	Treatment comparison	Events/Women: Allocated Anth first	Events/Women: Allocated Other first	Anth first events: Logrank O–E	Anth first events: Variance of O–E
**Anthracycline/Taxane sequencing (same regimens, reversing order)**					
00N DFCI 99278	(4A$_{60q2}$;9P$_{80q1}$ v 9P$_{80q1}$;4A$_{60q2}$)‡	*(61 patients)*		*(no data)*	
02K NCC Goyang	(4AC‡;4D$_{75}$Cap$_{2000d1-14}$ v 4D$_{75}$Cap$_{2000d1-14}$‡;4AC)$_{q3}$	24/103	31/106	−3·5	12·9
02U TAC	(4AC‡;4D$_{100}$ v 4D$_{100}$‡;4AC)$_{q3}$	*(120 patients)*		*(no data)*	
03L AERO B−03 France	(4E$_{100}$C;4D$_{100}$ v 4D$_{100}$;4E$_{100}$C)$_{q2}$	0/31	0/34		
03[KBCOG−02 Japan	4E$_{75}$C$_{q3}$‡;12P$_{80q1}$ v 12P$_{80q1}$‡;4E$_{75}$C$_{q3}$	18/87	21/84	−1·6	8·5
04^ OSU Ohio	(4AC;4D$_{75}$ v 4D$_{75}$;4AC)$_{q2}$	*(56 patients)*		*(no data)*	
04{ Monash CC Australia	(4FE$_{100}$C;4D$_{100}$ v 4D$_{100}$;4FE$_{100}$C)$_{q3}$‡	*(69 patients)*		*(no data)*	
05@ UZLeuven Belgium	(3FE$_{100}$C;3D$_{100}$ v 3D$_{100}$;3FE$_{100}$C)$_{q3}$	10/39	9/46	1·8	4·0
05A Neo−tAnGo, UK	(4E$_{90}$C$_{q3}$;4P$_{175}$±Gem$_{2000q2}$ v 4P$_{175}$±Gem$_{2000q2}$;4E$_{90}$C$_{q3}$)‡	158/414	151/414	3·3	68·6
05X H San Carlos, Madrid	(4A$_{75}$‡;4D$_{100}$ v 4D$_{100}$‡;4A$_{75}$)$_{q3}$	*(204 patients)*		*(no data)*	
06N Shiga Japan	(3FE$_{100}$C;3D$_{100}$ v 3D$_{100}$;3FE$_{100}$C)$_{q3}$	*(42 patients)*		*(no data)*	
07Y ACOSOG USA Z1041	(4E$_{75}$C$_{q3}$‡;12P$_{80q1}$‡ v 12P$_{80q1}$‡;4E$_{75}$C$_{q3}$‡)Trz$_{q3}$ to 1yr	18/140	21/142	2·1	7·4
09U JBCRG−10	(4FE$_{100}$C;4D$_{75}$C$_{600}$Trz v 4D$_{75}$C$_{600}$Trz;4FE$_{100}$C)$_{q3}$‡	2/21	2/21	−0·4	0·7
09V JBCRG−09	(3FE$_{100}$C;3D$_{75}$C$_{600}$ v 3D$_{75}$C$_{600}$;3FE$_{100}$C)$_{q3}$‡	9/65	9/63	0·2	4·4
10G SWOG s0800	(6AC$_{q2}$;12NabP$_{100q1}$ v 12NabP$_{100q1}$;6AC$_{q2}$)‡	13/53	13/63	0·6	5·6
10J INC Brazil	(3FA$_{50}$C;3D$_{100}$ v 3D$_{100}$;3FA$_{50}$C)$_{q3}$‡	31/61	18/59	8·1	11·1
**Total §**		**283/ 1014 (27·9%)**	**275/ 1032 (26·6%)**	**10·7**	**123·2**

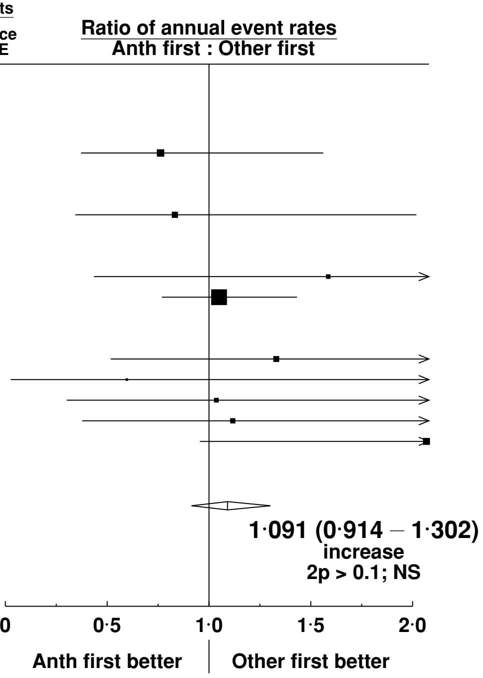

99% or 95% confidence intervals

Heterogeneity between 9 trials: $\chi^2_8 = 8·0$; p > 0.1; NS

Treatment effect 2p > 0.1; NS, adverse

§ 6 trials with no data do not contribute to the total.

‡ Pre−operative chemotherapy
Taxanes: D = docetaxel; P = paclitaxel
Anthracyclines: A = doxorubicin (Adriamycin); E = epirubicin
Other agents: C = cyclophosphamide; F = fluorouracil; M = methotrexate; Trz = trastuzumab; Cap = capecitabine; Gem = gemcitabine; NabP = Nab paclitaxel

P30: Any recurrence in trials of longer versus shorter athracycline/taxane regimens

Year code and study name		Treatment comparison	Cumulative anth dose and difference		Events/Women: Allocated Longer	Events/Women: Allocated Shorter	Longer events: Logrank O–E	Longer events: Variance of O–E	Ratio of annual event rates Longer : Shorter
**(a) Longer versus <6 months of polychemotherapy (anthracycline)**									
$74D_{1+2+4}$	Boston	10 v 5 $A_{45}C_{500}$q3	A_{450} vs A_{225}	A_{225}	95/148	109/151	–6·2	35·9	
$82Z_{2468}$	Metaxas Athens	3$Vcr_{0.9}A_{35}C_{300x4}$q4±7$F_{440x2}A_{25x2}C_{50x15}$q4	A_{455} vs A_{105}	A_{350}	71/158	82/150	–9·1	22·3	
$86P_{1}$	FASG GFEA 01	6 v 3 $F_{500}E_{100}C_{500}$q3	E_{600} vs E	E_{300}	110/212	110/209	–8·9	46·2	
87Q	PRONACAM 87 Argent.	6 v 4 $C_{400}M_{30}F_{400}E_{60}Pr_{40x4}$q3	E_{360} vs E_{240}	E_{120}	*(556 patients)*		*(no data)*		
02B	CALGB 40101	6 v 4 $A_{60}C_{600}$q3	A_{360} vs A_{240}	A_{120}	22/144	20/140	1·5	10·0	
02N	PACS 05 France	6 v 4 $F_{500}E_{100}C_{500}$q3	E_{600} vs E_{300}	E_{300}	88/759	97/756	–6·6	44·7	
$02X_{1}$	CALGB 40101	6 v 4 $A_{60}C_{500}$q2	A_{360} vs A_{240}	A_{120}	54/645	66/655	–6·6	29·2	
04C	NSABP B–36	(6$F_{500}E_{100}C_{500}$ v 4$A_{60}C_{600}$)q3	E_{600} vs A_{240}	E/A_{360}	188/1361	186/1361	1·5	90·4	
**(a) subtotal with data §**					**628/ 3427 (18·3%)**	**670/ 3422 (19·6%)**	**–34·4**	**278·7**	**0·88 (0·79 – 0·99) reduction 2p = 0·04**
**(b) Longer versus <6 months of polychemotherapy (anthracycline and taxane)**									
$97H_{2}$	Multicentre France	6 v 4 $A_{60}P_{200}$q3‡	A_{360} vs A_{240}	A_{120}	*(232 patients)*		*(no data)*		
$01S_{2}$	GeparTrio / GBG 24	8 v 6 $D_{75}A_{50}C_{500}$q3‡	A_{400} vs A_{300}	A_{100}	108/686	136/704	–13·9	55·6	
03%	Chungnam Korea	6 v 3 $E_{75}D_{75}$q3‡	E_{450} vs E_{225}	E_{225}	*(176 patients)*		*(no data)*		
**(b) subtotal with data §**					**108/ 686 (15·7%)**	**136/ 704 (19·3%)**	**–13·9**	**55·6**	**0·78 (0·60 – 1·01) reduction 2p = 0·06**
**Total (a + b)**					**736/ 4113 (17·9%)**	**806/ 4126 (19·5%)**	**–48·3**	**334·3**	**0·866 (0·778 – 0·964) reduction 2p = 0·008**

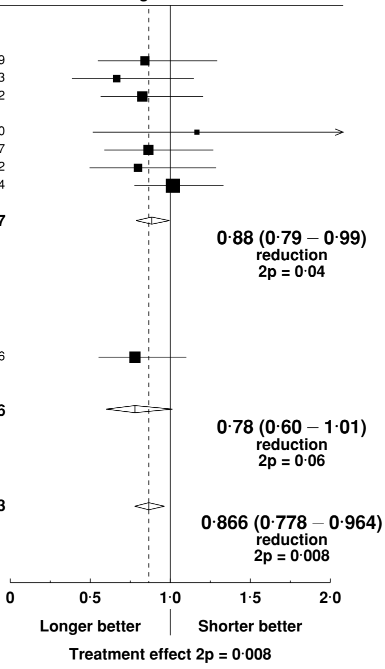

99% or 95% confidence intervals

Difference between treatment effects in 2 subtotals: $\chi^2_1 = 0{\cdot}7$; 2p > 0.1; NS

Heterogeneity within subtotals: $\chi^2_6 = 5{\cdot}0$; p > 0.1; NS

Heterogeneity between 8 trials: $\chi^2_7 = 5{\cdot}7$; p > 0.1; NS

§ 3 trials with no data do not contribute to subtotals or to the overall total.

‡ Pre–operative chemotherapy

Taxanes: D = docetaxel; P = paclitaxel

Anthracyclines: A = doxorubicin (Adriamycin); E = epirubicin

Other agents: C = cyclophosphamide; F = fluorouracil; M = methotrexate; Pr = prednisone; Vcr = vincristine

P31: Any recurrence in trials of higher versus lower anthracycline dose

Year code and study name		Regimens compared and drug doses (mg/m²) per cycle	Cumulative dosages, E/A and difference		Events/Women: Allocated Higher	Events/Women: Adjusted Lower	Higher events: Logrank O–E	Higher events: Variance of O–E	Ratio of annual event rates Higher : Lower
**(a) Increase in anthracycline dose/course and total dose (<100mg A or 150mg E difference)**									
$86P_1$	FASG GFEA 01	$3(FE_{75}C)$ v $3(F_{500}E_{50}C_{500})$	E_{225} vs E_{150}¶	E_{75}	116/200	110/209	0·3	46·9	
$94D_1$	CALGB 9344 *	$4(A_{75/90}$ v $A_{60})C_{600}$	$A_{300/360}$ vs A_{240}	A_{90}	491/1054	2(249/526)	1·0	147·9	
$94D_2$	CALGB 9344 *	$4(A_{75/90}$ v $A_{60})C_{600};4P_{175}$	$A_{300/360}$ vs A_{240}	A_{90}	438/1056	2(226/534)	–3·1	135·2	
05Z	CECOG Austria	$6(FE_{90}C$ v $F_{500}E_{75}C_{500})q2$	E_{540} vs E_{450}¶	E_{90}	*(51 patients)*		*(no data)*		
**(a) subtotal with data §**					**1045/ 2310 (45·2%)**	**1060/ 2329 (45·5%)**	**–1·9**	**330·0**	**0·99 (0·89 – 1·11) reduction 2p > 0.1; NS**
**(b) Increase in anthracycline dose/course and total dose (>100mg A or 150mg E difference)**									
$90C_{3+5}$	FASG GFEA 05	$6(FE_{100}C$ v $F_{500}E_{50}C_{500})$	E_{600} vs E_{300}¶	E_{300}	122/276	144/289	–17·3	54·3	
92N	ICCG C/9/91 UK	$8(FE_{75}C$ v $F_{600}E_{50}C_{600})$	E_{600} vs E_{400}¶	E_{200}	108/391	129/394	–9·9	49·1	
05S	Tianjin Chemo	$6(FE_{100}C$ v $F_{500}E_{60}C_{600})$	E_{600} vs E_{360}¶	E_{240}	*(98 patients)*		*(no data)*		
**(b) subtotal with data §**					**230/ 667 (34·5%)**	**273/ 683 (40·0%)**	**–27·2**	**103·5**	**0·77 (0·63 – 0·93) reduction 2p = 0·008**
**(c) Increase in anthracycline and other drug dose/course and total dose**									
77S	INT Milan 7701	$(6C_{[50-150]}M_{[20-60]}F_{[400-800]};4A_{[50-75]}Vcr$ v $6CMF;4A_{60}Vcr_{1.4})q4$	A_{250} vs A_{240}	A_{10}	45/70	52/70	–0·8	18·6	
85A	CALGB CLB–8541	$4(F_{600}A_{60}C_{600}$ v $F_{300}A_{30}C_{300})q4$	A_{240} vs A_{120}¶	A_{120}	267/528	311/522	–43·3	127·1	
88R	Brussels Belgium	$8(E_{100}C_{830}$ v $E_{60}C_{500})$	E_{800} vs E_{480}¶	E_{320}	121/265	152/272	–22·2	59·2	
91F	MD Anderson	$4(F_{1200}A_{60}C_{1000}$ v $F_{1000}A_{50}C_{500})$‡	A_{240} vs A_{200}¶	A_{40}	*(112 patients)*		*(no data)*		
93@	GFEA Inflammatory	$4(F_{750x4}E_{35x3}C_{400x3}$ v $F_{500}E_{75}C_{500})$‡;4FEC	E_{720} vs E_{600}¶	E_{120}			*(no data)*		
94Q	Austrian BCSG X	$4(E_{60x2}C_{600x2}$ v $E_{60}C_{600});4CMF$	E_{480} vs E_{240}¶	E_{240}	20/56	23/56	–2·9	8·7	
96L	OCSGL Poland	$(6F_{500x2}E_{60x2}C_{75x14})q4$ v $F_{500}E_{50}C_{500})q3$	E_{720} vs E_{300}¶	E_{420}	*(87 patients)*		*(no data)*		
96V	FNCLCC France	$4(F_{1000}E_{100}C_{1000}$ v $F_{500}E_{60}C_{500})$‡	E_{400} vs E_{240}¶	E_{160}	11/42	12/42	–1·1	3·5	
01Q	SBG 2000–1/CEF–60	$6(F_{600}E_{[75-90]}C_{[900-1200]}$ v $F_{600}E_{60}C_{600})$	E_{510} vs E_{360}¶	E_{150}	110/524	125/528	–11·2	54·0	
04=	SBG 2004–1, Sweden	$4E_{[90-120]}Cq2;4D_{[75-100]}q2$ v $4E_{90}C_{600}q2;4D_{75}q2$	E_{435} vs E_{360}	E_{75}	14/42	12/42	–0·3	5·3	
**(c) subtotal with data §**					**588/ 1527 (38·5%)**	**687/ 1532 (44·8%)**	**–81·7**	**276·3**	**0·74 (0·66 – 0·84) reduction 2p < 0·00001**
**Total (a + b + c)**					**1863/ 4504 (41·4%)**	**2020/ 4544 (44·5%)**	**–110·8**	**709·8**	**0·855 (0·795 – 0·921) reduction 2p = 0·00003**

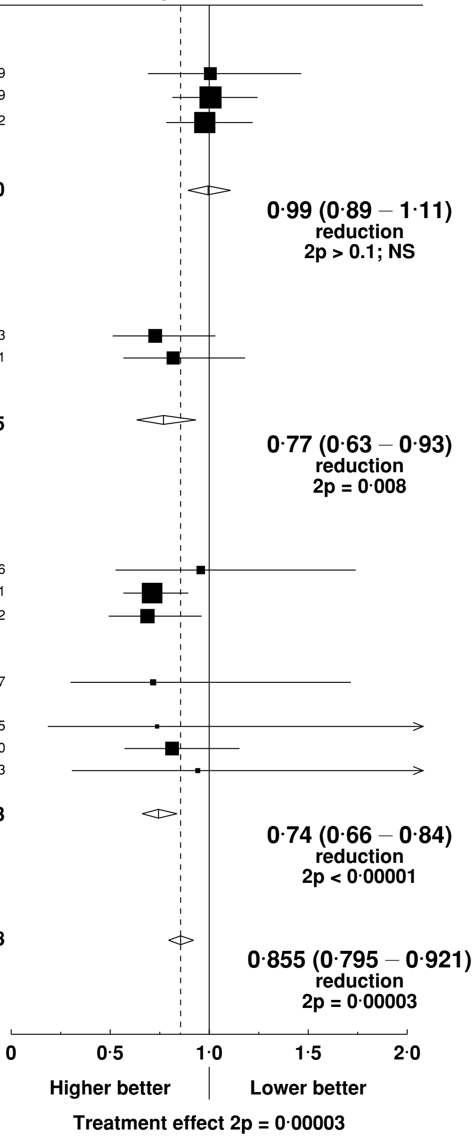

99% or 95% confidence intervals

Heterogeneity between 3 subtotals: $\chi^2_2 = 14·0$; p = 0·0009

Heterogeneity within subtotals: $\chi^2_9 = 2·9$; p > 0.1; NS

Heterogeneity between 12 trials: $\chi^2_{11} = 17·0$; p > 0.1; NS

§ 5 trials with no data do not contribute to subtotals or to the overall total.

* For balance, control patients in 3–way trials or trial strata count half or twice in subtotal(s) and in final total of events/patients.

‡ Pre-operative chemotherapy

¶ Control anthracycline dose less than E90 or A60 per cycle

Taxanes: D = docetaxel; P = paclitaxel. Anthracyclines: A = doxorubicin (Adriamycin); E = Epirubicin.

Other agents: C = cyclophosphamide; F = fluorouracil; M = methotrexate; Vcr = vincristine

All regimens q3week (unless specified as q1, q2 or q4). Semicolon [;] indicates treatment sequence. ×14 means d1-14 po; ×2 (×3) means d1, d8 (d15) iv

77S in control arm dose for CMF are $C_{100}M_{40}F_{600}$

P32: Breast cancer mortality in trials of taxane-plus-anthracycline-based regimen vs same, or more (<doubled or ~doubled) non-taxane cytotoxic chemotherapy

Year code	Study name	Regimens compared and drug doses (mg/m²) per cycle. Any unstated doses are as in the controls	Cumulative doses of taxane & anthracycline	Anthracycline difference	Deaths/Women: Allocated taxane	Deaths/Women: Allocated non-tax.	Taxane deaths*: Logrank O−E	Taxane deaths*: Variance of O−E	Ratio of annual death rates Taxane : Non-tax.
**(a) Taxane (D or P)–plus–anthracycline (A or E)–based regimen † vs the SAME non–taxane cytotoxic chemotherapy (ie, unconfounded trials of adding a taxane)**									
94D1	CALGB 9344	4AC; 4P175 vs 4A90C600	†P700 A360 vs A360	0	175/531	196/526	−17·2	84·7	
94D2	CALGB 9344	4AC; 4P175 vs 4A75C600	†P700 A300 vs A300	0	169/525	203/528	−19·9	85·0	
94D3	CALGB 9344	4AC; 4P175 vs 4A60C600	†P700 A240 vs A240	0	194/534	190/526	−2·6	88·4	
95J	NSABP B–27*	4AC‡; 4D100‡ vs 4(A60C600)‡	†D400 A240 vs A240	0	393/1607	418/1608	−11·2	190·7	
95K	NSABP B–28	4AC; 4P225 vs 4A60C600	†P900 A240 vs A240	0	333/1531	353/1529	−13·0	159·5	
98B	Taxit216 Italy	4E; 4D100; 4CMFq4 vs 4E120; 4(C600×2M40×2F600×2)q4	†D400 E480 vs E480	0	50/486	80/486	−15·0	31·0	
99T	GOIM 9902 Italy	4D100; 4EC vs 4E120C600	†D400 E480 vs E480	0	36/376	40/374	−4·9	17·8	
01Z	PEGASE 07 France	4EC‡; 4D75F750x5 vs 4E150C4000‡	†D300 E600 vs E600	0	*(174 patients)*		*(no data)*		
04!	Craiova Dolj	4P200‡; 4EC‡ vs 4E60C600‡	†P800 E240 vs E240	0	*(124 patients)*		*(no data)*		
**(a) subtotal with data §**					**1350/ 5590 (24·2%)**	**1480/ 5577 (26·5%)**	**−83·7**	**657·1**	**0·88 (0·82 – 0·95) reduction 2p = 0·001**
**(b) Taxane–plus–anthracycline–based regimen (taxane courses given alone) † vs MORE (but < doubled) non–taxane cytotoxic chemotherapy**									
95T	HORG Greece	4D100; 4EC vs 6(F700E75C700)	†D400 E300 vs E450¶	E150	73/391	71/397	0·7	32·5	
97R	HE1097 Greece	3Eq2; 3P250q2; 3CMFq2 vs 4(E110)q2; 4(C840M50F840)q2	†P750 E330 vs E440	E110	100/304	101/300	−5·2	43·9	
97V	IBIS 02 Italy	4P175;4E75Vrb25x2 vs 4E100;4(C600x2M40x2F600x2)q4	†P700 E300 vs E400	E100	33/122	32/122	1·3	14·8	
98D1	BIG 02–98	(3A75;3D100 vs 4A75 or 4A60C600); 3(C600x2M40x2F600x2)q4	†D300 A225 vs A270 (240/300)	A45	49/227	56/237	−0·8	24·2	
98D2	BIG 02–98	(3A75;3D100 vs 4A75 or 4A60C600); 3(C600x2M40x2F600x2)q4	†D300 A225 vs A270 (240/300)	A45	63/732	75/731	−6·5	33·2	
99K	GEICAM 9906 Spain	4FEC; 8(P100)q1 vs 6(F600E90C600)	†P800 E360 vs E540	E180	117/614	162/632	−22·2	64·8	
00E	FinHer/FBCG 00–01	3D(80/100); 3FEC vs 3Vrb25x3; 3(F600E60C600)	†D240 E180 vs E180¶	0≠	77/502	102/507	−12·4	41·3	
00F1	MA.21, Canada	6(E120C830)q2; 4P175 vs 6(F500x2E60x2C75x14)q4	†P700 E720 vs E720	0≠	48/285	58/281	−5·2	24·4	
00F2	MA.21, Canada	6(E120C830)q2; 4P175 vs 6(F500x2E60x2C75x14)q4	†P700 E720 vs E720	0≠	56/416	57/420	−0·1	27·3	
00S1	WSG/AGO AM–02	4E90C600; 4D100 vs 6(F500E100C500)	†D400 E360 vs E600	E240	35/811	50/795	−7·5	20·8	
00U	AERO B–2000 France	4FEC; 4P175 vs 6(F500E100C500)	†P700 E400 vs E600	E200	69/420	83/417	−4·2	34·2	
03$	GEICAM 2004–04	12P80q1‡;4FAC‡ vs 6(F500A50C500)‡	†P960 A200 vs A300¶	A100	16/138	16/135	1·2	5·3	
03R	GIM 1 Italy	4E90C; 4D100 vs 6(F600E75C600)	†D400 E360 vs E450¶	E90	*(1636 patients)*		*(no data)*		
03U	GEICAM 2003–02	4FEC; 8(P100)q1 vs 6(F500E50C500)	†P800 E200 vs E300¶	E100	41/951	48/974	−3·6	21·8	
**(b) subtotal with data §**					**777/ 5913 (13·1%)**	**911/ 5948 (15·3%)**	**−64·3**	**388·6**	**0·85 (0·77 – 0·94) reduction 2p = 0·001**
**(c) Taxane–plus–anthracycline–based regimen (taxane given concurrently) © vs MORE (but < doubled) non–taxane cytotoxic chemotherapy**									
95E	LMU Munich Germany	6E60D75 vs 4E90C600;3C600M40F600	©D300 E360 vs E360	0≠	*(36 patients)*		*(no data)*		
96#	H–V Athens	3EP‡;3EP200 vs 3FEC‡;3F600E75C600	©P1200 E450 vs E450¶	0≠	*(70 patients)*		*(no data)*		
96%	Multicentre Germany	4(EP175)q2; 3CMFq2 vs 4(E90C600); 3(C600M40F600)	©P700 E360 vs E360	0≠	39/113	51/115	−8·0	19·8	
96W1	ECTO Italy*	(4A60P200; 4CMFq4) vs 4A75; 4(C600×2M40×2F600×2)q4	©P800 A240 vs A300	A60	45/451	65/453	−11·1	26·2	
96W2	ECTO Italy*	(4A60P200; 4CMFq4)‡ vs **same control patients**	©P800 A240 vs A300	A60	56/451	65/453	−5·3	28·6	
96X	GONO Italy MIG 5	4E90P175 vs 6(F600E60C600)	©P700 E360 vs E360¶	0≠	110/535	116/520	−5·7	52·7	
97H1	*Multicentre France*	*4AP200‡ vs 4A60C600‡*	*©P800 A240 vs A240*	*0≠*	*0/133*	*0/67*			
97L	BCIRG 001 / GEICAM 9703	6D75AC vs 6(F500A50C500)	©D450 A300 vs A300	0≠	159/745	212/746	−29·5	86·6	
98D1	BIG 02–98	(4A50D75 vs 4A75 or 4A60C600); 3(C100x14M40x2F600x2)q4	©D300 A200 vs A270 (240/300)	A70	60/221	56/237	7·2	26·3	
98D2	BIG 02–98	(4A50D75 vs 4A75 or 4A60C600); 3(C100x14M40x2F600x2)q4	©D300 A200 vs A270 (240/300)	A70	77/739	75/731	0·6	36·3	
98T	ECOG EST2197	4AD60 vs 4A60C600	©D240 A240 vs A240	0≠	209/1476	193/1476	5·8	96·9	
99%	GEICAM 9805 / TARGET 0	6D75AC vs 6(F500A50C500)	©D450 A300 vs A300¶	0≠	38/539	36/521	−0·8	17·7	
99A	Anglo–Celtic II	6A50D75‡ vs 6A60C600‡	©D450 A300 vs A360	A60	49/183	58/180	−4·5	24·5	
99N	RAPP–01 France	4A50D75 vs 4A60C600	©D300 A200 vs A240	A40	11/311	16/316	−2·4	6·5	
01E1+3	PACS 04 France	6E75D75 vs 6(F500E100C500)	©D450 E450 vs E600	E150	222/1494	227/1515	−1·4	107·6	
02L	PetrovStPetersb'g	4P200A‡ vs 4F600A60C600‡	©P800 A240 vs A240		*(103 patients)*		*(no data)*		
02T	Craiova Dolj	4E75P175‡ vs 4F500E100C500‡	©P700 E300 vs E400	E100	*(98 patients)*		*(no data)*		
04N3+4	MATADOR/BOOG 05–02	6D75A50C600 vs 6A60C600q2	©D450 A300 vs A360	A60	27/332	29/332	−1·5	12·6	
09~1	TaiNAC Taiwan	4D35x2E45x2‡ vs 4E45x2F2000x2Fol300x2‡	©D280 E360 vs E360	0≠	3/25	4/18	0·1	1·4	
**(c) subtotal with data §**					**1105/ 7748 (14·3%)**	**1203/ 7680 (15·7%)**	**−40·2**	**488·9**	**0·92 (0·84 – 1·01) reduction 2p = 0·07**
**(d) Taxane–plus–anthracycline–based regimen † vs DOUBLED (or ≈ doubled) non–taxane cytotoxic chemotherapy**									
94B	MDA 94–002	4P250‡; 4FAC vs 4FAC‡; 4(F500×2A50C500)	†P1000 A200 vs A400¶	A200	48/239	47/235	3·3	21·0	
96F	*Aberdeen Scotland*	*4VcrAC‡; 4D100 vs 4VcrAC‡; 4(Vcr1·5A50C1000)*	*†D400 A200 vs A400¶*	*A200*	*4/52*	*12/52*	*−4·0*	*3·4*	
97A	DEVA UK	3Eq4; 3D100 vs 6(E50×2)q4	†D300 E300 vs E600	E300	46/406	62/397	−9·9	21·0	
97J	PACS 01 France	3FEC; 3D100 vs 6(F500E100C500)	†D300 E300 vs E600	E300	156/1003	203/996	−21·8	83·9	
00F1	MA.21, Canada	4A60C600; 4P175 vs 6(F500x2E60x2C75x14)q4	†P700 A240 vs E720	E720–A240	76/290	58/281	10·2	29·6	
00F2	MA.21, Canada	4A60C600; 4P175 vs 6(F500x2E60x2C75x14)q4	†P700 A240 vs E720	E720–A240	70/412	57/420	7·6	30·8	
01F	TACT UK	4FEC; 4D100 vs 8(F600E60C600)	†D400 E240 vs E480¶	E240	276/1258	283/1265	2·7	128·1	
01G1+2	TACT UK	4(F600E60C600); 4D100 vs 4E100; 4(C600×2M40×2F600×2)q4	†D400 E240 vs E400	E160	186/815	198/824	−2·6	88·7	
01J	EORTC10994/BIG 0–01	3D100‡;3E90D75‡ vs 6F500E100C500‡	†D300 E270 vs E600	E330	158/928	174/928	−4·9	71·5	
01T	LMU Munich ADEBAR	4E90C600; 4D100 vs 6(F500x2E60x2C75x14)q4	†D400 E360 vs E720	E360	121/688	109/675	3·7	52·3	
02D	GBG 42 / NNBC 3–Eur.	3FEC; 3D100 vs 6(F500E100C500)	†D300 E300 vs E600	E300	20/1333	24/1327	−1·8	10·7	
02G	Bari Italy	(4EVrb)‡;(4D100 vs 4E120Vrb25x2)	†D400 E480 vs E960	E480			*(no data)*		
07"	Kolkata MC India	3AC;3P175 vs 6A60C600	†P525 A180 vs A360	A180	*(50 patients)*		*(no data)*		
**(d) subtotal with data §**					**1161/ 7424 (15·6%)**	**1227/ 7400 (16·6%)**	**−17·6**	**541·2**	**0·97 (0·89 – 1·05) reduction 2p > 0.1; NS**
**Total (a + b + c + d)**					**4393/ 26675 (16·5%)**	**4821/ 26605 (18·1%)**	**−205·9**	**2075·8**	**0·906 (0·867 – 0·945) reduction 2p < 0·00001**

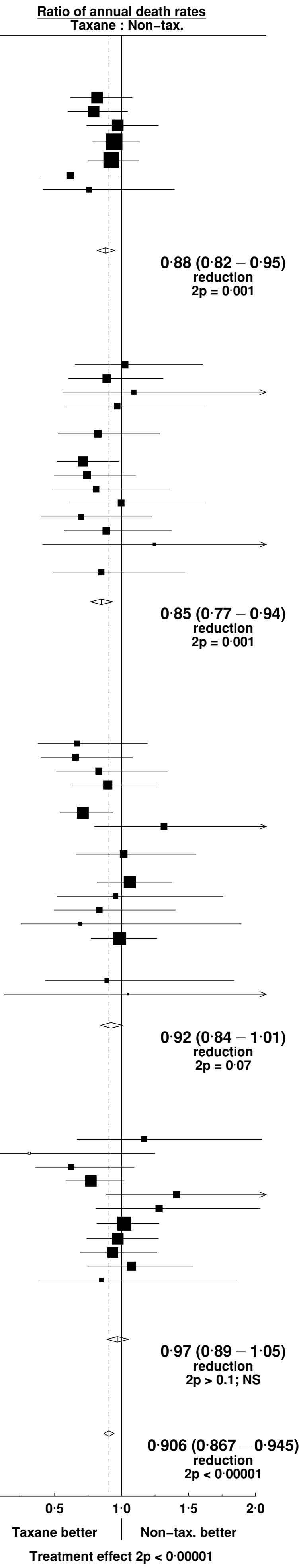

■ 99% or ◇ 95% confidence intervals

Heterogeneity between 4 subtotals: $\chi^2_3 = 4·8$; p > 0.1; NS

Heterogeneity within subtotals: $\chi^2_{35} = 47·3$; p = 0·08

Heterogeneity between 39 trials: $\chi^2_{38} = 52·1$; p = 0·06

* For 3–way trials versus the same control patients
† Taxane courses do not overlap with any other chemotherapy courses; hence, total chemotherapy duration is increased
‡ Pre–operative chemotherapy
© Concurrent treatment with taxane and anthracycline; total chemotherapy duration and number of courses not increased
≠ Same cumulative anthracycline dose, but differences in other drugs
¶ Control anthracycline dose less than E90 or A60 per cycle
Taxanes: D = docetaxel; P = paclitaxel. Anthracyclines: A = doxorubicin (Adriamycin); E = Epirubicin. Other agents: C = cyclophosphamide; F = fluorouracil; Fol = folinic acid; M = methotrexate; Vcr = vincristine; Vrb = vinorelbine
(Not shown: G–CSF, erythropoietin, trastuzumab, antibiotic, hormonal, local or steroid therapies)
All regimens q3week (unless specified as q1, q2 or q4). Semicolon [;] indicates treatment sequence. ×14 means d1–14 po; ×2 (×3) means d1, d8 (d15) iv
03$ at discretion local investigators could have used epirubicin 100mg/m2 instead of doxorubicin 50mg/m2

P33: Death without recurrence in trials of taxane-plus-anthracycline-based regimen vs same, or more (<doubled or ~doubled) non-taxane cytotoxic chemotherapy

Year code and study name	Regimens compared and drug doses (mg/m²) per cycle. Any unstated doses are as in the controls	Cumulative doses of taxane & anthracycline	Anthra-cycline difference	Deaths/Women: Allocated taxane	Deaths/Women: Allocated non-tax.	Taxane deaths*: Logrank O–E	Taxane deaths*: Variance of O–E	Ratio of annual death rates Taxane : Non-tax.
**(a) Taxane (D or P)–plus–anthracycline (A or E)–based regimen † vs the SAME non–taxane cytotoxic chemotherapy (ie, unconfounded trials of adding a taxane)**								
94D1 CALGB 9344	4AC; 4P175 vs 4A90C600	†P700 A360 vs A360	0	35/531	40/526	–2·9	17·3	
94D2 CALGB 9344	4AC; 4P175 vs 4A75C600	†P700 A300 vs A300	0	36/525	34/528	–2·4	15·8	
94D3 CALGB 9344	4AC; 4P175 vs 4A60C600	†P700 A240 vs A240	0	33/534	35/526	–3·7	15·5	
95J NSABP B–27*	4AC‡; 4D100‡ vs 4(A60C600)‡	†D400 A240 vs A240	0	78/1607	70/1608	0·5	35·6	
95K NSABP B–28	4AC; 4P225 vs 4A60C600	†P900 A240 vs A240	0	59/1531	64/1529	–3·8	29·9	
98B Taxit216 Italy	4E; 4D100; 4CMFq4 vs 4E120; 4(C600×2M40×2F600×2)q4	†D400 E480 vs E480	0	7/486	5/486	0·9	3·0	
99T GOIM 9902 Italy	4D100; 4EC vs 4E120C600	†D400 E480 vs E480	0	0/376	1/374	–0·5	0·2	
01Z PEGASE 07 France	4EC‡; 4D75F750x5 vs 4E150C4000‡	†D300 E600 vs E600	0	*(174 patients)*		*(no data)*		
04! Craiova Dolj	4P200‡; 4EC‡ vs 4E60C600‡	†P800 E240 vs E240	0	*(124 patients)*		*(no data)*		
**(a) subtotal with data §**				**248/ 5590 (4·4%)**	**249/ 5577 (4·5%)**	**–11·9**	**117·4**	**0·90 (0·75 – 1·08) reduction 2p > 0.1; NS**
**(b) Taxane–plus–anthracycline–based regimen (taxane courses given alone) † vs MORE (but < doubled) non–taxane cytotoxic chemotherapy**								
95T HORG Greece	4D100; 4EC vs 6(F700E75C700)	†D400 E300 vs E450¶	E150	1/391	4/397	–1·9	0·9	
97R HE1097 Greece	3Eq2; 3P250q2; 3CMFq2 vs 4(E110)q2; 4(C840M50F840)q2	†P750 E330 vs E440	E110	3/304	16/300	–5·5	3·6	
97V IBIS 02 Italy	4P175;4E75Vrb25x2 vs 4E100;4(C600x2M40x2F600x2)q4	†P700 E300 vs E400	E100	5/122	3/122	1·6	1·7	
98D1 BIG 02–98	(3A75;3D100 vs 4A75 or 4A60C600); 3(C600x2M40x2F600x2)q4	†D300 A225 vs A270 (240/300)	A45	1/227	3/237	–0·9	1·0	
98D2 BIG 02–98	(3A75;3D100 vs 4A75 or 4A60C600); 3(C600x2M40x2F600x2)q4	†D300 A225 vs A270 (240/300)	A45	2/732	4/731	–1·4	1·4	
99K GEICAM 9906 Spain	4FEC; 8(P100)q1 vs 6(F600E90C600)	†P800 E360 vs E540	E180	24/614	21/632	1·6	10·5	
00E FinHer/FBCG 00–01	3D(80/100); 3FEC vs 3Vrb25×3; 3(F600E60C600)	†D240 E180 vs E180¶	0≠	9/502	3/507	2·3	2·9	
00F1 MA.21, Canada	6(E120C830)q2; 4P175 vs 6(F500x2E60x2C75x14)q4	†P700 E720 vs E720	0≠	9/285	12/281	–2·3	4·8	
00F2 MA.21, Canada	6(E120C830)q2; 4P175 vs 6(F500x2E60x2C75x14)q4	†P700 E720 vs E720	0≠	11/416	10/420	1·2	5·0	
00S1 WSG/AGO AM–02	4E90C600; 4D100 vs 6(F500E100C500)	†D400 E360 vs E600	E240	5/811	4/795	–0·0	2·0	
00U AERO B–2000 France	4FEC; 4P175 vs 6(F500E100C500)	†P700 E400 vs E600	E200	6/420	7/417	0·0	3·1	
03$ GEICAM 2004–04	12P80q1‡;4FAC‡ vs 6(F500A50C500)‡	†P960 A200 vs A300¶	A100	3/138	6/135	–0·3	1·7	
03R GIM 1 Italy	4E90C; 4D100 vs 6(F600E75C600)	†D400 E360 vs E450¶	E90	*(1636 patients)*		*(no data)*		
03U GEICAM 2003–02	4FEC; 8(P100)q1 vs 6(F500E50C500)	†P800 E200 vs E300¶	E100	12/951	19/974	–4·0	7·7	
**(b) subtotal with data §**				**91/ 5913 (1·5%)**	**112/ 5948 (1·9%)**	**–9·6**	**46·2**	**0·81 (0·61 – 1·08) reduction 2p > 0.1; NS**
**(c) Taxane–plus–anthracycline–based regimen (taxane given concurrently) © vs MORE (but < doubled) non–taxane cytotoxic chemotherapy**								
95E LMU Munich Germany	6E60D75 vs 4E90C600;3C600M40F600	©D300 E360 vs E360	0≠	*(36 patients)*		*(no data)*		
96# H–V Athens	3EP‡;3EP200 vs 3FEC‡;3F600E75C600	©P1200 E450 vs E450¶	0≠	*(70 patients)*		*(no data)*		
96% Multicentre Germany	4(EP175)q2; 3CMFq2 vs 4(E90C600); 3(C600M40F600)	©P700 E360 vs E360	0≠	12/113	8/115	0·5	4·3	
96W1 ECTO Italy*	(4A60P200; 4CMFq4) vs 4A75; 4(C600×2M40×2F600×2)q4	©P800 A240 vs A300	A60	12/451	5/453	3·0	4·1	
96W2 ECTO Italy*	(4A60P200; 4CMFq4)‡ vs **same control patients**	©P800 A240 vs A300	A60	13/451	5/453	3·5	4·4	
96X GONO Italy MIG 5	4E90P175 vs 6(F600E60C600)	©P700 E360 vs E360¶	0≠	52/535	57/520	–2·4	26·4	
97H1 Multicentre France	*4AP200‡ vs 4A60C600‡*	*©P800 A240 vs A240*	*0≠*	*12/133*	*6/67*	*0·0*	*3·7*	
97L BCIRG 001 / GEICAM 9703	6D75AC vs 6(F500A50C500)	©D450 A300 vs A300	0≠	30/745	32/746	–1·1	15·0	
98D1 BIG 02–98	(4A50D75 vs 4A75 or 4A60C600); 3(C100x14M40x2F600x2)q4	©D300 A200 vs A270 (240/300)	A70	2/221	3/237	–0·4	1·2	
98D2 BIG 02–98	(4A50D75 vs 4A75 or 4A60C600); 3(C100x14M40x2F600x2)q4	©D300 A200 vs A270 (240/300)	A70	1/739	4/731	–1·5	1·2	
98T ECOG EST2197	4AD60 vs 4A60C600	©D240 A240 vs A240	0≠	107/1476	120/1476	–6·5	55·0	
99% GEICAM 9805 / TARGET 0	6D75AC vs 6(F500A50C500)	©D450 A300 vs A300¶	0≠	7/539	13/521	–2·8	4·9	
99A Anglo–Celtic II	6A50D75‡ vs 6A60C600‡	©D450 A300 vs A360	A60	0/183	1/180	–0·6	0·2	
99N RAPP–01 France	4A50D75 vs 4A60C600	©D300 A200 vs A240	A40	5/311	2/316	1·5	1·7	
01E1+3 PACS 04 France	6E75D75 vs 6(F500E100C500)	©D450 E450 vs E600	E150	22/1494	25/1515	–1·1	11·3	
02L PetrovStPetersb'g	4P200A‡ vs 4F600A60C600‡	©P800 A240 vs A240		*(103 patients)*		*(no data)*		
02T Craiova Dolj	4E75P175‡ vs 4F500E100C500‡	©P700 E300 vs E400	E100	*(98 patients)*		*(no data)*		
04N3+4 MATADOR/BOOG 05–02	6D75A50C600 vs 6A60C600q2	©D450 A300 vs A360	A60	9/332	12/332	–1·8	5·0	
09~1 TaiNAC Taiwan	4D35x2E45x2‡ vs 4E45x2F2000x2Fol300x2‡	©D280 E360 vs E360	0≠	1/25	0/18	0·4	0·2	
**(c) subtotal with data §**				**285/ 7748 (3·7%)**	**293/ 7680 (3·8%)**	**–15·7**	**130·4**	**0·89 (0·75 – 1·05) reduction 2p > 0.1; NS**
**(d) Taxane–plus–anthracycline–based regimen † vs DOUBLED (or ≈ doubled) non–taxane cytotoxic chemotherapy**								
94B MDA 94–002	4P250‡; 4FAC vs 4FAC‡; 4(F500×2A50C500)	†P1000 A200 vs A400¶	A200	13/239	14/235	–0·5	6·1	
96F Aberdeen Scotland	*4VcrAC‡; 4D100 vs 4VcrAC‡; 4(Vcr1·5A50C1000)*	*†D400 A200 vs A400¶*	*A200*	*0/52*	*0/52*			
97A DEVA UK	3Eq4; 3D100 vs 6(E50×2)q4	†D300 E300 vs E600	E300	6/406	13/397	–4·2	3·9	
97J PACS 01 France	3FEC; 3D100 vs 6(F500E100C500)	†D300 E300 vs E600	E300	13/1003	11/996	0·2	5·9	
00F1 MA.21, Canada	4A60C600; 4P175 vs 6(F500x2E60x2C75x14)q4	†P700 A240 vs E720	E720–A240	7/290	12/281	–2·1	4·1	
00F2 MA.21, Canada	4A60C600; 4P175 vs 6(F500x2E60x2C75x14)q4	†P700 A240 vs E720	E720–A240	6/412	10/420	–1·5	4·0	
01F TACT UK	4FEC; 4D100 vs 8(F600E60C600)	†D400 E240 vs E480¶	E240	25/1258	21/1265	1·8	11·3	
01G1+2 TACT UK	4(F600E60C600); 4D100 vs 4E100; 4(C600×2M40×2F600×2)q4	†D400 E240 vs E400	E160	13/815	15/824	–0·5	6·5	
01J EORTC10994/BIG 0–01	3D100‡;3E90D75‡ vs 6F500E100C500‡	†D300 E270 vs E600	E330	9/928	10/928	1·2	4·0	
01T LMU Munich ADEBAR	4E90C600; 4D100 vs 6(F500x2E60x2C75x14)q4	†D400 E360 vs E720	E360	13/688	22/675	–4·7	8·3	
02D GBG 42 / NNBC 3–Eur.	3FEC; 3D100 vs 6(F500E100C500)	†D300 E300 vs E600	E300	11/1333	12/1327	–0·5	5·7	
02G Bari Italy	(4EVrb)‡;(4D100 vs 4E120Vrb25x2)	†D400 E480 vs E960	E480			*(no data)*		
07" Kolkata MC India	3AC;3P175 vs 6A60C600	†P525 A180 vs A360	A180	*(50 patients)*		*(no data)*		
**(d) subtotal with data §**				**116/ 7424 (1·6%)**	**140/ 7400 (1·9%)**	**–10·8**	**59·9**	**0·83 (0·65 – 1·07) reduction 2p > 0.1; NS**
**Total (a + b + c + d)**				**740/ 26675 (2·8%)**	**794/ 26605 (3·0%)**	**–48·1**	**353·8**	**0·873 (0·787 – 0·969) reduction 2p = 0·01**

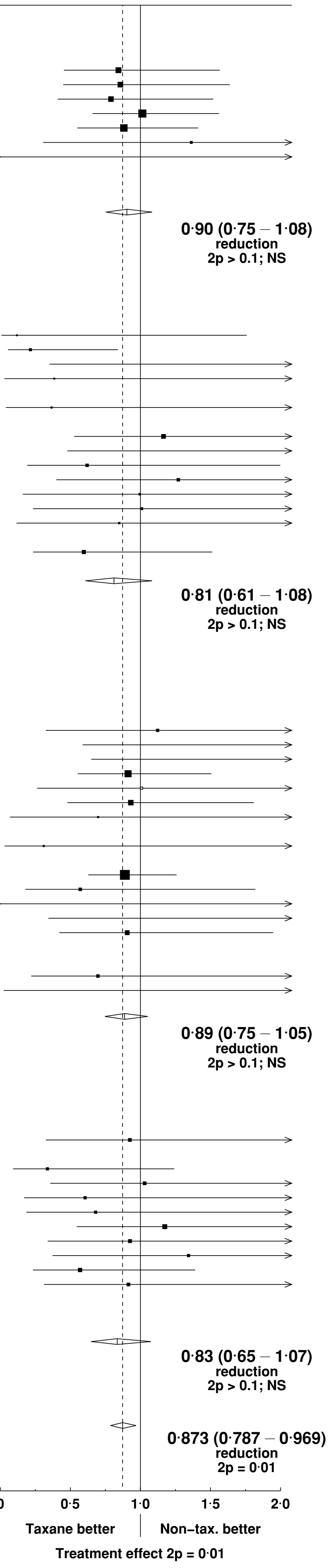

■ 99% or ◇ 95% confidence intervals

Heterogeneity between 4 subtotals: $\chi^2_3 = 0{\cdot}5$; p > 0.1; NS

Heterogeneity within subtotals: $\chi^2_{35} = 33{\cdot}4$; p > 0.1; NS

Heterogeneity between 39 trials: $\chi^2_{38} = 33{\cdot}9$; p > 0.1; NS

* For 3–way trials versus the same control patients

† Taxane courses do not overlap with any other chemotherapy courses; hence, total chemotherapy duration is increased

‡ Pre–operative chemotherapy

© Concurrent treatment with taxane and anthracycline; total chemotherapy duration and number of courses not increased

≠ Same cumulative anthracycline dose, but differences in other drugs

¶ Control anthracycline dose less than E90 or A60 per cycle

Taxanes: D = docetaxel; P = paclitaxel. Anthracyclines: A = doxorubicin (Adriamycin); E = Epirubicin. Other agents: C = cyclophosphamide; F = fluorouracil; Fol = folinic acid; M = methotrexate; Vcr = vincristine; Vrb = vinorelbine

(Not shown: G–CSF, erythropoietin, trastuzumab, antibiotic, hormonal, local or steroid therapies)

All regimens q3week (unless specified as q1, q2 or q4). Semicolon [;] indicates treatment sequence. ×14 means d1–14 po; ×2 (×3) means d1, d8 (d15) iv

03$ at discretion local investigators could have used epirubicin 100mg/m2 instead of doxorubicin 50mg/m2

P34: All-cause mortality in trials of taxane-plus-anthracycline-based regimen vs same, or more (<doubled or ~doubled) non-taxane cytotoxic chemotherapy

Year code	Study name	Regimens compared and drug doses (mg/m²) per cycle. Any unstated doses are as in the controls	Cumulative doses of taxane & anthracycline	Anthra-cycline difference	Deaths/Women: Allocated taxane	Deaths/Women: Allocated non-tax.	Taxane deaths*: Logrank O−E	Taxane deaths*: Variance of O−E	Ratio of annual death rates Taxane : Non-tax.
**(a) Taxane (D or P)-plus-anthracycline (A or E)-based regimen † vs the SAME non-taxane cytotoxic chemotherapy (ie, unconfounded trials of adding a taxane)**									
94D1	CALGB 9344	4AC; 4P175 vs 4A90C600	†P700 A360 vs A360	0	210/531	236/526	−20·1	102·0	
94D2	CALGB 9344	4AC; 4P175 vs 4A75C600	†P700 A300 vs A300	0	205/525	237/528	−22·4	100·8	
94D3	CALGB 9344	4AC; 4P175 vs 4A60C600	†P700 A240 vs A240	0	227/534	225/526	−6·3	103·9	
95J	NSABP B-27*	4AC‡; 4D100‡ vs 4(A60C600)‡	†D400 A240 vs A240	0	471/1607	488/1608	−10·7	226·4	
95K	NSABP B-28	4AC; 4P225 vs 4A60C600	†P900 A240 vs A240	0	392/1531	417/1529	−16·7	189·4	
98B	Taxit216 Italy	4E; 4D100; 4CMFq4 vs 4E120; 4(C600×2M40×2F600×2)q4	†D400 E480 vs E480	0	57/486	85/486	−14·0	34·0	
99T	GOIM 9902 Italy	4D100; 4EC vs 4E120C600	†D400 E480 vs E480	0	36/376	41/374	−5·4	18·0	
01Z	PEGASE 07 France	4EC‡; 4D75F750x5 vs 4E150C4000‡	†D300 E600 vs E600	0	*(174 patients)*		*(no data)*		
04!	Craiova Dolj	4P200‡; 4EC‡ vs 4E60C600‡	†P800 E240 vs E240	0	*(124 patients)*		*(no data)*		
	**(a) subtotal with data §**				**1598/ 5590 (28·6%)**	**1729/ 5577 (31·0%)**	**−95·6**	**774·5**	**0·88 (0·82 − 0·95) reduction 2p = 0·0006**
**(b) Taxane-plus-anthracycline-based regimen (taxane courses given alone) † vs MORE (but < doubled) non-taxane cytotoxic chemotherapy**									
95T	HORG Greece	4D100; 4EC vs 6(F700E75C700)	†D400 E300 vs E450¶	E150	74/391	75/397	−1·2	33·4	
97R	HE1097 Greece	3Eq2; 3P250q2; 3CMFq2 vs 4(E110)q2; 4(C840M50F840)q2	†P750 E330 vs E440	E110	103/304	117/300	−10·7	47·4	
97V	IBIS 02 Italy	4P175;4E75Vrb25x2 vs 4E100;4(C600x2M40x2F600x2)q4	†P700 E300 vs E400	E100	38/122	35/122	2·9	16·5	
98D1	BIG 02−98	(3A75;3D100 vs 4A75 or 4A60C600); 3(C600x2M40x2F600x2)q4	†D300 A225 vs A270 (240/300)	A45	50/227	59/237	−1·7	25·2	
98D2	BIG 02−98	(3A75;3D100 vs 4A75 or 4A60C600); 3(C600x2M40x2F600x2)q4	†D300 A225 vs A270 (240/300)	A45	65/732	79/731	−7·9	34·6	
99K	GEICAM 9906 Spain	4FEC; 8(P100)q1 vs 6(F600E90C600)	†P800 E360 vs E540	E180	141/614	183/632	−20·6	75·3	
00E	FinHer/FBCG 00−01	3D(80/100); 3FEC vs 3Vrb25x3; 3(F600E60C600)	†D240 E180 vs E180¶	0≠	86/502	105/507	−10·1	44·2	
00F1	MA.21, Canada	6(E120C830)q2; 4P175 vs 6(F500x2E60x2C75x14)q4	†P700 E720 vs E720	0≠	57/285	70/281	−7·5	29·3	
00F2	MA.21, Canada	6(E120C830)q2; 4P175 vs 6(F500x2E60x2C75x14)q4	†P700 E720 vs E720	0≠	67/416	67/420	1·1	32·2	
00S1	WSG/AGO AM−02	4E90C600; 4D100 vs 6(F500E100C500)	†D400 E360 vs E600	E240	40/811	54/795	−7·5	22·8	
00U	AERO B−2000 France	4FEC; 4P175 vs 6(F500E100C500)	†P700 E400 vs E600	E200	75/420	90/417	−4·1	37·3	
03$	GEICAM 2004−04	12P80q1‡;4FAC‡ vs 6(F500A50C500)‡	†P960 A200 vs A300¶	A100	19/138	22/135	0·9	7·0	
03R	GIM 1 Italy	4E90C; 4D100 vs 6(F600E75C600)	†D400 E360 vs E450¶	E90	*(1636 patients)*		*(no data)*		
03U	GEICAM 2003−02	4FEC; 8(P100)q1 vs 6(F500E50C500)	†P800 E200 vs E300¶	E100	53/951	67/974	−7·5	29·4	
	**(b) subtotal with data §**				**868/ 5913 (14·7%)**	**1023/ 5948 (17·2%)**	**−74·0**	**434·7**	**0·84 (0·77 − 0·93) reduction 2p = 0·0004**
**(c) Taxane-plus-anthracycline-based regimen (taxane given concurrently) © vs MORE (but < doubled) non-taxane cytotoxic chemotherapy**									
95E	LMU Munich Germany	6E60D75 vs 4E90C600;3C600M40F600	©D300 E360 vs E360	0≠	*(36 patients)*		*(no data)*		
96#	H−V Athens	3EP‡;3EP200 vs 3FEC‡;3F600E75C600	©P1200 E450 vs E450¶	0≠	*(70 patients)*		*(no data)*		
96%	Multicentre Germany	4(EP175)q2; 3CMFq2 vs 4(E90C600); 3(C600M40F600)	©P700 E360 vs E360	0≠	51/113	59/115	−7·5	24·1	
96W1	ECTO Italy*	(4A60P200; 4CMFq4) vs 4A75; 4(C600×2M40×2F600×2)q4	©P800 A240 vs A300	A60	57/451	70/453	−8·1	30·3	
96W2	ECTO Italy*	(4A60P200; 4CMFq4)‡ vs **same control patients**	©P800 A240 vs A300	A60	69/451	70/453	−1·8	33·0	
96X	GONO Italy MIG 5	4E90P175 vs 6(F600E60C600)	©P700 E360 vs E360¶	0≠	162/535	173/520	−8·2	79·1	
97H1	*Multicentre France*	*4AP200‡ vs 4A60C600‡*	*©P800 A240 vs A240*	*0≠*	*12/133*	*6/67*	*0·0*	*3·7*	
97L	BCIRG 001 / GEICAM 9703	6D75AC vs 6(F500A50C500)	©D450 A300 vs A300	0≠	189/745	244/746	−30·6	101·6	
98D1	BIG 02−98	(4A50D75 vs 4A75 or 4A60C600); 3(C100x14M40x2F600x2)q4	©D300 A200 vs A270 (240/300)	A70	62/221	59/237	6·8	27·5	
98D2	BIG 02−98	(4A50D75 vs 4A75 or 4A60C600); 3(C100x14M40x2F600x2)q4	©D300 A200 vs A270 (240/300)	A70	78/739	79/731	−0·9	37·5	
98T	ECOG EST2197	4AD60 vs 4A60C600	©D240 A240 vs A240	0≠	316/1476	313/1476	−0·7	151·9	
99%	GEICAM 9805 / TARGET 0	6D75AC vs 6(F500A50C500)	©D450 A300 vs A300¶	0≠	45/539	49/521	−3·6	22·7	
99A	Anglo−Celtic II	6A50D75‡ vs 6A60C600‡	©D450 A300 vs A360	A60	49/183	59/180	−5·0	24·8	
99N	RAPP−01 France	4A50D75 vs 4A60C600	©D300 A200 vs A240	A40	16/311	18/316	−0·9	8·3	
01E1+3	PACS 04 France	6E75D75 vs 6(F500E100C500)	©D450 E450 vs E600	E150	244/1494	252/1515	−2·6	118·9	
02L	PetrovStPetersb'g	4P200A‡ vs 4F600A60C600‡	©P800 A240 vs A240		*(103 patients)*		*(no data)*		
02T	Craiova Dolj	4E75P175‡ vs 4F500E100C500‡	©P700 E300 vs E400	E100	*(98 patients)*		*(no data)*		
04N3+4	MATADOR/BOOG 05−02	6D75A50C600 vs 6A60C600q2	©D450 A300 vs A360	A60	36/332	41/332	−3·3	17·6	
09~1	TaiNAC Taiwan	4D35x2E45x2‡ vs 4E45x2F2000x2Fol300x2‡	©D280 E360 vs E360	0≠	4/25	4/18	0·4	1·6	
	**(c) subtotal with data §**				**1390/ 7748 (17·9%)**	**1496/ 7680 (19·5%)**	**−55·9**	**619·3**	**0·91 (0·84 − 0·99) reduction 2p = 0·02**
**(d) Taxane-plus-anthracycline-based regimen † vs DOUBLED (or ≈ doubled) non-taxane cytotoxic chemotherapy**									
94B	MDA 94−002	4P250‡; 4FAC vs 4FAC‡; 4(F500×2A50C500)	†P1000 A200 vs A400¶	A200	61/239	61/235	2·8	27·1	
96F	*Aberdeen Scotland*	*4VcrAC‡; 4D100 vs 4VcrAC‡; 4(Vcr1·5A50C1000)*	*†D400 A200 vs A400¶*	*A200*	*4/52*	*12/52*	*−4·0*	*3·4*	
97A	DEVA UK	3Eq4; 3D100 vs 6(E50×2)q4	†D300 E300 vs E600	E300	52/406	75/397	−14·2	24·9	
97J	PACS 01 France	3FEC; 3D100 vs 6(F500E100C500)	†D300 E300 vs E600	E300	169/1003	214/996	−21·7	89·8	
00F1	MA.21, Canada	4A60C600; 4P175 vs 6(F500x2E60x2C75x14)q4	†P700 A240 vs E720	E720−A240	83/290	70/281	8·1	33·8	
00F2	MA.21, Canada	4A60C600; 4P175 vs 6(F500x2E60x2C75x14)q4	†P700 A240 vs E720	E720−A240	76/412	67/420	6·1	34·7	
01F	TACT UK	4FEC; 4D100 vs 8(F600E60C600)	†D400 E240 vs E480¶	E240	301/1258	304/1265	4·5	139·4	
01G1+2	TACT UK	4(F600E60C600); 4D100 vs 4E100; 4(C600×2M40×2F600×2)q4	†D400 E240 vs E400	E160	199/815	213/824	−3·1	95·2	
01J	EORTC10994/BIG 0−01	3D100‡;3E90D75‡ vs 6F500E100C500‡	†D300 E270 vs E600	E330	167/928	184/928	−3·7	75·6	
01T	LMU Munich ADEBAR	4E90C600; 4D100 vs 6(F500x2E60x2C75x14)q4	†D400 E360 vs E720	E360	134/688	131/675	−1·0	60·7	
02D	GBG 42 / NNBC 3−Eur.	3FEC; 3D100 vs 6(F500E100C500)	†D300 E300 vs E600	E300	31/1333	36/1327	−2·3	16·5	
02G	Bari Italy	(4EVrb)‡;(4D100 vs 4E120Vrb25x2)	†D400 E480 vs E960	E480			*(no data)*		
07"	Kolkata MC India	3AC;3P175 vs 6A60C600	†P525 A180 vs A360	A180	*(50 patients)*		*(no data)*		
	**(d) subtotal with data §**				**1277/ 7424 (17·2%)**	**1367/ 7400 (18·5%)**	**−28·4**	**601·1**	**0·95 (0·88 − 1·03) reduction 2p > 0.1; NS**
	**Total (a + b + c + d)**				**5133/ 26675 (19·2%)**	**5615/ 26605 (21·1%)**	**−254·0**	**2429·6**	**0·901 (0·866 − 0·937) reduction 2p < 0·00001**

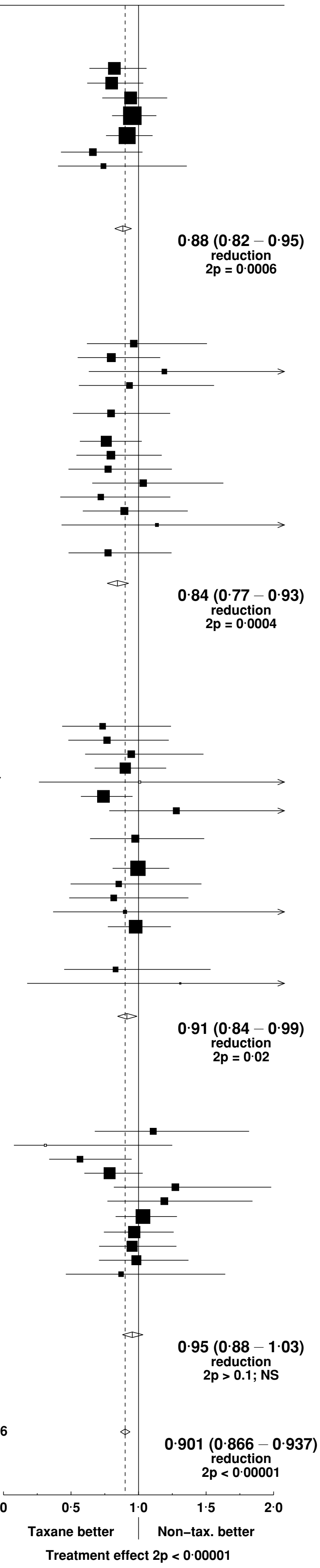

■ 99% or ◇ 95% confidence intervals

Heterogeneity between 4 subtotals: $\chi^2_3 = 4·2$; p > 0.1; NS

Heterogeneity within subtotals: $\chi^2_{36} = 42·0$; p > 0.1; NS

Heterogeneity between 40 trials: $\chi^2_{39} = 46·2$; p > 0.1; NS

* For 3−way trials versus the same control patients
† Taxane courses do not overlap with any other chemotherapy courses; hence, total chemotherapy duration is increased
‡ Pre−operative chemotherapy
© Concurrent treatment with taxane and anthracycline; total chemotherapy duration and number of courses not increased
≠ Same cumulative anthracycline dose, but differences in other drugs
¶ Control anthracycline dose less than E90 or A60 per cycle
Taxanes: D = docetaxel; P = paclitaxel. Anthracyclines: A = doxorubicin (Adriamycin); E = Epirubicin. Other agents: C = cyclophosphamide; F = fluorouracil; Fol = folinic acid; M = methotrexate; Vcr = vincristine; Vrb = vinorelbine
(Not shown: G−CSF, erythropoietin, trastuzumab, antibiotic, hormonal, local or steroid therapies)
All regimens q3week (unless specified as q1, q2 or q4). Semicolon [;] indicates treatment sequence. ×14 means d1−14 po; ×2 (×3) means d1, d8 (d15) iv
03$ at discretion local investigators could have used epirubicin 100mg/m2 instead of doxorubicin 50mg/m2

P35: Breast cancer mortality in trials of taxane-based regimens versus anthracyline-based regimens

Year code	Study name	Treatment comparison	Cumulative taxane and antracycline doses	Deaths/Women: Allocated Taxane	Deaths/Women: Allocated Anth	Taxane deaths: Logrank O−E	Taxane deaths: Variance of O−E	Ratio of annual death rates Taxane : Anth
**(a) Docetaxel vs Anthracycline**								
97N	USO 97−35	$4(\mathbf{D}_{75}\mathbf{C}_{600}$ v $\mathbf{AC})_{q3}$	$\mathbf{D}_{300}$ vs $\mathbf{A}_{240}$	47/506	53/510	−2·5	22·5	
$06Z_1$	MINDACT:EORTC10041	$6(\mathbf{D}_{75}\mathbf{Cap}_{1650x14})_{q3}$ v $6\mathbf{F(E/A)C}_{q3/q4}$	$\mathbf{D}_{450}$ vs $\mathbf{E}_{600}/\mathbf{A}_{300}$	13/454	15/455	−1·8	6·7	
08)	NCC Singapore	neo$4(\mathbf{D}_{75}\mathbf{C}_{600}$ v $\mathbf{AC})_{q3}$	$\mathbf{D}_{300}$ vs $\mathbf{A}_{240}$	*(20 patients)*		*(no data)*		
08Y	Duke Univ USA	neo$4(\mathbf{D}_{75}\mathbf{C}_{600}$ v $\mathbf{AC})_{q3}$	$\mathbf{D}_{300}$ vs $\mathbf{A}_{240}$	*(56 patients)*		*(no data)*		
■ **(a) subtotal with data §**				**60/ 960 (6·2%)**	**68/ 965 (7·0%)**	**−4·3**	**29·2**	**0·86 (0·60 – 1·24) reduction 2p > 0.1; NS**
**(b) Paclitaxel vs Anthracycline**								
97[	Epi−Tax trial Norway	$4(\mathbf{P}_{200}$ v $\mathbf{E}_{90})_{q3}$‡	$\mathbf{P}_{800}$ vs $\mathbf{E}_{360}$	59/114	47/109	5·5	22·7	
02B	CALGB 40101	$12/18\mathbf{P}_{80q1}$ v $4/6\mathbf{AC}_{q3}$	$\mathbf{P}_{960/1440}$ vs $\mathbf{A}_{240/360}$	24/287	18/284	3·2	9·9	
02X	CALGB 40101	$(4/6\mathbf{P}_{175}$ v $4/6\mathbf{AC})_{q2}$	$\mathbf{P}_{700/1050}$ vs $\mathbf{A}_{240/360}$	82/1653	54/1647	13·4	32·5	
■ **(b) subtotal**				**165/ 2054 (8·0%)**	**119/ 2040 (5·8%)**	**22·1**	**65·1**	**1·40 (1·10 – 1·79) increase 2p = 0·006**
■ **Total (a + b)**				**225/ 3014 (7·5%)**	**187/ 3005 (6·2%)**	**17·8**	**94·2**	**1·207 (0·987 – 1·478) increase 2p = 0·07**

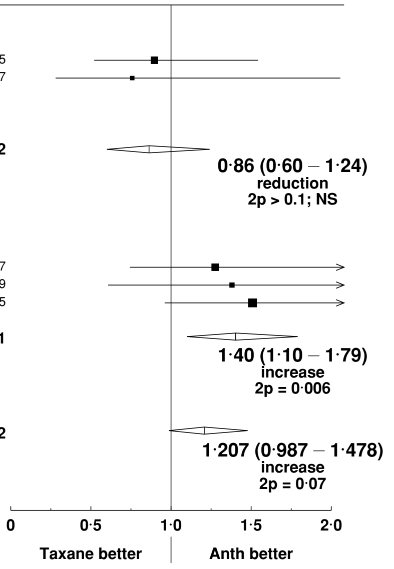

-■- 99% or <> 95% confidence intervals

Difference between treatment effects in 2 subtotals: $\chi^2_1 = 4{\cdot}8$; 2p = 0·03

Heterogeneity within subtotals: $\chi^2_3 = 0{\cdot}5$; p > 0.1; NS

Heterogeneity between 5 trials: $\chi^2_4 = 5{\cdot}3$; p > 0.1; NS

Treatment effect 2p = 0·07, adverse

§ 2 trials with no data do not contribute to subtotals or to the overall total.

‡ Pre−operative chemotherapy

Taxanes: D = docetaxel; P = paclitaxel

Anthracyclines: A = doxorubicin (Adriamycin); E = epirubicin

Other agents: C = cyclophosphamide; F = fluorouracil; Cap = capecitabine

P36: Death without recurrence in trials of taxane-based regimens versus anthracyline-based regimens

Year code and study name	Treatment comparison	Cumulative taxane and antracycline doses	Deaths/Women: Allocated Taxane	Deaths/Women: Allocated Anth	Taxane deaths: Logrank O–E	Taxane deaths: Variance of O–E
**(a) Docetaxel vs Anthracycline**						
97N USO 97–35	4(**D**$_{75}$**C**$_{600}$ v **AC**)$_{q3}$	**D**$_{300}$ vs **A**$_{240}$	8/506	20/510	–6·4	6·2
06Z1 MINDACT:EORTC10041	6(**D**$_{75}$**Cap**$_{1650x14}$)$_{q3}$ v 6**F(E/A)C**$_{q3/q4}$	**D**$_{450}$ vs **E**$_{600}$/**A**$_{300}$	6/454	11/455	–2·0	4·0
08) NCC Singapore	neo4(**D**$_{75}$**C**$_{600}$ v **AC**)$_{q3}$	**D**$_{300}$ vs **A**$_{240}$	*(20 patients)*		*(no data)*	
08Y Duke Univ USA	neo4(**D**$_{75}$**C**$_{600}$ v **AC**)$_{q3}$	**D**$_{300}$ vs **A**$_{240}$	*(56 patients)*		*(no data)*	
**(a) subtotal with data §**			**14/ 960 (1·5%)**	**31/ 965 (3·2%)**	**–8·4**	**10·2**
**(b) Paclitaxel vs Anthracycline**						
97[Epi–Tax trial Norway	4(**P**$_{200}$ v **E**$_{90}$)$_{q3}$‡	**P**$_{800}$ vs **E**$_{360}$	6/114	3/109	1·4	1·9
02B CALGB 40101	12/18**P**$_{80q1}$ v 4/6**AC**$_{q3}$	**P**$_{960/1440}$ vs **A**$_{240/360}$	9/287	8/284	1·0	4·2
02X CALGB 40101	(4/6**P**$_{175}$ v 4/6**AC**)$_{q2}$	**P**$_{700/1050}$ vs **A**$_{240/360}$	28/1653	32/1647	–1·6	14·6
**(b) subtotal**			**43/ 2054 (2·1%)**	**43/ 2040 (2·1%)**	**0·9**	**20·7**
**Total (a + b)**			**57/ 3014 (1·9%)**	**74/ 3005 (2·5%)**	**–7·6**	**31·0**

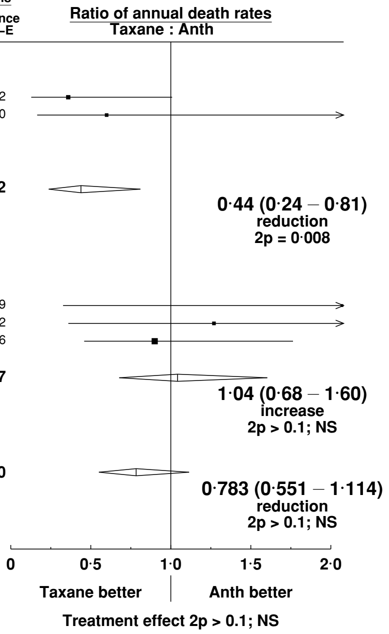

99% or 95% confidence intervals

Difference between treatment effects in 2 subtotals: $\chi^2_1 = 5·1$; 2p = 0·02

Heterogeneity within subtotals: $\chi^2_3 = 2·0$; p > 0.1; NS

Heterogeneity between 5 trials: $\chi^2_4 = 7·2$; p > 0.1; NS

§ 2 trials with no data do not contribute to subtotals or to the overall total.

‡ Pre–operative chemotherapy

Taxanes: D = docetaxel; P = paclitaxel

Anthracyclines: A = doxorubicin (Adriamycin); E = epirubicin

Other agents: C = cyclophosphamide; F = fluorouracil; Cap = capecitabine

P37: All-cause mortality in trials of taxane-based regimens versus anthracyline-based regimens

Year code and study name	Treatment comparison	Cumulative taxane and antracycline doses	Deaths/Women: Allocated Taxane	Deaths/Women: Allocated Anth	Taxane deaths: Logrank O−E	Taxane deaths: Variance of O−E	Ratio of annual death rates Taxane : Anth
**(a) Docetaxel vs Anthracycline**							
97N USO 97−35	4($D_{75}C_{600}$ v AC)$_{q3}$	D_{300} vs A_{240}	55/506	73/510	−8·8	28·7	
06Z1 MINDACT:EORTC10041	6($D_{75}Cap_{1650x14}$)$_{q3}$ v 6$F(E/A)C_{q3/q4}$	D_{450} vs E_{600}/A_{300}	19/454	26/455	−3·9	10·7	
08) NCC Singapore	neo4($D_{75}C_{600}$ v AC)$_{q3}$	D_{300} vs A_{240}	(20 patients)		(no data)		
08Y Duke Univ USA	neo4($D_{75}C_{600}$ v AC)$_{q3}$	D_{300} vs A_{240}	(56 patients)		(no data)		
**(a) subtotal with data §**			**74/ 960 (7·7%)**	**99/ 965 (10·3%)**	**−12·7**	**39·4**	**0·72 (0·53 − 0·99) reduction 2p = 0·04**
**(b) Paclitaxel vs Anthracycline**							
97[Epi−Tax trial Norway	4(P_{200} v E_{90})$_{q3}$‡	P_{800} vs E_{360}	65/114	50/109	6·9	24·6	
02B CALGB 40101	12/18$P_{80 q1}$ v 4/6AC_{q3}	$P_{960/1440}$ vs $A_{240/360}$	33/287	26/284	4·2	14·0	
02X CALGB 40101	(4/6P_{175} v 4/6AC)$_{q2}$	$P_{700/1050}$ vs $A_{240/360}$	110/1653	86/1647	11·8	47·1	
**(b) subtotal**			**208/ 2054 (10·1%)**	**162/ 2040 (7·9%)**	**22·9**	**85·8**	**1·31 (1·06 − 1·61) increase 2p = 0·01**
**Total (a + b)**			**282/ 3014 (9·4%)**	**261/ 3005 (8·7%)**	**10·2**	**125·2**	**1·085 (0·911 − 1·293) increase 2p > 0.1; NS**

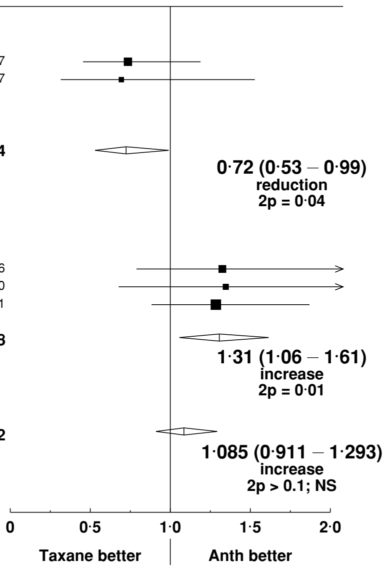

■ 99% or ◇ 95% confidence intervals

Difference between treatment effects in 2 subtotals: $\chi^2_1 = 9·4$; 2p = 0·002

Heterogeneity within subtotals: $\chi^2_3 = 0·1$; p > 0.1; NS

Heterogeneity between 5 trials: $\chi^2_4 = 9·5$; p = 0·05

§ 2 trials with no data do not contribute to subtotals or to the overall total.

‡ Pre−operative chemotherapy
Taxanes: D = docetaxel; P = paclitaxel
Anthracyclines: A = doxorubicin (Adriamycin); E = epirubicin
Other agents: C = cyclophosphamide; F = fluorouracil; Cap = capecitabine

P38: Breast cancer mortality in trials of Docetaxel versus Paclitaxel

Year code and study name	Treatment comparison	Cumulative docetaxel and paclitaxel doses	Deaths/Women: Allocated Docetaxel	Deaths/Women: Allocated Paclitaxel	Docetaxel deaths: Logrank O–E	Docetaxel deaths: Variance of O–E	Ratio of annual death rates Docetaxel : Paclitaxel
**(a) Docetaxel q2 or q3 vs Paclitaxel q2 or q3**							
99V ECOG EST1199	4**AC**; 4(**D**$_{100}$ v **P**$_{175}$)$_{q3}$	**D**$_{400}$ vs **P**$_{700}$	221/1262	271/1272	−21·4	113·4	
00W N–SAS–BC 02 Japan	±4**AC**/**EC**; (4/8**D**$_{75}$ v 4/8**P**$_{175}$)$_{q3}$	**D**$_{300/600}$ vs **P**$_{700/1400}$	73/530	97/530	−14·5	40·7	
04R HORG CT/04.22 Greece	4**FEC**; 4(**D**$_{75}$ v **P**$_{175}$)$_{q2}$	**D**$_{300}$ vs **P**$_{700}$	25/240	27/241	−1·4	11·1	
??K Mansoura Egypt	4**AC**; 4(**D**$_{100}$ v **P**$_{175}$)$_{q3}$	**D**$_{400}$ vs **P**$_{700}$	*(160 patients)*		*(no data)*		
**(a) subtotal with data §**			**319/ 2032 (15·7%)**	**395/ 2043 (19·3%)**	**−37·3**	**165·1**	**0·80 (0·69 – 0·93) reduction 2p = 0·004**
**(b) Docetaxel q1 vs Paclitaxel q1**							
99V ECOG EST1199	4**AC**; 12(**D**$_{35}$ v **P**$_{80}$)$_{q1}$	**D**$_{420}$ vs **P**$_{960}$	259/1260	231/1258	19·7	115·5	
05C HE 10/05 Greece	3**E**$_{110q2}$; 3**CMF**; 9(**D**$_{35}$ v **P**$_{80}$)$_{q1}$	**D**$_{315}$ vs **P**$_{720}$	36/334	46/330	−5·5	19·3	
07' KBCOG–06 Japan	4(**D**$_{75q3}$ v **P**$_{80d1,8,15q4}$)‡; 4**FEC**$_{q3}$‡	**D**$_{300}$ vs **P**$_{240}$	*(50 patients)*		*(no data)*		
**(b) subtotal with data §**			**295/ 1594 (18·5%)**	**277/ 1588 (17·4%)**	**14·1**	**134·9**	**1·11 (0·94 – 1·31) increase 2p > 0.1; NS**
**Total (a + b)**			**614/ 3626 (16·9%)**	**672/ 3631 (18·5%)**	**−23·1**	**300·0**	**0·926 (0·827 – 1·037) reduction 2p > 0.1; NS**

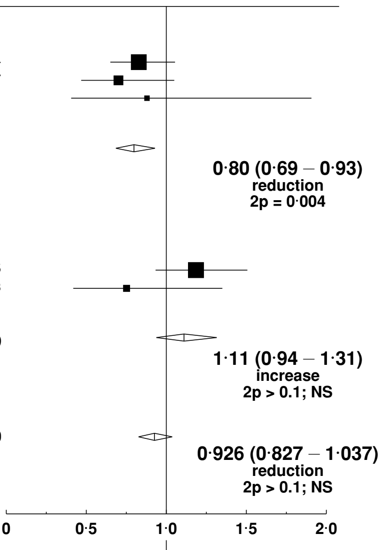

■ 99% or ◇ 95% confidence intervals

Difference between treatment effects in 2 subtotals: $\chi^2_1 = 8{\cdot}1$; **2p = 0·004**

Heterogeneity within subtotals: $\chi^2_3 = 4{\cdot}4$; **p > 0.1; NS**

Heterogeneity between 5 trials: $\chi^2_4 = 12{\cdot}5$; **p = 0·01**

Docetaxel better | Paclitaxel better

Treatment effect 2p > 0.1; NS

§ 2 trials with no data do not contribute to subtotals or to the overall total.

‡ Pre-operative chemotherapy

Taxanes: D = docetaxel; P = paclitaxel

Anthracyclines: A = doxorubicin (Adriamycin); E = epirubicin

Other agents: C = cyclophosphamide; F = fluorouracil

P39: Death without recurrence in trials of Docetaxel versus Paclitaxel

Year code and study name	Treatment comparison	Cumulative docetaxel and paclitaxel doses	Deaths/Women: Allocated Docetaxel	Deaths/Women: Allocated Paclitaxel	Docetaxel deaths: Logrank O–E	Docetaxel deaths: Variance of O–E	Ratio of annual death rates Docetaxel : Paclitaxel
**(a) Docetaxel q2 or q3 vs Paclitaxel q2 or q3**							
99V ECOG EST1199	4**AC**; 4(D_{100} v P_{175})$_{q3}$	D_{400} vs P_{700}	83/1262	73/1272	1·6	37·6	
00W N–SAS–BC 02 Japan	±4**AC**/**EC**; (4/8D_{75} v 4/8P_{175})$_{q3}$	$D_{300/600}$ vs $P_{700/1400}$	6/530	4/530	0·8	2·5	
04R HORG CT/04.22 Greece	4**FEC**; 4(D_{75} v P_{175})$_{q2}$	D_{300} vs P_{700}	0/240	0/241			
??K Mansoura Egypt	4**AC**; 4(D_{100} v P_{175})$_{q3}$	D_{400} vs P_{700}	*(160 patients)*		*(no data)*		
**■ (a) subtotal with data §**			**89/ 2032 (4·4%)**	**77/ 2043 (3·8%)**	**2·4**	**40·0**	**1·06 (0·78 – 1·45) increase 2p > 0.1; NS**
**(b) Docetaxel q1 vs Paclitaxel q1**							
99V ECOG EST1199	4**AC**; 12(D_{35} v P_{80})$_{q1}$	D_{420} vs P_{960}	90/1260	79/1258	3·7	39·7	
05C HE 10/05 Greece	3E_{110q2}; 3**CMF**; 9(D_{35} v P_{80})$_{q1}$	D_{315} vs P_{720}	7/334	8/330	–0·3	3·6	
07' KBCOG–06 Japan	4(D_{75q3} v $P_{80d1,8,15q4}$)‡; 4FEC_{q3}‡	D_{300} vs P_{240}	*(50 patients)*		*(no data)*		
**■ (b) subtotal with data §**			**97/ 1594 (6·1%)**	**87/ 1588 (5·5%)**	**3·4**	**43·3**	**1·08 (0·80 – 1·46) increase 2p > 0.1; NS**
**■ Total (a + b)**			**186/ 3626 (5·1%)**	**164/ 3631 (4·5%)**	**5·8**	**83·3**	**1·072 (0·865 – 1·328) increase 2p > 0.1; NS**

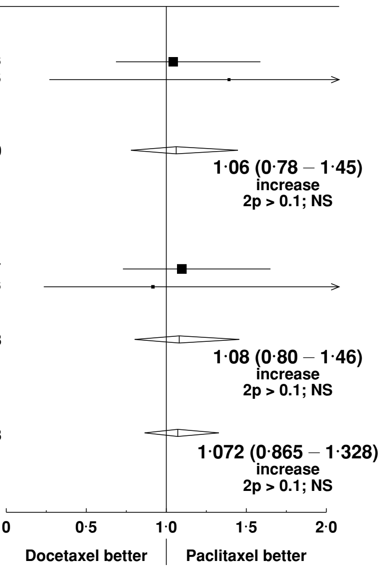

Treatment effect 2p > 0.1; NS, adverse

■ 99% or ◇ 95% confidence intervals

Difference between treatment effects in 2 subtotals: $\chi^2_1 = 0{\cdot}0$; 2p > 0.1; NS

Heterogeneity within subtotals: $\chi^2_2 = 0{\cdot}3$; p > 0.1; NS

Heterogeneity between 4 trials: $\chi^2_3 = 0{\cdot}3$; p > 0.1; NS

§ 2 trials with no data do not contribute to subtotals or to the overall total.

‡ Pre–operative chemotherapy

Taxanes: D = docetaxel; P = paclitaxel

Anthracyclines: A = doxorubicin (Adriamycin); E = epirubicin

Other agents: C = cyclophosphamide; F = fluorouracil

P40: All-cause mortality in trials of Docetaxel versus Paclitaxel

Year code and study name	Treatment comparison	Cumulative docetaxel and paclitaxel doses	Deaths/Women: Allocated Docetaxel	Deaths/Women: Allocated Paclitaxel	Docetaxel deaths: Logrank O–E	Docetaxel deaths: Variance of O–E
**(a) Docetaxel q2 or q3 vs Paclitaxel q2 or q3**						
99V ECOG EST1199	4**AC**; 4(**D**$_{100}$ v **P**$_{175}$)$_{q3}$	**D**$_{400}$ vs **P**$_{700}$	304/1262	344/1272	−19·8	150·9
00W N–SAS–BC 02 Japan	±4**AC**/**EC**; (4/8**D**$_{75}$ v 4/8**P**$_{175}$)$_{q3}$	**D**$_{300/600}$ vs **P**$_{700/1400}$	79/530	101/530	−13·6	43·1
04R HORG CT/04.22 Greece	4**FEC**; 4(**D**$_{75}$ v **P**$_{175}$)$_{q2}$	**D**$_{300}$ vs **P**$_{700}$	25/240	27/241	−1·4	11·1
??K Mansoura Egypt	4**AC**; 4(**D**$_{100}$ v **P**$_{175}$)$_{q3}$	**D**$_{400}$ vs **P**$_{700}$	*(160 patients)*		*(no data)*	
**(a) subtotal with data §**			**408/ 2032 (20·1%)**	**472/ 2043 (23·1%)**	**−34·9**	**205·1**
**(b) Docetaxel q1 vs Paclitaxel q1**						
99V ECOG EST1199	4**AC**; 12(**D**$_{35}$ v **P**$_{80}$)$_{q1}$	**D**$_{420}$ vs **P**$_{960}$	349/1260	310/1258	23·3	155·2
05C HE 10/05 Greece	3**E**$_{110q2}$; 3**CMF**; 9(**D**$_{35}$ v **P**$_{80}$)$_{q1}$	**D**$_{315}$ vs **P**$_{720}$	43/334	54/330	−5·8	22·9
07' KBCOG–06 Japan	4(**D**$_{75q3}$ v **P**$_{80d1,8,15q4}$)‡; 4**FEC**$_{q3}$‡	**D**$_{300}$ vs **P**$_{240}$	*(50 patients)*		*(no data)*	
**(b) subtotal with data §**			**392/ 1594 (24·6%)**	**364/ 1588 (22·9%)**	**17·5**	**178·1**
**Total (a + b)**			**800/ 3626 (22·1%)**	**836/ 3631 (23·0%)**	**−17·4**	**383·3**

Ratio of annual death rates Docetaxel : Paclitaxel

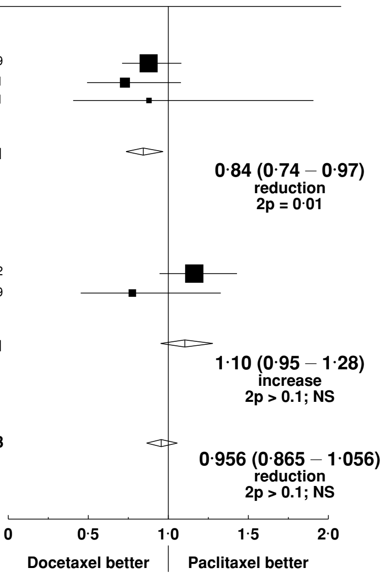

(a) subtotal: **0·84 (0·74 – 0·97) reduction 2p = 0·01**

(b) subtotal: **1·10 (0·95 – 1·28) increase 2p > 0.1; NS**

Total (a + b): **0·956 (0·865 – 1·056) reduction 2p > 0.1; NS**

Treatment effect 2p > 0.1; NS

99% or 95% confidence intervals

Difference between treatment effects in 2 subtotals: χ^2_1 = 6·9; 2p = 0·009

Heterogeneity within subtotals: χ^2_3 = 4·4; p > 0.1; NS

Heterogeneity between 5 trials: χ^2_4 = 11·3; p = 0·02

§ 2 trials with no data do not contribute to subtotals or to the overall total.

‡ Pre–operative chemotherapy
Taxanes: D = docetaxel; P = paclitaxel
Anthracyclines: A = doxorubicin (Adriamycin); E = epirubicin
Other agents: C = cyclophosphamide; F = fluorouracil

P41: Breast cancer mortality in trials comparing similar cumulative dose taxane chemotherapy delivered in smaller vs larger fractions

Year code and study name		Regimens compared and drug doses (mg/m²) per cycle	Difference mg/w	Deaths/Women Allocated Smaller	Deaths/Women Allocated Larger	Smaller deaths Logrank O–E	Smaller deaths Variance of O–E
**(a) Chemotherapy in smaller fractions: Paclitaxel**							
98K123456	MDA 98–240	(12$P_{80/150/175q1}$ v 4P_{225q3}); 4FAC	$P_{720–2100/12}$ vs $P_{900/12}$	6/128	15/126	0·9	1·9
99V	ECOG EST1199	4AC; (12$P_{80\,q1}$ v 4P_{175q3})	$P_{960/12}$ vs $P_{700/12}$	231/1258	271/1272	−21·0	117·6
03*	SWOG S0221	(15($A_{24}C_{60d1–7}$)$_{q1}$/6AC_{q2}); (12P_{80q1} v 6P_{175q2})	$P_{960/12}$ vs $P_{1050/12}$	212/1641	220/1653	−2·8	101·0
05C	HE 10/05 Greece	3E_{110q2}; (3CMF_{q2};9P_{80q1} v 3P_{200q2};3CMF_{q2})	$P_{720/9}$ vs $P_{600/6}$	46/330	53/337	−3·0	23·0
05R	PKU Beijing	CPt_{AUC6}‡ + 4($P_{60d1,8,15}$ v P_{175})$_{q3}$‡	$P_{720/12}$ vs $P_{700/12}$	(220 patients)		(no data)	
**(a) subtotal with data §**				**495/ 3357 (14·7%)**	**559/ 3388 (16·5%)**	**−26·0**	**243·5**
**(b) Chemotherapy in smaller fractions: Docetaxel**							
99V	ECOG EST1199	4AC; (12D_{35q1} v 4D_{100q3})	$D_{420/12}$ vs $D_{400/12}$	259/1260	221/1262	17·8	112·6
00^	U Hull / Lincoln CH	4AC; (12D_{33q1} v 4D_{100q3})	$D_{400/12}$ vs $D_{400/12}$	8/41	6/41	0·3	1·9
04Z	BREAST–10	12$E_{30}D_{35q1}$ v 4$E_{75}D_{80q3}$	$D_{400/12}$ vs $D_{320/12}$	3/22	2/22	0·6	1·1
05@	UZLeuven Belgium	(4$F_{375}E_{75}C_{375}$;4$D_{75/v.v}$)$_{q2}$ v (3$F_{500}E_{100}C_{500}$;3$D_{100/v.v}$)$_{q3}$	$D_{300/8}$ vs $D_{300/9}$	5/54	5/31	0·5	1·7
07e	Emory Taxane trial	8($D_{50}Cap_{2000d1–7}$)$_{q2}$ v 4(D_{100};$Cap_{2000d1–14}$)$_{q3}$	$D_{400/12}$ vs $D_{400/12}$	(51 patients)		(no data)	
09~5	TaiNAC Taiwan	4($D_{35}E_{45d1+8}$)$_{q3}$‡ v 4($D_{70}E_{90}$)$_{q3}$‡	$D_{280/12}$ vs $D_{280/12}$	2/20	1/18	0·1	0·7
**(b) subtotal with data §**				**277/ 1397 (19·8%)**	**235/ 1374 (17·1%)**	**19·3**	**118·0**
**Total (a + b)**				**772/ 4754 (16·2%)**	**794/ 4762 (16·7%)**	**−6·7**	**361·5**

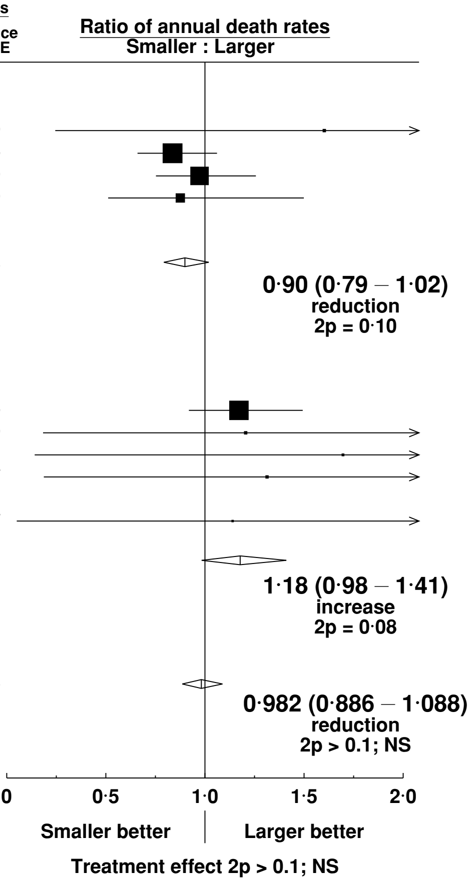

99% or 95% confidence intervals

Difference between treatment effects in 2 subtotals: χ^2_1 = 5·8; 2p = 0·02

Heterogeneity within subtotals: χ^2_7 = 2·0; p > 0.1; NS

Heterogeneity between 9 trials: χ^2_8 = 7·8; p > 0.1; NS

§ 2 trials with no data do not contribute to subtotals or to the overall total.

‡ Pre-operative chemotherapy
v.v Vice versa
Taxanes: D = docetaxel; P = paclitaxel
Anthracyclines: A = doxorubicin (Adriamycin); E = epirubicin
Other agents: C = cyclophosphamide; F = fluorouracil; M = methotrexate; Cap = capecitabine; Cpt = carboplatin

P42: Death without recurrence in trials comparing similar cumulative dose taxane chemotherapy delivered in smaller vs larger fractions

Year code and study name		Regimens compared and drug doses (mg/m²) per cycle	Difference mg/w	Deaths/Women Allocated Smaller	Deaths/Women Allocated Larger	Smaller deaths Logrank O−E	Smaller deaths Variance of O−E	Ratio of annual death rates Smaller : Larger
**(a) Chemotherapy in smaller fractions: Paclitaxel**								
98K123456	MDA 98−240	(12P80/150/175q1 v 4P225q3); 4FAC	P720−2100/12 vs P900/12	1/128	3/126	−0·8	0·9	
99V	ECOG EST1199	4AC; (12P80 q1 v 4P175q3)	P960/12 vs P700/12	79/1258	73/1272	2·1	35·8	
03*	SWOG S0221	(15(A24C60d1−7)q1/6ACq2); (12P80q1 v 6P175q2)	P960/12 vs P1050/12	81/1641	54/1653	13·0	32·4	
05C	HE 10/05 Greece	3E110q2; (3CMFq2;9P80q1 v 3P200q2;3CMFq2)	P720/9 vs P600/6	8/330	10/337	−1·6	4·3	
05R	PKU Beijing	CPtAUC6‡ + 4(P60d1,8,15 v P175)q3‡	P720/12 vs P700/12	(220 patients)		(no data)		
**(a) subtotal with data §**				**169/ 3357 (5·0%)**	**140/ 3388 (4·1%)**	**12·7**	**73·4**	**1·19 (0·95 − 1·49) increase 2p > 0.1; NS**
**(b) Chemotherapy in smaller fractions: Docetaxel**								
99V	ECOG EST1199	4AC; (12D35q1 v 4D100q3)	D420/12 vs D400/12	90/1260	83/1262	4·7	41·2	
00^	U Hull / Lincoln CH	4AC; (12D33q1 v 4D100q3)	D400/12 vs D400/12	0/41	0/41			
04Z	BREAST−10	12E30D35q1 v 4E75D80q3	D400/12 vs D320/12	0/22	1/22	−0·6	0·2	
05@	UZLeuven Belgium	(4F375E75C375;4D75/v.v)q2 v (3F500E100C500;3D100/v.v)q3	D300/8 vs D300/9	1/54	1/31			
07e	Emory Taxane trial	8(D50Cap2000d1−7)q2 v 4(D100;Cap2000d1−14)q3	D400/12 vs D400/12	(51 patients)		(no data)		
09~5	TaiNAC Taiwan	4(D35E45d1+8)q3‡ v 4(D70E90)q3‡	D280/12 vs D280/12	0/20	0/18			
**(b) subtotal with data §**				**91/ 1397 (6·5%)**	**85/ 1374 (6·2%)**	**4·1**	**41·5**	**1·10 (0·81 − 1·50) increase 2p > 0.1; NS**
**Total (a + b)**				**260/ 4754 (5·5%)**	**225/ 4762 (4·7%)**	**16·8**	**114·8**	**1·157 (0·964 − 1·390) increase 2p > 0.1; NS**

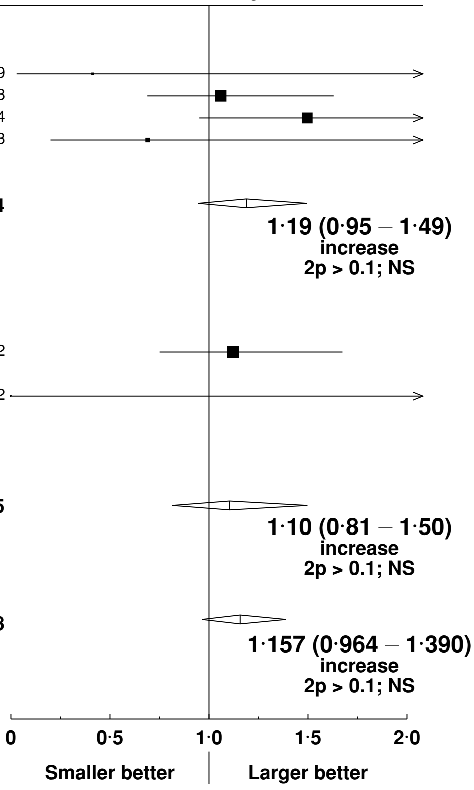

■ 99% or ◇ 95% confidence intervals

Difference between treatment effects in 2 subtotals: χ^2_1 **= 0·1; 2p > 0.1; NS**

Heterogeneity within subtotals: χ^2_4 **= 6·1; p > 0.1; NS**

Heterogeneity between 6 trials: χ^2_5 **= 6·3; p > 0.1; NS**

Treatment effect 2p > 0.1; NS, adverse

§ 2 trials with no data do not contribute to subtotals or to the overall total.

‡ Pre−operative chemotherapy
v.v Vice versa
Taxanes: D = docetaxel; P = paclitaxel
Anthracyclines: A = doxorubicin (Adriamycin); E = epirubicin
Other agents: C = cyclophosphamide; F = fluorouracil; M = methotrexate; Cap = capecitabine; Cpt = carboplatin

P43: All-cause mortality in trials comparing similar cumulative dose taxane chemotherapy delivered in smaller vs larger fractions

Year code and study name		Regimens compared and drug doses (mg/m²) per cycle	Difference mg/w	Deaths/Women: Allocated Smaller	Deaths/Women: Allocated Larger	Smaller deaths: Logrank O–E	Smaller deaths: Variance of O–E
**(a) Chemotherapy in smaller fractions: Paclitaxel**							
$98K_{123456}$	MDA 98–240	$(12P_{80/150/175q1}$ v $4P_{225q3})$; 4FAC	$P_{720–2100/12}$ vs $P_{900/12}$	7/128	18/126	0·1	2·8
99V	ECOG EST1199	4AC; $(12P_{80\,q1}$ v $4P_{175q3})$	$P_{960/12}$ vs $P_{700/12}$	310/1258	344/1272	−18·9	153·4
03*	SWOG S0221	$(15(A_{24}C_{60d1–7})_{q1}/6AC_{q2})$; $(12P_{80q1}$ v $6P_{175q2})$	$P_{960/12}$ vs $P_{1050/12}$	293/1641	274/1653	10·2	133·4
05C	HE 10/05 Greece	$3E_{110q2}$; $(3CMF_{q2};9P_{80q1}$ v $3P_{200q2};3CMF_{q2})$	$P_{720/9}$ vs $P_{600/6}$	54/330	63/337	−4·6	27·3
05R	PKU Beijing	CPt_{AUC6}‡ + $4(P_{60d1,8,15}$ v $P_{175})_{q3}$‡	$P_{720/12}$ vs $P_{700/12}$	(220 patients)		(no data)	
**(a) subtotal with data §**				**664/ 3357 (19·8%)**	**699/ 3388 (20·6%)**	**−13·3**	**316·8**
**(b) Chemotherapy in smaller fractions: Docetaxel**							
99V	ECOG EST1199	4AC; $(12D_{35q1}$ v $4D_{100q3})$	$D_{420/12}$ vs $D_{400/12}$	349/1260	304/1262	22·5	153·8
00^	U Hull / Lincoln CH	4AC; $(12D_{33q1}$ v $4D_{100q3})$	$D_{400/12}$ vs $D_{400/12}$	8/41	6/41	0·3	1·9
04Z	BREAST–10	$12E_{30}D_{35q1}$ v $4E_{75}D_{80q3}$	$D_{400/12}$ vs $D_{320/12}$	3/22	3/22	−0·0	1·3
05@	UZLeuven Belgium	$(4F_{375}E_{75}C_{375};4D_{75/v.v})_{q2}$ v $(3F_{500}E_{100}C_{500};3D_{100/v.v})_{q3}$	$D_{300/8}$ vs $D_{300/9}$	6/54	6/31	0·5	1·7
07e	Emory Taxane trial	$8(D_{50}Cap_{2000d1–7})_{q2}$ v $4(D_{100};Cap_{2000d1–14})_{q3}$	$D_{400/12}$ vs $D_{400/12}$	(51 patients)		(no data)	
$09{\sim}5$	TaiNAC Taiwan	$4(D_{35}E_{45d1+8})_{q3}$‡ v $4(D_{70}E_{90})_{q3}$‡	$D_{280/12}$ vs $D_{280/12}$	2/20	1/18	0·1	0·7
**(b) subtotal with data §**				**368/ 1397 (26·3%)**	**320/ 1374 (23·3%)**	**23·4**	**159·5**
**Total (a + b)**				**1032/ 4754 (21·7%)**	**1019/ 4762 (21·4%)**	**10·1**	**476·3**

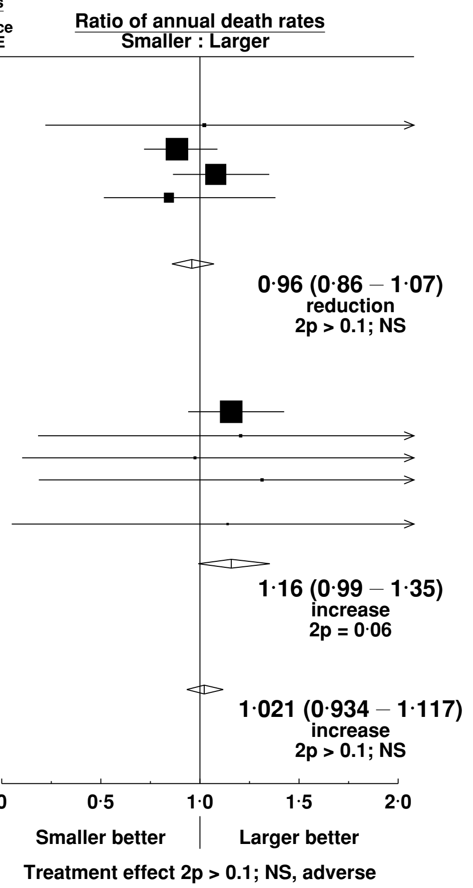

99% or 95% confidence intervals

Difference between treatment effects in 2 subtotals: $\chi^2_1 = 3{\cdot}8$; 2p = 0·05

Heterogeneity within subtotals: $\chi^2_7 = 3{\cdot}4$; p > 0.1; NS

Heterogeneity between 9 trials: $\chi^2_8 = 7{\cdot}2$; p > 0.1; NS

§ 2 trials with no data do not contribute to subtotals or to the overall total.

‡ Pre-operative chemotherapy
v.v Vice versa
Taxanes: D = docetaxel; P = paclitaxel
Anthracyclines: A = doxorubicin (Adriamycin); E = epirubicin
Other agents: C = cyclophosphamide; F = fluorouracil; M = methotrexate; Cap = capecitabine; Cpt = carboplatin

P44: Breast cancer mortality in trials comparing sequencing order of anthracycline and taxane chemotherapy

Year code and study name	Treatment comparison	Deaths/Women: Allocated Anth first	Deaths/Women: Allocated Other first	Anth first deaths: Logrank O−E	Anth first deaths: Variance of O−E
**Anthracycline/Taxane sequencing (same regimens, reversing order)**					
00N DFCI 99278	(4**A**$_{60q2}$;9**P**$_{80q1}$ v 9**P**$_{80q1}$;4**A**$_{60q2}$)‡	*(61 patients)*		*(no data)*	
02K NCC Goyang	(4**AC**‡;4**D**$_{75}$**Cap**$_{2000d1-14}$ v 4**D**$_{75}$**Cap**$_{2000d1-14}$‡;4**AC**)$_{q3}$	18/103	14/106	3·3	7·3
02U TAC	(4**AC**‡;4**D**$_{100}$ v 4**D**$_{100}$‡;4**AC**)$_{q3}$	*(120 patients)*		*(no data)*	
03L AERO B−03 France	(4**E**$_{100}$**C**;4**D**$_{100}$ v 4**D**$_{100}$;4**E**$_{100}$**C**)$_{q2}$	0/31	0/34		
03[KBCOG−02 Japan	4**E**$_{75}$**C**$_{q3}$‡;12**P**$_{80q1}$ v 12**P**$_{80q1}$‡;4**E**$_{75}$**C**$_{q3}$	13/87	10/84	1·5	5·1
04^ OSU Ohio	(4**AC**;4**D**$_{75}$ v 4**D**$_{75}$;4**AC**)$_{q2}$	*(56 patients)*		*(no data)*	
04{ Monash CC Australia	(4**FE**$_{100}$**C**;4**D**$_{100}$ v 4**D**$_{100}$;4**FE**$_{100}$**C**)$_{q3}$‡	*(69 patients)*		*(no data)*	
05@ UZLeuven Belgium	(3**FE**$_{100}$**C**;3**D**$_{100}$ v 3**D**$_{100}$;3**FE**$_{100}$**C**)$_{q3}$	5/39	5/46	0·6	1·8
05A Neo−tAnGo, UK	(4**E**$_{90}$**C**$_{q3}$;4**P**$_{175}$±**Gem**$_{2000q2}$ v 4**P**$_{175}$±**Gem**$_{2000q2}$;4**E**$_{90}$**C**$_{q3}$)‡	126/414	113/414	5·9	54·7
05X H San Carlos, Madrid	(4**A**$_{75}$‡;4**D**$_{100}$ v 4**D**$_{100}$‡;4**A**$_{75}$)$_{q3}$	*(204 patients)*		*(no data)*	
06N Shiga Japan	(3**FE**$_{100}$**C**;3**D**$_{100}$ v 3**D**$_{100}$;3**FE**$_{100}$**C**)$_{q3}$	*(42 patients)*		*(no data)*	
07Y ACOSOG USA Z1041	(4**E**$_{75}$**C**$_{q3}$‡;12**P**$_{80q1}$‡ v 12**P**$_{80q1}$‡;4**E**$_{75}$**C**$_{q3}$‡)**Trz**$_{q3}$ to 1yr	6/140	9/142	1·0	2·6
09U JBCRG−10	(4**FE**$_{100}$**C**;4**D**$_{75}$**C**$_{600}$**Trz** v 4**D**$_{75}$**C**$_{600}$**Trz**;4**FE**$_{100}$**C**)$_{q3}$‡	1/21	0/21	0·5	0·2
09V JBCRG−09	(3**FE**$_{100}$**C**;3**D**$_{75}$**C**$_{600}$ v 3**D**$_{75}$**C**$_{600}$;3**FE**$_{100}$**C**)$_{q3}$‡	2/65	4/63	−1·2	1·4
10G SWOG s0800	(6**AC**$_{q2}$;12**NabP**$_{100q1}$ v 12**NabP**$_{100q1}$;6**AC**$_{q2}$)‡	10/53	6/63	2·8	3·5
10J INC Brazil	(3**FA**$_{50}$**C**;3**D**$_{100}$ v 3**D**$_{100}$;3**FA**$_{50}$**C**)$_{q3}$‡	26/61	13/59	7·2	8·9
**■ Total §**		**207/ 1014 (20·4%)**	**174/ 1032 (16·9%)**	**21·6**	**85·6**

Ratio of annual death rates Anth first : Other first

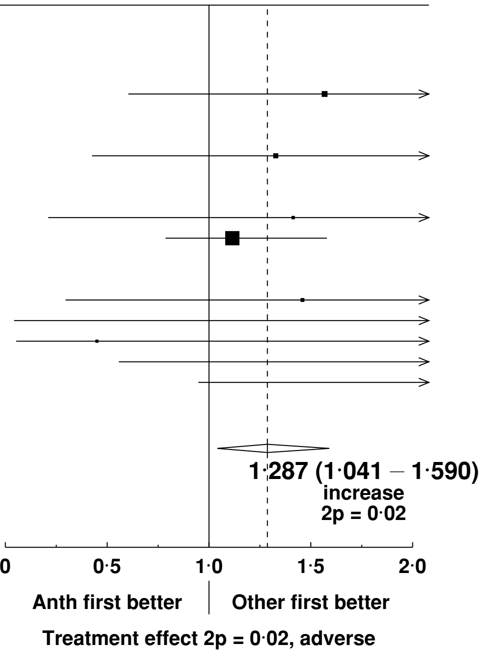

■ 99% or ◇ 95% confidence intervals

Heterogeneity between 9 trials: χ^2_8 = 7·6; p > 0.1; NS

§ 6 trials with no data do not contribute to the total.

‡ Pre−operative chemotherapy

Taxanes: D = docetaxel; P = paclitaxel

Anthracyclines: A = doxorubicin (Adriamycin); E = epirubicin

Other agents: C = cyclophosphamide; F = fluorouracil; M = methotrexate; Trz = trastuzumab; Cap = capecitabine; Gem = gemcitabine; NabP = Nab paclitaxel

P45: Death without recurrence in trials comparing sequencing order of anthracycline and taxane chemotherapy

Year code and study name	Treatment comparison	Deaths/Women: Allocated Anth first	Deaths/Women: Allocated Other first	Anth first deaths: Logrank O–E	Anth first deaths: Variance of O–E
**Anthracycline/Taxane sequencing (same regimens, reversing order)**					
00N DFCI 99278	(4A60q2;9P80q1 v 9P80q1;4A60q2)‡	(61 patients)		(no data)	
02K NCC Goyang	(4AC‡;4D75Cap2000d1−14 v 4D75Cap2000d1−14‡;4AC)q3	2/103	2/106	−0·2	0·9
02U TAC	(4AC‡;4D100 v 4D100‡;4AC)q3	(120 patients)		(no data)	
03L AERO B−03 France	(4E100C;4D100 v 4D100;4E100C)q2	0/31	0/34		
03[KBCOG−02 Japan	4E75Cq3‡;12P80q1 v 12P80q1‡;4E75Cq3	1/87	0/84	0·5	0·2
04^ OSU Ohio	(4AC;4D75 v 4D75;4AC)q2	(56 patients)		(no data)	
04{ Monash CC Australia	(4FE100C;4D100 v 4D100;4FE100C)q3‡	(69 patients)		(no data)	
05@ UZLeuven Belgium	(3FE100C;3D100 v 3D100;3FE100C)q3	1/39	1/46	−0·2	0·1
05A Neo−tAnGo, UK	(4E90Cq3;4P175±Gem2000q2 v 4P175±Gem2000q2;4E90Cq3)‡	3/414	4/414	−0·4	1·7
05X H San Carlos, Madrid	(4A75‡;4D100 v 4D100‡;4A75)q3	(204 patients)		(no data)	
06N Shiga Japan	(3FE100C;3D100 v 3D100;3FE100C)q3	(42 patients)		(no data)	
07Y ACOSOG USA Z1041	(4E75Cq3‡;12P80q1‡ v 12P80q1‡;4E75Cq3‡)Trzq3 to 1yr	2/140	3/142	−1·0	1·0
09U JBCRG−10	(4FE100C;4D75C600Trz v 4D75C600Trz;4FE100C)q3‡	2/21	0/21	0·6	0·2
09V JBCRG−09	(3FE100C;3D75C600 v 3D75C600;3FE100C)q3‡	1/65	0/63	0·3	0·2
10G SWOG s0800	(6ACq2;12NabP100q1 v 12NabP100q1;6ACq2)‡	2/53	2/63	0·1	1·0
10J INC Brazil	(3FA50C;3D100 v 3D100;3FA50C)q3‡	3/61	1/59	1·0	1·0
**Total §**		**17/ 1014 (1·7%)**	**13/ 1032 (1·3%)**	**0·9**	**6·4**

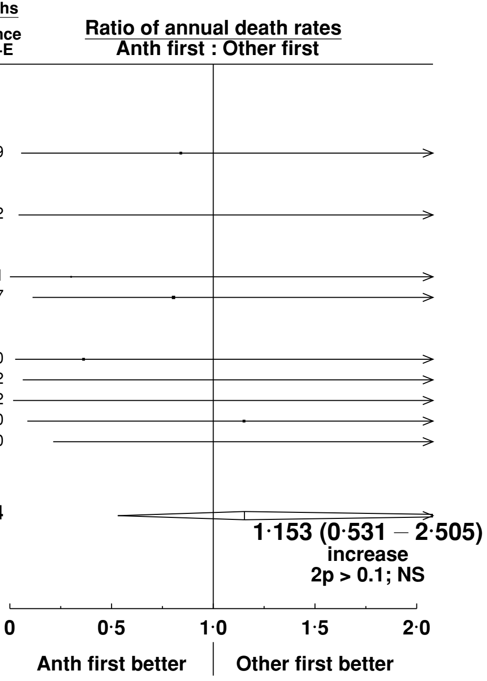

99% or 95% confidence intervals

Heterogeneity between 9 trials: $\chi^2_8 = 5{\cdot}2$; p > 0.1; NS

Treatment effect 2p > 0.1; NS, adverse

§ 6 trials with no data do not contribute to the total.

‡ Pre−operative chemotherapy
Taxanes: D = docetaxel; P = paclitaxel
Anthracyclines: A = doxorubicin (Adriamycin); E = epirubicin
Other agents: C = cyclophosphamide; F = fluorouracil; M = methotrexate; Trz = trastuzumab; Cap = capecitabine; Gem = gemcitabine; NabP = Nab paclitaxel

P46: All-cause mortality in trials comparing sequencing order of anthracycline and taxane chemotherapy

Year code and study name	Treatment comparison	Deaths/Women: Allocated Anth first	Deaths/Women: Allocated Other first	Anth first deaths: Logrank O−E	Anth first deaths: Variance of O−E
**Anthracycline/Taxane sequencing (same regimens, reversing order)**					
00N DFCI 99278	(4A60q2;9P80q1 v 9P80q1;4A60q2)‡	*(61 patients)*		*(no data)*	
02K NCC Goyang	(4AC‡;4D75Cap2000d1−14 v 4D75Cap2000d1−14‡;4AC)q3	20/103	16/106	3·1	8·2
02U TAC	(4AC‡;4D100 v 4D100‡;4AC)q3	*(120 patients)*		*(no data)*	
03L AERO B−03 France	(4E100C;4D100 v 4D100;4E100C)q2	0/31	0/34		
03[KBCOG−02 Japan	4E75Cq3‡;12P80q1 v 12P80q1‡;4E75Cq3	14/87	10/84	2·0	5·4
04^ OSU Ohio	(4AC;4D75 v 4D75;4AC)q2	*(56 patients)*		*(no data)*	
04{ Monash CC Australia	(4FE100C;4D100 v 4D100;4FE100C)q3‡	*(69 patients)*		*(no data)*	
05@ UZLeuven Belgium	(3FE100C;3D100 v 3D100;3FE100C)q3	6/39	6/46	0·5	2·0
05A Neo−tAnGo, UK	(4E90Cq3;4P175±Gem2000q2 v 4P175±Gem2000q2;4E90Cq3)‡	129/414	117/414	5·6	56·4
05X H San Carlos, Madrid	(4A75‡;4D100 v 4D100‡;4A75)q3	*(204 patients)*		*(no data)*	
06N Shiga Japan	(3FE100C;3D100 v 3D100;3FE100C)q3	*(42 patients)*		*(no data)*	
07Y ACOSOG USA Z1041	(4E75Cq3‡;12P80q1‡ v 12P80q1‡;4E75Cq3‡)Trzq3 to 1yr	8/140	12/142	0·0	3·6
09U JBCRG−10	(4FE100C;4D75C600Trz v 4D75C600Trz;4FE100C)q3‡	3/21	0/21	1·1	0·5
09V JBCRG−09	(3FE100C;3D75C600 v 3D75C600;3FE100C)q3‡	3/65	4/63	−0·8	1·7
10G SWOG s0800	(6ACq2;12NabP100q1 v 12NabP100q1;6ACq2)‡	12/53	8/63	2·9	4·5
10J INC Brazil	(3FA50C;3D100 v 3D100;3FA50C)q3‡	29/61	14/59	8·2	9·8
**Total §**		**224/ 1014 (22·1%)**	**187/ 1032 (18·1%)**	**22·5**	**92·0**

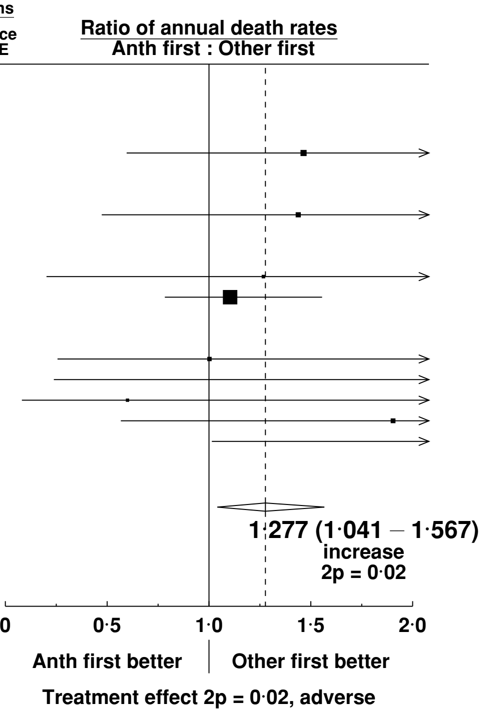

99% or 95% confidence intervals

Heterogeneity between 9 trials: $\chi^2_8 = 8{\cdot}7$; p > 0.1; NS

§ 6 trials with no data do not contribute to the total.

‡ Pre−operative chemotherapy
Taxanes: D = docetaxel; P = paclitaxel
Anthracyclines: A = doxorubicin (Adriamycin); E = epirubicin
Other agents: C = cyclophosphamide; F = fluorouracil; M = methotrexate; Trz = trastuzumab; Cap = capecitabine; Gem = gemcitabine; NabP = Nab paclitaxel

P47: Breast cancer mortality in trials of longer versus shorter athracycline/taxane regimens

Year code and study name		Treatment comparison	Cumulative anth dose and difference		Deaths/Women: Allocated Longer	Deaths/Women: Allocated Shorter	Longer deaths: Logrank O–E	Longer deaths: Variance of O–E
**(a) Longer versus <6 months of polychemotherapy (anthracycline)**								
74D_{1+2+4}	Boston	10 v 5 $A_{45}C_{500}$q3	A_{450} vs A_{225}	A_{225}	90/148	84/151	7·1	33·2
82Z_{2468}	Metaxas Athens	3$Vcr_{0.9}A_{35}C_{300x4}$q4±7$F_{440x2}A_{25x2}C_{50x15}$q4	A_{455} vs A_{105}	A_{350}	40/158	47/150	–4·4	13·6
86P_1	FASG GFEA 01	6 v 3 $F_{500}E_{100}C_{500}$q3	E_{600} vs E_{300}	E_{300}	79/212	82/209	–6·9	35·4
87Q	PRONACAM 87 Argent.	6 v 4 $C_{400}M_{30}F_{400}E_{60}Pr_{40x4}$q3	E_{360} vs E_{240}	E_{120}	*(556 patients)*		*(no data)*	
02B	CALGB 40101	6 v 4 $A_{60}C_{600}$q3	A_{360} vs A_{240}	A_{120}	11/144	7/140	2·0	4·3
02N	PACS 05 France	6 v 4 $F_{500}E_{100}C_{500}$q3	E_{600} vs E_{300}	E_{300}	33/759	41/756	–5·5	17·9
02X_1	CALGB 40101	6 v 4 $A_{60}C_{500}$q2	A_{360} vs A_{240}	A_{120}	22/645	29/655	–4·3	12·4
04C	NSABP B–36	(6$F_{500}E_{100}C_{500}$ v 4$A_{60}C_{600}$)q3	E_{600} vs A_{240}	E/A_{360}	91/1361	95/1361	–1·8	45·6
**(a) subtotal with data §**					**366/ 3427 (10·7%)**	**385/ 3422 (11·3%)**	**–13·8**	**162·3**
**(b) Longer versus <6 months of polychemotherapy (anthracycline and taxane)**								
97H_2	Multicentre France	6 v 4 $A_{60}P_{200}$q3‡	A_{360} vs A_{240}	A_{120}	*(232 patients)*		*(no data)*	
01S_2	GeparTrio / GBG 24	8 v 6 $D_{75}A_{50}C_{500}$q3‡	A_{400} vs A_{300}	A_{100}	69/686	84/704	–6·0	34·8
03%	Chungnam Korea	6 v 3 $E_{75}D_{75}$q3‡	E_{450} vs E_{225}	E_{225}	*(176 patients)*		*(no data)*	
**(b) subtotal with data §**					**69/ 686 (10·1%)**	**84/ 704 (11·9%)**	**–6·0**	**34·8**
**Total (a + b)**					**435/ 4113 (10·6%)**	**469/ 4126 (11·4%)**	**–19·8**	**197·2**

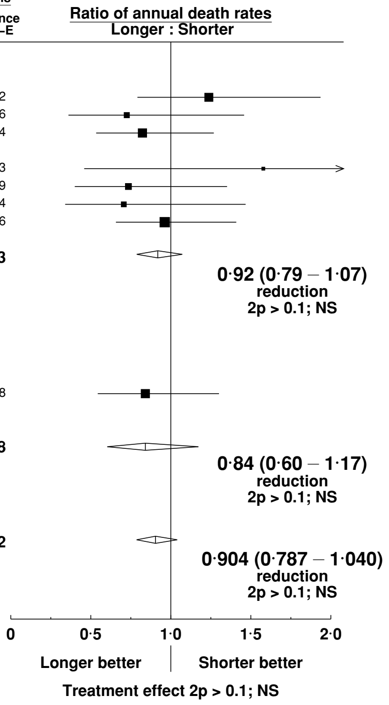

99% or 95% confidence intervals

Difference between treatment effects in 2 subtotals: $\chi^2_1 = 0{\cdot}2$; 2p > 0.1; NS

Heterogeneity within subtotals: $\chi^2_6 = 7{\cdot}3$; p > 0.1; NS

Heterogeneity between 8 trials: $\chi^2_7 = 7{\cdot}5$; p > 0.1; NS

§ 3 trials with no data do not contribute to subtotals or to the overall total.

‡ Pre–operative chemotherapy
Taxanes: D = docetaxel; P = paclitaxel
Anthracyclines: A = doxorubicin (Adriamycin); E = epirubicin
Other agents: C = cyclophosphamide; F = fluorouracil; M = methotrexate; Pr = prednisone; Vcr = vincristine

P48: Death without recurrence in trials of longer versus shorter athracycline/taxane regimens

Year code	Study name	Treatment comparison	Cumulative anth dose	Difference	Deaths/Women Allocated Longer	Deaths/Women Allocated Shorter	Longer deaths Logrank O–E	Longer deaths Variance of O–E	Ratio of annual death rates Longer : Shorter
**(a) Longer versus <6 months of polychemotherapy (anthracycline)**									
74D$_{1+2+4}$	Boston	10 v 5 $A_{45}C_{500}$q3	A_{450} vs A_{225}	A_{225}	16/148	13/151	0·1	4·7	
82Z$_{2468}$	Metaxas Athens	3$Vcr_{0.9}A_{35}C_{300x4}$q4±7$F_{440x2}A_{25x2}C_{50x15}$q4	A_{455} vs A_{105}	A_{350}	4/158	3/150	0·5	1·4	
86P$_{1}$	FASG GFEA 01	6 v 3 $F_{500}E_{100}C_{500}$q3	E_{600} vs E_{300}	E_{300}	6/212	4/209	0·8	2·5	
87Q	PRONACAM 87 Argent.	6 v 4 $C_{400}M_{30}F_{400}E_{60}Pr_{40x4}$q3	E_{360} vs E_{240}	E_{120}	*(556 patients)*		*(no data)*		
02B	CALGB 40101	6 v 4 $A_{60}C_{600}$q3	A_{360} vs A_{240}	A_{120}	4/144	4/140	0·1	2·0	
02N	PACS 05 France	6 v 4 $F_{500}E_{100}C_{500}$q3	E_{600} vs E_{300}	E_{300}	3/759	9/756	–2·7	2·9	
02X$_{1}$	CALGB 40101	6 v 4 $A_{60}C_{500}$q2	A_{360} vs A_{240}	A_{120}	16/645	10/655	2·6	6·1	
04C	NSABP B–36	(6$F_{500}E_{100}C_{500}$ v 4$A_{60}C_{600}$)q3	E_{600} vs A_{240}	E/A_{360}	48/1361	37/1361	4·8	21·1	
■	**(a) subtotal with data §**				**97/ 3427 (2·8%)**	**80/ 3422 (2·3%)**	**6·2**	**40·7**	**1·16 (0·86 – 1·58) increase 2p > 0.1; NS**
**(b) Longer versus <6 months of polychemotherapy (anthracycline and taxane)**									
97H$_{2}$	Multicentre France	6 v 4 $A_{60}P_{200}$q3‡	A_{360} vs A_{240}	A_{120}	*(232 patients)*		*(no data)*		
01S$_{2}$	GeparTrio / GBG 24	8 v 6 $D_{75}A_{50}C_{500}$q3‡	A_{400} vs A_{300}	A_{100}	25/686	40/704	–5·9	15·7	
03%	Chungnam Korea	6 v 3 $E_{75}D_{75}$q3‡	E_{450} vs E_{225}	E_{225}	*(176 patients)*		*(no data)*		
■	**(b) subtotal with data §**				**25/ 686 (3·6%)**	**40/ 704 (5·7%)**	**–5·9**	**15·7**	**0·69 (0·42 – 1·13) reduction 2p > 0.1; NS**
■	**Total (a + b)**				**122/ 4113 (3·0%)**	**120/ 4126 (2·9%)**	**0·3**	**56·4**	**1·006 (0·775 – 1·306) increase 2p > 0.1; NS**

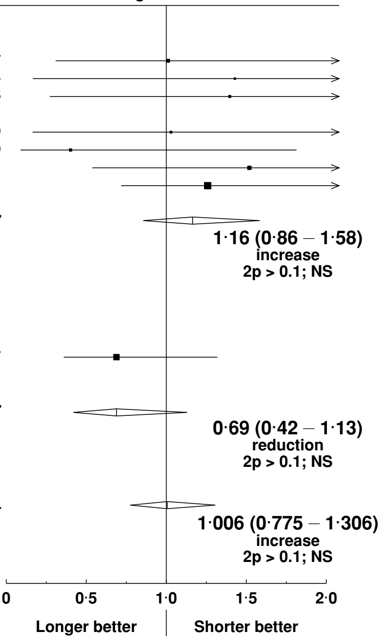

■ 99% or ◇ 95% confidence intervals

Difference between treatment effects in 2 subtotals: $\chi^2_1 = 3{\cdot}1$; 2p = 0·08

Heterogeneity within subtotals: $\chi^2_6 = 4{\cdot}1$; p > 0.1; NS

Heterogeneity between 8 trials: $\chi^2_7 = 7{\cdot}3$; p > 0.1; NS

§ 3 trials with no data do not contribute to subtotals or to the overall total.

‡ Pre–operative chemotherapy

Taxanes: D = docetaxel; P = paclitaxel

Anthracyclines: A = doxorubicin (Adriamycin); E = epirubicin

Other agents: C = cyclophosphamide; F = fluorouracil; M = methotrexate; Pr = prednisone; Vcr = vincristine

P49: All-cause mortality in trials of longer versus shorter athracycline/taxane regimens

Year code and study name	Treatment comparisons	Cumulative anth dose and difference		Deaths/Women Allocated Longer	Deaths/Women Allocated Shorter	Longer deaths Logrank O−E	Longer deaths Variance of O−E	Ratio of annual death rates Longer : Shorter
**(a) Longer versus <6 months of polychemotherapy (anthracycline)**								
$74D_{1+2+4}$ Boston	10 v 5 $A_{45}C_{500}$q3	A_{450} vs A_{225}	A_{225}	106/148	97/151	7·1	37·9	
$82Z_{2468}$ Metaxas Athens	3$Vcr_{0.9}A_{35}C_{300x4}$q4±7$F_{440x2}A_{25x2}C_{50x15}$q4	A_{455} vs A_{105}	A_{350}	44/158	50/150	−3·9	15·0	
$86P_1$ FASG GFEA 01	6 v 3 $F_{500}E_{100}C_{500}$q3	E_{600} vs E_{300}	E_{300}	85/212	86/209	−6·1	37·9	
87Q PRONACAM 87 Argent.	6 v 4 $C_{400}M_{30}F_{400}E_{60}Pr_{40x4}$q3	E_{360} vs E_{240}	E_{120}	*(556 patients)*		*(no data)*		
02B CALGB 40101	6 v 4 $A_{60}C_{600}$q3	A_{360} vs A_{240}	A_{120}	15/144	11/140	2·0	6·3	
02N PACS 05 France	6 v 4 $F_{500}E_{100}C_{500}$q3	E_{600} vs E_{300}	E_{300}	36/759	50/756	−8·2	20·8	
$02X_1$ CALGB 40101	6 v 4 $A_{60}C_{500}$q2	A_{360} vs A_{240}	A_{120}	38/645	39/655	−1·7	18·6	
04C NSABP B−36	(6$F_{500}E_{100}C_{500}$ v 4$A_{60}C_{600}$)q3	E_{600} vs A_{240}	E/A_{360}	139/1361	132/1361	3·1	66·6	
**(a) subtotal with data §**				**463/ 3427 (13·5%)**	**465/ 3422 (13·6%)**	**−7·6**	**203·1**	**0·96 (0·84 – 1·11) reduction 2p > 0.1; NS**
**(b) Longer versus <6 months of polychemotherapy (anthracycline and taxane)**								
$97H_2$ Multicentre France	6 v 4 $A_{60}P_{200}$q3‡	A_{360} vs A_{240}	A_{120}	*(232 patients)*		*(no data)*		
$01S_2$ GeparTrio / GBG 24	8 v 6 $D_{75}A_{50}C_{500}$q3‡	A_{400} vs A_{300}	A_{100}	94/686	124/704	−11·9	50·5	
03% Chungnam Korea	6 v 3 $E_{75}D_{75}$q3‡	E_{450} vs E_{225}	E_{225}	*(176 patients)*		*(no data)*		
**(b) subtotal with data §**				**94/ 686 (13·7%)**	**124/ 704 (17·6%)**	**−11·9**	**50·5**	**0·79 (0·60 – 1·04) reduction 2p = 0·09**
**Total (a + b)**				**557/ 4113 (13·5%)**	**589/ 4126 (14·3%)**	**−19·5**	**253·6**	**0·926 (0·819 – 1·047) reduction 2p > 0.1; NS**

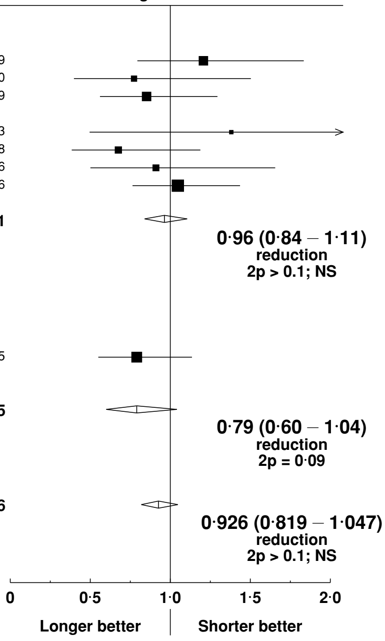

99% or 95% confidence intervals

Difference between treatment effects in 2 subtotals: $\chi^2_1 = 1{\cdot}6$; 2p > 0.1; NS

Heterogeneity within subtotals: $\chi^2_6 = 7{\cdot}2$; p > 0.1; NS

Heterogeneity between 8 trials: $\chi^2_7 = 8{\cdot}8$; p > 0.1; NS

Treatment effect 2p > 0.1; NS

§ 3 trials with no data do not contribute to subtotals or to the overall total.

‡ Pre−operative chemotherapy

Taxanes: D = docetaxel; P = paclitaxel

Anthracyclines: A = doxorubicin (Adriamycin); E = epirubicin

Other agents: C = cyclophosphamide; F = fluorouracil; M = methotrexate; Pr = prednisone; Vcr = vincristine

P50: Breast cancer mortality in trials of higher versus lower anthracycline dose

Year code	Study name	Regimens compared and drug doses (mg/m²) per cycle	Cumulative dosages, E/A	and difference	Deaths/Women: Allocated Higher	Deaths/Women: Adjusted Lower	Higher deaths: Logrank O−E	Higher deaths: Variance of O−E	Ratio of annual death rates Higher : Lower
**(a) Increase in anthracycline dose/course and total dose (<100mg A or 150mg E difference)**									
86P1	FASG GFEA 01	3(FE75C) v 3(F500E50C500)	E225 vs E150¶	E75	77/200	82/209	–5·9	35·7	
94D1	CALGB 9344 *	4(A75/90 v A60)C600	A300/360 vs A240	A90	399/1054	2(190/526)	6·3	120·0	
94D2	CALGB 9344 *	4(A75/90 v A60)C600;4P175	A300/360 vs A240	A90	344/1056	2(194/534)	–16·0	110·9	
05Z	CECOG Austria	6(FE90C v F500E75C500)q2	E540 vs E450¶	E90	*(51 patients)*		*(no data)*		
	**(a) subtotal with data §**				**820/ 2310 (35·5%)**	**850/ 2329 (36·5%)**	**–15·6**	**266·6**	**0·94 (0·84 – 1·06) reduction 2p > 0.1; NS**
**(b) Increase in anthracycline dose/course and total dose (>100mg A or 150mg E difference)**									
90C3+5	FASG GFEA 05	6(FE100C v F500E50C500)	E600 vs E300¶	E300	88/276	109/289	–14·3	42·6	
92N	ICCG C/9/91 UK	8(FE75C v F600E50C600)	E600 vs E400¶	E200	59/391	68/394	–0·8	27·5	
05S	Tianjin Chemo	6(FE100C v F500E60C600)	E600 vs E360¶	E240	*(98 patients)*		*(no data)*		
	**(b) subtotal with data §**				**147/ 667 (22·0%)**	**177/ 683 (25·9%)**	**–15·1**	**70·1**	**0·81 (0·64 – 1·02) reduction 2p = 0·07**
**(c) Increase in anthracycline and other drug dose/course and total dose**									
77S	INT Milan 7701	(6C[50–150]M[20–60]F[400–800];4A[50–75]Vcr v 6CMF;4A60Vcr1.4)q4	A250 vs A240	A10	40/70	50/70	–3·6	18·7	
85A	CALGB CLB–8541	4(F600A60C600 v F300A30C300)q4	A240 vs A120¶	A120	224/528	268/522	–35·1	111·9	
88R	Brussels Belgium	8(E100C830 v E60C500)	E800 vs E480¶	E320	84/265	101/272	–11·9	40·5	
91F	MD Anderson	4(F1200A60C1000 v F1000A50C500)‡	A240 vs A200¶	A40	*(112 patients)*		*(no data)*		
93@	GFEA Inflammatory	4(F750x4E35x3C400x3 v F500E75C500)‡;4FEC	E720 vs E600¶	E120			*(no data)*		
94Q	Austrian BCSG X	4(E60x2C600x2 v E60C600);4CMF	E480 vs E240¶	E240	13/56	12/56	–0·6	4·9	
96L	OCSGL Poland	(6F500x2E60x2C75x14)q4 v F500E50C500)q3	E720 vs E300¶	E420	*(87 patients)*		*(no data)*		
96V	FNCLCC France	4(F1000E100C1000 v F500E60C500)‡	E400 vs E240¶	E160	8/42	8/42	–1·9	1·4	
01Q	SBG 2000–1/CEF–60	6(F600E[75–90]C[900–1200] v F600E60C600)	E510 vs E360¶	E150	77/524	96/528	–12·0	40·1	
04=	SBG 2004–1, Sweden	4E[90–120]Cq2;4D[75–100]q2 v 4E90C600q2;4D75q2	E435 vs E360	E75	9/42	8/42	–0·6	3·3	
	**(c) subtotal with data §**				**455/ 1527 (29·8%)**	**543/ 1532 (35·4%)**	**–65·7**	**220·9**	**0·74 (0·65 – 0·85) reduction 2p < 0·00001**
	**Total (a + b + c)**				**1422/ 4504 (31·6%)**	**1570/ 4544 (34·6%)**	**–96·4**	**557·5**	**0·841 (0·774 – 0·914) reduction 2p = 0·00004**

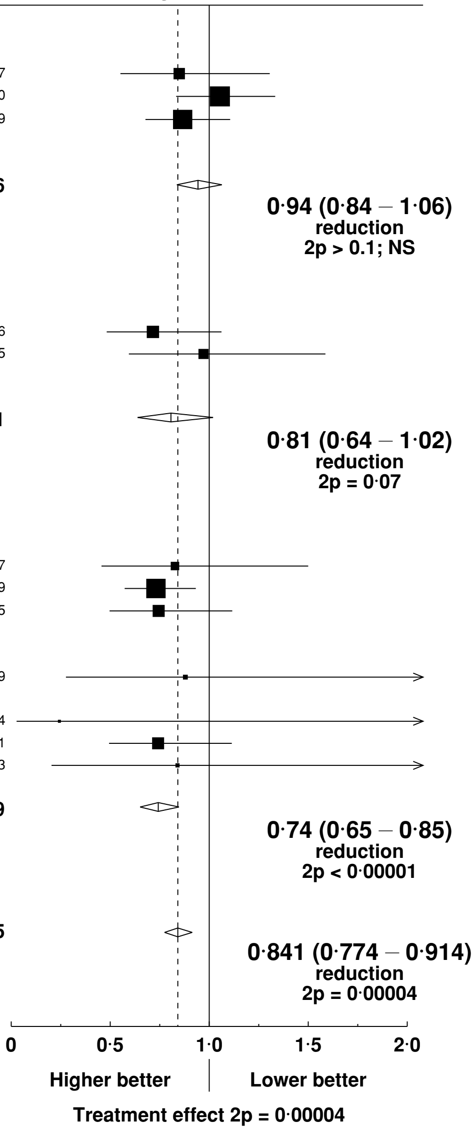

99% or 95% confidence intervals

Heterogeneity between 3 subtotals: χ^2_2 = 7·1; p = 0·03

Heterogeneity within subtotals: χ^2_9 = 6·4; p > 0.1; NS

Heterogeneity between 12 trials: χ^2_{11} = 13·5; p > 0.1; NS

§ 5 trials with no data do not contribute to subtotals or to the overall total.

* For balance, control patients in 3–way trials or trial strata count half or twice in subtotal(s) and in final total of deaths/patients.

‡ Pre-operative chemotherapy
¶ Control anthracycline dose less than E90 or A60 per cycle
Taxanes: D = docetaxel; P = paclitaxel. Anthracyclines: A = doxorubicin (Adriamycin); E = Epirubicin.
Other agents: C = cyclophosphamide; F = fluorouracil; M = methotrexate; Vcr = vincristine
All regimens q3week (unless specified as q1, q2 or q4). Semicolon [;] indicates treatment sequence. ×14 means d1-14 po; ×2 (×3) means d1, d8 (d15) iv
77S in control arm dose for CMF are C100M40F600

P51: Death without recurrence in trials of higher versus lower anthracycline dose

Year code and study name		Regimens compared and drug doses (mg/m²) per cycle	Cumulative dosages, E/A and difference		Deaths/Women: Allocated Higher	Deaths/Women: Adjusted Lower	Higher deaths: Logrank O−E	Higher deaths: Variance of O−E
**(a) Increase in anthracycline dose/course and total dose (<100mg A or 150mg E difference)**								
86P1	FASG GFEA 01	3(FE75C) v 3(F500E50C500)	E225 vs E150¶	E75	3/200	4/209	−0·9	1·6
94D1	CALGB 9344 *	4(A75/90 v A60)C600	A300/360 vs A240	A90	74/1054	2(35/526)	2·5	22·4
94D2	CALGB 9344 *	4(A75/90 v A60)C600;4P175	A300/360 vs A240	A90	71/1056	2(33/534)	3·1	21·9
05Z	CECOG Austria	6(FE90C v F500E75C500)q2	E540 vs E450¶	E90	*(51 patients)*		*(no data)*	
■	**(a) subtotal with data §**				**148/ 2310 (6·4%)**	**140/ 2329 (6·0%)**	**4·7**	**45·9**
**(b) Increase in anthracycline dose/course and total dose (>100mg A or 150mg E difference)**								
90C3+5	FASG GFEA 05	6(FE100C v F500E50C500)	E600 vs E300¶	E300	6/276	6/289	−0·6	2·9
92N	ICCG C/9/91 UK	8(FE75C v F600E50C600)	E600 vs E400¶	E200	2/391	2/394	0·2	0·9
05S	Tianjin Chemo	6(FE100C v F500E60C600)	E600 vs E360¶	E240	*(98 patients)*		*(no data)*	
▪	**(b) subtotal with data §**				**8/ 667 (1·2%)**	**8/ 683 (1·2%)**	**−0·4**	**3·8**
**(c) Increase in anthracycline and other drug dose/course and total dose**								
77S	INT Milan 7701	(6C[50−150]M[20−60]F[400−800];4A[50−75]Vcr v 6CMF;4A60Vcr1.4)q4	A250 vs A240	A10	10/70	7/70	1·5	3·6
85A	CALGB CLB−8541	4(F600A60C600 v F300A30C300)q4	A240 vs A120¶	A120	42/528	27/522	3·9	15·5
88R	Brussels Belgium	8(E100C830 v E60C500)	E800 vs E480¶	E320	25/265	15/272	4·7	8·8
91F	MD Anderson	4(F1200A60C1000 v F1000A50C500)‡	A240 vs A200¶	A40	*(112 patients)*		*(no data)*	
93@	GFEA Inflammatory	4(F750x4E35x3C400x3 v F500E75C500)‡;4FEC	E720 vs E600¶	E120			*(no data)*	
94Q	Austrian BCSG X	4(E60x2C600x2 v E60C600);4CMF	E480 vs E240¶	E240	2/56	1/56	0·4	0·7
96L	OCSGL Poland	(6F500x2E60x2C75x14)q4 v F500E50C500)q3	E720 vs E300¶	E420	*(87 patients)*		*(no data)*	
96V	FNCLCC France	4(F1000E100C1000 v F500E60C500)‡	E400 vs E240¶	E160	3/42	0/42	0·4	0·2
01Q	SBG 2000−1/CEF−60	6(F600E[75−90]C[900−1200] v F600E60C600)	E510 vs E360¶	E150	14/524	16/528	−1·1	7·0
04=	SBG 2004−1, Sweden	4E[90−120]Cq2;4D[75−100]q2 v 4E90C600q2;4D75q2	E435 vs E360	E75	0/42	1/42	−0·7	0·2
■	**(c) subtotal with data §**				**96/ 1527 (6·3%)**	**67/ 1532 (4·4%)**	**9·0**	**36·0**
■	**Total (a + b + c)**				**252/ 4504 (5·6%)**	**215/ 4544 (4·7%)**	**13·4**	**85·7**

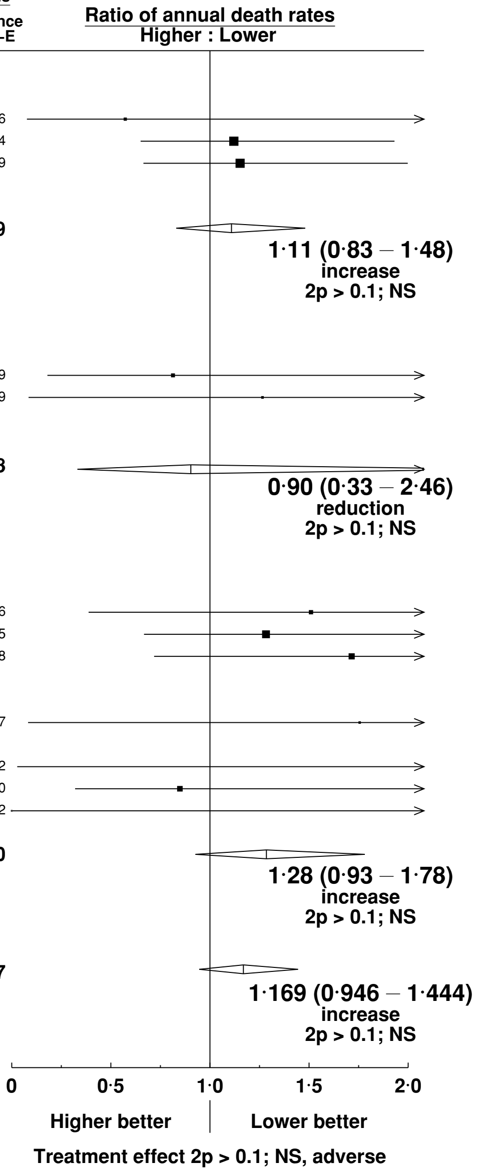

■ 99% or ◇ 95% confidence intervals

Heterogeneity between 3 subtotals: $\chi^2_2 = 0{\cdot}7$; p > 0.1; NS

Heterogeneity within subtotals: $\chi^2_9 = 6{\cdot}2$; p > 0.1; NS

Heterogeneity between 12 trials: $\chi^2_{11} = 6{\cdot}9$; p > 0.1; NS

§ 5 trials with no data do not contribute to subtotals or to the overall total.

* For balance, control patients in 3−way trials or trial strata count half or twice in subtotal(s) and in final total of deaths/patients.

‡ Pre-operative chemotherapy
¶ Control anthracycline dose less than E90 or A60 per cycle
Taxanes: D = docetaxel; P = paclitaxel. Anthracyclines: A = doxorubicin (Adriamycin); E = Epirubicin.
Other agents: C = cyclophosphamide; F = fluorouracil; M = methotrexate; Vcr = vincristine
All regimens q3week (unless specified as q1, q2 or q4). Semicolon [;] indicates treatment sequence. ×14 means d1-14 po; ×2 (×3) means d1, d8 (d15) iv
77S in control arm dose for CMF are C100M40F600

P52: All-cause mortality in trials of higher versus lower anthracycline dose

Year code	Study name	Regimens compared and drug doses (mg/m²) per cycle	Cumulative dosages, E/A	Difference	Deaths/Women: Allocated Higher	Deaths/Women: Adjusted Lower	Higher deaths: Logrank O−E	Higher deaths: Variance of O−E	Ratio of annual death rates Higher : Lower
**(a) Increase in anthracycline dose/course and total dose (<100mg A or 150mg E difference)**									
86P1	FASG GFEA 01	3(FE75C) v 3(F500E50C500)	E225 vs E150¶	E75	80/200	86/209	−6·8	37·4	
94D1	CALGB 9344 *	4(A75/90 v A60)C600	A300/360 vs A240	A90	473/1054	2(225/526)	8·8	142·4	
94D2	CALGB 9344 *	4(A75/90 v A60)C600;4P175	A300/360 vs A240	A90	415/1056	2(227/534)	−12·9	132·8	
05Z	CECOG Austria	6(FE90C v F500E75C500)q2	E540 vs E450¶	E90	(51 patients)		(no data)		
	**(a) subtotal with data §**				**968/ 2310 (41·9%)**	**990/ 2329 (42·5%)**	**−10·8**	**312·5**	**0·97 (0·86 – 1·08) reduction 2p > 0.1; NS**
**(b) Increase in anthracycline dose/course and total dose (>100mg A or 150mg E difference)**									
90C3+5	FASG GFEA 05	6(FE100C v F500E50C500)	E600 vs E300¶	E300	94/276	115/289	−14·9	45·5	
92N	ICCG C/9/91 UK	8(FE75C v F600E50C600)	E600 vs E400¶	E200	61/391	70/394	−0·6	28·4	
05S	Tianjin Chemo	6(FE100C v F500E60C600)	E600 vs E360¶	E240	(98 patients)		(no data)		
	**(b) subtotal with data §**				**155/ 667 (23·2%)**	**185/ 683 (27·1%)**	**−15·5**	**73·9**	**0·81 (0·65 – 1·02) reduction 2p = 0·07**
**(c) Increase in anthracycline and other drug dose/course and total dose**									
77S	INT Milan 7701	(6C[50−150]M[20−60]F[400−800];4A[50−75]Vcr v 6CMF;4A60Vcr1.4)q4	A250 vs A240	A10	50/70	57/70	−2·1	22·3	
85A	CALGB CLB−8541	4(F600A60C600 v F300A30C300)q4	A240 vs A120¶	A120	266/528	295/522	−31·2	127·5	
88R	Brussels Belgium	8(E100C830 v E60C500)	E800 vs E480¶	E320	109/265	116/272	−7·2	49·2	
91F	MD Anderson	4(F1200A60C1000 v F1000A50C500)‡	A240 vs A200¶	A40	(112 patients)		(no data)		
93@	GFEA Inflammatory	4(F750x4E35x3C400x3 v F500E75C500)‡;4FEC	E720 vs E600¶	E120			(no data)		
94Q	Austrian BCSG X	4(E60x2C600x2 v E60C600);4CMF	E480 vs E240¶	E240	15/56	13/56	−0·2	5·7	
96L	OCSGL Poland	(6F500x2E60x2C75x14)q4 v F500E50C500)q3	E720 vs E300¶	E420	(87 patients)		(no data)		
96V	FNCLCC France	4(F1000E100C1000 v F500E60C500)‡	E400 vs E240¶	E160	11/42	8/42	−1·5	1·6	
01Q	SBG 2000−1/CEF−60	6(F600E[75−90]C[900−1200] v F600E60C600)	E510 vs E360¶	E150	91/524	112/528	−13·1	47·1	
04=	SBG 2004−1, Sweden	4E[90−120]Cq2;4D[75−100]q2 v 4E90C600q2;4D75q2	E435 vs E360	E75	9/42	9/42	−1·3	3·5	
	**(c) subtotal with data §**				**551/ 1527 (36·1%)**	**610/ 1532 (39·8%)**	**−56·7**	**256·9**	**0·80 (0·71 – 0·91) reduction 2p = 0·0004**
	**Total (a + b + c)**				**1674/ 4504 (37·2%)**	**1785/ 4544 (39·3%)**	**−83·0**	**643·2**	**0·879 (0·814 – 0·950) reduction 2p = 0·001**

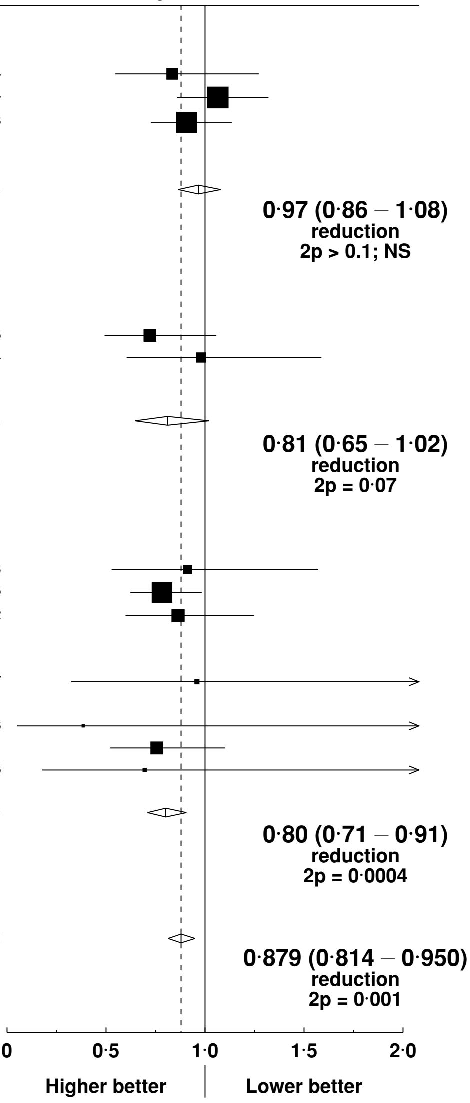

Treatment effect 2p = 0·001

99% or 95% confidence intervals

Heterogeneity between 3 subtotals: χ^2_2 = 5·4; p = 0·07

Heterogeneity within subtotals: χ^2_9 = 6·3; p > 0.1; NS

Heterogeneity between 12 trials: χ^2_{11} = 11·7; p > 0.1; NS

§ 5 trials with no data do not contribute to subtotals or to the overall total.

* For balance, control patients in 3−way trials or trial strata count half or twice in subtotal(s) and in final total of deaths/patients.

‡ Pre−operative chemotherapy
¶ Control anthracycline dose less than E90 or A60 per cycle
Taxanes: D = docetaxel; P = paclitaxel. Anthracyclines: A = doxorubicin (Adriamycin); E = Epirubicin.
Other agents: C = cyclophosphamide; F = fluorouracil; M = methotrexate; Vcr = vincristine
All regimens q3week (unless specified as q1, q2 or q4). Semicolon [;] indicates treatment sequence. ×14 means d1-14 po; ×2 (×3) means d1, d8 (d15) iv
77S in control arm dose for CMF are C100M40F600

P53: 10-year cumulative risk of (A) any recurrence, (B) breast cancer mortality, (C) death without recurrence, (D) all-cause mortality; in trials of longer versus shorted anthracycline/taxane regimen (smoothed after 5 years)

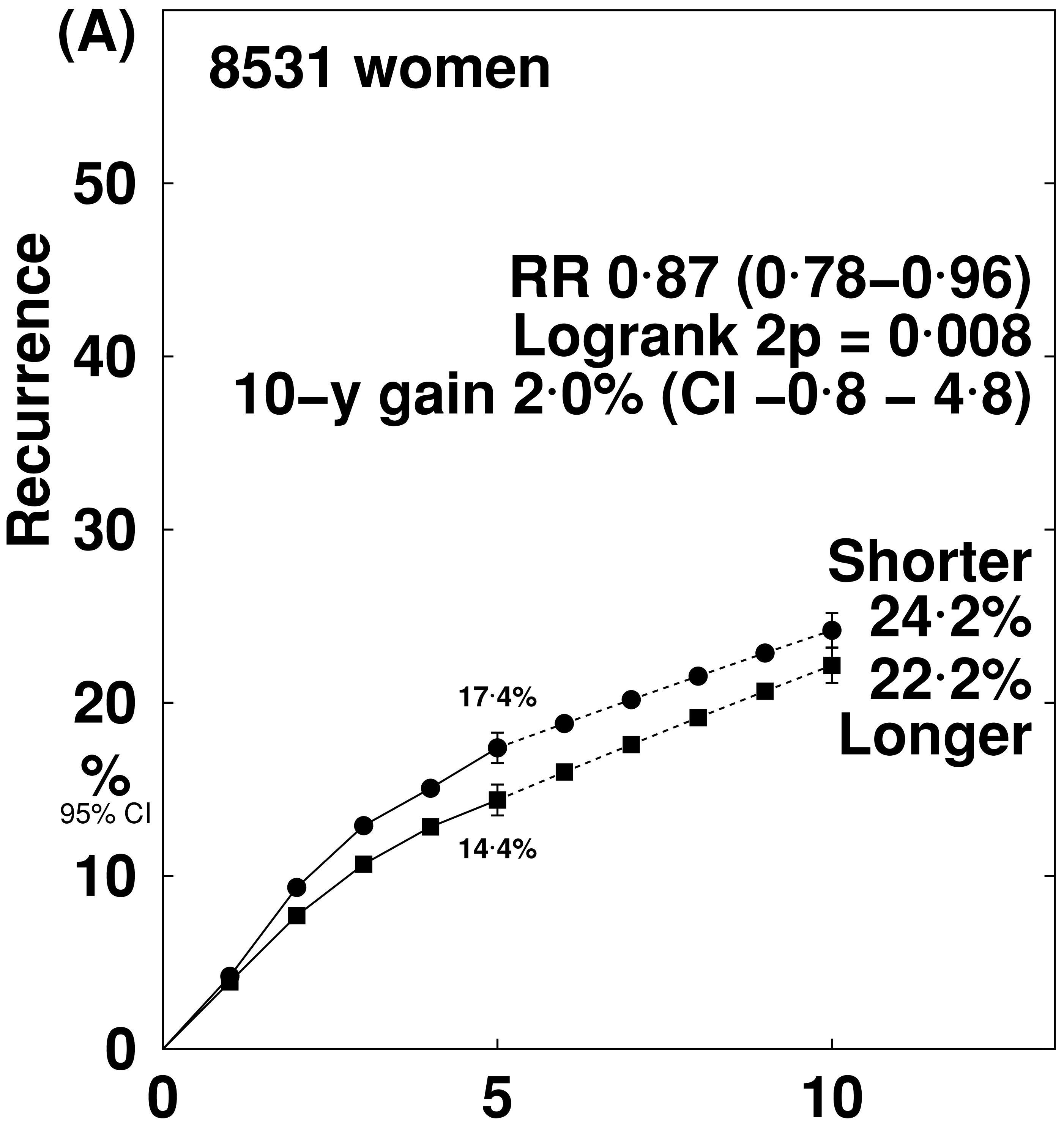

Recurrence rates (% / year) and logrank analyses

Allocation	Years 0 – 4	Years 5 – 9	Year 10+
Longer	3·25 (573 / 17623)	1·91 (148 / 7763)	2·98 (15 / 503)
Shorter	3·83 (667 / 17424)	1·71 (129 / 7538)	2·12 (10 / 472)
Rate ratio, from (O–E) / V	0·81 CI 0·72 – 0·92<br>–54·9 / 268·3	1·11 CI 0·87 – 1·43<br>6·5 / 61·9	1·03 CI 0·39 – 2·67<br>0·1 / 4·2

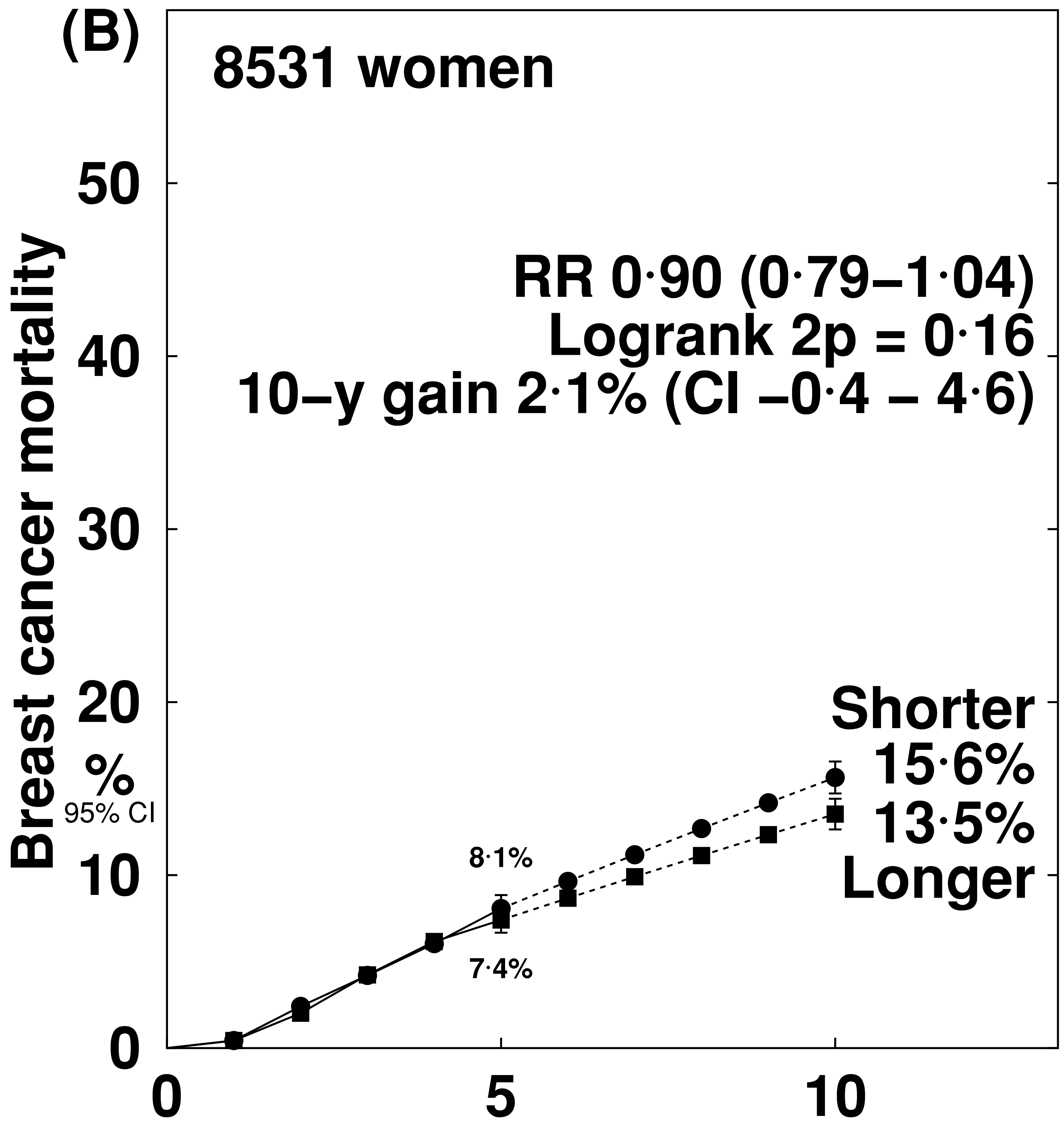

Death rates (% / year: total rate – rate in women without recurrence) & logrank analyses

Allocation	Years 0 – 4	Years 5 – 9	Year 10+
Longer	1·53 CI 1·35 – 1·70	1·44 CI 1·19 – 1·70	3·41 CI 1·99 – 4·84
Shorter	1·65 CI 1·46 – 1·83	1·64 CI 1·37 – 1·91	2·44 CI 1·25 – 3·64
Rate ratio, from (O–E) / V	0·93 CI 0·78 – 1·10<br>–10·2 / 131·4	0·79 CI 0·61 – 1·03<br>–13·4 / 58·4	1·67 CI 0·81 – 3·45<br>3·8 / 7·4

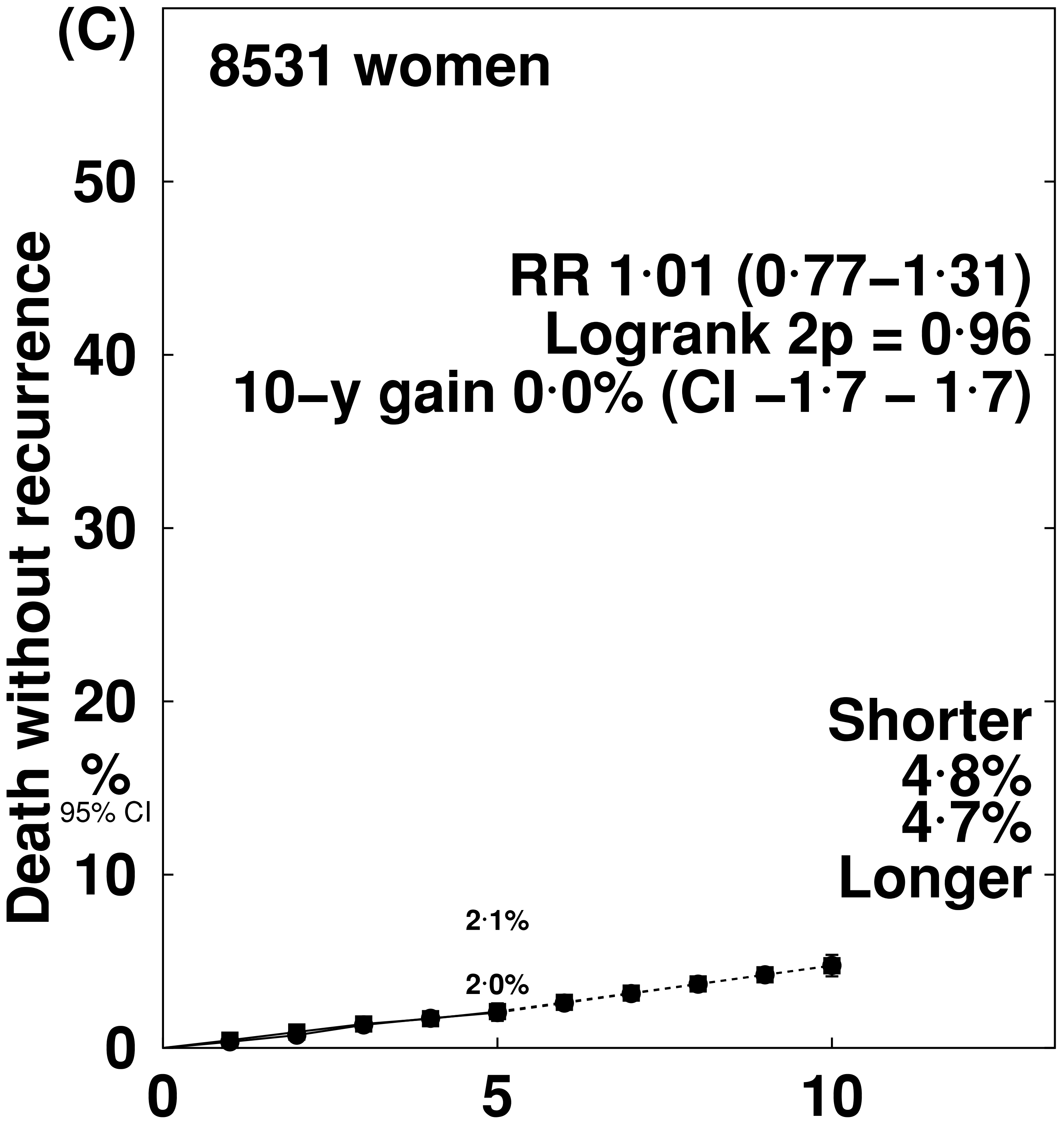

Death–without–recurrence rates (% / year) and logrank analyses

Allocation	Years 0 – 4	Years 5 – 9	Year 10+
Longer	0·41 (72 / 17623)	0·57 (44 / 7763)	1·19 (6 / 503)
Shorter	0·43 (75 / 17424)	0·54 (41 / 7538)	0·85 (4 / 472)
Rate ratio, from (O–E) / V	1·03 CI 0·74 – 1·44<br>1·1 / 35·1	0·97 CI 0·63 – 1·51<br>–0·5 / 19·9	0·83 CI 0·16 – 4·35<br>–0·3 / 1·4

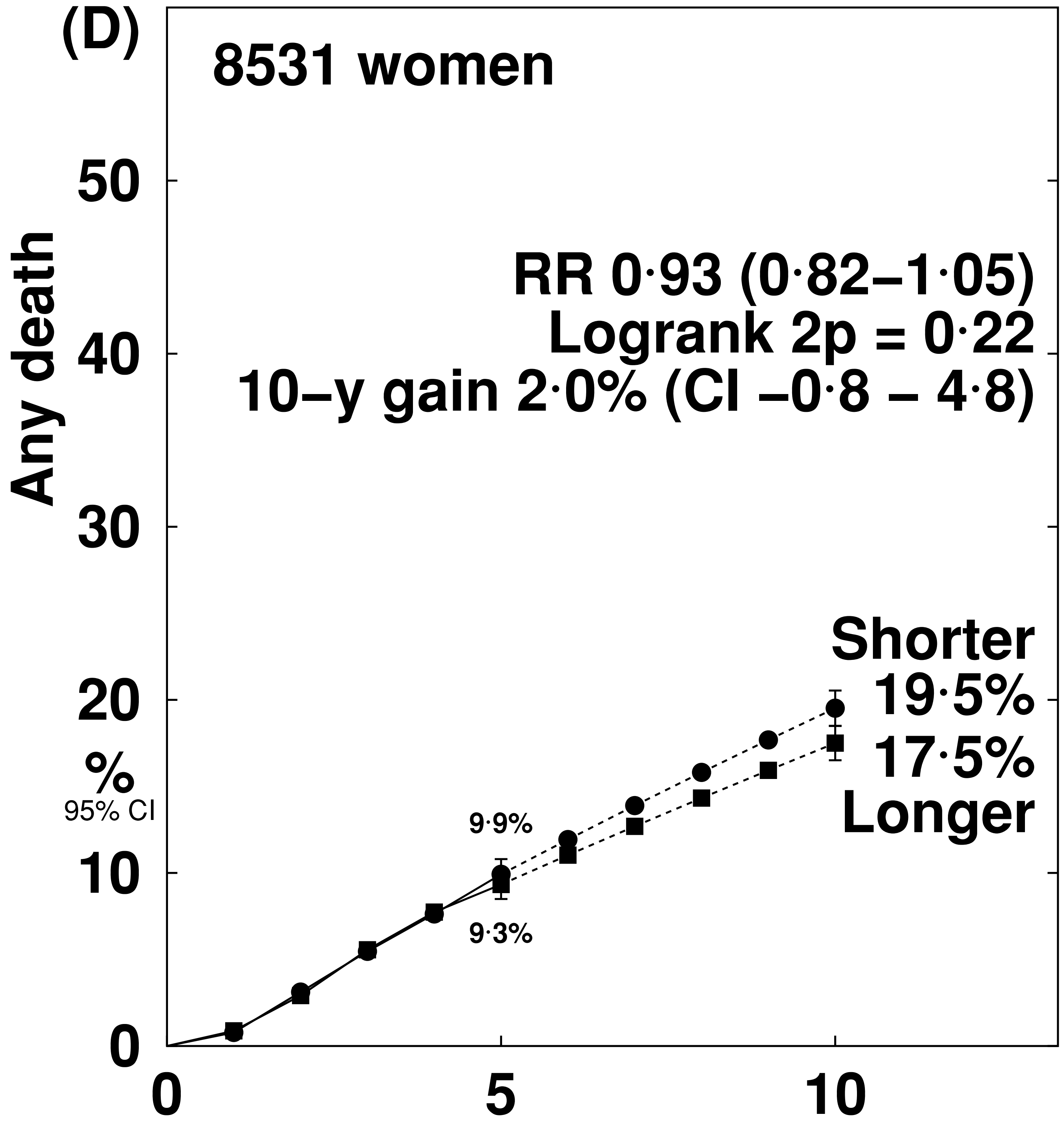

Death rates (% / year) and logrank analyses

Allocation	Years 0 – 4	Years 5 – 9	Year 10+
Longer	1·93 (359 / 18621)	1·98 (170 / 8600)	4·34 (28 / 645)
Shorter	2·07 (386 / 18633)	2·17 (183 / 8449)	3·05 (20 / 655)
Rate ratio, from (O–E) / V	0·95 CI 0·81 – 1·10<br>–9·1 / 166·6	0·84 CI 0·67 – 1·04<br>–13·9 / 78·3	1·50 CI 0·77 – 2·90<br>3·5 / 8·8

P54: 10-year cumulative risk of (A) any recurrence, (B) breast cancer mortality, (C) death without recurrence, (D) all-cause mortality; in trials of higher versus lower anthracycline dose

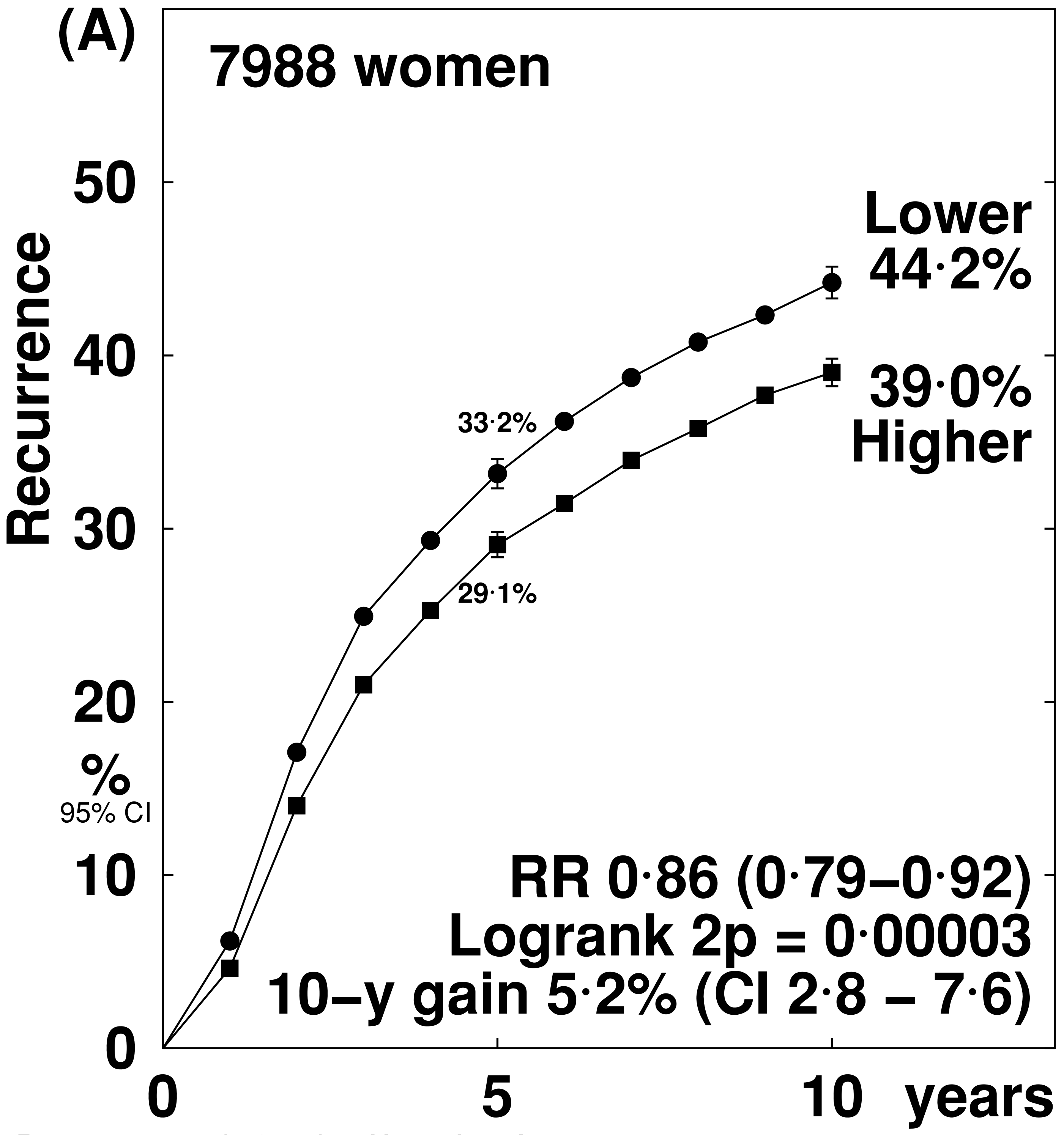

Recurrence rates (% / year) and logrank analyses

Allocation	Years 0 – 4	Years 5 – 9	Year 10+
Higher	7·16 (1306 / 18250)	3·09 (372 / 12039)	2·33 (185 / 7935)
Lower	8·10 (1104 / 13625)	3·77 (326 / 8657)	2·20 (115 / 5234)
Rate ratio, from (O–E) / V	0·84 CI 0·77 – 0·92<br>–86·2 / 493·8	0·83 CI 0·71 – 0·98<br>–28·1 / 153·1	1·06 CI 0·82 – 1·35<br>3·4 / 62·9

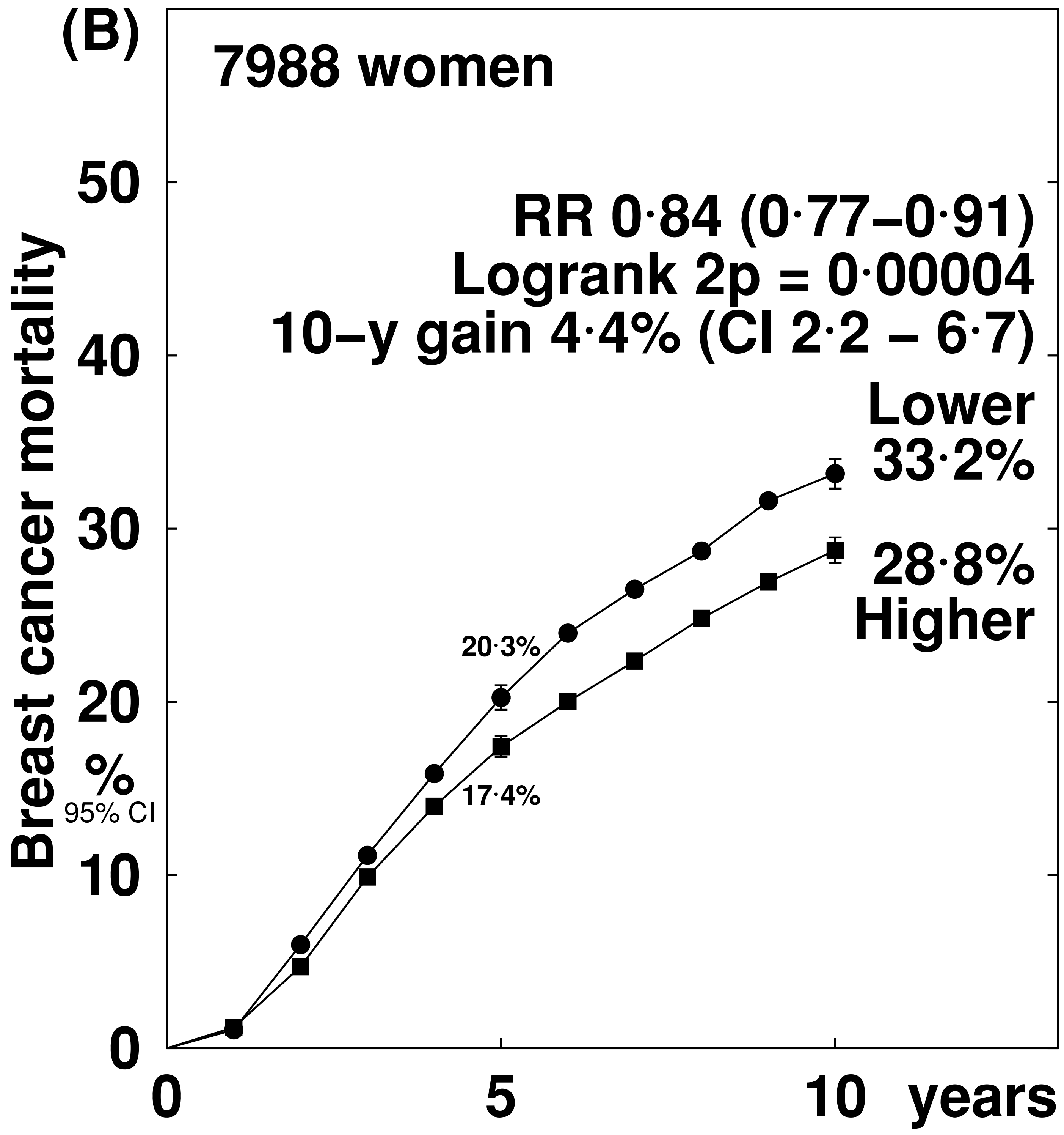

Death rates (% / year: total rate – rate in women without recurrence) & logrank analyses

Allocation	Years 0 – 4	Years 5 – 9	Year 10+
Higher	3·92 CI 3·64 – 4·19	3·06 CI 2·78 – 3·35	1·85 CI 1·59 – 2·11
Lower	4·31 CI 3·98 – 4·64	3·60 CI 3·24 – 3·97	2·26 CI 1·90 – 2·62
Rate ratio, from (O–E) / V	0·84 CI 0·75 – 0·94<br>–52·4 / 308·8	0·83 CI 0·71 – 0·96<br>–33·8 / 176·1	0·87 CI 0·69 – 1·09<br>–10·1 / 72·6

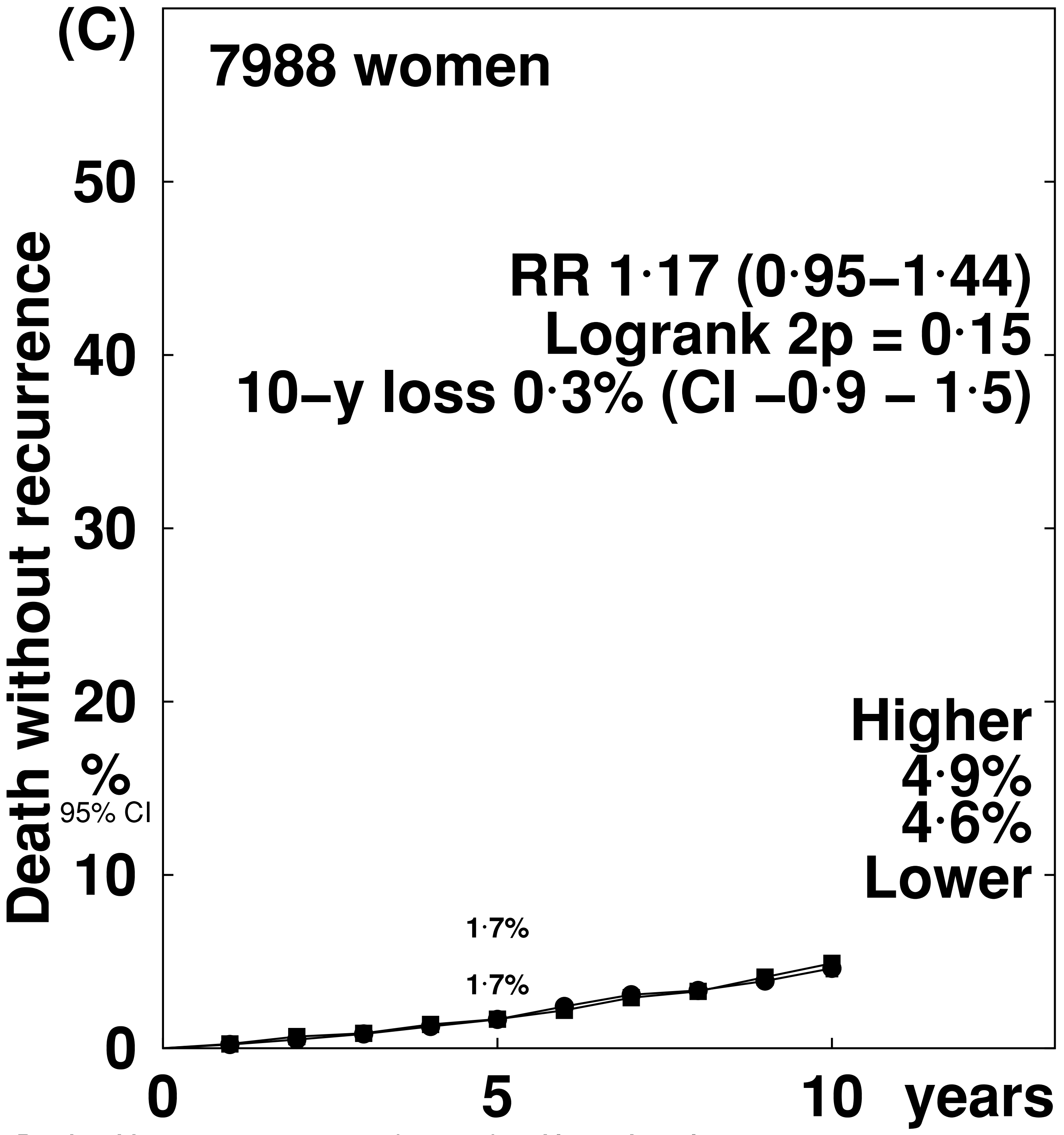

Death–without–recurrence rates (% / year) and logrank analyses

Allocation	Years 0 – 4	Years 5 – 9	Year 10+
Higher	0·35 (64 / 18250)	0·66 (79 / 12039)	1·37 (109 / 7935)
Lower	0·30 (41 / 13625)	0·60 (52 / 8657)	1·03 (54 / 5234)
Rate ratio, from (O–E) / V	1·03 CI 0·68 – 1·54<br>0·6 / 23·3	1·07 CI 0·75 – 1·54<br>2·0 / 29·2	1·38 CI 0·98 – 1·94<br>10·8 / 33·2

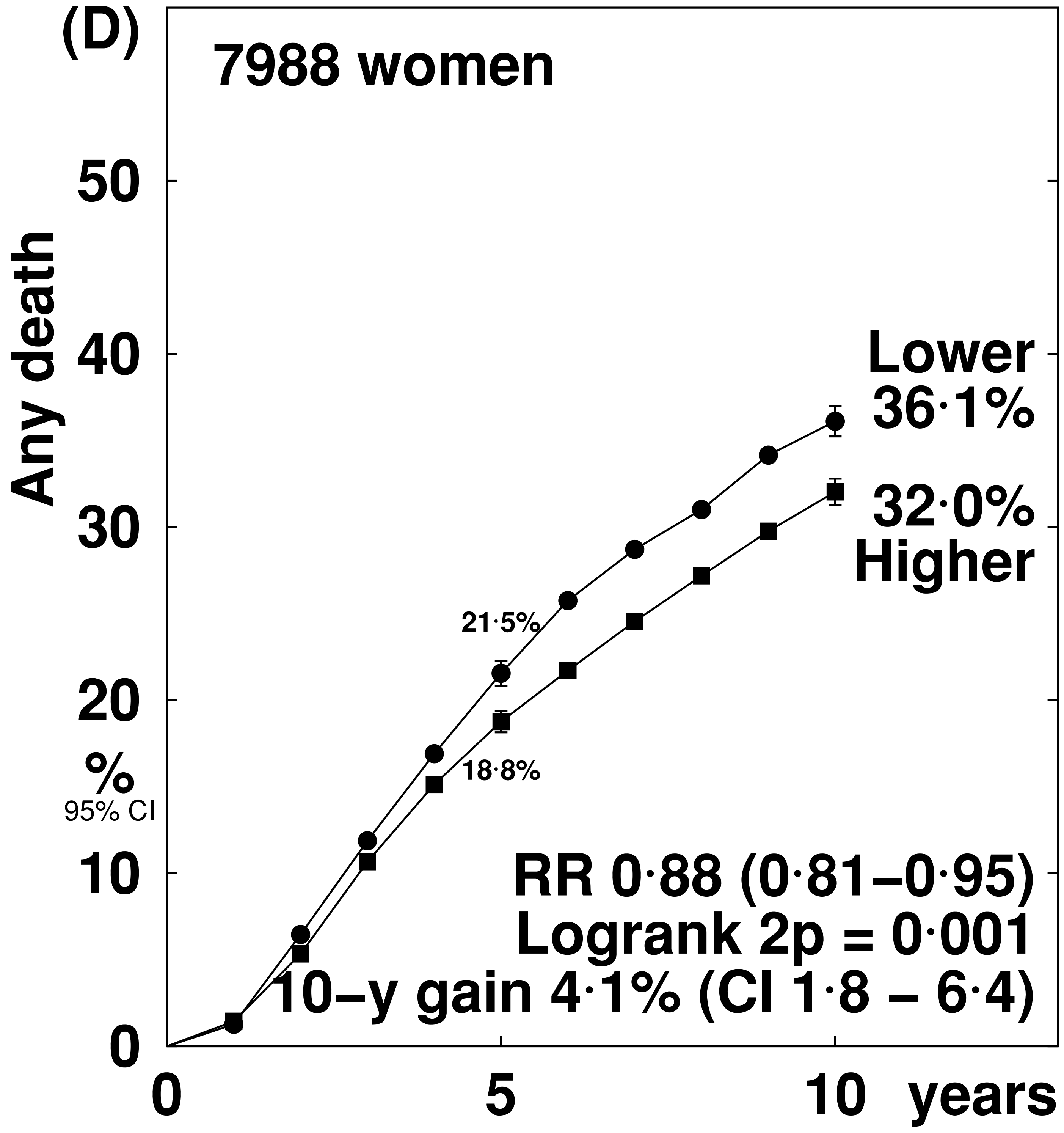

Death rates (% / year) and logrank analyses

Allocation	Years 0 – 4	Years 5 – 9	Year 10+
Higher	4·26 (854 / 20036)	3·69 (520 / 14104)	3·12 (300 / 9604)
Lower	4·60 (703 / 15284)	4·14 (427 / 10314)	3·13 (203 / 6479)
Rate ratio, from (O–E) / V	0·86 CI 0·77 – 0·95<br>–51·8 / 332·1	0·86 CI 0·75 – 0·98<br>–31·8 / 205·3	1·01 CI 0·83 – 1·22<br>0·6 / 105·9

P55-57: Toxicity table 1 – Anthracycline plus taxane versus taxane without anthracycline. Selected reported toxicity data (extracted, where available, from individual trial publications). All columns reported as Taxane plus anthracycline versus Taxane without anthracycline. n= number of patients, (%), NR = not reported

	Trial name	No. pts	All toxicity Grade >3 n (%)	Cardiac and Vascular events Grade >3 n (%)	Peripheral Sensory Neuropathy Grade >2 n (%)	Neutropenia Grade >3 n (%)	Febrile Neutropenia Grade >3 n (%)	Fatigue Grade >3 n (%)	Compliance completed all cycles %	Reference
(a) Concurrent docetaxel + anthracycline vs same dose docetaxel + cyclophosphamide										
07&	**USO 6090/11271** 6($A_{50}D_{75}C_{500}$ vs $D_{75}C_{600}$) q3	1263	350 vs 380 (56.3 vs 59.3)	**Combined:** 25 vs 4 (4.0 vs 0.6)<br>Asystole 1 vs 0<br>Cardiomyopathy/cardiac failure/MI 11 vs 0<br>Hypertension 1 vs 0<br>Tachycardia/arrhythmia 2 vs 2<br>Thromboembolic (including CVA) 10 vs 2	33 vs 40 (5.3 vs 6.2)	213 vs 268 (29.4 vs 41.8)	49 vs 50 (7.9 vs 7.8)	46 vs 19 (7.4 vs 3.0)	NR	Blum et al J Clin Oncol 2017, 35:2647-55
09J	**NSABP-B46i** 6($A_{50}D_{75}C_{500}$ vs $D_{75}C_{600}$) q3	1055	230 vs 201 (43.7 vs 40.0)	**Combined:** 21 vs 7 (4.0 vs 1.3)<br>Cardiac ischaemia 0 vs 0<br>Hypertension 1 vs 0<br>LV dysfunction 5 vs 0<br>Thromboembolic 10 vs 5<br>CNS ischaemia 3 v 0<br>Arrhythmia 3 vs 2	38 vs 47 (7.2 vs 8.9)	76 vs 52 (14.4 vs 9.8)	35 vs 27 (6.7 vs 5.1)	34 vs 23 (6.5 vs 4.4)	NR	Blum et al J Clin Oncol 2017, 35:2647-55
09R	**Shanghai Jiao Tong** 6($A_{50}/E_{60}D_{75}C_{500}$ vs $D_{75}C_{600}$) q3‡	96	NR	NR	NR	40 vs 24 (78.4 vs 53.3)	3 vs 2 (5.9 vs 4.4)	NR	88.2 vs 71	Chen at al Breast Can Res Treat 2013, 142:549-58<br>Chen et al Chin J Can Res 2016, 28:561-9
(b) Sequential taxane plus anthracycline versus higher cumulative dose docetaxel + cyclophosphamide										
08L	**DBCG 07 READ** ($3E_{90}C_{600}$;$3D_{100}$ vs $6D_{75}C_{600}$) q3	2012	NR	**Combined:** 8 vs 8 (0.8 vs 0.8)<br>Cardiomyopathy 1 vs 2<br>Heart failure 7 vs 6	349 vs 297 (35.1 vs 29.5)	NR	102 vs 59 (10.3 vs 5.9)	297 vs 245 (29.9 vs 24.4)	90.7 vs 87.3	Ejlertsen et al J Clin Oncol 2017, 35:2639-46
09&	**Kangawa, Japan** ($3FE_{100}C_{500}$;$3D_{100}$ vs $6D_{75}C_{600}$) q3‡	103	NR	NR	19 vs 19 (36.5 vs 39.6) reported as nerve disorder	46 vs 47 (88.5 vs 97.9)	11 vs 2 (21.2 vs 4.2)	1 vs 1 (1.9 vs 2.1)	94.3 vs 94	Narui et al The Breast 2019, 47:1-9

	Trial name	No. pts	All toxicity Grade ≥3 n (%)	Cardiac and Vascular events Grade ≥3 n (%)	Peripheral Sensory Neuropathy Grade ≥2 n (%)	Neutropenia Grade ≥3 n (%)	Febrile Neutropenia Grade ≥3 n (%)	Fatigue Grade ≥3 n (%)	Compliance completed all cycles %	Reference
09=	**NSABP-B49 (results only as %)** (4A_{60}Cq2/3;12P_{80} q1/4P_{175} q2)/ 6$A_{50}D_{75}C_{500}$ q3 vs 6$D_{75}C_{600}$ q3	1832	(42 vs 40)	LV dysfunction (<1 vs <1) Hypertension (3 vs 3) Thromboembolic event (G3 1, G4< 1 vs G3 < 1 %) Heart failure 0 vs 0	≥G3 (4 vs 1)	G3 (4 vs <1) G4 (1 vs 1)	G3 (3 vs 7) G4 (<1 vs 1)	(4 vs 4)	NR	Blum et al J Clin Oncol 2017, 35:2647-55
09E	**SUCCESS C^** (3$FE_{100}C_{500}$;3D_{100} vs 6$D_{75}C_{600}$) q3	3643	^2245 vs 2089 (76.3 vs 70.1)	NR	^≥G3 45 vs 23 (1.5 vs 0.8)	^1187 vs 1101 (40.3 vs 36.9)	^114 vs 145 (3.9 vs 4.9)	^131 vs 83 (4.4 vs 2.8)	NR	de Gregorio et al Br J Cancer 2022, 126:1715-24
09M	**WSG PLAN-B^** (4$E_{90}C_{600}$;4D_{100} vs 6$D_{75}C_{600}$) q3	2449		**Combined:** 5 vs 6 Heart failure 3 vs 3 Cardiac death 2 vs 2 PE 0 vs 1					87.5 vs 93.0	Nitz et al J Clin Oncol 2019, 37:799-808 de Gregorio et al Br J Cancer 2022, 126:1715-24
09U	**JBCRG-10** (4$D_{75}C_{600}$;4$FE_{100}C_{500}$ or vv vs 6$D_{75}C_{600}$)Trz q3‡	67 (43 vs 24)	24 vs 11 (55.8 vs 45.8)	Heart failure 1 vs 0 (2.3 vs 0) PE 1 vs 0 (2.3 vs 0)	NR	7 vs 4 (16.2 vs 16.7)	11 vs 4 (25.6 vs 16.7)	0 vs 0 (0 vs 0)	NR	Ueno et al Jpn J Clin Oncol 2020, 50:3-11
09V	**JBCRG-09** (3$D_{75}C_{600}$;3$FE_{100}C_{500}$ or vv vs 6$D_{75}C_{600}$) q3‡	195	NR	NR	NR	55 vs 36 (43.0 vs 55.4)	26 vs 9 (20.3 vs 13.8)	1 vs 0 (0.8 vs 0)	95.3 vs 86.1	Ishiguro et al Breast Can Res and Treatment 2020, 180:715-24
10V	**MASTER** (3$FEC_{100}C_{500}$;3D_{100} vs 6$D_{75}C_{600}$) q3	1047	NR	**Combined:** 11 vs 4 (2.1 vs 0.8) Decreased ejection fraction 5 vs 1 (0.9 vs 0.2) Ventricular arrhythmia 5 vs 2 (0.9 vs 0.3) Thrombosis 1 vs 1 (0.2 vs 0.2)	≥G3 4 vs 15 (0.8 vs 2.8)	279 vs 290 (53.4 vs 55.3)	9 vs 11 (1.7 vs 2.0)	24 vs 24 (4.5 vs 4.6)	93.5 vs 94.5	Yu et al Lancet Regional Health 2021, 11:100158

	Trial name	No. pts	All toxicity Grade ≥3 n (%)	Cardiac and Vascular events Grade ≥3 n (%)	Peripheral Sensory Neuropathy Grade ≥2 n (%)	Neutropenia Grade ≥3 n (%)	Febrile Neutropenia Grade ≥3 n (%)	Fatigue Grade ≥3 n (%)	Compliance completed all cycles %	Reference
(c) Taxane plus anthracycline versus taxane +/- capecitabine										
00W	**N-SAS-BC 02 Japan (results only as %)** (4A$_{60}$C6$_{00}$;4P$_{175}$/4D$_{75}$ vs 8P$_{175}$ /D$_{75}$) q3	1049	NR	Cardiac arrhythmia: AC-P vs P: (1.0 vs 0.5) AC-D vs D: (0 vs 1.1)	≥G3 AC-P vs P: (4.2 vs 5.7) AC-D vs D: (0.4 vs 3.8)	AC-P vs P: (17.2 vs 1.9) AC-D vs D: (19.5 vs 6.9)	AC-P vs P: (5.7 vs 0.4) AC-D vs D: (11.1 vs 8.1)	AC-P vs P: (3.8 vs 1.9) AC-D vs D: (3.1 vs 1.9)	87 vs 86	Watanabe et al Cancer 2017, 123:759-68
06Z	**MINDACT** (3FE$_{100}$C$_{500}$;3D$_{100}$ vs 6D$_{75}$Cap$_{1650d1-14}$) q3	392	NR	NR	NR	NR	NR	NR	NR	Cardoso et al NEJM 2016, 375:717-29 Piccart et al Lancet Oncology 2021, 476-88
(d) Taxane plus anthracycline versus taxane + carboplatin										
01M	**BCIRG 006** (4A$_{60}$C$_{600}$;4D$_{100}$ vs 6 D$_{75}$Cpt$_{AUC6}$) q3 +12Trz q1;Trz q3 to 1yr	2149	NR	Congestive heart failure 21 v 4 (2.0 vs 0.4) Hypertension (any grade) 178 vs 190 (16.6 vs 17.7)	Grade ≥1 531 vs 380 (49.7 vs 36.0)	764 vs 696 (71.5 vs 65.9)	116 vs 101 (10.9 vs 9.6)	77 vs 76 (7.2 vs 7.2)	NR	Slamon et al NEJM 2011, 365:1273-83
11R	**PATTERN** 3FE$_{100}$C$_{500}$;3D$_{100}$ q3 vs 6((P$_{80}$Cpt$_{AUC2}$) $_{d1,8,15}$) q4	647	NR	NR	3 vs 12 (0.9 vs 3.7)	297 vs 283 (92.8 vs 87.9)	30 vs 3 (9.4 vs 0.9)	3 vs 3 (0.9 vs 0.9)	94.7 vs 92.9	Yu et al JAMA oncology 2020, 6:1390-6

^ combined results from joint publication

P58: Toxicity table 2 – Trials of docetaxel versus paclitaxel. Selected toxicity reporting extracted from individual trial publications
Where available results presented as n=number, (%), NR = not reported

	Trial name	No. pts	All toxicity Grade >3 n (%)	Peripheral Sensory neuropathy Grade >2 n (%)	Neutropenia Grade >3 n (%)	Febrile neutropenia Grade >3 n (%)	Fatigue Grade >3 n (%)	Dose modification n (%)	Compliance % Completed all taxane cycles	Reference
**Docetaxel q2 or 3 vs Paclitaxel q2 or 3**										
99V	**ECOG E1199 (results only as %)** 4AC;4(D_{100} vs P_{175}) q3	2534	(71 vs 30)	(16 vs 20)	Grade >4 (46 vs 4)	Grade >4 (16 vs <1)	(9 vs 2)	(28 vs 22)	87 vs 95	Sparano et al. NEJM 2008, 358:1663-71
00W	**N-SAS BC02 (results only as %)** 4AC/EC;4(D_{75} vs P_{175}) q3	525	NR	Grade >3 (0.4 vs 4.2)	(19.5 vs 17.2)	(11.1 vs 5.7)	(3.1 vs 3.8)	NR	86.7 vs 86.7	Watanabe et al Cancer 2017, 123:759-68
00W	**N-SAS BC02 (results only as %)** 8(D_{75} vs P_{175}) q3	524	NR	Grade >3 (3.8 vs 5.7)	(6.9 vs 1.9)	(8.1 vs 1.9)	(1.9 vs 1.9)	NR	84.7 vs 86.7	Watanabe et al Cancer 2017, 123:759-68
04R	**HORG CT-04** 4FE_{75}C;4(D_{75} vs P_{175}) q2	481	NR	7 vs 11 (2.9 vs 4.6)	74 vs 51 (31 vs 23)	4 vs 3 (1.7 vs 1.2)	18 vs 20 (7.5 vs 8.3)	(1.7 vs 2.8)	96.3 vs 90	Saloustros et al Br Can Res Trt 2014, 148:591-7
**Docetaxel q1 vs paclitaxel q1**										
99V	**ECOG E1199 (results only as %)** 4AC;12(D_{35} vs P_{80}) q1	2518	(45 vs 28)	(16 vs 27)	>Grade 4 (3 vs 2)	>Grade 4 (1 vs 1)	(11 vs 3)	(40 vs 29)	75 vs 88	Sparano et al. NEJM 2008, 358:1663-71
05C	**HE10/05** 3E_{110} q2;3CMF; 9(D_{35} vs P_{80}) q1	664	NR	Grade >3 neurology 4 vs 4 (1.3 vs 1.3)	85 vs 85 (26.9 vs 26.6)	14 vs 17 (4.4 vs 5.3)	7 vs 10 (2.2 vs 3.1)	NR	88.3 vs 88	Fountzilas et al. BMC Cancer 2014, 14:515

P59-60: Toxicity table 3 – Trials of paclitaxel or docetaxel in smaller fractions versus larger fractions. Selected toxicity reporting extracted from individual trial publications
Where available results presented as n=number, (%), NR = not reported

	Trial name	No. pts	All toxicity Grade ≥3 n (%)	Peripheral Sensory neuropathy Grade ≥2 n (%)	Neutropenia Grade ≥3 n (%)	Febrile neutropenia Grade ≥3 n (%)	Fatigue Grade ≥3 n (%)	Dose modification n (%)	Compliance (%) Completed all taxane cycles	Reference
**Paclitaxel q1 versus Paclitaxel q2 or q3**										
98K	**MDA 98-240 (results only as %)**<br>($12P_{80/150/175}$q1 vs $4P_{225}$ q3);4 FAC q3	254	NR	(36.5* vs 51.2)<br>*80mg/m^2 dose	NR	(1 vs 9.5)	NR	NR	NR	Green et al. J Clin Oncol 2005, 23:5983-92
99V	**ECOG E1199 (results only as %)**<br>4AC; ($12P_{80}$q1 vs $4P_{175}$ q3)	2530	(28 vs 30)	(27 vs 20)	Grade ≥4<br>(2 vs 4)	Grade ≥4<br>(1 vs <1)	(3 vs 2)	(29 vs 22)	88.0 vs 95.0	Sparano et al. NEJM 2008, 358:1663-71
03*	**SWOG S0221**<br>($15A_{24}C_{60dq1-7}$/ 6AC)q2; ($12P_{80}$ q1 v $6P_{175}$ q2)	3294	414 vs 412<br>(36.3 vs 35.5)	Grade ≥3<br>119 vs 201<br>(10.5 vs 17.3)<br>(reported as neurology)	139 vs 22<br>(12.2 vs 1.9)*<br>*GCSF in q2 arm	1 vs 2<br>(0.1 vs 0.2)	NR	NR	75.0 vs 71.0	Budd et al. J Clin Oncol 2015, 33:58-64
05C	**HE10/05**<br>$3E_{110}$ q2;(3CMF q2; $9P_{80}$q1 vs $3P_{200}$ q2; 3CMF q2)	667	NR	Grade ≥3<br>4 vs 16<br>(1.3 vs 4.9)<br>(reported as neurology)	85 vs 99<br>(26.6 vs 30.4)	17 vs 20<br>(5.3 vs 6.1)	10 vs 4<br>(3.1 vs 1.2)	NR	88.0 vs 93.6	Fountzilas et al. BMC Cancer 2014, 14:515
**Docetaxel q1 or q2 vs Docetaxel q3**										
99V	**ECOG E1199**<br>4AC; ($12D_{35}$ q1 vs $4D_{100}$ q3)	2522	(45 vs 71)	(16 vs 16)	Grade ≥4<br>(3 vs 46)	Grade ≥4<br>(1 vs 16)	(11 vs 9)	(40 vs 28)	75.0 vs 87.0	Sparano et al. NEJM 2008, 358:1663-71
O0^	**U Hull / Lincoln CH**<br>4AC; ($12D_{33}$ q1 vs $4D_{100}$ q3)	82	NR	0 vs 16<br>(0 vs 38.2)<br>Grade not reported	5/82 overall<br>(6)	4/82<br>(5)	4 vs 7<br>(10 vs 17)<br>Grade not reported	NR	91.1 vs 93.2	Walker et al. BMC Cancer 2011, 11:179
04Z	**BREAST-10**<br>$12E_{30}D_{35}$ q1 vs $4E_{75}D_{80}$ q3<br>*reported for whole trial (n=135) including patients	135 (44 in outcomes analysis)	NR	2 vs 1<br>(3 vs 1.4)	11 vs 45<br>(16.7 vs 65.2)	0 vs 2<br>(0 vs 2.9)	1 vs 0<br>(1.4 vs 0)	NR	NR	Nuzzo et al. BMC Cancer 2011, 11:75

	with metastatic disease									
05@	**UZ Leuven Belgium** ($4F_{375}E_{75}C_{375}$;$4D_{75}$ or vv) q2 vs ($3F_{500}E_{100}C_{500}$; $3D_{100}$ or vv) q3	85	24 vs 15 (30.7 vs 38.4)	NR	5 vs 8 (6.4 vs 21)	0 vs 2 (0 vs 5.1)	8 vs 0 (7.7 vs 0)	10 vs 3 (12.8 vs 7.9)	94.8 vs 97.4	Wildiers et al Breast Can Res Trt 2009, 114:103-12

Statistical Analysis Plan for a Meta-Analysis of randomised trials comparing anthracycline and taxane containing chemotherapy regimens for early breast cancer

This meta-analysis aims to compare the long-term benefits and risks of different anthracycline and taxane containing chemotherapy regimens for early breast cancer

Cohort definitions

The current list of trials contributing to these 8 cohorts are shown in Appendix 1.

Cohort 1. Taxane with and without anthracycline: randomised trials that compare any taxane plus anthracycline regimen versus any taxane regimen without anthracycline.

Cohort 2. Anthracycline with taxane versus anthracycline regimens without taxane: randomised trials that compare any anthracycline plus taxane regimen versus any anthracycline regimen without taxane.

Cohort 3. Taxane versus anthracycline: randomised trials that compare any taxane regimen wihout anthracycline versus any anthracycline regimen without taxane.

Cohort 4. Head-to-head taxane comparisons: randomised trials with head-to-head comparisons between different taxanes (docetaxel versus paclitaxel).

Cohort 5. Dose fractionation: randomised trials that compare taxane regimens with similar cumulative doses but different scheduling (i.e. small more frequent versus larger less frequent).

Cohort 6. Sequencing of sequential anthracycline and taxane chemotherapy: randomised trials of anthracycline then taxane versus the same drug regimens in the opposite order.

Cohort 7. Longer versus shorter duration of anthracycline: randomised trials comparing longer versus shorter duration of anthracycline based chemotherapy.

Cohort 8. Higher versus lower dose of anthracycline: randomised trials comparing higher versus lower doses of anthracycline chemotherapy.

Analysis population

The analysis population will be women with early stage (i.e. operable) or locally advanced breast cancer where treatment is administered with curative intent. Data will be sought from all relevant randomised trials with greater than 10 women randomised, irrespective of primary outcome measure.

Data checking

The usual EBCTCG quality assurance checks for range, consistency and balance between randomisation arms will be undertaken prior to analysis. The dependence of deaths without recorded recurrence and of second cancers (overall and by site) on TN status will also be investigated with any association used to estimate what proportion might actually have been from breast cancer. Checks will be undertaken to compare the incidence of death without recurrence and of second cancers (overall and by site) by age group in each trial to characterise whether these might include miscoded breast cancer events.

Analyses

Primary analyses will be by Intention to Treat (ITT), including all randomised patients irrespective of treatment compliance.

The data variables to be requested from each trial are detailed in Appendix 2.

Primary outcomes

The main endpoint definitions and methods are those used in previous EBCTCG reports.

1. Time to recurrence: includes distant recurrence, invasive loco-regional recurrence and new second primary invasive breast cancer (ipsilateral or contralateral), and the definitions of these will be as in each trial.

2. Breast cancer mortality: information about mortality rates without recurrence will be subtracted from information about overall mortality rates, estimated by log-rank subtraction (as in previous EBCTCG reports). The same statistical methods will be used to construct Kaplan-Meier graphs that estimate breast cancer mortality (i.e. the pattern of

mortality that would have been seen if it had been possible to avoid all deaths before or after recurrence from causes other than breast cancer).

3. Death without recurrence: i.e. without the EBCTCG secretariat having any record of recurrence. The quality of the recurrence data in these trials is likely to be reasonably good, deaths from wholly unknown cause without recorded recurrence will be treated as death from an unknown cause that was not breast cancer.

4. All-cause mortality

Exploratory endpoints

1. Time to first distant recurrence: includes distant recurrence and ignores any prior loco-regional or contralateral recurrences.

2. Time to loco-regional recurrence as first event: includes ipsilateral breast, chest wall and loco-regional lymph nodes (axilla and SCF).

3. Time to new contralateral recurrence

4. AML incidence

5. Incidence and site of second cancers: Information on the site of other non-breast second primary before any recurrence of breast cancer will be collected. Categorisation of sites will include but is not limited to: haematological, lung, colorectal, GI, gynaecological, urological.

6. Safety – Data on other non-fatal adverse events (e.g. ischaemic heart disease, stroke, pulmonary embolus, DVT) will be sought and analysed.

Subgroup Analyses

Pre-specified subgroup analyses will be undertaken but, given the well-known hazards of subgroup analysis, will be interpreted appropriately cautiously. Investigation of potential interactions between tumour or patient characteristics and treatment efficacy will be undertaken with breast cancer recurrence as primary outcome.

Subgroup Analyses of Recurrence Forest plots for subgroup analyses by:

- Site of first recurrence (distant metastasis, invasive loco-regional recurrence or contralateral breast cancer)
- Period of follow-up (years 0-1, 2-4, 5-9, and 10+ after randomisation)
- Age (<45, 45-54, 55-64, 65+);
- ER status (ER-, ER+, ER unknown);
- ER/PR status (ER+/PR- vs ER+/PR+)
- Nodal status (N0/N-, N1-3, N4+, N unknown);
- Tumour size (T1, T2, T3/T4);
- Tumour grade (well-differentiated, moderately differentiated, poorly differentiated);
- Proliferation index (%Ki-67 0-9, 10-19, 20-49, 50+)
- HER2 status (HER2-, HER2+, unknown)
- Tumour histology (ductal, lobular, other, unknown)
- BMI (<20, 20-<25, 25-<30, 30+)

Statistical analyses

If a log-rank statistic (observed [o]-expected [e]) have variance v, then defining z=(o-e)/sqrt(v) and b=(o-e)/v, where b has variance 1/v, the outcome RR is estimated as exp(b) with SE=(RR-1)/z. 95% CIs and 99% CIs for RRs are derived from those for b (by normal approximations). Two-sided significance with p values of less than 0.05 were considered significant for analyses of the primary and secondary outcomes, and to compensate for multiple investigations, p values of less than 0.01 were considered significant for subgroup analyses.

Tests of heterogeneity and of trend

First calculate the log-rank statistic (o-e) and its variance v in each separate stratum, and add these up to get the overall logrank (O-E) and it variance V (i.e. the sum of the separate variances). Delete any uninformative strata (i.e. those for which v is zero), and number the remaining strata from 1 to n. A χ^2 test (on n-1 degrees of freedom) for

heterogeneity between the treatment effects in different strata can be obtained by subtracting $(O\text{-}E)^2/V$ from the sum of the separate values, one per stratum, of $(o\text{-}e)^2/v$.

Alternatively, a χ^2 test for trend (i.e. for whether the treatment effect changes progressively from one stratum to the next) will be calculated as follows: if the stratum numbered s has logrank statistics (o-e) and v then define m, the mean stratum number, to be the sum, one term per stratum, of sv/V and define T to be the sum, one term per stratum, of (s-m)(o-e). The variance of T, var(T), is then the sum, one term per stratum, of $(s\text{-}m)^2v$, and the χ^2 test (on 1 degree of freedom) for trend is $T^2/var(T)$. If there are only two strata then the tests for trend and heterogeneity are identical.

Appendix 1. Taxane trial list, updated to November 2019

Cohort1: Taxane + Anthracycline vs Taxane without Anthracycline

Trial & year code	Trial name	Comparisons	N	Received	Published
**(a) Concurrent docetaxel plus anthracycline versus same dose docetaxel plus cyclophosphamide**					
34206 2007&	USO 06090 / 11271, USA	(Docetaxel [75 mg/m2 iv] d1 + Doxorubicin [50 mg/m2 iv] d1 + Cyclophosphamide [600 mg/m2 iv] d1) q3wk × 6 vs (Docetaxel [75 mg/m2 iv] d1 + Cyclophosphamide [600 mg/m2 iv] d1) q3wk × 6	1296	Yes	✓ 2017[1]
4772 2009J1	NSABP B-46-I, USA	Docetaxel [75 mg/m2 iv] + Doxorubicin [50 mg/m2 iv] + Cyclophosphamide [500 mg/m2] + pegfilgrastim [6 mg sc d2] q21d × 6 vs Docetaxel [75 mg/m2 iv] + Cyclophosphamide [600 mg/m2] q21d × 6	1077	Yes	✓ 2017[1]
46701 2009R	NATT / NCT00912444, Shanghai Jiao Tong University School of Medicine, China	(Docetaxel [75mg/m2 iv] + (Doxorubicin [50mg/m2 iv] or Epirubicin [60 mg/m2 iv]) + Cyclophosphamide [500 mg/m2 iv] + G-CSF) q3wk × 6 preoperative vs (Docetaxel [75mg/m2 iv] + Cyclophosphamide [600 mg/m2 iv] + G-CSF) q3wk × 6 preoperative	96	Yes	✓ 2016[2]
**(b) Sequential taxane plus anthracycline versus higher cumulative dose docetaxel plus cyclophophamide**					
38406 2007I	HORG CT/07.17, Greece	(5-fluorouracil [500mg/m2] + Epirubicin [75mg/m2] + Cyclophosphamide [500mg/m2]) q3wk × 4 then Docetaxel [75mg/m2]q2wk × 4 vs (Docetaxel [75mg/m2] + Cyclophosphamide [500mg/m2]) q2wk × 6	650	Yes - ineligible	✓2016[116]
2225 2008L	DBCG 07 READ, Denmark	(Epirubicin [90 mg/m2 iv] + Cyclophosphamide [600 mg/m2 iv]) q3wk × 3 then docetaxel [100 mg/m2 iv] q3wk × 3 vs (Cyclophosphamide [600 mg/m2 iv] + Docetaxel [75 mg/m2 iv]) q3wk × 6	2012	Yes	✓ 2017[3]
50702 2008]	Shiga Anthracycline trial	Docetaxel [100mg/m2] q3wk × 3 then (5-Fluorouracil [500 mg/m2] + Epirubicin [100 mg/m2] + Cyclophosphamide [500 mg/m2]) q3wk × 3 vs (Docetaxel [75mg/m2] + Cyclophosphamide [600 mg/m2]) q3wk × 6	42	No - ineligible	✓ 2013[4]
51701 2009&	Kanagawa Japan	(5-Fluorouracil [500 mg/m2 iv d1] + Epirubicin [100 mg/m2 d1] + Cyclophosphamide [500 mg/m2 iv d1]) q21d × 3 then Docetaxel [100mg/m2 iv d1] q21d × 3 preoperative vs (Cyclophosphamide [600mg/m2 iv d1] + Docetaxel [75 mg/m2]) q21d × 6 preoperative	103	Yes	✓ 2019[5]
4774 2009J2	NSABP B-49, USA	((Docetaxel [75 mg/m2] + doxorubicin [50 mg/m2] + cyclophosphamide [500 mg/m2 iv]) q3wk × 6) or ((Doxorubicin [60 mg/m2] + cyclophosphamide [600 mg/m2 iv]) q3wk × 4 then paclitaxel [80 mg/m2 iv] qwk × 12) or ((Doxorubicin [60 mg/m2] + cyclophosphamide [600 mg/m2 iv]) q2wk × 4 then paclitaxel [80 mg/m2 iv] qwk × 12) or ((Doxorubicin [60 mg/m2] + cyclophosphamide [600 mg/m2 iv]) q2wk × 4 then paclitaxel [175 mg/m2 iv] q2wk × 4) vs Docetaxel [75 mg/m2 iv] + Cyclophosphamide [600 mg/m2] q21d × 6	1870	Yes	✓ 2017[1]
33810 2009E1	SUCCESS C, Ludwig-Maximilians University, Germany	(5-Fluorouracil [500 mg/m2 iv] + Epirubicin [100 mg/m2 iv] + Cyclophosphamide [500 mg/m2 iv]) q3wk × 3 then Docetaxel [100 mg/m2 iv] q3wk × 3 vs (Docetaxel [75 mg/m2 iv] + Cyclophosphamide [600 mg/m2 iv]) q3wk × 6	3643	Yes	✓ 2018[6]

30906 2009M	WSG Plan B, Germany	(Epirubicin [90 mg/m2] + Cyclophosphamide [600 mg/m2]) q3wk × 4 then Docetaxel [100 mg/m2] q3wk × 4 vs (Docetaxel [75 mg/m2] + Cyclophosphamide [600 mg/m2]) q3wk × 6	2449	Yes	✓ 2019[7]
48102 2009U	JBCRG-10, Japan Breast Cancer Research Group	(5-fluorouracil [500mg/m2] + epirubicin [100mg/m2] + cyclophosphamide [500mg/m2] q3wk) × 4 then (docetaxel [75mg/m2] + cyclophosphamide [600mg/m2] q3wk + trastuzumab [4mg/kg loading dose then 2mg/kg thereafter] qwk or [8mg/kg loading dose then 6mg/kg thereafter q3wk]) × 4 preoperative or (docetaxel [75mg/m2] + cyclophosphamide [600mg/m2] q3wk + trastuzumab [4mg/kg loading dose then 2mg/kg thereafter] qwk or [8mg/kg loading dose then 6mg/kg thereafter q3wk]) × 4 then (5-fluorouracil [500mg/m2] + epirubicin [100mg/m2] + cyclophosphamide [500mg/m2] q3wk) × 4 preoperative vs (docetaxel [75mg/m2] + cyclophosphamide [600mg/m2] q3wk + trastuzumab [4mg/kg loading dose then 2mg/kg thereafter] qwk or [8mg/kg loading dose then 6mg/kg thereafter q3wk]) × 4 then (5-fluorouracil [500mg/m2] + epirubicin [100mg/m2] + cyclophosphamide [500mg/m2] q3wk) × 4 preoperative	67	Yes	✓ 2020[8]
48103 2009V	JBCRG-09, Japan Breast Cancer Research Group	(5-Fluoro-Uracil [500 mg/m2] + Epirubicin [100 mg/m2] + Cyclophosphamide [500 mg/m2]) q3wk × 3 then (Docetaxel [75 mg/m2] + Cyclophosphamide [600 mg/m2]) q3wk × 3 preoperative or (Docetaxel [75 mg/m2] + Cyclophosphamide [600 mg/m2]) q3wk × 3 then (5-Fluoro-Uracil [500 mg/m2] + Epirubicin [100 mg/m2] + Cyclophosphamide [500 mg/m2]) q3wk × 3 preoperative vs (Docetaxel [75 mg/m2] + Cyclophosphamide [600 mg/m2]) q3wk × 6 preoperative	195	Yes	✓ 2020[9]
36509 2010V	Fudan BC MASTER / NCT01314833, Fudan University, Shanghai, China	(Cyclophosphamide [500 mg/m2 d1] + Epirubicin [90-100 mg/m2 d1] + Fluorouracil [500 mg/m2 d1]) q21d × 3 then Docetaxel [100 mg/m2 d1] q21d × 3 vs (Cyclophosphamide [600 mg/m2 d1] + Docetaxel [75 mg/m2 d1]) q21d × 6	1047	Yes	✓ 2021[10]
**(c) Taxane plus anthracycline versus taxane ± capecitabine**					
35901 2000W	N-SAS BC 02, Japan	((Doxorubicin [60mg/m²] or Epirubicin [75mg/m2]) + Cyclophosphamide [600mg/m²]) q3wk × 4 then (Paclitaxel [175mg/m²] or Docetaxel [75mg/m²]) q3wk × 4 vs (Paclitaxel [175mg/m²] or Docetaxel [75mg/m²]) q3wk × 8	1060	Yes	✓ 2017[11]
36901 2001V	Multicentre France Neoadjuvant Trial, France	(Epirubicin[100mg/m²] + Docetaxel [75mg/m²] + filgrastim) q3wk × 6 maximum preoperative vs Docetaxel [100mg/m²] q3wk × 8 maximum preoperative	21	No	✓ 2004[12]
54201 2005]	Asan Neoadjuvant Capecitabine trial, University of Ulsan College of Medicine, Seoul, Korea	(Doxorubicin [60 mg/m2 d1] + Cyclophosphamide [600 mg/m2 d1]) q3wk × 4 then Docetaxel [75 mg/m2] q3wk × 4 preoperative vs (Capecitabine [2000 mg/m2 d1-14] + Vinorelbine [25 mg/m2 d1,8]) q 3wk × 4 then Docetaxel [75 mg/m2] q3wk × 4 preoperative	75	No	✓ 2015[13]
6332 2006Z1&3	EORTC 10041 / BIG 3-04 / MINDACT, Belgium	(Fluorouracil [500 mg/m2 iv] + Epirubicin [100 mg/m2 iv] + Cyclophosphamide [500 mg/m2 iv]) d1 q3wk × 3; Docetaxel [?? mg/m2 iv d1] q3wk × 3 vs Docetaxel [75 mg/m2 iv d1] + Capecitabine [1650 mg/m2 po d1-14] q3wk × 6	392	Yes	✓ 2021[14]
37004 2009[2	Remagus 04, France	FEC × 4 then Docetaxel × 4 preoperative vs Docetaxel-capecitabine × 6 preoperative	100	No	2012[15]
52106 2009~6	TaiNAC, Taiwan	(Docetaxel [35 mg/m2 iv] + Epirubicin [45 mg/m2 iv]) d1+8 × 4 preoperative vs (Docetaxel [35 mg/m2] + 5-Fluorouracil [2000mg/m2] + Leucovorin [300 mg/m2]) d1+8 × 4 preoperative	10	Yes – ineligible	No

2630 2011K	ABCSG-32, Austria	(Docetaxel [100 mg/m2 iv] + Pegfilgrastim [6mg sc d2] + Trastuzumab [8mg/kg loading dose then 6mg/kg iv]) + non-pegylated liposomal Doxorubicin [50mg/m2 iv] q21d × 6 preoperative or (Docetaxel [100 mg/m2 iv] + Pegfilgrastim [6mg sc d2] + Trastuzumab [8mg/kg loading dose then 6mg/kg iv] + non-pegylated liposomal Doxorubicin [50mg/m2 iv] + Bevacizumab [15 mg/kg]) q21d × 6 preoperative vs (Docetaxel [100 mg/m2 iv] + Pegfilgrastim [6mg sc d2] + Trastuzumab [8mg/kg loading dose then 6mg/kg iv] + non-pegylated liposomal Doxorubicin [50mg/m2 iv] + Bevacizumab [15 mg/kg]) q21d × 6 preoperative or (Docetaxel [100 mg/m2 iv] + Pegfilgrastim [6mg sc d2] + Trastuzumab [8mg/kg loading dose then 6mg/kg iv] + Bevacizumab [15 mg/kg]) q21d × 6 preoperative	100	Yes - ineligible	✓ Abstract 2015[16]
54101 2011N	PUMCH-Breast-TCX / NCT01354522, Beijing	Docetaxel [75 mg/m2 iv d1] + (Doxorubicin [50 mg/m2 iv d1] or Epirubicin [75 mg/m2 iv d1]) + Cyclophosphamide [500 mg/m2 iv d1] q3wk × 6 vs Docetaxel [75 mg/m2 iv d1] + Cyclophosphamide [500 mg/m2 iv d1] + Capecitabine [950 mg/m2 bid po d1-14] q3wk × 6	400	No	No
**(d) Taxane plus anthracycline versus taxane plus carboplatin (confounded)**					
33505 2001M	BCIRG 006, TRIO (formerly Breast Cancer International Research Group)	(Doxorubicin [60mg/m²] + Cyclophosphamide [600mg/m²]) q21d × 4 then (Docetaxel [100mg/m²] q21d × 4 + Trastuzumab [4-2mg/kg/wk] qwk × 12) then Trastuzumab [6mg/kg] q21d until 1 yr vs (Docetaxel [75mg/m²] + (Cis-Platinum [75mg/m²] or Carboplatin [AUC 6])) q21d × 6 + Trastuzumab [4-2mg/kg/wk] qwk × 18 then Trastuzumab [6mg/kg] q21d until 1 yr	2149	Yes	✓ 2011[18]
22311 2006~	CAMS Neoadjuvant Paclitaxel trial, Beijing, China	(Paclitaxel [175 mg/m2 iv d1] + Epirubicin [75 mg/m2 iv d1]) q21d × 2-6 cycles preoperative vs (Paclitaxel [175 mg/m2 iv d1] + carboplatin [AUC 5 iv d2]) q21d × 2-6 cycles preoperative	80	No	✓ 2016[19]
43903 2009T	TRYPHAENA, Basel, Switzerland	(Pertuzumab [840 mg then 420 mg iv q3wk] + Trastuzumab [8mg/kg iv then 6mg/kg q3wk iv]) × 6 + ((5-Fluoro-Uracil [500 mg/m2 iv] + Epirubicin [100 mg/m2 iv] + Cyclophosphamide [500 mg/m2 iv]) for cycles 1-3 then Docetaxel [75 mg/m2 iv then 100 mg/m2 iv q3wk] for cycles 4-6) preoperative then Trastuzumab [6mg/kg iv] q3wk to 1 yr vs (Trastuzumab [8mg/kg iv then 6mg/kg iv] + Pertuzumab [840 mg then 420 mg iv] + Carboplatin [AUC6] + Docetaxel [75 mg/m2]) q3w × 6 preoperative then Trastuzumab [6mg/kg iv] q3wk to 1 yr	225	No	✓ 2013[20]
52107 2009~7	TaiNAC, Taiwan	(Docetaxel [35 mg/m2 iv] + Epirubicin [45 mg/m2 iv]) d1+8 × 4 preoperative vs (Docetaxel [35 mg/m2] + Cisplatin [35 mg/m2 iv]) d1+8 × 4 preoperative	8	Yes – ineligible	No
22309 2010U	CH-BC-007 / NCT01150513, Beijing, China	Epirubicin [90 mg/m2 d1] + Cyclophosphamide [600 mg/m2 d1] q3wk × 4 then Docetaxel [100 mg/m2 d1] q3wk × 4 vs Docetaxel [75 mg/m2 d1] + Carboplatin [AUC 5 d1] q3wk × 6	298	No	✓ 2016[21]
36505 2011H	Fudan Pay/NCT01428414, Fudan University, Shanghai, China	(Trastuzumab [4 mg/kg loading dose then 2 mg/kg iv] + paclitaxel [75 mg/m2]) qwk × 3 + epirubicin [75 mg/m2] q3wk × 4 or 6 cycles preoperative vs (Trastuzumab [4 mg/kg loading dose then 2 mg/kg iv] + paclitaxel [75 mg/m2] + carboplatin [AUC 2]) qwk × 12 or 18 preoperative	100	No	2015[22]
22310 2011M	CH-BC-012 / NCT01378533, Beijing, China	(Epirubicin [80 mg/m2 iv d1 or divided over 2d] + Cyclophosphamide [600 mg/m2 iv d1] + G-CSF [3 mcg/kg d5-9]) q14d × 4 then (Paclitaxel [175 mg/m2 iv d1] + G-CSF [3 mcg/kg d5-9]) q14d × 4 vs (Paclitaxel [150 mg/m2 iv d1] + Carboplatin [AUC=3 iv d2] + G-CSF [3 mcg/kg d5-9]) q14d × 8	132	No	✓ Abstract 2019[17]

36510<br>2001R	Fudan PATTERN / NCT01216111, Fudan University, Shanghai, China	Cyclophosphamide [500mg/m2] + Epirubicin [100mg/m2] + Fluorouracil [500mg/m2] q3wk × 3 then Docetaxel [100mg/m2wk] q3wk × 3 vs Paclitaxel [80mg/m2 d1,8,16] + Carboplatin [AUC = 2 d1,8,16] q4wk × 6	647	Yes	✓ 2020[23]

Cohort 2: Anthracycline with taxane versus anthracycline regimens without taxane

Trial & year code	Trial name	Comparisons	N	Received	Published
**(a) Taxane plus anthracycline based regimen vs the SAME non-taxane cytotoxic chemotherapy**					
8316<br>1994D	CALGB Study 9344 / Intergroup 0148	(Cyclophosphamide [600mg/m²] + Doxorubicin [90mg/m²] + filgrastim [5 or 10µg/kg/d sc d2-12]) q3wk × 4 then Paclitaxel [175mg/m²] q3wk × 4 vs (Cyclophosphamide [600mg/m²] + Doxorubicin [90mg/m²] + filgrastim [5 or 10µg/kg/d sc d2-12]) q3wk × 4<br>Or<br>(Cyclophosphamide [600mg/m²] + Doxorubicin [75mg/m²]) q3wk × 4 then Paclitaxel [175mg/m²] q3wk × 4 vs (Cyclophosphamide [600mg/m²] + Doxorubicin [75mg/m²]) q3wk × 4<br>Or<br>(Cyclophosphamide [600mg/m²] + Doxorubicin [60mg/m²]) q3wk × 4 then Paclitaxel [175mg/m²] q3wk × 4 vs (Cyclophosphamide [600mg/m²] + Doxorubicin [60mg/m²]) q3wk × 4	3170	Yes	✓ 2003[24]
4747<br>1995J	NSABP B-27, USA	((Doxorubicin [60mg/m²] + Cyclophosphamide [600mg/m²]) q21d × 4 preoperative then Docetaxel [100mg/m²] q21d × 4 preoperative then (Lumpectomy + RT) or Mastectomy) + Tamoxifen [20mg/d] × 5yr or ((Doxorubicin [60mg/m²] + Cyclophosphamide [600mg/m²]) q21d × 4 preoperative then (Lumpectomy + RT) or Mastectomy) then Docetaxel [100mg/m²] q21d × 4 + Tamoxifen [20mg/d] × 5yr vs ((Doxorubicin [60mg/m²] + Cyclophosphamide [600mg/m²]) q21d × 4 preoperative then (Lumpectomy + RT) or Mastectomy) + Tamoxifen [20mg/d] × 5yr	2411	Yes	✓ 2003[25]
4748-50<br>1995K	NSABP B-28, USA	(Doxorubicin [60mg/m²] + Cyclophosphamide [600mg/m²]) q21d × 4 then Paclitaxel [225mg/m²] q21d × 4 then Tamoxifen [20mg/d] × 5yr vs (Doxorubicin [60mg/m²] + Cyclophosphamide [600mg/m²]) q21d × 4 then Tamoxifen [20mg/d] × 5yr<br>Or<br>(Doxorubicin [60mg/m²] + Cyclophosphamide [600mg/m²]) q21d × 4 then Paclitaxel [225mg/m²] q21d × 4 vs (Doxorubicin [60mg/m²] + Cyclophosphamide [600mg/m²]) q21d × 4<br>Or<br>(Doxorubicin [60mg/m²] + Cyclophosphamide [600mg/m²]) q21d × 4 then Paclitaxel [225mg/m²] q21d × 4 then Tamoxifen [20mg/d] × 5yr vs (Doxorubicin [60mg/m²] + Cyclophosphamide [600mg/m²]) q21d × 4 then Tamoxifen [20mg/d] × 5yr	3060	Yes	✓ 2005[26]

4312 1998B	Taxit216, Italy	Epirubicin[120mg/m² iv d1] q21d × 4 then Docetaxel [100mg/m² iv d1] q21d × 4 then (Cyclophosphamide [600mg/m² iv d1,8] + Methotrexate [40mg/m² ivd1,8] + 5-Fluoro-Uracil [600mg/m² iv d1,8]) q28d × 4 vs Epirubicin[120mg/m² iv d1] q21d × 4 then (Cyclophosphamide [600mg/m² iv d1,8] + Methotrexate [40mg/m² ivd1,8] + 5-Fluoro-Uracil [600mg/m² iv d1,8]) q28d × 4	972	Yes	✓ Abstract 2008[27]
34301 1999T	GOIM 9902, Italy	Docetaxel [100mg/m² iv d1] q21d × 4 then (Epirubicin[120mg/m² iv d1] + Cyclophosphamide [600mg/m² iv d1]) q21d × 4 vs (Epirubicin[120mg/m² iv d1] + Cyclophosphamide [600mg/m² iv d1]) q21d × 4	750	Yes	✓ 2012[28]
30403 2001Z	Pegase 07, France	Epirubicin [150 mg/m2 d1] + (Cyclophosphamide [4000 mg/m2 d1] + Mesna + filgrastim [10mcg/kg d5 until last cytapheresis (c1) then 5mcg/kg d3 until neutrophils >500/mm^3 (c2-4)]) q3wk × 4 preoperative then Mastectomy + Axillary Lymph Node Dissection then Radiotherapy + (Docetaxel [75mg/m2 d1] + 5-Fluoro-Uracil [750 mg/m2/day iv d1-d5]) q3wk × 4 +/- Tamoxifen [20 mg/d] × 5yr postoperative vs (Epirubicin [150 mg/m2 d1] + Cyclophosphamide [4000 mg/m2 d1] + Mesna + filgrastim [10mcg/kg d5 until last cytapheresis (c1) then 5mcg/kg d3 until neutrophils >500/mm^3 (c2-4)]) q3wk × 4 preoperative then Mastectomy + Axillary Lymph Node Dissection then Radiotherapy +/- Tamoxifen [20 mg/d] × 5yr postoperative	174	No	✓ 2015[29]
44802 2004!	Craiova Dolj Taxane trial, Romania	(Doxorubicin [60 mg/m2] + Cyclophosphamide [600 mg/m2] + Paclitaxel [200 mg/m2]) q21d × 2-4 cycles preoperative vs (Doxorubicin [60 mg/m2] + Cyclophosphamide [600 mg/m2]) q21d × 2-4 cycles preoperative	124	No	✓ Abstract 2009[30]
**(b) Taxane plus anthracycline based regimen (taxane course given alone) vs MORE (but < doubled) non-taxane chemotherapy**					
38401-02 1995T	HORG Docetaxel trial, Greece	Docetaxel [100mg/m² iv] q3wk × 4 then (Epirubicin[75mg/m² iv] + Cyclophosphamide [700mg/m² iv]) q3wk × 4 vs (5-Fluoro-Uracil [700mg/m² iv] + Epirubicin[75mg/m² iv] + Cyclophosphamide [700mg/m² iv]) q3wk × 6<br>Or<br>Docetaxel [100mg/m² iv] q3wk × 4 then (Epirubicin[75mg/m² iv] + Cyclophosphamide [700mg/m² iv]) q3wk × 4 + Tamoxifen [20mg/d po] × 5yr vs (5-Fluoro-Uracil [700mg/m² iv] + Epirubicin[75mg/m² iv] + Cyclophosphamide [700mg/m² iv]) q3wk × 6 + Tamoxifen [20mg/d po] × 5yr	788	Yes	✓ 2010[31]
24604 1997R	HE1097, Greece	(Epirubicin[110mg/m²] + filgrastim [5µg/kg d3-10]) q14d × 3 then (Paclitaxel [250mg/m² over 3h] + filgrastim [5µg/kg d3-10]) q14d × 3 then (Cyclophosphamide [840mg/m²] + Methotrexate [50mg/m²] + 5-Fluoro-Uracil [840mg/m²] + filgrastim [5µg/kg d3-10]) q14d × 3 vs (Epirubicin[110mg/m²] + filgrastim [5µg/kg d3-10]) q14d × 4 then (Cyclophosphamide [840mg/m²] + Methotrexate [50mg/m²] + 5-Fluoro-Uracil [840mg/m²] + filgrastim [5µg/kg d3-10]) q14d × 4	604	Yes	✓ 2005[32]
28507 1997V	IBIS 02, Italy	Paclitaxel [175mg/m² iv d1] q3wk × 4 then (Epirubicin[75mg/m² iv d1] + Vinorelbine [25mg/m² iv d1,8]) q3wk × 4 vs Epirubicin[100mg/m² iv d1] q3wk × 4 then (Cyclophosphamide [600mg/m² iv d1,8] + Methotrexate [40mg/m² iv d1,8] + 5-Fluoro-Uracil [600mg/m² d1,8]) q4wk × 4	244	Yes	✓ 2010[33]
35403-04 1998D	BIG 02-98, Belgium	Doxorubicin [75mg/m² iv] q3wk × 3 then Docetaxel [100mg/m² iv] q3wk × 3 then (Cyclophosphamide [100mg/m² po d1-14] + Methotrexate [40mg/m² d1,8] + 5-Fluoro-Uracil [600mg/m² iv d1,8]) q28d × 3 vs Doxorubicin [75mg/m² iv]	1927	Yes	✓ 2013[34]

		q3wk × 4 then (Cyclophosphamide [100mg/m² po d1-14] + Methotrexate [40mg/m² d1,8] + 5-Fluoro-Uracil [600mg/m² iv d1,8]) q28d × 3 or (Doxorubicin [60mg/m² iv] + Cyclophosphamide [600mg/m² iv]) q3wk × 4 then (Cyclophosphamide [100mg/m² po d1-14] + Methotrexate [40mg/m² d1,8] + 5-Fluoro-Uracil [600mg/m² iv d1,8]) q28d × 3			
29607 1999K	GEICAM 9906, Spain	(5-Fluoro-Uracil [600mg/m² d1] + Epirubicin[90mg/m² d1] + Cyclophosphamide [600mg/m² d1]) q3wk × 4 then Paclitaxel [100mg/m²] qwk × 8 vs (5-Fluoro-Uracil [600mg/m² d1] + Epirubicin[90mg/m² d1] + Cyclophosphamide [600mg/m² d1]) q3wk × 6	1246	Yes	✓ 2008[35]
28902-03 2000E	FinHer/FBCG 00-01, Finland	Docetaxel [80/100mg/m² d1] q21d × 3 then (5-Fluorouracil [600mg/m² d1] + Epirubicin[60mg/m² d1] + Cyclophosphamide [600mg/m² d1]) q21d × 3 vs Vinorelbine [25mg/m² d1, 8, 15] q21d × 3 then (5-Fluorouracil [600mg/m² d1] + Epirubicin[60mg/m² d1] + Cyclophosphamide [600mg/m² d1]) q21d × 3<br>Or<br>Docetaxel [80/100mg/m² d1] q21d × 3 then (5-Fluorouracil [600mg/m² d1] + Epirubicin[60mg/m² d1] + Cyclophosphamide [600mg/m² d1]) q21d × 3 vs Vinorelbine [25mg/m² d1,8,15] q21d × 3 then (5-Fluorouracil [600mg/m² d1] + Epirubicin[60mg/m² d1] + Cyclophosphamide [600mg/m² d1]) q21d × 3	1009	Yes	✓ 2009[36]
26109/13 2000F	MA.21, Canada	Radiotherapy then (Epirubicin[120mg/m² iv d1] + Cyclophosphamide [830mg/m² iv d1] + filgrastim [5µg/kg/d sc d2-13]) q14d × 6 + Erythropoietin [150µg/kg sc q3d/wk] then Paclitaxel [175mg/m² iv q3wk] × 4 (after 3wk delay) vs Radiotherapy then (5-Fluoro-Uracil [500mg/m² iv d1,8] + Epirubicin[60mg/m² iv d1,8] + Cyclophosphamide [75mg/m² po d1-14]) q28d × 6 + (Co-trimoxazole [480mg po bd] / Ciprofloxacin [500mg po bd])	1402	Yes	✓ 2010[37]
30903 2000S	WSG/AGO AM-02, Germany	(Epirubicin[90mg/m²] + Cyclophosphamide [600mg/m²]) q3wk × 4 then Docetaxel [100mg/m²] q3wk × 4 vs (Cyclophosphamide [500mg/m²] + Epirubicin[100mg/m²] + 5-Fluoro-Uracil [500mg/m²]) q3wk × 6	1606	Yes	✓ 2014[38]
36601 2000U	AERO B-2000, France	(5-Fluoro-Uracil [500mg/m²] + Epirubicin[100mg/m²] + Cyclophosphamide [500mg/m²]) q3wk × 4 then Paclitaxel [175mg/m²] q3wk × 4 vs (5-Fluoro-Uracil [500mg/m²] + Epirubicin[100mg/m²] + Cyclophosphamide [500mg/m²]) q3wk × 6	837	Yes	✓ 2014[39]
1140 2003$	GEICAM 2004-04	Paclitaxel [80mg/m² iv] qwk × 12 then ((5-Fluoro-Uracil [500mg/m² iv d1] + Doxorubicin [50mg/m² iv d1] + Cyclophosphamide [500mg/m² iv d1]) q3wk × 4 / (5-Fluoro-Uracil [500mg/m² iv d1] + Epirubicin[100mg/m² iv d1] + Cyclophosphamide [500mg/m² iv d1]) q3wk × 4) preoperative vs ((5-Fluoro-Uracil [500mg/m² iv d1] + Doxorubicin [50mg/m² iv d1] + Cyclophosphamide [500mg/m² iv d1]) q3wk × 6 / (5-Fluoro-Uracil [500mg/m² iv d1] + Epirubicin[100mg/m² iv d1] + Cyclophosphamide [500mg/m² iv d1]) q3wk × 6) preoperative	273	Yes	✓ 2010[40]
37804 2003R	GIM 1, Italy	(Epirubicin[90mg/m²] + Cyclophosphamide [600mg/m²]) q21d × 4 then Docetaxel [100mg/m²] q21d × 4 vs (5-Fluoro-Uracil [600mg/m²] + Epirubicin[75mg/m²] + Cyclophosphamide [600mg/m²]) q21d × 6	1636	No	No
29610-12 2003U	GEICAM 2003-02, Spain	(5-Fluoro-Uracil [500mg/m²] + Doxorubicin [50mg/m²] + Cyclophosphamide [500mg/m²]) q3wk × 4 then Paclitaxel [100mg/m²] qwk × 8 vs (5-Fluoro-Uracil	1925	Yes	✓ 2013[41]

		[500mg/m²] + Doxorubicin [50mg/m²] + Cyclophosphamide [500mg/m²]) q3wk × 6			
**(c) Taxane plus anthracycline (taxane given concurrently) vs MORE (but < doubled) non-taxane chemotherapy**					
33801 1995E	LMU Munich, Germany	(Docetaxel [75mg/m²] + Epirubicin[60mg/m²]) q21d × 6 vs (Epirubicin[90mg/m²] + Cyclophosphamide [600mg/m²]) q21d × 4 then (Cyclophosphamide [600mg/m²] + Methotrexate [40mg/m²] + 5-Fluoro-Uracil [600mg/m²]) q21d × 3	36	No	✓ 2000[42]
31101-02 1996#	Helena-Venizelou Chemotherapy Trial, Greece	(Epirubicin[75mg/m² iv d1] + Paclitaxel [200mg/m² over 3h d1]) q21d × 3 preoperative then (Epirubicin[75mg/m² iv d1] + Paclitaxel [200mg/m² over 3h d1]) q21d × 3 postoperative + Radiotherapy [to chest wall] then Tamoxifen × 5yr vs (5-Fluoro-Uracil [600mg/m² d1] + Epirubicin[75mg/m² d1] + Cyclophosphamide [600mg/m² d1]) q21d × 3 preoperative then (5-Fluoro-Uracil [600mg/m² d1] + Epirubicin[75mg/m² d1] + Cyclophosphamide [600mg/m² d1]) q21d × 3 postoperative + Radiotherapy [to chest wall] then Tamoxifen × 5yr	70	No	✓ Abstract 1998[43]
30801 1996%	Multicentre Germany Chemotherapy Trial	(Epirubicin[90mg/m² iv d1] + Paclitaxel [175mg/m² iv d1]) q14d × 4 + filgrastim [5mcg/kg sc d5-13] then (Cyclophosphamide [600mg/m² iv d1] + Methotrexate [40mg/m² iv d1] + 5-Fluoro-Uracil [600mg/m² iv d1]) q14d × 3 vs (Epirubicin[90mg/m² iv d1] + Cyclophosphamide [600mg/m² iv d1]) q21d × 4 then (Cyclophosphamide [600mg/m² iv d1] + Methotrexate [40mg/m² iv d1] + 5-Fluoro-Uracil [600mg/m² iv d1]) q21d × 3	228	Yes	✓ 2006[44]
3521 1996Q	European Cooperative Trial in Operable Breast Cancer (ECTO)	(Epirubicin[60mg/m² iv] then Paclitaxel [200mg/m² iv over 3h]) q3wk × 4 then (Cyclophosphamide [600mg/m² iv d1,8] + Methotrexate[40mg/m² iv d1,8] + 5-Fluoro-Uracil [600mg/m² iv d1,8]) q4wk × 4 then Tamoxifen [20mg/d] × 5yr or (Epirubicin[60mg/m² iv] then Paclitaxel [200mg/m² iv over 3h]) q3wk × 4 preoperative then (Cyclophosphamide [600mg/m² iv d1,8] + Methotrexate [40mg/m² iv d1,8] + 5-Fluoro-Uracil [600mg/m² iv d1,8]) q4wk × 4 preoperative then Tamoxifen [20mg/d] × 5yr vs Epirubicin[75mg/m² iv] q3wk × 4 then (Cyclophosphamide [600mg/m² iv d1,8] + Methotrexate [40mg/m² iv d1,8] + 5-Fluoro-Uracil [600mg/m² iv d1,8]) q4wk × 4 then Tamoxifen [20mg/d] × 5yr	1355	Yes	✓ 2009[45]
31401 1996X	GONO-MIG 5, Italy	(Epirubicin[90mg/m² iv d1] + Paclitaxel [175mg/m² iv over 3h d1]) q3wk × 4 vs (5-Fluoro-Uracil [600mg/m² iv d1] + Epirubicin[60mg/m² iv d1] + Cyclophosphamide [600mg/m² iv d1]) q3wk × 6	1055	Yes	✓ 2016[46]
33102 1997H1	Multicentre France Neoadjuvant Chemotherapy Trial	(Doxorubicin [60mg/m² iv] + Paclitaxel [200mg/m² iv]) q3wk × 4 preoperative vs (Doxorubicin [60mg/m² iv] + Cyclophosphamide [600mg/m² iv]) q3wk × 4 preoperative	200	Synthetic	✓ 2004[47]
33501 1997L	BCIRG 001 / GEICAM 9703	(Doxorubicin [50mg/m² iv d1] + Cyclophosphamide [500mg/m² iv d1] + Docetaxel [75mg/m² iv d1]) q21d × 6 vs (Doxorubicin [50mg/m² iv d1] + 5-Fluoro-Uracil [500mg/m² iv d1] + Cyclophosphamide [500mg/m² iv d1]) q21d × 6	1491	Yes	✓ 2013[48]
35403-04 1998D	BIG 02-98, Belgium	(Doxorubicin [50mg/m² iv] + Docetaxel [75mg/m² iv]) q3wk × 4 then (Cyclophosphamide [100mg/m² po d1-14] + Methotrexate [40mg/m² d1,8] + 5-Fluoro-Uracil [600mg/m² iv d1,8]) q28d × 3 vs Doxorubicin [75mg/m² iv] q3wk	1928	Yes	✓ 2013[34]

		× 4 then (Cyclophosphamide [100mg/m² po d1-14] + Methotrexate [40mg/m² d1,8] + 5-Fluoro-Uracil [600mg/m² iv d1,8]) q28d × 3 or (Doxorubicin [60mg/m² iv] + Cyclophosphamide [600mg/m² iv]) q3wk × 4 then (Cyclophosphamide [100mg/m² po d1-14] + Methotrexate [40mg/m² d1,8] + 5-Fluoro-Uracil [600mg/m² iv d1,8]) q28d × 3			
7520<br>1998T	ECOG EST2197, USA	(Doxorubicin [60mg/m²] + Docetaxel [60mg/m²]) q3wk × 4 vs (Doxorubicin [60mg/m²] + Cyclophosphamide [600mg/m²]) q3wk × 4	2952	Yes	✓ 2008[49]
29603-06<br>1999%	GEICAM 9805 / TARGET 0, Spain	(Docetaxel [75mg/m² d1] + Doxorubicin [50mg/m² d1] + Cyclophosphamide [500mg/m² d1]) q3wk × 6 vs (5-Fluoro-Uracil [500mg/m² d1] + Doxorubicin [50mg/m² d1] + Cyclophosphamide [500mg/m² d1]) q3wk × 6	1060	Yes	✓ 2010[50]
27402<br>1999A	Anglo-Celtic II (Primary Medical Therapy Trial IIA), UK	(Doxorubicin [50mg/m²] + Docetaxel [75mg/m²]) q3wk × 6 primary preoperative vs (Doxorubicin [60mg/m²] + Cyclophosphamide [600mg/m²]) q3wk × 6 primary preoperative	363	Yes	✓ 2010[51]
20603<br>1999N	RAPP-01, France	(Doxorubicin [50mg/m²] + Docetaxel [75mg/m²]) q3wk × 4 postoperative vs (Doxorubicin [60mg/m²] + Cyclophosphamide [600mg/m²]) q3wk × 4 postoperative	627	Yes	✓ 2005[52]
33202/04<br>2001E1+3	PACS 04, France	(Epirubicin[75mg/m² iv d1] + Docetaxel [75mg/m² iv d1]) q3wk × 6 + Radiotherapy vs (5-Fluoro-Uracil [500mg/m² iv d1] + Epirubicin[100mg/m² iv d1] + Cyclophosphamide [500mg/m² iv d1]) q3wk × 6 + Radiotherapy	3009	Yes	✓ 2009[53]
8727<br>2002L	St Petersburg Neoadjuvant trial, Russia	(Paclitaxel [200 mg/m2] + Doxorubicin [60 mg/m2]) q3wk × 4 preoperative vs (5-Fluorouracil [600 mg/m2] + Doxorubicin [60 mg/m2] + Cyclophosphamide [600 mg/m2]) q3wk × 4 preoperative	103	No	✓ Abstract 2002[54]
38913-14<br>2004N	MATADOR / BOOG 2005-02 / CKTO 2004-04	(Docetaxel [75mg/m iv d1] + Doxorubicin [50mg/m² iv d1] + Cyclophosphamide [500mg/m² iv d1] + Pegfilgrastim [6mg sc 1d after chemo]) q3wk × 6 vs (Doxorubicin [60mg/m² iv d1] + Cyclophosphamide [600mg/m² iv d1] + Pegfilgrastim [6mg sc 1d after chemo]) q2wk × 6	664	Yes	✓ 2018[55]
40302<br>2007”	Kolkata Chemotherapy trial, India	Doxorubicin [60 mg/m2] + Cyclophosphamide [600 mg/m2] q3wk × 6 then Paclitaxel [175 mg/m2] q3wk × 3 vs Doxorubicin [60 mg/m2] + Cyclophosphamide [600 mg/m2] q3wk × 6	50	No	✓2012[56]
**(d) Taxane plus anthracycline vs DOUBLED non-taxane cytotoxic chemotherapy**					
1122-24, 27-29<br>1994B	Protocol 94-002 MDA	Paclitaxel [250mg/m² 24h infusion] q3wk × 4 then (5-Fluoro-Uracil [500mg/m² iv d1,4] + Doxorubicin [50mg/m² 72h infusion] + Cyclophosphamide [500mg/m² iv d1]) q3wk × 4 then Radiotherapy vs (5-Fluoro-Uracil [500mg/m² iv d1,4] + Doxorubicin [50mg/m² 72h infusion] + Cyclophosphamide [500mg/m² iv d1]) q3wk × 8 then Radiotherapy	474	Yes	✓ 2011[57]
34001<br>1996F	Aberdeen Trial, Scotland	(Cyclophosphamide [1000mg/m²] + Doxorubicin [50mg/m²] + Vincristine [1·5mg/m²] + Prednisolone [40mg]) q21d × 4 preoperative then (Prednisolone [100mg po] + Docetaxel [100mg/m²]) q21d × 4 postoperative vs (Cyclophosphamide [1000mg/m²] + Doxorubicin [50mg/m²] + Vincristine [1·5mg/m²] + Prednisolone [40mg]) q21d × 4 preoperative then (Cyclophosphamide [1000mg/m²] + Doxorubicin [50mg/m²] + Vincristine [1·5mg/m²] + Prednisolone [100mg po]) q21d × 4 postoperative	104	Synthetic	✓ Abstract 2003[58]

20008-09 1997A	DEVA, UK	(Epirubicin[50mg/m² d1,8] q4wk × 3 then Docetaxel [100mg/m² d1] q3wk × 3) + Tamoxifen [20 mg/d] × 5yr concurrent vs Epirubicin[50mg/m² d1,8] q4wk × 6 + Tamoxifen [20 mg/d] × 5yr concurrent	803	Yes	✓ 2011[59]
33201 1997J	PACS 01, France	(5-Fluoro-Uracil [500mg/m² d1] + Epirubicin[100mg/m² d1] + Cyclophosphamide [500mg/m² d1]) q21d × 3 then Docetaxel [100mg/m² d1] q21d × 3 vs (5-Fluoro-Uracil [500mg/m² d1] + Epirubicin[100mg/m² d1] + Cyclophosphamide [500mg/m² d1]) q21d × 6	1999	Yes	✓ 2012[60]
26109/13 2000F	MA.21, Canada	Radiotherapy then (Doxorubicin [60mg/m² iv d1] + Cyclophosphamide [600mg/m² iv d1]) q21d × 4 then Paclitaxel [175mg/m² iv q3wk] × 4 (after 3wk delay) vs Radiotherapy then (5-Fluoro-Uracil [500mg/m² iv d1,8] + Epirubicin[60mg/m² iv d1,8] + Cyclophosphamide [75mg/m² po d1-14]) q28d × 6 + (Co-trimoxazole [480mg po bd] / Ciprofloxacin [500mg po bd])	1403	Yes	✓ 2010[37]
24016/18 2001F	TACT, UK	(5-Fluoro-Uracil [600mg/m² iv] + Epirubicin[60mg/m² iv] + Cyclophosphamide [600mg/m² iv]) q3wk × 4 then Docetaxel [100mg/m² iv d1] q3wk × 4 vs (5-Fluoro-Uracil [600mg/m² iv] + Epirubicin[60mg/m² iv] + Cyclophosphamide [600mg/m² iv]) q3wk × 8	2523	Yes	✓ 2009[61]
24017/19 2001G1+2	TACT, UK	(5-Fluoro-Uracil [600mg/m² iv] + Epirubicin[60mg/m² iv] + Cyclophosphamide [600mg/m² iv]) q3wk × 4 then Docetaxel [100mg/m² iv d1] q3wk × 4 vs Epirubicin[100mg/m² iv] q3wk × 4 then (Cyclophosphamide [100mg/m² po d1-14 / 600mg/m² iv d1,8] + Methotrexate [40mg/m² iv d1,8] + 5-Fluoro-Uracil [600mg/m² iv d1,8]) q4wk × 4	1639	Yes	✓ 2009[61]
6320-21 2001J	E.O.R.T.C. Trial 10994	Docetaxel [100mg/m² iv] q3wk × 3 then (Epirubicin[90mg/m² iv] + Docetaxel [75mg/m² iv]) q3wk × 3 preoperative vs ((5-Fluoro-Uracil [500mg/m² iv] + Epirubicin[100mg/m² iv] + Cyclophosphamide [500mg/m² iv]) q3wk × 6) / ((5-Fluoro-Uracil [iv d1,8] + Epirubicin[iv d1,8] + Cyclophosphamide [po d1-14]) q4wk × 6) / ((5-Fluoro-Uracil [iv d1] + Epirubicin[iv d1] + Cyclophosphamide [iv d1] + Filgrastim [sc d2-15]) q3wk × 6) preoperative	1856	Yes	✓ 2014[62]
33804 2001T	ADEBAR Study, Germany	Radiotherapy + (Epirubicin[90mg/m²] + Cyclophosphamide [600mg/m²]) q21d ×4 then Docetaxel [100mg/m²] q21d × 4 vs Radiotherapy + (Epirubicin[60mg/m² d1,8] + 5-Fluoro-Uracil [500mg/m² d1,8] Cyclophosphamide [75mg/m² d1-14]) q4wk × 6	1363	Yes	✓ 2016[63]
35009-10 2002D	GBG 42, Germany	(5-Fluoro-Uracil [500mg/m²] + Epirubicin[100mg/m²] + Cyclophosphamide [500mg/m²]) q3wk × 3 then Docetaxel [100mg/m²] q3wk × 3 vs (5-Fluoro-Uracil [500mg/m²] + Epirubicin[100mg/m²] + Cyclophosphamide [500mg/m²]) q3wk × 6	2660	Yes	✓ Abstract[64] 2012
28403-08 2002G	Bari Inflammatory Breast Cancer with Surgery, Italy	(Epirubicin[120mg/m² d1] + Vinorelbine [25mg/m² d1,8]) × 4 preoperative then Surgery/Radiotherapy then Docetaxel [100mg/m²] q3wk × 4 vs (Epirubicin[120mg/m² d1] + Vinorelbine [25mg/m² d1,8]) × 4 preoperative then Surgery/Radiotherapy then (Epirubicin[120mg/m² d1] + Vinorelbine [25mg/m² d1,8]) q3wk × 4	56	No	✓ Abstract 2008[65]

Cohort 3: Taxane vs Anthracycline

Trial & year code	Trial name	Comparisons	N	Received	Published
**(a) Docetaxel vs anthracycline**					
34201&03 1997N1&2	U.S. Oncology Trial 97-35, USA	(Docetaxel [75mg/m²] + Cyclophosphamide [600mg/m²]) q3wk × 4 vs (Doxorubicin [60mg/m²] + Cyclophosphamide [600mg/m²]) q3wk × 4	1016	Yes	✓ 2005[66]
6328 2006Z2	MINDACT/ EORTC 10041 / BIG 3-04, Belgium	Docetaxel [75 mg/m2 iv d1] + Capecitabine [825 mg/m2 po bd d1-14] q3wk × 6 vs (Fluorouracil [500 mg/m2 iv] + Epirubicin [100 mg/m2 iv] + Cyclophosphamide [500 mg/m2 iv]) d1 q3wk × 6 (or various other anthracycline based regimens)	909	Yes	✓ 2017[67]
51901 2008)	Singapore-NCC0705	(Cyclophosphamide [iv d1] + Docetaxel [iv d1]) q3wk × 4 preoperative vs (Cyclophosphamide [iv d1] + Doxorubicin [iv d1]) q3wk × 4 preoperative	20	No	No
48301 2008Y	Duke Taxane trial/NCT00636441, USA	(Docetaxel [75 mg/m2] + Cyclophosphamide [600 mg/m2]) q3wk × 4 preoperative vs (Doxorubicin [60 mg/m2] + Cyclophosphamide [600 mg/m2]) q3wk × 4 preoperative	56	No	✓ 2010[68]
**(b) Paclitaxel vs anthracycline**					
44701&02 1997[1&2	Epi-Tax trial, Norway	Paclitaxel [200 mg/m2] q3wk × 4 preoperative vs Epirubicin [90 mg/m2] q3wk × 4 preoperative	223	Yes	✓ 2011[69]
8322 2002B1	CALGB 40101, USA	(Doxorubicin [60mg/m²] + Cyclophosphamide [600mg/m²]) q3wk × 4 or × 6 vs Paclitaxel [80mg/m²] qwk × 12 or × 18	570	Yes	✓ 2014[70]
8323 2002B2	CALGB 40101, USA	(Doxorubicin [60mg/m²] + Cyclophosphamide [600mg/m²]) q2wk × 4 or × 6 vs Paclitaxel [175mg/m²] q2wk × 4 or × 6	2622	Yes	✓ 2014[70]
8324 2002B3	CALGB 40101, USA	(Doxorubicin [60mg/m²] + Cyclophosphamide [600mg/m²]) q2wk × 4 vs Paclitaxel [175mg/m²] q2wk × 4	679	Yes	✓ 2014[70]

Cohort 4: Head-to-Head Taxanes

Trial code	Trial name	Comparisons	N	Received	Published
**(a) Docetaxel 2-weekly or 3-weekly vs paclitaxel 2-weekly or 3-weekly**					
7521-22 1999V	E1199/Intergroup Trial, ECOG, USA	(Doxorubicin [60 mg/m2 iv] + Cyclophosphamide [600 mg/m2 iv]) q3wk × 4 then Docetaxel [100 mg/m2 iv over 1h] q3wk × 4 vs (Doxorubicin [60 mg/m2 iv] + Cyclophosphamide [600 mg/m2 iv]) q3wk × 4 then Paclitaxel [175 mg/m2 iv over 3h] q3wk × 4	2534	Yes	✓ 2015[71]

35901 2000W	N-SAS BC 02, Japan	((Doxorubicin [60mg/m²] or Epirubicin [75mg/m2]) + Cyclophosphamide [600mg/m²]) q3wk × 4 then Docetaxel [75mg/m²] q3wk × 4 then Tam/AIs × 5yr if ER+/PR+ vs ((Doxorubicin [60mg/m²] or Epirubicin [75mg/m2]) + Cyclophosphamide [600mg/m²]) q3wk × 4 then Paclitaxel [175mg/m²] q3wk × 4 then Tam/AIs × 5yr if ER+/PR+<br>Or<br>Docetaxel [75mg/m²] q3wk × 8 then Tam/AIs × 5yr if ER+/PR+ vs Paclitaxel [175mg/m²] q3wk × 8 then Tam/AIs × 5yr if ER+/PR+	1060	Yes	✓ 2017[11]
38404 2004R	CT/04.22, HORG, Greece	(5-fluorouracil [700mg/m2 iv] d1 + epirubicin [75mg/m2 iv d1,15] + cyclophosphamide [700mg/m2 iv d1]) q4w × 4 then (Docetaxel [75mg/m2 iv] q2w × 4 vs Paclitaxel [175mg/m2 iv] q2w × 4)	481	Yes	✓ 2014[73]
42201	Mansoura Taxane trial, Egypt	(Doxorubicin [60 mg/m2] + Cyclophosphamide [600 mg/m2]) q3wk × 4 then Docetaxel [100 mg/m2] q3wk × 4 vs (Doxorubicin [60 mg/m2] + Cyclophosphamide [600 mg/m2]) q3wk × 4 then Paclitaxel [175 mg/m2] q3wk × 4	160	No	✓ Abstract 2011[73]
**(b) Docetaxel weekly vs paclitaxel weekly**					
7521-22 1999V	E1199/Intergroup Trial, ECOG, USA	(Doxorubicin [60 mg/m2 iv] + Cyclophosphamide [600 mg/m2 iv]) q3wk × 4 then Docetaxel [35 mg/m2 iv over 1h] qwk × 12 vs (Doxorubicin [60 mg/m2 iv] + Cyclophosphamide [600 mg/m2 iv]) q3wk × 4 then Paclitaxel [80 mg/m2 iv over 1h] qwk × 12	2518	Yes	✓ 2015[71]
24610 2005C	HE 10/05, HECOG, Greece	Epirubicin [110mg/m2] q2wk × 3 then (Cyclophosphamide [840mg/m2] + Methotrexate [57mg/m2] + 5-Fluorouracil [840mg/m2]) q2wk × 3 then Docetaxel [35mg/m2] qwk × 9 vs Epirubicin [110mg/m2] q2wk × 3 then (Cyclophosphamide [840mg/m2] + Methotrexate [57mg/m2] + 5-Fluorouracil [840mg/m2]) q2wk × 3 then Paclitaxel [80mg/m2] qwk × 9	664	Yes	✓ 2014[75]
38302 2007'	KBCOG-06, Japan	Docetaxel [75mg/m2] q3wk × 4 then (5-Fluoro-Uracil + Epirubicin + Cyclophosphamide) preoperative vs Paclitaxel [80mg/m2 d1,8,15] q4wk × 4 then (5-Fluoro-Uracil + Epirubicin + Cyclophosphamide) preoperative	50	No	No

Cohort 5: Dose fractionation

Trial & year code	Trial name	Comparisons	N	Received	Published
**(a) Chemotherapy in smaller fractions: Paclitaxel**					
1125-26, 1134-37 1998K	MDA 98-240, USA	Paclitaxel [80mg/m²] qwk × 12 + (5-Fluoro-Uracil [500mg/m² d1,4] + Cyclophosphamide [500mg/m² d1] + Doxorubicin [50mg/m² over 72h]) q3wk × 4 preoperative then Local Therapy vs Paclitaxel [225mg/m² over 24h] q3wk × 4 + (5-Fluoro-Uracil [500mg/m² d1,4] + Cyclophosphamide [500mg/m² d1] + Doxorubicin [50mg/m² over 72h]) q3wk × 4 preoperative then Local Therapy<br>Or	254	Yes	✓ 2005[76]

		Paclitaxel [150/175mg/m²] qwk × 12 + (5-Fluoro-Uracil [500mg/m² d1,4] + Cyclophosphamide [500mg/m² d1] + Doxorubicin [50mg/m² over 72h]) q3wk × 4 preoperative then Local Therapy vs Paclitaxel [225mg/m² over 24h] q3wk × 4 + (5-Fluoro-Uracil [500mg/m² d1,4] + Cyclophosphamide [500mg/m² d1] + Doxorubicin [50mg/m² over 72h]) q3wk × 4 preoperative then Local Therapy			
7521-22 1999V	E1199/Intergroup Trial, ECOG, USA	(Doxorubicin [60 mg/m2 iv] + Cyclophosphamide [600 mg/m2 iv]) q3wk × 4 then Paclitaxel [80 mg/m2 iv over 1h] qwk × 12 vs (Doxorubicin [60 mg/m2 iv] + Cyclophosphamide [600 mg/m2 iv]) q3wk × 4 then Paclitaxel [175 mg/m2 iv over 3h] q3wk × 4	2530	Yes	✓ 2015[71]
1215/19 2003*	SWOG S0221, USA	(Doxorubicin [24mg/m²/wk] + Cyclophosphamide [60mg/m²/d po] + filgrastim [5mcg/kg/d sc d2-7]) qwk × 15wk then Paclitaxel [80mg/m²] qwk × 12 or (Doxorubicin [60mg/m²] + Cyclophosphamide [600mg/m²] + Pegfilgrastim [6mg sc d2]) q2wk × 6 then Paclitaxel [80mg/m²] qwk × 12 vs (Doxorubicin [24mg/m²/wk iv] + Cyclophosphamide [60mg/m²/d po] + filgrastim [5mcg/kg/d sc d2-7]) qwk × 15wk then (Paclitaxel [175mg/m²] + Pegfilgrastim [6mg sc d2]) q2wk × 6 or (Doxorubicin [60mg/m² d1] + Cyclophosphamide [600mg/m² iv d1] + Pegfilgrastim [6mg sc d2]) q2wk × 6 then (Paclitaxel [175mg/m²] + Pegfilgrastim [6mg sc d2]) q2wk × 6	3294	Yes	✓ 2015[77]
24610 2005C	HE 10/05, HECOG, Greece	Epirubicin [110mg/m2] q2wk × 3 then (Cyclophosphamide [840mg/m2] + Methotrexate [57mg/m2] + 5-Fluorouracil [840mg/m2]) q2wk × 3 then Paclitaxel [80mg/m2] qwk × 9 vs Epirubicin [110mg/m2] q2wk × 3 then Paclitaxel [200mg/m2] q2wk × 3 then (Cyclophosphamide [840mg/m2] + Methotrexate [57mg/m2] + 5-Fluorouracil [840mg/m2]) q2wk × 3	667	Yes	✓ 2012[75]
51801 2005R	PKU Paclitaxel trial, Beijing China	(Carboplatin [AUC 6 d1] + Paclitaxel [60 mg/m2 d1,8,15]) q3wk × 4 preoperative vs (Carboplatin [AUC 6 d1] + Paclitaxel [175 mg/m2 d1]) q3wk × 4 preoperative	220	No	Abstract 2010[78]
**(b) Chemotherapy in smaller fractions: Docetaxel**					
7521-22 1999V	E1199/Intergroup Trial, ECOG, USA	(Doxorubicin [60 mg/m2 iv] + Cyclophosphamide [600 mg/m2 iv]) q3wk × 4 then Docetaxel [35 mg/m2 iv over 1h] qwk × 12 vs (Doxorubicin [60 mg/m2 iv] + Cyclophosphamide [600 mg/m2 iv]) q3wk × 4 then Docetaxel [100 mg/m2 iv over 1h] q3wk × 4	2886	Yes	✓ 2015[71]
45201-02 2000^	Hull Neoadjuvant trial, UK	(Doxorubicin [60mg/m2 iv] + Cyclophosphamide [600mg/m² iv] q3wk) × 4 then Docetaxel [33mg/m² iv] qwk × 12 (with a 2 week break between cycles 6 and 7) preoperative vs (Doxorubicin [60mg/m2 iv] + Cyclophosphamide [600mg/m² iv] q3wk) × 4 then Docetaxel [100mg/m2 iv] q3wk × 4 preoperative	82	Yes	✓ 2011[79]
38803 2004Z	BREAST-10, Italy	Epirubicin [30mg/m2 d1,8,15] + Docetaxel [35mg/m2 d1,8,15] q4wk × 3 preoperative vs Epirubicin [75mg/m2 d1] + Docetaxel [80mg/m2 d1] q3wk × 4 preoperative	44	Yes	✓ 2011[80]
46401 2005@	UZLeuven Chemotherapy trial, Belgium	((5-fluorouracil [375 mg/m2] + Epirubicin [75 mg/m2] + Cyclophosphamide [375 mg/m2]) q10-11d × 4 then Docetaxel [75 mg/m2] q2wk × 4) + pegfilgrastim [6mg sc] d2 of each cycle vs (5-fluorouracil [500 mg/m2] +	85	Yes	✓ 2009[81]

		Epirubicin [100 mg/m2] + Cyclophosphamide [500 mg/m2]) q3wk × 3 then Docetaxel [100 mg/m2] q3wk × 3<br>Or<br>(Docetaxel [75 mg/m2] q2wk × 4 then (5-fluorouracil [375 mg/m2] + Epirubicin [75 mg/m2] + Cyclophosphamide [375 mg/m2]) q10-11d × 4) + pegfilgrastim [6mg sc] d2 of each cycle vs Docetaxel [100 mg/m2] q3wk × 3 then (5-fluorouracil [500 mg/m2] + Epirubicin [100 mg/m2] + Cyclophosphamide [500 mg/m2]) q3wk × 3			
45504<br>2007e	Emory Induction trial / NCT00209092, USA	(Docetaxel [50 mg/m2 iv d1] + Capecitabine [2000 mg/m2 po d1-7]) q2wk × 8 vs Docetaxel [100 mg/m2 iv d1] q3wk × 4 then Capecitabine [2000 mg/m2 po d1-14] q3wk × 4 preoperative	51	No	✓ 2016[83]

Cohort 6: Sequencing of sequential anthracycline and taxane chemotherapy

Trial & year code	Trial name	Comparisons	N	Received	Published
6607<br>2000N	DFCI 99278, USA	Doxorubicin [60mg/m² iv] q2wk × 4 preoperative then Paclitaxel [80mg/m² iv] qwk × 9 preoperative/postoperative vs Paclitaxel [80mg/m² iv] qwk × 9 preoperative then Doxorubicin [60mg/m² iv] q2wk × 4 preoperative/postoperative	62	No	✓ 2005[82]
40801<br>2002K	Goyang Neoadjuvant Crossover Trial, South Korea	(Doxorubicin [60mg/m² d1] + Cyclophosphamide [600mg/m² d1]) q3wk × 4 preoperative then (Docetaxel [75mg/m² d1] + Capecitabine [1000mg/m² bd d1-14]) q3wk × 4 postoperative vs (Docetaxel [75mg/m² d1] + Capecitabine [1000mg/m² bd d1-14]) q3wk × 4 preoperative then (Doxorubicin [60mg/m² d1] + Cyclophosphamide [600mg/m² d1]) q3wk × 4 postoperative	209	Yes	✓ 2008[84]
45101<br>2002U	Mount Vernon & Baylor College TAC, UK	(Doxorubicin [60 mg/m2] + Cyclophosphamide [600 mg/m2]) × 4 preoperative then Docetaxel [100 mg/m2] × 4 postoperative vs Docetaxel [100 mg/m2] × 4 preoperative then (Doxorubicin [60 mg/m2] + Cyclophosphamide [600 mg/m2]) × 4 postoperative	120	No	✓ Abstract 2007[86]
36602<br>2003L	AERO-B03, France	(Epirubicin[100mg/m²] + Cyclophosphamide [600mg/m²] + Pegfilgrastim [d2]) q2wk × 4 then (Docetaxel [100mg/m²] + Pegfilgrastim [6mg d2]) q2wk × 4 vs (Docetaxel [75mg/m²] + Epirubicin[75mg/m²] + Cyclophosphamide [500mg/m²] + Pegfilgrastim [6mg d2]) q3wk × 6	65	Yes	✓ 2007[85]
38301<br>2003[	KBCOG-02, Japan	(Epirubicin[75mg/m²] + Cyclophosphamide [600mg/m²]) q3wk × 4 preoperative then Paclitaxel [80mg/m²] qwk × 12 postoperative vs Paclitaxel [80mg/m²] qwk × 12 preoperative then (Epirubicin[75mg/m²] + Cyclophosphamide [600mg/m²]) q3wk × 4 postoperative	171	Yes	✓ Abstract 2008[87]
50601<br>2004^	OSU Chemotherapy Sequencing, USA	(Doxorubicin [60mg/m2 iv] + cyclophosphamide [600 mg/m2 iv]) q2wk × 4 then docetaxel [75mg/m2 iv] q2wk × 4 then pegfilgrastim [6mg sc] following each chemotherapy cycle vs Docetaxel [75mg/m2 iv] q2wk × 4 then	56	No	✓ 2008[88]

		(doxorubicin [60mg/m2 iv] + cyclophosphamide [600 mg/m2 iv]) q2wk × 4 then pegfilgrastim [6mg sc] following each chemotherapy cycle			
53101 2004{	SETUP / ACTRN12605000588695, Australia	(5-fluorouracil [500mg/m2 iv] + Epirubicin [100mg/m2 iv] + Cyclophosphamide [500mg/m2 iv]) q3wk × 4 then Docetaxel [100mg/m2 iv] preoperative vs Docetaxel [100mg/m2 iv] then (5-fluorouracil [500mg/m2 iv] + Epirubicin [100mg/m2 iv] + Cyclophosphamide [500mg/m2 iv]) q3wk × 4 preoperative	69	No	✓ 2014[89]
46401 2005@	UZLeuven Chemotherapy trial	(5-fluorouracil [500 mg/m2] + Epirubicin [100 mg/m2] + Cyclophosphamide [500 mg/m2]) q3wk × 3 then Docetaxel [100 mg/m2] q3wk × 3 vs Docetaxel [100 mg/m2] q3wk × 3 then (5-fluorouracil [500 mg/m2] + Epirubicin [100 mg/m2] + Cyclophosphamide [500 mg/m2]) q3wk × 3<br>Or<br>((5-fluorouracil [375 mg/m2] + Epirubicin [75 mg/m2] + Cyclophosphamide [375 mg/m2]) q10-11d × 4 then Docetaxel [75 mg/m2] q2wk × 4) + pegfilgrastim [6mg sc] d2 of each cycle vs (Docetaxel [75 mg/m2] q2wk × 4 then (5-fluorouracil [375 mg/m2] + Epirubicin [75 mg/m2] + Cyclophosphamide [375 mg/m2]) q10-11d × 4) + pegfilgrastim [6mg sc] d2 of each cycle	85	Yes	✓ 2009[81]
1812 2005A	Neo-tAnGo Trial, UK	(Epirubicin[90mg/m²] + Cyclophosphamide [600mg/m² d1]) q3wk × 4 preoperative then Paclitaxel [175mg/m² d1] q2wk × 4 preoperative vs Paclitaxel [175mg/m² d1] q2wk × 4 preoperative then (Epirubicin[90mg/m²] + Cyclophosphamide [600mg/m² d1]) q3wk × 4 preoperative<br>Or<br>(Epirubicin[90mg/m²] + Cyclophosphamide [600mg/m² d1]) q3wk × 4 preoperative then (Paclitaxel [175mg/m² d1] + Gemcitabine [2g/m² d1]) q2wk × 4 preoperative vs (Paclitaxel [175mg/m² d1] + Gemcitabine [2g/m² d1]) q2wk × 4 preoperative then (Epirubicin[90mg/m²] + Cyclophosphamide [600mg/m² d1]) q3wk × 4 preoperative	828	Yes	✓ 2014[90]
24903 2005X	NCT00123929 H San Carlos Madrid, Spain	Doxorubicin [75 mg/m2] q3wk × 4 preoperative then surgery then Docetaxel [100 mg/m2] q3wk × 4 + Radiotherapy + Tam/AIs if ER+/PR+ vs Docetaxel [100 mg/m2] q3wk × 4 preoperative then surgery then Doxorubicin [75mg/m2] q3wk × 4 + Radiotherapy + Tam/AIs if ER+/PR+	204	No	✓ 2011[91]
50701 2006N	Shiga Chemotherapy Sequencing, Japan	(5-Fluorouracil [500 mg/m2] + Epirubicin [110 mg/m2] + Cyclophosphamide [500 mg/m2]) q3wk × 3 then Docetaxel [100mg/m2] q3wk × 3 vs Docetaxel [100mg/m2] q3wk × 3 then (5-Fluorouracil [500 mg/m2] + Epirubicin [110 mg/m2] + Cyclophosphamide [500 mg/m2]) q3wk × 3	42	No	✓ 2013[92]
32502 2007Y	ACOSOG Z1041, USA	5-Fluorouracil [500 mg/m2 iv] + Epirubicin [75 mg/m2 iv] + Cyclophosphamide [500 mg/m2] d1 q3wk × 4 then (Paclitaxel [80 mg/m2] + Trastuzumab [4 mg/kg loading dose and 2 mg/kg weekly dose] d1, d8, d15) q3wk × 4 preoperative then surgery then Trastuzumab [6 mg/kg] d1 q21d for up to 52 wks vs Paclitaxel [80 mg/m2] + Trastuzumab [4 mg/kg loading dose and 2 mg/kg weekly dose] d1, d8, d15 q3wk × 4 then 5-Fluorouracil [500 mg/m2 iv] + Epirubicin [75 mg/m2 iv] + Cyclophosphamide [500	282	Yes	✓ 2019[93]

		mg/m2] d1 q3w × 4 + Trastuzumab [2 mg/kg] d1, d8, d15 q3wk × 4 concurrently preoperative then surgery then Trastuzumab [6 mg/kg] d1 q21d for up to 52 wks			
48102 2009U	JBCRG-10, Japan	(5-fluorouracil [500mg/m2] + Epirubicin [100mg/m2] + Cyclophosphamide [500mg/m2] q3wk) × 4 then (Docetaxel [75mg/m2] + Cyclophosphamide [600mg/m2] q3wk + Trastuzumab [4mg/kg loading dose then 2mg/kg thereafter] qwk or [8mg/kg loading dose then 6mg/kg thereafter q3wk]) × 4 preoperative vs (Docetaxel [75mg/m2] + Cyclophosphamide [600mg/m2] q3wk + Trastuzumab [4mg/kg loading dose then 2mg/kg thereafter] qwk or [8mg/kg loading dose then 6mg/kg thereafter q3wk]) × 4 then (5-fluorouracil [500mg/m2] + Epirubicin [100mg/m2] + Cyclophosphamide [500mg/m2] q3wk) × 4 preoperative	42	Yes	✓ Abstract 2020[8]
48103 2009V	JBCRG-09, Japan	(5-Fluoro-Uracil [500 mg/m2 iv] + Epirubicin [100 mg/m2 iv] + Cyclophosphamide [500 mg/m2 iv]) q3wk × 3 then (Docetaxel [75 mg/m2 iv] + Cyclophosphamide [600 mg/m2 iv]) q3wk × 3 preoperative vs (Docetaxel [75 mg/m2 iv] + Cyclophosphamide [600 mg/m2 iv]) q3wk × 3 then (5-Fluoro-Uracil [500 mg/m2 iv] + Epirubicin [100 mg/m2 iv] + Cyclophosphamide [500 mg/m2 iv]) q3wk × 3 preoperative	128	Yes	✓ 2020[9]
1223 2010G	SWOG S0800, USA	(Doxorubicin [60 mg/m2 iv d1] + cyclophosphamide [600mg/m2 iv d1] + pegfilgrastim [6mg sc d2]) q2wk × 6 then Nab-paclitaxel [100mg/m2 iv d1] qwk × 12 preoperative vs Nab-paclitaxel [100mg/m2 iv] qwk × 12 then (doxorubicin [60 mg/m2 iv d1] + cyclophosphamide [600mg/m2 iv d1] + pegfilgrastim [6mg sc d2]) q2wk × 6 preoperative	116	Yes	✓ 2016[94]
29902 2010J	NeoSAMBA, Brazil	(5-Fluoro-Uracil [500mg/m2 iv] + Doxorubicin [50mg/m2 iv] + Cyclophosphamide [500mg/m2 iv]) q21d × 3 then Docetaxel [100mg/m2 iv] q21d × 3 preoperative vs Docetaxel [100mg/m2 iv] q21d × 3 then (5-Fluoro-Uracil [500mg/m2 iv] + Doxorubicin [50mg/m2 iv] + Cyclophosphamide [500mg/m2 iv]) q21d × 3 preoperative	120	Yes	✓ 2020[95]

Cohort 7: Longer vs shorter duration of anthracycline

Trial & year code	Trial name	Comparisons	N	Received	Published
**(a) Longer versus shorter less than 6 months of polychemotherapy (anthracycline)**					
6601-02, 6604 1974D	Adjuvant Breast 74-063 A, USA<br>Adjuvant Breast 75-122 B	(Cyclophosphamide [500 mg/m2 iv] + Doxorubicin [45 mg/m2 iv]) q3wk × 10 vs (Cyclophosphamide [500 mg/m2 iv] + Doxorubicin [45 mg/m2 iv]) q3wk × 5	299	Yes	1987[96]
19305, 07, 09, 11 1982Z	Metaneira B Stage II and III, Greece	Oophorectomy + (Vincristine [0.9 mg/m2 iv d1] + Doxorubicin [35 mg/m2 iv d1] + Cyclophosphamide [100 mg po tds d1-4]) qm × 3 + (5-Fluoro-Uracil [440 mg/m2 iv d1,8] + Doxorubicin [25 mg/m2 iv d1,8] + Cyclophosphamide [50 mg po tds d1-15]) × 7 vs Oophorectomy + (Vincristine [0.9 mg/m2 iv d1]	308	Yes	No

		+ Doxorubicin [35 mg/m2 iv d1] + Cyclophosphamide [100 mg po tds d1-4]) qm × 3			
13001<br>1986P1	FASG GFEA 01, France	(Epirubicin[50mg/m² iv] + 5-Fluoro-Uracil [500mg/m2 iv] + Cyclophosphamide [500mg/m2 iv]) q21d × 3 then Radiotherapy then (Epirubicin[50mg/m² iv] + 5-Fluoro-Uracil [500mg/m2 iv] + Cyclophosphamide [500mg/m2 iv]) q21d × 3 vs (Epirubicin[50mg/m² iv] + 5-Fluoro-Uracil [500mg/m2 iv] + Cyclophosphamide [500mg/m2 iv]) q21d × 3 then Radiotherapy	421	Yes	✓ 2003[97]
28001<br>1987Q	PRONACAM 87, Argentina	(Cyclophosphamide [400mg/m²] + Methotrexate [30mg/m²] + 5-Fluoro-Uracil [400mg/m²] + Epirubicin[60mg/m²] + Prednisone [40mg/m² po] × 4d) q21d × 6 vs (Cyclophosphamide [400mg/m²] + Methotrexate [30mg/m²] + 5-Fluoro-Uracil [400mg/m²] + Epirubicin[60mg/m²] + Prednisone [40mg/m² po] × 4d) q21d × 4	556	No	✓ Abstract 1995[98]
8322<br>2002B	CALGB 40101, USA	(Doxorubicin [60mg/m²] + Cyclophosphamide [600mg/m²]) q3wk × 6 vs (Doxorubicin [60mg/m²] + Cyclophosphamide [600mg/m²]) q3wk × 4	282	Yes	✓ 2012[70]
33206<br>2002N	PACS 05, France	(5-Fluorouracil [500 mg/m2 iv] + Epirubicin [100 mg/m2 iv] + Cyclophosphamide [500 mg/m2 iv]) q3wk × 6 vs (5-Fluorouracil [500 mg/m2 iv] + Epirubicin [100 mg/m2 iv] + Cyclophosphamide [500 mg/m2 iv]) q3wk × 4	1515	Yes	✓ 2017[99]
8323<br>2002X1	CALGB 40101, USA	(Doxorubicin [60mg/m² iv over 10-15min d1] + Cyclophosphamide [600mg/m² iv bolus d1]) q2wk × 6 vs (Doxorubicin [60mg/m² iv over 10-15min d1] + Cyclophosphamide [600mg/m² iv bolus d1]) q2wk × 4	1300	Yes	✓ 2012[70]
4762/78<br>2004C	NSABP B-36, USA	(5-Fluoro-Uracil [500mg/m2] + Epirubicin[100mg/m2] + Cyclophosphamide [500mg/m2]) q21d × 6 then celecoxib placebo [bid po] × 3yr vs (Doxorubicin [60mg/m2] + Cyclophosphamide [600mg/m2]) q21d × 4 then celecoxib placebo [bid po] × 3yr<br>Or<br>(5-Fluoro-Uracil [500mg/m2] + Epirubicin[100mg/m2] + Cyclophosphamide [500mg/m2]) q21d × 6 then celecoxib [bid po] × 3yr vs (Doxorubicin [60mg/m2] + Cyclophosphamide [600mg/m2]) q21d × 4 then celecoxib [bid po] × 3yr<br>Or<br>(5-Fluoro-Uracil [500mg/m2] + Epirubicin[100mg/m2] + Cyclophosphamide [500mg/m2]) q21d × 6 vs (Doxorubicin [60mg/m2] + Cyclophosphamide [600mg/m2]) q21d × 4	2722	No	✓ 2022[72]
**(b) Longer versus shorter <6 months of polychemotherapy (anthracycline and taxane)**					
33103<br>1997H2	Multicentre France Phase II Neoadjuvant Chemotherapy Trial	(Doxorubicin [60mg/m² iv] + Paclitaxel [200mg/m² iv]) q3wk × 6 preoperative vs (Doxorubicin [60mg/m² iv] + Paclitaxel [200mg/m² iv]) q3wk × 4 preoperative	232	No	✓ Abstract 2002[100]

2619<br>1999M	ABCSG-14, Austria	(Epirubicin[75mg/m^2 d1] + Docetaxel [75mg/m^2 d1] + filgrastim [5mcg/kg sc d3-10]) q3wk × 6 preoperative vs (Epirubicin[75mg/m^2 d1] + Docetaxel [75mg/m^2 d1] + filgrastim [5mcg/kg sc d3-10]) q3wk × 3 preoperative	292	Yes - ineligible	✓ 2007[101]
35003<br>2001S2	GeparTrio / GBG 24, Germany	(Doxorubicin [50mg/m^2 d1] + Cyclophosphamide [500mg/m^2 d1] + Docetaxel [75mg/m^2 d1]) q21d × 2 then (Doxorubicin [50mg/m^2 d1] then Cyclophosphamide [500mg/m^2 d1] + Docetaxel [75mg/m^2 d1]) q21d × 6 preoperative vs (Doxorubicin [50mg/m^2 d1] + Cyclophosphamide [500mg/m^2 d1] + Docetaxel [75mg/m^2 d1]) q21d × 2 then (Doxorubicin [50mg/m^2 d1] then Cyclophosphamide [500mg/m^2 d1] + Docetaxel [75mg/m^2 d1]) q21d × 4 preoperative	1390	Yes	✓ 2010[102]
53501<br>2003%	Chungnam Neoadjuvant trial, South Korea	(Epirubicin [75 mg/m2] + Docetaxel [75 mg/m2]) q3wk × 6 preoperative vs (Epirubicin [75 mg/m2] + Docetaxel [75 mg/m2]) q3wk × 4 preoperative	176	No	2009[103]

Cohort 8: Higher vs lower dose anthracycline

Trial & year code	Trial name	Comparisons	N	Received	Published
**(a) Increase in anthracycline dose/course and total dose (<100mg A or 150mg E difference)**					
13001<br>1986P1	GFEA 01, France	(Epirubicin[75mg/m^2 iv] + 5-Fluoro-Uracil [500mg/m2 iv] + Cyclophosphamide [500mg/m2 iv]) q21d × 3 then Radiotherapy vs (Epirubicin[50mg/m^2 iv] + 5-Fluoro-Uracil [500mg/m2 iv] + Cyclophosphamide [500mg/m2 iv]) q21d × 3 then Radiotherapy	409	Yes	✓ 2003[97]
8316<br>1994D1	CALGB 9344, USA	(Cyclophosphamide [600mg/m^2] + Doxorubicin [75mg/m^2]) q3wk × 4 or (Cyclophosphamide [600mg/m^2] + Doxorubicin [90mg/m^2] + filgrastim [5 or 10µg/kg/d sc d2-12]) q3wk × 4 vs (Cyclophosphamide [600mg/m^2] + Doxorubicin [60mg/m^2]) q3wk × 4	1580	Yes	✓ 2003[24]
8316<br>1994D2	CALGB 9344, USA	(Cyclophosphamide [600mg/m^2] + Doxorubicin [75mg/m^2]) q3wk × 4 then Paclitaxel [175mg/m^2] q3wk × 4 or (Cyclophosphamide [600mg/m^2] + Doxorubicin [90mg/m^2] + filgrastim [5 or 10µg/kg/d sc d2-12]) q3wk × 4 then Paclitaxel [175mg/m^2] q3wk × 4 vs (Cyclophosphamide [600mg/m^2] + Doxorubicin [60mg/m^2]) q3wk × 4 then Paclitaxel [175mg/m^2] q3wk × 4	1590	Yes	✓ 2003[24]
46301<br>2005Z	CECOG/BREAST.2.2.005, Austria	(5-Fluorouracil [500 mg/m^2 iv] + Epirubicin [90mg/m^2 iv] + Cyclophosphamide [500mg/m^2 iv] d1 + pegfilgrastim [6mg sc] d2) q14d × 6 vs (5-Fluorouracil [500 mg/m^2 iv] + Epirubicin [75mg/m^2 iv] + Cyclophosphamide [500mg/m^2 iv] d1 + pegfilgrastim [6mg sc] d2) q14d × 6	50	No	✓ 2008[104]
**(b) Increase in anthracycline dose/course and total dose (>100mg A or 150mg E difference)**					
13006,<br>13008<br>1990C3+5	GFEA 05, France	(Epirubicin[100mg/m^2] + 5-Fluoro-Uracil [500mg/m^2] + Cyclophosphamide [500mg/m^2]) × 6 vs (Epirubicin[50mg/m^2] + 5-Fluoro-Uracil [500mg/m^2] + Cyclophosphamide [500mg/m^2]) × 6	565	Yes	✓ 2005[105]

20006-07 1992N	C/9/91 / HMFEC, UK	(Cyclophosphamide [600mg/m² d1] + Epirubicin[75mg/m² d1] + 5-Fluoro-Uracil [600mg/m² d1]) q3wk × 8 vs (Cyclophosphamide [600mg/m² d1] + Epirubicin[50mg/m² d1] + 5-Fluoro-Uracil [600mg/m² d1]) q3wk × 8<br>Or<br>(Cyclophosphamide [600mg/m² d1] + Epirubicin[75mg/m² d1] + 5-Fluoro-Uracil [600mg/m² d1]) q3wk × 8 + Tamoxifen [20mg/d] × 5yr vs (Cyclophosphamide [600mg/m² d1] + Epirubicin[50mg/m² d1] + 5-Fluoro-Uracil [600mg/m² d1]) q3wk × 8 + Tamoxifen [20mg/d] × 5yr<br>Or	785	Yes	✓ 2016[106]
12911 2005S	Tianjin Chemotherapy trial, China	(Cyclophosphamide [600 mg/m2] + Epirubicin [100 mg/m2] + 5-Fluorouracil [500 mg/m2]) q21d × 6 vs (Cyclophosphamide [600 mg/m2] + Epirubicin [60 mg/m2] + 5-Fluorouracil [500 mg/m2] q21d × 6)	98	No	✓ Abstract 2007[107]
**(c) Increase in anthracycline and other drug dose/course and total dose**					
3504 0977S	B1/ Protocol 7701, Italy	(Cyclophosphamide [50-100-150 mg/m2 po d1-14] + Methotrexate [20-40-60 mg/m2 iv d1,8] + 5-Fluoro-Uracil [400-600-800 mg/m2 iv d1,8] + Prednisolone [40 mg/m2 im d1-14]) q4w × 6 then (Doxorubicin [50-75 mg/m2 iv d1,8] + Vincristine [1.4 mg/m2 iv d1,8]) q4wk × 4 [intensification every 2 cycles] vs (Cyclophosphamide [100 mg/m2 po d1-14] + Methotrexate [40 mg/m2 iv d1,8] + 5-Fluoro-Uracil [600 mg/m2 iv d1,8] + Prednisolone [40 mg/m2 im d1-14]) q4wk × 6 then (Doxorubicin [60 mg/m2 iv d1,8] + Vincristine [1.4 mg/m2 iv d1,8]) q4wk × 4	140	Yes	✓ Abstract 1985[108]
8307 1985A	CALGB Study CLB-8541, USA	(Cyclophosphamide [600 mg/m2 iv d1] + Doxorubicin [60 mg/m2 iv d1] + 5-Fluoro-Uracil [600 mg/m2 iv d1,8]) q4w × 4 vs (Cyclophosphamide [300 mg/m2 iv d1] + Doxorubicin [30 mg/m2 iv d1] + 5-Fluoro-Uracil [300 mg/m2 iv d1,8]) q4w × 4	1050	Yes	✓ 1994[110]
25701 1988R	Jules Bordet Classical CMF Versus EC Standard Dose Versus EC High Dose in Node Positive Breast Cancer, Belgium	(Epirubicin[100mg/m² iv d1] + Cyclophosphamide [830mg/m² iv] d1) q21d × 8 vs (Epirubicin[60mg/m² iv d1] + Cyclophosphamide [500mg/m² iv d1]) q21d × 8	537	Yes	✓ 2009[109]
1132 1991F	MD Anderson Neoadjuvant Trial, USA	(5-Fluoro-Uracil [1200mg/m²] + Doxorubicin [60mg/m²] + Cyclophosphamide [1000mg/m²] + G-CSF [5µg/kg/d d4-17]) q18d × 4 preoperative vs (5-Fluoro-Uracil [1000mg/m²] + Doxorubicin [50mg/m²] + Cyclophosphamide [500mg/m²]) q21d × 4 preoperative	112	No	✓ Abstract 1999[111]
13013 1993@	GFEA Inflammatory BC trial, France	(5-Fluoro-Uracil [750 mg/m2 iv d1-4] + Epirubicin [35 mg/m2 iv d2-4] + Cyclophosphamide [400 mg/m2 iv d2-4] + lenograstim) preoperative then (Radiotherapy or Surgery) then (5-Fluoro-Uracil [500 mg/m2 iv d1] + Epirubicin [75 mg/m2 iv d1] + Cyclophosphamide [500 mg/m2 iv d1]) × 4 postoperative vs (5-Fluoro-Uracil [500 mg/m2 iv d1] + Epirubicin [75 mg/m2 iv d1] + Cyclophosphamide [500 mg/m2 iv d1]) preoperative then (Radiotherapy or Surgery) then (5-Fluoro-Uracil [500 mg/m2 iv d1] + Epirubicin [75 mg/m2 iv d1] + Cyclophosphamide [500 mg/m2 iv d1]) × 4 postoperative	Not known	No	

2616 1994Q	ABCSG Trial X, Austria	(Epirubicin[60mg/m2 iv d1,d2] + Cyclophosphamide [600mg/m2 iv d1,d2] + Uromitexan + filgrastim [5mcg/kg sc d3-13]) q3wk × 4 then (Cyclophosphamide [600mg/m2] + Methotrexate [40mg/m2] + 5-Fluoro-Uracil [600mg/m2]) q3wk × 4 vs (Epirubicin[60mg/m2 iv d1] + Cyclophosphamide [600mg/m2 iv d1]) q3w × 4 then (Cyclophosphamide [600mg/m2] + Methotrexate [40mg/m2] + 5-Fluoro-Uracil [600mg/m2]) q3wk × 4	112	Yes	No
21102 1996L	OCSGL Trial, Poland	(Cyclophosphamide [75mg/m² po d1-14] + Epirubicin[60mg/m² iv d1,8] + 5-Fluoro-Uracil [500mg/m² iv d1,8]) q28d × 6 then Tamoxifen × 3yr vs (5-Fluoro-Uracil [500mg/m² iv] + Epirubicin[50mg/m² iv] + Cyclophosphamide [500mg/m² iv]) q21d × 6 then Tamoxifen × 3yr	87	No	✓ 2003[112]
16213 1996V	Gustave-Roussy Neoadjuvant Chemotherapy Trial, France	(5-Fluoro-Uracil [1000mg/m² d1] + Cyclophosphamide [1000mg/m² d1] + Epirubicin[100mg/m² d1 iv]) q21d × 4 + G-CSF [5µg/m² sc d6-11] preoperative vs (5-Fluoro-Uracil [500mg/m² d1] + Cyclophosphamide [500mg/m² d1] + Epirubicin[60mg/m² d1 iv]) q21d × 4 preoperative	84	Yes	✓ Abstract 2003[113]
21611 2001Q	SBG 2000-1 / SBG CEF-60, Sweden	(5-Fluoro-Uracil [600mg/m²] + Epirubicin[75-90mg/m²] + Cyclophosphamide [900-1200mg/m²]) q3wk × 6 vs (5-Fluoro-Uracil [600mg/m²] + Epirubicin[60mg/m²] + Cyclophosphamide [600mg/m²]) q3wk × 6	1052	Yes	✓ 2018[114]
2024 2004=	SBG 2004-1 Phase II Feasibility, Sweden	(Epirubicin[start 90mg/m²] + Cyclophosphamide [start 600mg/m²]) escalating q2wk × 4 then Docetaxel [start 75mg/m²] escalating q2wk × 4 + lenograstim [5mcg/kg d4-11] or pegfilgrastim [6mg d2] + Ciprofloxacin vs (Epirubicin[90mg/m²] + Cyclophosphamide [600mg/m²]) q2wk × 4 then Docetaxel [75mg/m²] q2wk × 4 + lenograstim [5mcg/kg d4-11] or pegfilgrastim [6mg d2] + Ciprofloxacin	84	Yes	✓ 2018[115]

Reference list

1. Blum JL, Flynn PJ, Yothers G, et al. Anthracyclines in early breast cancer: the ABC Trials--USOR 06-090, NSABP B-46-I/USOR 07132, and NSABP B-49 (NRG Oncology). J Clin Oncol; 2017. p. 2647-55.
2. Chen X, Ye G, Zhang C, Li X, Shen K. Non-anthracycline-containing docetaxel and cyclophosphamide regimen is associated with sustained worse outcome compared with docetaxel, anthracycline and cyclophosphamide in neoadjuvant treatment of triple negative and HER2-positive breast cancer patients: updated follow-up data from NATT study. Chin J Cancer Res; 2016. p. 561-9.
3. Ejlertsen B, Tuxen MK, Jakobsen EH, et al. Adjuvant cyclophsphamide and docetaxel with or without epirbicin for early TOP2A-normal breast cancer: DBCG 07-READ, an open-label, phase III, randomized trial. J Clin Oncol; 2017. p. 2639-46.
4. Abe H, Mori T, Kawai Y, et al. Feasibility study of docetaxel and cyclophosphamide six- cycle therapy as adjuvant chemotherapy for Japanese human epidermal growth factor receptor 2-negative breast cancer patients. Asian Pacific Journal of Cancer Prevention: Apjcp; 2013. p. 4835-8.
5. Narui K, Ishikawa T, Shimizu D, et al. Anthracycline could be essential for triple-negative breast cancer: A randomised phase II study by the Kanagawa Breast Oncology Group (KBOG) 1101. The Breast; 2019. p. 1-9.
6. Janni W, Nitz U, Rack BK, et al. Pooled analysis of two randomized phase III trials (PlanB/SuccessC) comparing six cycles of docetaxel and cyclophosphamide to sequential anthracycline taxane chemotherapy in patients with intermediate and high risk HER2-negative early breast cancer (n=5,923). Journal of Clinical Oncology; 2018. p. A522.
7. Nitz U, Gluz O, Clemens M, et al. West German Study PlanB trial: adjuvant four cycles of epirubicin and cyclophosphamide plus docetaxel versus six cycles of docetaxel and cyclophosphamide in HER2-negative early breast cancer. J Clin Oncol; 2019. p. 799-808.
8. Ueno T, Masuda N, Sato N, et al. Multicenter study of primary systemic therapy with docetaxel, cyclophosphamide and trastuzumab for HER2-positive operable breast cancer: the JBCRG-10 study. Jpn J Clin Oncol; 2020. p. 3-11.
9. Ishiguro H, Masuda N, Sato N, et al. A randomized study comparing docetaxel/cyclophosphamide (TC), 5-fluorouracil/epirubicin/cyclophosphamide (FEC) followed by TC, and TC followed by FEC for patients with hormone receptor-positive HER2-negative primary breast cancer. Breast Cancer Res Treat; 2020. p. 715-24.
10. Yu K-D, Liu X-Y, Chen L, et al. Anthracycline-free or short-term regimen as adjuvant chemotherapy for operable breast cancer: A phase III randomized non-inferiority trial. The Lancet Regional Health - Western Pacific; 2021. p. 100158.
11. Watanabe T, Kuranami M, Inoue K, et al. Comparison of an AC-taxane versus AC-free regimen and paclitaxel versus docetaxel in patients with lymph node-positive breast cancer: Final results of the National Surgical Adjuvant Study of Breast Cancer 02 trial, a randomized comparative phase 3 study. Cancer; 2017. p. 759-68.
12. Bougnoux P, Combe M, Goudier M-J, Facchini T, Lotz V. Phase II randomized trial of docetaxel (D) montherapy vs epirubicin plus docetaxel (ED) as neoadjuvant treatment of operable breast cancer (BC). J Clin Oncol; 2004. p. 27s, A599.
13. Yoo C, Kim S-B, Ahn J-H, et al. A randomized phase II trial of capecitabine plus vinorelbine followed by docetaxel versus adriamycin plus cyclophosphamide followed by docetaxel as neoadjuvant chemotherapy for breast cancer. Cancer Res Treat; 2015. p. 406-15.
14. Piccart M, J. van 't Veer L, Poncet C, et al. 70-gene signature as an aid for treatment decisions in early breast cancer: updated results of the phase 3 randomised MINDACT trial with an exploratory analysis by age. Lancet Oncol; 2021. p. 476-88.
15. Pierga J, Asselain B, Alsafadi S, et al. A prospective randomized trial evaluating gene expression arrays to select neoadjuvant chemotherapy regimen for operable breast cancer: first report of the Remagus04 trial. Annals of Oncology; 2012. p. ixe3, A245O.
16. Steger GG, Greil R, Hubalek M, et al. Bevacizumab in combination with docetaxel+trastuzumab +/- non-pegylated liposomal doxorubicin: Final results of ABCSG-32, a prospective, randomized phase II-study. Cancer Res; 2015. p. P3-11-06.
17. Wang J, Li Q, Mu Y, et al. A randomized phase III trial comparing dose-dense epirubicin and cyclophosphamide (ECdd) followed by paclitaxel (T) with paclitaxel plus carboplatin (PCdd) as adjuvant chemotherapy for early triple-negative breast cancer patients with high-recurrence risk. J Clin Oncol; 2019. p. A528.
18. Slamon D, Eiermann W, Robert N, et al. Adjuvant trastuzumab in HER2-positive breast cancer. N Engl J Med; 2011. p. 1273-83.

19. Zhang P, Yin Y, Mo H, et al. Better pathologic complete response and relapse-free survival after carboplatin plus paclitaxel compared with epirubicin plus paclitaxel as neoadjuvant chemotherapy for locally advanced triple-negative breast cancer: a randomized phase 2 trial. Oncotarget; 2016. p. 60647-56.
20. Schneeweiss A, Chia S, Hickish T, et al. Long-term efficacy analysis of the randomised, phase II TRYPHAENA cardiac safety study: Evaluating pertuzumab and trastuzumab plus standard neoadjuvant anthracycline-containing and anthracycline-free chemotherapy regimens in patients with HER2-positive early breast cancer. Eur J Cancer; 2018. p. 27-35.
21. Yuan P, Du F, Wang J, Ma F, Fan Y, Xu B. Comparison of four cycles epirubicin and cyclophosphamide (EC) followed by four cycles docetaxel (T) versus six cycles docetaxel and carboplatin (TP) as adjuvant chemotherapy for women with operable triple negative breast cancer. J Clin Oncol; 2016. p. A1068.
22. Huang L, Chen S, Yang W, et al. Efficacy and safety analysis of trastuzumab and paclitaxel based regimen plus carboplatin or epirubicin as neoadjuvant therapy for clinical stage II-III, HER2-positive breast cancer patients: a phase 2, open-label, multicenter, randomized trial. Oncotarget; 2015. p. 18683-92.
23. Yu K, Ye F, He M, et al. Effect of adjuvant paclitaxel and carboplatin on survival in women with triple-negative breast cancer: a phase 3 randomized clinical trial. JAMA Oncology; 2020. p. 1390-6.
24. Henderson IC, Berry DA, Demetri GD, et al. Improved outcomes from adding sequential paclitaxel but not from escalating doxorubicin dose in an adjuvant chemotherapy regimen for patients with node-positive primary breast cancer. J Clin Oncol; 2003. p. 976-83.
25. Bear HD, Anderson S, Brown A, et al. The effect on tumor response of adding sequential preoperative docetaxel to preoperative doxorubicin and cyclophosphamide: preliminary results from National Surgical Adjuvant Breast and Bowel Project Protocol B-27. J Clin Oncol; 2003. p. 4165-74.
26. Mamounas EP, Bryant J, Lembersky B, et al. Paclitaxel after doxorubicin plus cyclophosphamide as adjuvant chemotherapy for node-positive breast cancer: results from NSABP B-28. J Clin Oncol; 2005. p. 3686-96.
27. Bianco AR, De Laurentiis M, De Placido S, et al. Sequential epirubicin-docetaxel-CMF as adjuvant therapy for node-positive early-stage breast cancer: Subgroup analysis of the TAXit216 randomized trial. 2008 Breast Cancer Symposium. 2008; 2008. p. A187.
28. Vici P, Brandi M, Giotta F, et al. A multicenter phase III prospective randomized trial of high-dose epirubicin in combination with cyclophosphamide (EC) versus docetaxel followed by EC in node-positive breast cancer. GOIM (Gruppo Oncologico Italia Meridionale) 9902 study. Annals of Oncology; 2012. p. 1121-9.
29. Gonçalves A, Pierga J-Y, Ferrero J-M, et al. UNICANCER-PEGASE 07 study: a randomized phase III trial evaluating postoperative docetaxel-5FU regimen after neoadjuvant dose-intense chemotherapy for treatment of inflammatory breast cancer. Ann Oncol; 2015. p. 1692-7.
30. Schenker M, Badulescu F, Badulescu A, et al. Role of paclitaxel in neoadjuvant chemotherapy in stage IIA-IIIA breast cancer. Eur J Cancer Suppl; 2009. p. 284, A5079.
31. Polyzos, A.; Malamos, N.; Boukovinas, I.; Adamou, A.; Ziras, N.; Kalbakis, K.; Kakolyris, S.; Syrigos, K.; Papakotoulas, P.; Kouroussis, C.; Karvounis, N.; Vamvakas, L.; Christophyllakis, C. ; Athanasiadis, A.; Varthalitis, I.; Georgoulias, V., and Mavroudis, D. FEC versus sequential docetaxel followed by epirubicin/cyclophosphamide as adjuvant chemotherapy in women with axillary node-positive early breast cancer: a randomized study of the Hellenic Oncology Research Group (HORG). 2010; 119, (1): 95-104.
32. Fountzilas G, Skarlos D, Dafni U, et al. Postoperative dose-dense sequential chemotherapy with epirubicin, followed by CMF with or without paclitaxel, in patients with high-risk operable breast cancer: a randomized phase III study conducted by the Hellenic Cooperative Oncology Group. Ann Oncol; 2005. p. 1762-71.
33. Boccardo F, Amadori D, Guglielmini P, et al. Epirubicin followed by cyclophosphamide, methotrexate and 5-fluorouracil versus paclitaxel followed by epirubicin and vinorelbine in patients with high-risk operable breast cancer. Oncology; 2010. p. 274-81.
34. Oakman C, Francis PA, Crown J, et al. Overall survival benefit for sequential doxorubicin-docetaxel compared with concurrent doxorubicin and docetaxel in node-positive breast cancer--8-year results of the Breast International Group 02-98 phase III trial. Annals of Oncology; 2013. p. 1203-11.
35. Martin M, Rodriguez-Lescure A, Ruiz A, et al. Randomized phase 3 trial of fluorouracil, epirubicin, and cyclophosphamide alone or followed by Paclitaxel for early breast cancer. J Natl Cancer Inst; 2008. p. 805-14.
36. Joensuu H, Bono P, Kataja V, et al. Fluorouracil, epirubicin, and cyclophosphamide with either docetaxel or vinorelbine, with or without trastuzumab, as adjuvant treatments of breast cancer: final results of the FinHer Trial. J Clin Oncol; 2009. p. 5685-92.
37. Burnell M, Levine MN, Chapman J-AW, et al. Cyclophosphamide, epirubicin, and fluorouracil versus dose-dense epirubicin and cyclophosphamide followed by paclitaxel versus doxorubicin and cyclophosphamide followed by paclitaxel in node-positive or high-risk node-negative breast cancer. J Clin Oncol; 2010. p. 77-82.

38. Nitz U, Gluz O, Huober J, et al. Final analysis of the prospective WSG-AGO EC-Doc versus FEC phase III trial in intermediate-risk (pN1) early breast cancer: efficacy and predictive value of Ki67 expression. Ann Oncol; 2014. p. 1551-7.
39. Delbaldo C, Serin D, Mousseau M, et al. A phase III adjuvant randomised trial of 6 cycles of 5-fluorouracil-epirubicine-cyclophosphamide (FEC100) versus 4 FEC 100 followed by 4 Taxol (FEC-T) in node positive breast cancer patients (Trial B2000). Eur J Cancer; 2014. p. 23-30.
40. Tabchy A, Valero V, Vidaurre T, et al. Evaluation of a 30-gene paclitaxel, fluorouracil, doxorubicin, and cyclophosphamide chemotherapy response predictor in a multicenter randomized trial in breast cancer. Clin Cancer Res; 2010. p. 5351-61.
41. Martin M, Ruiz A, Ruiz Borrego M, et al. Fluorouracil, doxorubicin, and cyclophosphamide (FAC) versus FAC followed by weekly paclitaxel as adjuvant therapy for high-risk, node-negative breast cancer: results from the GEICAM/2003-02 study. Journal of Clinical Oncology; 2013. p. 2593-9.
42. Braun S, Kentenich C, Janni W, et al. Lack of effect of adjuvant chemotherapy on the elimination of single dormant tumor cells in bone marrow of high-risk breast cancer patients. J Clin Oncol; 2000. p. 80-6.
43. Malamos N, Kosmas C, Antonopoulos MJ, et al. Prospective randomized study of neoadjuvant chemotherapy (NACT) with paclitaxel/epirubicin (PE) versus fluorouracil/epirubicin/cyclophosphamide (FEC) in operable stage II-IIIA breast cancer (BC). Ann Oncol; 1998. p. A105P.
44. Kümmel S, Krocker J, Kohls A, et al. Randomised trial: survival benefit and safety of adjuvant dose-dense chemotherapy for node-positive breast cancer. Br J Cancer; 2006. p. 1237-44.
45. Gianni L, Baselga J, Eiermann W, et al. Phase III trial evaluating the addition of paclitaxel to doxorubicin followed by cyclophosphamide, methotrexate, and fluorouracil, as adjuvant or primary systemic therapy: European Cooperative Trial in Operable Breast Cancer. J Clin Oncol; 2009. p. 2474-81.
46. Del Mastro L, Levaggi A, Michelotti A, et al. 5-Fluorouracil, epirubicin and cyclophosphamide versus epirubicin and paclitaxel in node-positive early breast cancer: a phase-III randomized GONO-MIG5 trial. Breast Cancer Res Treat; 2016. p. 117-26.
47. Dieras V, Fumoleau P, Romieu G, et al. Randomized parallel study of doxorubicin plus paclitaxel and doxorubicin plus cyclophosphamide as neoadjuvant treatment of patients with breast cancer. J Clin Oncol; 2004. p. 4958-65.
48. Mackey JR, Martin M, Pienkowski T, et al. Adjuvant docetaxel, doxorubicin, and cyclophosphamide in node-positive breast cancer: 10-year follow-up of the phase 3 randomised BCIRG 001 trial. Lancet Oncology; 2013. p. 72-80.
49. Verma N, Harding D, Mohammadi A, et al. Image-based risk score to predict recurrence of ER+ breast cancer in ECOG-ACRIN Cancer Research Group E2197. Journal of Clinical Oncology; 2018. p. A540.
50. Martín M, Seguí MA, Antón A, et al. Adjuvant docetaxel for high-risk, node-negative breast cancer. N Engl J Med; 2010. p. 2200-10.
51. Mansi JL, Yellowlees A, Lipscombe J, et al. Five-year outcome for women randomised in a phase III trial comparing doxorubicin and cyclophosphamide with doxorubicin and docetaxel as primary medical therapy in early breast cancer: an Anglo-Celtic Cooperative Oncology Group study. Breast Cancer Res Treat; 2010. p. 787-94.
52. Brain EGC, Bachelot T, Serin D. Life-threatening complications from doxorubicin-docetaxel chemotherapy for breast cancer. JAMA; 2005. p. 2166-7.
53. Spielmann M, Roche H, Delozier T, et al. Trastuzumab for patients with axillary-node-positive breast cancer: results of the FNCLCC-PACS 04 trial. J Clin Oncol; 2009. p. 6129-34.
54. Semlglazov VF, Bojok AA, Arsumanov AS, et al. Breast conserving surgery after neoadjuvant chemotherapy paclitaxel + doxorubicin vs fluorouracil + doxorubicin + cyclophosphamide in locally advanced breast cancer. Breast Cancer Res Treat; 2002. p. S52, A159.
55. van Rossum AGJ, M. K, van Werkhoven E, et al. Adjuvant dose-dense doxorubicin-cyclophosphamide versus docetaxel-doxorubicin-cyclophosphamide for high-risk breast cancer: First results of the randomised MATADOR trial (BOOG 2004-04). Eur J Cancer; 2018. p. 40-8.
56. Roy C, Choudhury KB, Pal M, Saha A, Bag S, Banerjee C. Adjuvant chemotherapy with six cycles of AC regimen versus three cycles of AC regimen followed by three cycles of Paclitaxel in node-positive breast cancer. Indian Journal of Cancer; 2012. p. 266-71.
57. Albert JM, Buzdar AU, Guzman R, et al. Prospective randomized trial of 5-fluorouracil, doxorubicin, and cyclophosphamide (FAC) versus paclitaxel and FAC (TFAC) in patients with operable breast cancer: impact of taxane chemotherapy on locoregional control. Breast Cancer Res Treat; 2011. p. 421-7.
58. Hutcheon AW, Heys SD, Sarkar TK, et al. Docetaxel primary chemotherapy in breast cancer: a five year update of the Aberdeen trial. Breast Cancer Res Treat; 2003. p. S9, A11.

59. Coombes RC, Bliss JM, Espie M, et al. Randomized, phase III trial of sequential epirubicin and docetaxel versus epirubicin alone in postmenopausal patients with node-positive breast cancer. J Clin Oncol; 2011. p. 3247-54.
60. Coudert B, Asselain B, Campone M, et al. Extended benefit from sequential administration of docetaxel after standard fluorouracil, epirubicin, and cyclophosphamide regimen for node-positive breast cancer: the 8-year follow-up results of the UNICANCER-PACS01 trial. Oncologist; 2012. p. 900-9.
61. Ellis P, Barrett-Lee P, Johnson L, et al. Sequential docetaxel as adjuvant chemotherapy for early breast cancer (TACT): an open-label, phase III, randomised controlled trial. Lancet; 2009. p. 1681-92.
62. Bonnefoi H, Litiere S, Piccart M, et al. Pathological complete response after neoadjuvant chemotherapy is an independent predictive factor irrespective of simplified breast cancer intrinsic subtypes: a landmark and two-step approach analyses from the EORTC 10994/BIG 1-00 phase III trial. Annals of Oncology; 2014. p. 1128-36.
63. Janni W, Harbeck N, Rack B, et al. Randomised phase III trial of FEC120 vs EC-docetaxel in patients with high-risk node-positive primary breast cancer: final survival analysis of the ADEBAR study. Br J Cancer; 2016. p. 863-71.
64. Thomssen C, Kantelhardt EJ, Meisner C, et al. First planned efficacy analysis of the NNBC 3-Europe trial: Addition of docetaxel to anthracycline containing adjuvant chemotherapy in high risk node-negative breast cancer patients. Cancer Res; 2012. p. 199s, P1-13-13.
65. Brandi M, Sr., Petroni S, Stea B, et al. Randomized study testing as adjuvant chemotherapy the same primary (neoadjuvant) epirubicin/vinorelbine (EN) regimen versus docetaxel (TXT) in inflammatory breast cancer (IBC). J Clin Oncol; 2008. p. 28s, A590.
66. Jones SE, Savin MA, Holmes FA, et al. Phase III trial comparing doxorubicin plus cyclophosphamide with docetaxel plus cyclophosphamide as adjuvant therapy for operable breast cancer. J Clin Oncol; 2006. p. 5381-7.
67. Delaloge S, Piccart M, Rutgers E, et al. Standard anthracycline based versus docetaxel-capecitabine in early high clinical and/or genomic risk breast cancer in the EORTC 10041/BIG 3-04 MINDACT phase III trial. J Clin Oncol; 2020. p. Published online Feb 21, 2020.
68. Marcom PK, Barry W, Datto MB, et al. A randomized phase II trial evaluating the performance of genomic expression profiles to direct the use of preoperative chemotherapy for early-stage breast cancer. J Clin Oncol; 2010. p. 7S, TPS102.
69. Mathiesen RR, Nesland JM, Renolen A, et al. Prognostic impact of disseminated and circulating tumor cells in patients treated for locally advanced breast cancer. Cancer Res; 2011. p. 444s, P4-06-05.
70. Shulman LN, Berry DA, Cirrincione CT, et al. Comparison of doxorubicin and cyclophosphamide versus single-agent paclitaxel as adjuvant therapy for breast cancer in women with 0 to 3 positive axillary nodes: CALGB 40101 (Alliance). Journal of Clinical Oncology; 2014. p. 2311-7.
71. Sparano JA, Zhao F, Martino F, et al. Long-term follow-up of the E1199 phase III trial evaluating the role of taxane and schedule in operable breast cancer. J Clin Oncol; 2015. p. 2353-60.
72. Geyer CE, Jr., Bandos H, Rastogi P, et al. Definitive results of a phase III adjuvant trial comparing six cycles of FEC-100 to four cycles of AC in women with operable node-negative breast cancer: the NSABP B-36 trial (NRG Oncology). Breast Cancer Res Treat; 2022.
73. Saloustros E, Malamos N, Boukovinas I, et al. Dose-dense paclitaxel versus docetaxel following FEC as adjuvant chemotherapy in axillary node-positive early breast cancer: a multicenter randomized study of the Hellenic Oncology Research Group (HORG). Breast Cancer Research & Treatment; 2014. p. 591-7.
74. Abdel-Halim I, El-Sadda W. Adriamycin & cyclophosphamide (AC) followed by docetaxel (D) versus AC followed by paclitaxel (P) in operable node positive breast cancer. Breast; 2011. p. S65, P274.
75. Fountzilas G, Dafni U, Papadimitriou C, et al. Dose-dense sequential adjuvant chemotherapy followed, as indicated, by trastuzumab for one year in patients with early breast cancer: first report at 5-year median follow-up of a Hellenic Cooperative Oncology Group randomized phase III trial. BMC Cancer; 2014. p. 515.
76. Green MC, Buzdar AU, Smith T, et al. Weekly paclitaxel improves pathologic complete remission in operable breast cancer when compared with paclitaxel once every 3 weeks. J Clin Oncol; 2005. p. 5983-92.
77. Budd GT, Barlow WE, Moore HC, et al. SWOG S0221: a phase III trial comparing chemotherapy schedules in high-risk early-stage breast cancer. J Clin Oncol; 2015. p. 58-64.
78. Qi M, Li JF, Xie YT, Lu AP, Lin BY, Ouyang T. Weekly paclitaxel improved pathologic response of primary chemotherapy compared with standard 3 weeks schedule in primary breast cancer. Breast Cancer Res Treat; 2010. p. 197-202.
79. Walker LG, Eremin JM, Aloysius MM, et al. Effects on quality of life, anti-cancer responses, breast conserving surgery and survival with neoadjuvant docetaxel: a randomised study of sequential weekly versus three-weekly docetaxel following neoadjuvant doxorubicin and cyclophosphamide in women with primary breast cancer. BMC Cancer; 2011. p. 179.

80. Nuzzo F, Morabito A, Gravina A, et al. Effects on quality of life of weekly docetaxel-based chemotherapy in patients with locally advanced or metastatic breast cancer: results of a single-centre randomized phase 3 trial. BMC Cancer; 2011. p. 75.
81. Wildiers H, Dirix L, Neven P, et al. Delivery of adjuvant sequential dose-dense FEC-Doc to patients with breast cancer is feasible, but dose reductions and toxicity are dependent on treatment sequence. Breast Cancer Res Treat; 2009. p. 103-12.
82. Taghian AG, Abi Raad R, Assaad SI, et al. Paclitaxel decreases the interstitial fluid pressure and improves oxygenation in breast cancers in patients treated with neoadjuvant chemotherapy: clinical implications. J Clin Oncol; 2005. p. 1951-61.
83. Zelnak AB, Styblo TM, Rizzo M, et al. Final results from phase II trial of neoadjuvant docetaxel and capecitabine given sequentially or concurrently for HER2-negative breast cancers. Clinical Breast Cancer; 2013. p. 173-9.
84. Lee KS, Ro J, Nam BH, et al. A randomized phase-III trial of docetaxel/capecitabine versus doxorubicin/cyclophosphamide as primary chemotherapy for patients with stage II/III breast cancer. Breast Cancer Res Treat; 2008. p. 481-9.
85. Piedbois P, Serin D, Priou F, et al. Dose-dense adjuvant chemotherapy in node-positive breast cancer: docetaxel followed by epirubicin/cyclophosphamide (T/EC), or the reverse sequence (EC/T), every 2 weeks, versus docetaxel, epirubicin and cyclophosphamide (TEC) every 3 weeks. AERO B03 randomized phase II study. Ann Oncol; 2007. p. 52-7.
86. Makris A, Creighton C, Osborne KC, et al. Predictive gene expression patterns of response to adriamycin and cyclophosphamide (AC) in human breast cancers. J Clin Oncol; 2007. p. 9s, A527.
87. Miyashita M, Kohno N, Takao S, et al. Randomized phase II clinical trial of comparison of epirubicin (E) plus cyclophosphamide (C) and weekly paclitaxel (P) as primary systemic therapy (PST) in patients (pts) with stages II and III breast cancer (BC). J Clin Oncol; 2008. p. 23s, A568.
88. Puhalla S, Mrozek E, Young D, et al. Randomized phase II adjuvant trial of dose-dense docetaxel before or after doxorubicin plus cyclophosphamide in axillary node-positive breast cancer. J Clin Oncol; 2008. p. 1691-7.
89. Alamgeer M, Ganju V, Kumar B, et al. Changes in aldehyde dehydrogenase-1 expression during neoadjuvant chemotherapy predict outcome in locally advanced breast cancer. Breast Cancer Research; 2014. p. R44.
90. Earl HM, Vallier AL, Hiller L, et al. Effects of the addition of gemcitabine, and paclitaxel-first sequencing, in neoadjuvant sequential epirubicin, cyclophosphamide, and paclitaxel for women with high-risk early breast cancer (Neo-tAnGo): an open-label, 2x2 factorial randomised phase 3 trial. Lancet Oncology; 2014. p. 201-12.
91. Martin M, Romero A, Cheang MCU, et al. Genomic predictors of response to doxorubicin versus docetaxel in primary breast cancer. Breast Cancer Research and Treatment; 2011. p. 127-36.
92. Abe H, Mori T, Kawai Y, Cho H, Kubota Y, Umeda T. Feasibility and toxicity of docetaxel before or after fluorouracil, epirubicin and cyclophosphamide as adjuvant chemotherapy for early breast cancer. Int J Clin Oncol; 2013. p. 487-91.
93. Buzdar AU, Suman VJ, Meric-Bernstam F. Disease-free and overall survival among patients with operable HER2-positive breast cancer treated with sequential vs concurrent chemotherapy. The ACOSOG Z1041 (Alliance) randomized clinical trial. JAMA Oncol; 2019. p. 45-50.
94. Nahleh ZA, Barlow WE, Hayes DF, et al. SWOG S0800 (NCI CDR0000636131): addition of bevacizumab to neoadjuvant nab-paclitaxel with dose-dense doxorubicin and cyclophosphamide improves pathologic complete response (pCR) rates in inflammatory or locally advanced breast cancer. Breast Cancer Res Treat; 2016. p. 485-95.
95. Bines J, Small I, Sarmento R, et al. Does the sequence of anthracycline and taxane matter? The NeoSAMBA trial. Oncologist; 2020. p. 758–64.
96. Griem K, Henderson I, Gelman R, et al. The 5 year results of a randomized trial of adjuvant radiation therapy after chemotherapy in breast cancer patients treated with mastectomy. J Clin Oncol; 1987. p. 1546-55.
97. Fumoleau P, Kerbrat P, Romestaing P, et al. Randomized trial comparing six versus three cycles of epirubicin-based adjuvant chemotherapy in premenopausal, node-positive breast cancer patients: 10-year follow-up results of the French Adjuvant Study Group 01 trial. J Clin Oncol; 2003. p. 298-305.
98. Chacon R, Blajman C, Galvez C, et al. Adjuvant chemotherapy for premenopausal, stage ii, node-positive breast cancer patients (pronacam 87): first analysis. The pronacam group (meeting abstract). Proc Annu Meet Am Soc Clin Oncol; 1995. p. A162.
99. Kerbrat P, Desmoulins I, Roca L, et al. Optimal duration of adjuvant chemotherapy for high-risk node-negative (N-) breast cancer patients: 6-year results of the prospective randomised multicentre phase III UNICANCER-PACS 05 trial (UCBG-0106). Eur J Cancer; 2017. p. 166-75.

100. Romieu G, Tubiana-Hukin M, Fumoleau P, et al. A multicenter randomized phase II study of 4 or 6 cycles of adriamycin/Taxol [R] (paclitaxel) (AT) as neoadjuvant treatment of breast cancer (BC). Ann Oncol; 2002. p. 33, A118.
101. Steger GG, Galid A, Gnant M, et al. Pathologic complete response with six compared with three cycles of neoadjuvant epirubicin plus docetaxel and granulocyte colony-stimulating factor in operable breast cancer: results of ABCSG-14. J Clin Oncol; 2007. p. 2012-8.
102. Huober J, von Minckwitz G, Denkert C, et al. Effect of neoadjuvant anthracycline-taxane-based chemotherapy in different biological breast cancer phenotypes: overall results from the GeparTrio study. Breast Cancer Res Treat; 2010. p. 133-40.
103. Han S, Kim J, Lee J, et al. Comparison of 6 cycles versus 4 cycles of neoadjuvant epirubicin plus docetaxel chemotherapy in stages II and III breast cancer. Eur J Surg Oncol; 2009. p. 583-7.
104. Kahan Z, Spanik S, Wagnerova M, et al. Feasibility of two dose-dense FEC regimens with growth factor support for adjuvant therapy in patients with early breast cancer: Results from a randomised study of the Central European Cooperative Oncology Group (CECOG). Breast Cancer Res Treat; 2008. p. 557-63.
105. Bonneterre J, Roche H, Kerbrat P, et al. Epirubicin increases long-term survival in adjuvant chemotherapy of patients with poor-prognosis, node-positive, early breast cancer: 10-year follow-up results of the French Adjuvant Study Group 05 randomized trial. J Clin Oncol; 2005. p. 2686-93.
106. Coombes RC, Kilburn LS, Tubiana-Mathieu N, et al. Epirubicin dose and sequential hormonal therapy-Mature results of the HMFEC randomised phase III trial in premenopausal patients with node positive early breast cancer. Eur J Cancer; 2016. p. 146-53.
107. Hui R, Zhang M, Hao XM, Zhang J. [Comparison of tolerance and toxicity of CEF-100 regimen versus CEF-60 regimen as adjuvant therapy for breast cancer] [Chinese]. Chung Hua Chung Liu Tsa Chih; 2007. p. 871-4.
108. Brambilla C, Rossi A, Valagussa P, Bonadonna G. Adjuvant chemotherapy in postmenopausal women: Results of sequential noncross resistant regimens. World J Surg; 1985. p. 728-37.
109. de Azambuja E, Paesmans M, Beauduin M, et al. Long-term benefit of high-dose epirubicin in adjuvant chemotherapy for node-positive breast cancer: 15-year efficacy results of the Belgian multicentre study. J Clin Oncol; 2009. p. 720-5.
110. Wood WC, Budman DR, Korzun AH, et al. Dose and dose intensity of adjuvant chemotherapy for stage II, node positive breast carcinoma. N Engl J Med; 1994. p. 1253-9.
111. Dhingra K, Esparza-Guerra L, Valero V, et al. A Phase III Randomized Trial of Dose-Intensive, Neoadjuvant 5FU, Doxorubicin, Cyclophosphamide (FAC) with G-CSF (filgrastim) in Locally Advanced Breast Cancer (LABC)-Efficacy and Safety Data. Proc Annu Meet Am Soc Clin Oncol; 1999. p. A278.
112. Dyczka J, Czernek U, Pluzanska A. Evaluation of efficacy three schedules with different doses of epirubicin in adjuvant treatment of breast cancer with high risk factors - Randomized clinical trial. Onkol Pols; 2003. p. 11-3.
113. Pélissier P, Delaloge S, Mathieu M-C, et al. Intensified anthracyclin doses do not improve clinical and pathological responses to neoadjuvant FEC for operable breast cancer (BC): results of a multicenter randomized trial. Proc Annu Meet Am Soc Clin Oncol; 2002. p. 64a, A254.
114. Lindman H, Andersson M, Ahlgren J, et al. A randomised study of tailored toxicity-based dosage of fluorouracil-epirubicin-cyclophosphamide chemotherapy for early breast cancer (SBG 2000-1). Eur J Cancer; 2018. p. 79-86.
115. Matikas A, Margolin S, Hellstrom M, et al. Long-term safety and survival outcomes from the Scandinavian Breast Group 2004-1 randomized phase II trial of tailored dose-dense adjuvant chemotherapy for early breast cancer. Breast Cancer Res Treat; 2018. p. 349-55.
116. Mavoudis D, Matikas A, Malamos N, et al. Dose-dense FEC followed by docetaxel versus docetaxel plus cyclophosphamide in women with HER2-negative, axillary lymph node-positive early breast cancer: a multicentre randomized study by the Hellenic Oncology Research Group (HORG). Annals of Oncology; 2016. p.1873-1878.

Appendix 2 – EBCTCG seventh cycle: List of variables for taxane trials

Either using the codes we suggest below or using your own codes, please extract from your dataset the variables that correspond most closely to the items listed below and send them to us. Please provide one record for each woman ever randomised (including anyonewho was randomised and then was later categorised as ineligible, withdrawn, unevaluable, lost or "protocol deviant" – but, please tell us in question 9 which patients your group's preferred analyses would exclude, and why).

For trials where a dataset has previously been sent to the EBCTCG it is probably easiest and most reliable to update by re-sending all variables. If, however, this would cause difficulties then you can send only the additional variables; let us know if you want a file of the data you previously sent and we will provide it.

If any variable is not available or not applicable, please omit it and send only the remaining variables. If you have any of the requested variables in your records in a form that would require substantial additional work to supply (eg computerisation, or manual coding), please feel free to omit them for now, but in your cover document please tell us of their existence.

- Please send your data in a separate Excel spreadsheet for each separate trial, if possible.
- Please send a cover document giving all your coding conventions (including your format for dates).
- Please send your data to: bc.overview@ctsu.ox.ac.uk with your research group's name (and/or the EBCTCG number for your research group) and your group's name for the trial in the subject line.

If you have any questions about this data request, please contact the EBCTCG secretariat on bc.overview@ndph.ox.ac.uk (tel: +44-1865-743852). All data supplied to the secretariat will be held securely and treated confidentially, in accordance with UK Medical Research Council data security policies.

CORE VARIABLES - BASELINE (Q1-28)

A) RANDOMISATION AND PATIENT CHARACTERISTICS (Q1-9)

1. **Your patient identifier** *(preferably specifying uniquely which trial as well as which patient)*
2. **Date of randomisation** *(specify your format for dates [in your covering document])*
3. **Allocated treatment** *(specify your codes)*
4. **Age** at randomisation *(years)* **<u>NB Here & everywhere else, leaving an item Blank means Not Known</u>**
5. **Height** at randomisation *(m)*
6. **Weight** at randomisation *(kg)*
7. **Menopausal status** at randomisation *(1=pre-, 2=peri-, 3=postmenopausal with intact ovaries & uterus, 4=ovarian ablation, 5=hysterectomy, 6=both [ie, 4 and 5])*
8. **Did chemotherapy cause apparently permanent cessation of menses?** *(1=no/not applicable, 2=yes)*
9. **Would your group's preferred analyses exclude this patient?** NB A few trial patients may be randomised in error, otherwise ineligible, lost with no follow-up, unevaluable or withdraw consent. *(1=no known reason for exclusion, 2=yes [specify main reason(s) for preferring exclusion, if known])*

B) SURGICAL DETAILS (Q10-12; OR, DEFINE AND USE YOUR OWN CODES)

10. **Date of surgery** *Please provide date patient received surgery if known.*
11. **Breast surgery** *(1=none, 2=only lumpectomy or wide local excision, 3=quadrantectomy or sector resection, 4=partial mastectomy, 5=simple or total mastectomy, 6=radical mastectomy)*
12. **Axillary surgery** *(1=none, 2=sentinel node biopsy only, 3=axillary sampling, 4=surgical clearance of less than levels I & II, 5=full clearance of axillary levels I & II, 6=clearance of more than levels I & II, 7=axillary clearance, but levels cleared unspecified)*

C) NODAL STATUS (Q13-14; OR, USE YOUR OWN CODES [EG, TNM])

13. **Sentinel node biopsy** *(1=not done, 2=done and negative for cancer, 3= isolated tumour cells [≤0.2 mm], 4=micrometastasis, 5=macroscopic nodal deposit [>2 mm], 6=positive, size unknown)*
14. **Axillary status** *(specify codes, or: 1=N- histologically, 2=N- other/unknown method, 3=1-3 positive nodes, 4=4-9 [or 4+] positive, 5=10+ positive, 6=N+ histologically, unknown number, 7=N+ other/unknown method)*

D) TUMOUR CHARACTERISTICS (Q15-19; OR, USE YOUR OWN CODES [EG, TNM])

15. **Method first detected** *(1=mammographic screening, 2=incidental, 3=symptomatic, 4=other)*
16. **Laterality** *(1=left, 2=right, 3=bilateral)*
17. **Pathological grade** *(1=well differentiated, 2=moderately,3=poorly)*
18. **Histological type** *(1=invasive, not otherwise specified, 2=ductal, 3=lobular, 4=other, 5=mixed)*
19. **Tumour diameter in mm:** largest diameter of excised primary *(mm)*

E) RECEPTOR STATUS (Q20-28; OR, USE YOUR OWN CODES)

20. **Summary of Estrogen Receptor (ER) status of primary tumour** *(1=ER-poor, 2=ER+, 3=ER++ [define in cover document, unless ER-poor is <10 fm/mg and ER++ is ER definitely ≥100 fm/mg])*
21. **Quantitative ER measurement** *(measured in central/reference lab if possible, otherwise best available)*
22. **Units for ER** *(1=fm/mg, 2=% +ve by IHC, 3=Allred score [category score], 4=H-score, 9=other [specify]*)

23. **Summary of Progesterone Receptor (PR) status of primary tumour** *(1=PR-poor, 2=PR+, 3=PR++ [define in cover document, unless PR-poor is <10 fm/mg and PR++ is PR definitely ≥100 fm/mg])*
24. **Quantitative PR measurement** *(done in central/reference lab if possible, otherwise best available)*
25. **Units for PR** *(coded as Q21)*

26. **Summary of HER2 status of primary** *(1=negative/normal, 2=positive/over-expressing)*
27. **Quantitative HER2 measurement** *(done in central/reference lab if possible, otherwise best available)*
28. **Units for HER2** *(1=IHC [% staining], 2=IHC score [0, 1+, 2+, 3+], 3=FISH [# copies], 4=FISH [HER2:CEP17 ratio], 5=CISH [# copies], 6=CISH [HER2:CEP17], 9=other [please specify])*

CORE VARIABLES – FOLLOW-UP (Q29-47)

F) ADJUVANT TREATMENT (Q29-33; OR, USE YOUR OWN CODES)

29. **Chemotherapy given** *(1=No, 2=Yes, 3=Unknown)*
30. **Date of start of endocrine therapy (***Please provide date patient started endocrine therapy if known, else date of surgery.)*
31. **Date Endocrine therapy stopped**
32. **Any substantial deviation from trial treatment allocation (before any breast cancer recurrence)?** *(1=no, 2=never started, 3=discontinued, 4=switched to opposite trial group, 5=other [specify])*
33. **Date of first such deviation from allocated treatment** *(ignore deviations after recurrence)*

G) CANCER RECURRENCE AND SECOND CANCERS (Q34-44; OR, USE YOUR OWN CODES)

34. **Any recurrence of invasive breast cancer (ie, locoregional, contralateral or distant)?** NB Includes any occurrence of new ipsilateral or contralateral breast cancer *(1=no, 2=yes)*
35. **<u>If no:</u> Date patient last known to be free of such recurrence; <u>If yes:</u> Date of first such recurrence**

36. **Site of first distant recurrence (ie, possibly distant; not just locoregional/contralateral)** *(1=no distant recurrence, 2=recurrence, unknown if distant, 3=distant recurrence, unknown/multiple sites, 4=only in distant soft tissue, 5=only in distant nodes, 6=only in bone, 7=only visceral, 8=only in CNS)*
37. **Date of first distant recurrence** NB Locoregional recurrence can precede first distant recurrence

38. **Site of first locoregional recurrence** *(1=no locoregional recurrence recorded, 2=multiple or unspecified locoregional sites 3=only in breast [new or recurrent cancer] or chest wall, 4=only in axilla, 5=only in other locoregional nodes [eg, supraclavicular or internal mammary])*
39. **Date of first locoregional recurrence**

40. **Contralateral breast cancer?** *(1=no, 2=yes: new invasive cancer thought to have arisen during follow-up in the contralateral breast)*
41. **Date of first contralateral breast cancer**

NB If patient had more than one second malignancy during follow-up, <u>repeat</u> variables 38-40 for each.

42. **Site of any second malignancy *[except breast cancer]* during follow-up** *(Describe ALL sites. Use and specify your own codes; if you use ICD codes specify revision, eg ICD-9 or ICD-10)*
43. **Date of this second malignancy**
44. **MIGHT this have been a breast cancer metastasis?** *(1=no, 2=possibly/not yet certain [eg, possible lung, liver, bone or brain metastasis: please do not report definite breast metastases as second cancers])*

H) SURVIVAL (Q45-47)

45. **Is patient known to have died?** *(1=no, 2=yes)*
46. **<u>If no:</u> Date patient last known to be alive; <u>If yes:</u> Date of death**
47. **<u>If yes:</u> Cause of death** *(use and specify your own codes; if you use ICD codes specify which version, eg ICD-9 or ICD-10)*

ADDITIONAL VARIABLES (Q48-58)

I) ADDITIONAL TUMOUR MARKER DATA (Q48-56; OR, USE YOUR OWN CODES)

Note: If tests of gene expression or special tests of IHC quantitation were done on the excised primary then please send a separate file in your own format with the fully detailed set of results on each individual.

48. Summary of gene-expression status of primary tumour *(1=low risk, 2=intermediate risk, 3=high risk):* NB Please also provide the fully detailed gene expression results for each patient as a separate dataset.

49. Quantitative gene-expression prognostic score *(best available single numerical measure)*

50. Prognostic score used to quantify gene expression profile *(use own code, or: 1=OncotypeDx prognostic score, 2=Mammaprint prognostic score, 9=other [please specify])*

51. Summary of Topo-isomerase II alpha (TOPO2A) status of primary tumour *(1= normal [ie, no gene over-expression or deletion], 2=positive/over-expressing, 3=deleted)*

52. Quantitative TOPO2A measurement *(done in central/reference laboratory if possible)*

53. Units for TOPO2A *(1=IHC [% staining], 2=IHC score [0, 1+, 2+, 3+], 3=FISH [number of copies], 4=FISH [TOPO:CEP17 ratio], 5=CISH [# copies], 6=CISH [TOPO:CEP17], 9=other [please specify])*

54. Summary of Proliferation Index of primary tumour *(1=low, 2=intermediate, 3=high)*

55. Quantitative Proliferation Index *(best available single numerical measure, in central/ ref lab if possible)*

56. Factor measured for Proliferation Index *(1=S-phase fraction [%], 2=thymidine labelling index [%], 3=Ki-67 by IHC [% staining], 9=other [please specify]*)

J) BONE FRACTURES AND CARDIOVASCULAR EVENTS BEFORE RECURRENCE (Q57-58; OMIT IF NOT SOUGHT)

Some trial treatments may cause or prevent bone fractures or cardiovascular events. Please describe all such events (eg, hip fracture, spinal fracture, myocardial infarction, stroke, pulmonary embolus, episode of cardiac failure) if, but only if, such events were sought and recorded systematically for the trial.

If more than one bone fracture or cardiovascular event was recorded, repeat variables 55-56 for each.

57. Nature of event *(use your own codes; if you use ICD codes, specify which version, eg ICD-9 or ICD-10, and if you use CTC Adverse Event codes, please specify version number, eg CTCAE-3 or CTCAE-4)*

58. Date of event

P92-94: Search strategies

Assessment of treatment effects in EBCTCG meta-analyses involves identification of all available randomised data, wherever possible, including not only published but also unpublished trial results (with follow-up updated, where records permit, beyond the last available publication). The searches and updating are both important because trial results that do not show significant treatment effects initially, or that become less promising with longer follow-up, may be less likely to be published, or have updates published, than other trial results.

To achieve unbiased ascertainment of the randomised evidence, the EBCTCG secretariat remains in regular contact with all known trials that still have an active investigator (or an active successor), and has for decades been seeking both published and unpublished trials by interactions with all known trialists as well as by electronic searches and scans of potentially relevant reference lists. Hence, the unbiasedness of the meta-analyses in EBCTCG reports depends not on whether there would have been *publication* bias if the EBCTCG dataset was restricted to published data (for it has no such restriction), but whether, despite all efforts, any material *ascertainment* bias remains.

Formal statistical tests for publication bias or ascertainment bias are so weak that appreciable bias might escape them, scientifically reliable assessment of the potential for such bias should chiefly be based on an understanding of EBCTCG procedures for identifying trials and obtaining and updating the results according to the randomly allocated treatment (with no potentially biased exclusions, regardless of what the trial publications excluded). These EBCTCG methods are described below.

The EBCTCG secretariat request regular data updates from trialists, and include the latest available data in meta-analyses. Where only certain trial arms or patient groups of a particular trial are relevant to a given question, they are also included.

The need to include all available eligible randomised data has led to the development of comprehensive methods of identifying published and unpublished trials. The EBCTCG has existed for more than 30 years and the methods for identifying studies have developed over this period. The search strategy is comprehensive and labour-intensive. It involves a team of six research assistants who search databases and conference proceedings, and assess the search results.

i) Database searches

The EBCTCG secretariat has been performing methodical literature searches since the 1980s and the search strategy has developed over this period. Initially trials were sought through

literature searches, contact with research groups worldwide and with pharmaceutical companies. Later, this was extended over a wider range of databases, trial registers, conference proceedings and adverts/surveys.

In recent years database searches of MEDLINE, Embase and the Cochrane Library are conducted on a regular basis using the following search terms:

1 random$.af.
2 ((singl$ or doubl$ or trebl$ or tripl$) adj (blind$ or mask$)).af.
3 (phase III or phase 3 or phase IV or phase 4).af.
4 controlled clinical trial$.af.
5 placebo$.af.
6 (meta?analys$ or (meta adj1 analys$)).af.
7 1 or 2 or 3 or 4 or 5 or 6
8 exp breast neoplasms/
9 (breast$ adj5 (neoplas$ or carcinom$ or cancer$ or tumor$ or tumour$)).af.
10 (advanced or metastatic or inoperable).ti.
11 locally advanced.af. or (neoadjuvant or adjuvant or early or operable).ti.
12 10 not 11
13 (8 or 9) not 12
14 7 and 13
15 Human$/
16 Animal$/
17 16 not (15 and 16)
18 14 not 17
19 18

Search results are screened manually. Further information is sought for any search results which indicate a possible eligible trial.

ii) Conference proceedings

Lists of titles and abstracts of cancer conferences are searched systematically. Research assistants read all titles and abstracts from each conference listed below. Further information is sought for any search results which indicate a possible eligible trial.

- San Antonio Breast Cancer Congress
- American Society of Clinical Oncology
- European Cancer Organisation
- European Society of Medical Oncology

iii) EBCTCG Steering Committee

These searches are supplemented by input from breast cancer trialists' around the world. The EBCTCG's Steering Committee includes 56 individuals from 17 countries. The committee meet annually to discuss progress. They review lists of included trials in ongoing meta-

analyses. Committee members are invited to consider whether any eligible trials have been omitted and, if so, to provide contacts for any missing trials.

iv) EBCTCG Collaborative Group

The EBCTCG Collaborative group includes >500 individuals representing >200 trial groups from >30 countries. Each draft manuscript is circulated to the collaborative group before submission, giving further opportunity for any unpublished trials to be identified.

These methods have led to the compilation of a database of some 40,000 articles of relevance to EBCTCG overviews (by 1.1.2023), which continues to be populated on a regular basis, with additional targeted updates for any meta-analyses prior to submission for publication.

Methods to assess integrity of individual trial data

For each meta-analysis, a study protocol is developed by the EBCTCG secretariat in conjunction with leads of the trials which may contribute to the analysis. A data request, outlining the variables required, is sent to each contributing trial group. Received data are standardised and added to the EBCTCG database enabling various tests of internal integrity. Data are then checked against published reports and assessed for valid randomisation sequence, balance of baseline variables and follow-up dates across the intervention groups. A summary report is sent back to the trialist, and any queries resolved. Issues relating to the non-availability of data, for example because they are not supplied for unpublished studies, are discussed in each EBCTCG report.
